# Prevalence, antimicrobial susceptibility and plasmid profiling of *Vibrio* spp. isolated from cultured groupers in Peninsular Malaysia

**DOI:** 10.1186/s12866-019-1624-2

**Published:** 2019-11-11

**Authors:** Nor Zulkiply Amalina, Silvaraj Santha, Dzarifah Zulperi, Mohammad Noor Azmai Amal, Mohd Termizi Yusof, Mohd Zamri-Saad, Md Yasin Ina-Salwany

**Affiliations:** 1 Laboratory of Marine Biotechnology, Institute of Bioscience, Universiti Putra Malaysia, 43400 Serdang, Selangor Malaysia; 2 Department of Plant Protection, Faculty of Agriculture, Universiti Putra Malaysia, 43400 Serdang, Selangor Malaysia; 3 Department of Biology, Faculty of Science, Universiti Putra Malaysia, 43400 Serdang, Selangor Malaysia; 4 Department of Microbiology, Faculty of Biotechnology and Biomolecular Sciences, Universiti Putra Malaysia, 43400 Serdang, Selangor Malaysia; 5 Department of Veterinary Laboratory Diagnosis, Faculty of Veterinary Medicine, Universiti Putra Malaysia, 43400 Serdang, Selangor Malaysia; 6 Department of Aquaculture, Faculty of Agriculture, Universiti Putra Malaysia, 43400 Serdang, Selangor Malaysia

**Keywords:** *Vibrio*, Grouper, Multiple antibiotic resistance, Plasmid, Chromosome, Malaysia

## Abstract

**Background:**

Numerous prevalence studies of *Vibrio* spp. infection in fish have been extensively reported worldwide, including Malaysia. Unfortunately, information on the prevalence of *Vibrio* spp. in groupers (*Epinephelus* spp.) is limited. In this study, groupers obtained from nine farms located at different geographical regions in Malaysia were sampled for the presence of pathogenic *Vibrio* spp. and their susceptibility profiles against seven antibiotics.

**Results:**

Out of 270 grouper samples, 195 (72%) were detected with the presence of *Vibrio* spp. *Vibrio communis* showed highest prevalence in grouper (28%), followed by *V. parahaemolyticus* (25%), *V. alginolyticus* (19%), *V. vulnificus* (14%), *V. rotiferianus* (3%), *Vibrio* sp. (3%), *V. campbellii* (2%)*, V. mytili* (2%)*, V. furnissii* (2%)*, V. harveyi* (1%)*, V. tubiashii* (1%)*, V. fluvialis* (0.3%) and *V. diabolicus* (0.3%)*.* Assessment on the antibiotic susceptibility profiles of the *Vibrio* spp. revealed that majority of the isolates were susceptible to tetracycline, streptomycin, erythromycin and bacitracin, but resistance to ampicillin, penicillin G and vancomycin. The mean MAR index of the *Vibrio* isolates was 0.51, with 85% of the isolates showed MAR index value of higher than 0.2. Results indicate that the *Vibrio* spp. were continuously exposed to antibiotics. Furthermore, the plasmid profiles of *Vibrio* spp. showed that 38.7% of the isolates harbored plasmid with molecular weight of more than 10 kb, while 61.3% were without plasmid. During curing process, *Vibrio* spp. lost their plasmid, but remained resistant to ampicillin, penicillin G, bacitracin and vancomycin while a few isolates remained resistant to erythromycin, streptomycin and tetracycline. The results suggested that the resistance to antibiotics in isolated *Vibrio* spp. might be due to chromosomal and plasmid borne.

**Conclusions:**

This study demonstrates the prevalence of *Vibrio* spp. in groupers and the distribution of multidrug resistance strains that could be of concern to the farmers in Malaysia. In addition, data from this study can be further used in fish disease management plan.

## Background

Aquaculture is a growing sector for food production, representing 47% of the total 171 million metric tons of fish supplies worldwide [22]. However, efficient fish production was hindered by health problems that cause mortalities and significant stock losses [6, 65]. Disease outbreaks following infections by pathogenic bacteria have been reported among various cultured marine fish such as grouper (*Epinephelus* spp.), pompano (*Trachinotus blochii*) and Asian seabass (*Lates calcarifer*) [3, 15, 44, 60].

Generally, molecular methods were used for the identification of bacteria species based on the specific molecular markers. *pyrH* genes is one of the common markers used in PCR and multi-locus sequence analysis (MLSA) to determine the taxonomic diversity of *Vibrio* spp. It is a housekeeping gene that encodes for Uridylate kinase (UMP kinase) and plays an important role for survival and growth of *Vibrio* [34]. Various studies have reported on the efficiency of the *pyrH* gene in identification and differentiation of *Vibrio* spp. [48, 54, 55, 63]. In addition, the *pyrH* gene has high discriminatory power at species level due to slight overlapped of intraspecies and interspecies distance [48, 59].

Antibiotics are the first line of treatment for bacterial infection and are frequently used by farmers, especially the wide spectrum antibiotics [8, 53]. Antibiotic is a chemical substance that has the capacity as therapeutic and prophylactic activities against growth of bacteria and is safe to the host [9]. In Malaysia, antibiotics are used both as prophylaxis and therapy in cultured fish. They are administered via feed additives or immersion baths [25].

Unfortunately, extensive use of antibiotics encouraged the emergence of antibiotic resistance bacterial strains [45]. According to Kumar et al. [32], occurrence of antibiotic resistance bacteria was common in areas where antibiotics were frequently used such as in outbreaks area. Letchumanan et al. [36] reported that the resistance level of pathogenic *Vibrio* spp. toward antibiotics used in aquaculture was increasing every year. In fact, some antibiotics have been reported to be ineffective in controlling bacterial pathogens [20].

When bacteria are overly exposed to antibiotics, they tend to acquire antimicrobial resistance genes, either via horizontal gene transfer or vertical gene transfer [57]. Thus, plasmid is one of the mediators that plays an important role in spreading of resistance genes since it consists most of the genetic determinants of antibiotic resistance. In fact, correlation between plasmid and antibiotic resistance among *Vibrio* spp. has been reported [37, 42, 68]. Similarly, several studies have shown that the antibiotic resistance genes were actually located in the bacterial chromosomal DNA [26, 40, 41].

Plasmid curing is a method that allows determination mode of antibiotic resistance mediation by eliminate bacteria plasmid. Chemical agents such as ethidium bromide (EtBr), sodium dodecyl sulphate (SDS) and acridine orange (AO) are commonly used to cure the plasmid [39, 50]. The mechanism involves inhibition of plasmid replication by intercalation of the chemical agent into the plasmid leading to unwinding of the super helical plasmid to form the relaxed molecule and subsequently changed to become a linear or open circular plasmid [58]. After the curing process, changes in the antibiotic resistance profile indicate a plasmid mediated, while unchanged profile indicated chromosomal mediated [36].

Even though studies on the prevalence and assessment of antibiotic resistance profile in Malaysia have been carried out, most were focused on *Vibrio parahaemolyticus, V. vulnificus, V. alginolyticus* and *V. cholerae* isolated from while leg shrimp, Asian seabass, tilapia and oyster, but not on grouper [23, 36, 44, 52]. Thus, this study aims to provide important information regarding prevalence, antibiotic resistance patterns and plasmid profiling of *Vibrio* spp. isolated from cultured groupers in Peninsular Malaysia.

## Results

### Clinical signs and gross lesions of groupers

A total of 150 (56%) of the 270 groupers were collected from nine farms were healthy and the remaining 120 (44%) were unhealthy due to observed clinical signs and gross lesions. Observations on the diseased groupers in all farms showed similar clinical abnormalities of vibriosis such as lethargy, loss of appetite and swimming on the surface of water. Based on 270 collected groupers, 107 (40%) had external and internal lesions suspecting of vibriosis, 57 (21%) had external lesions only, 29 (10%) had internal lesions only and 77 (29%) were asymptomatic.

**Fig. 1:**
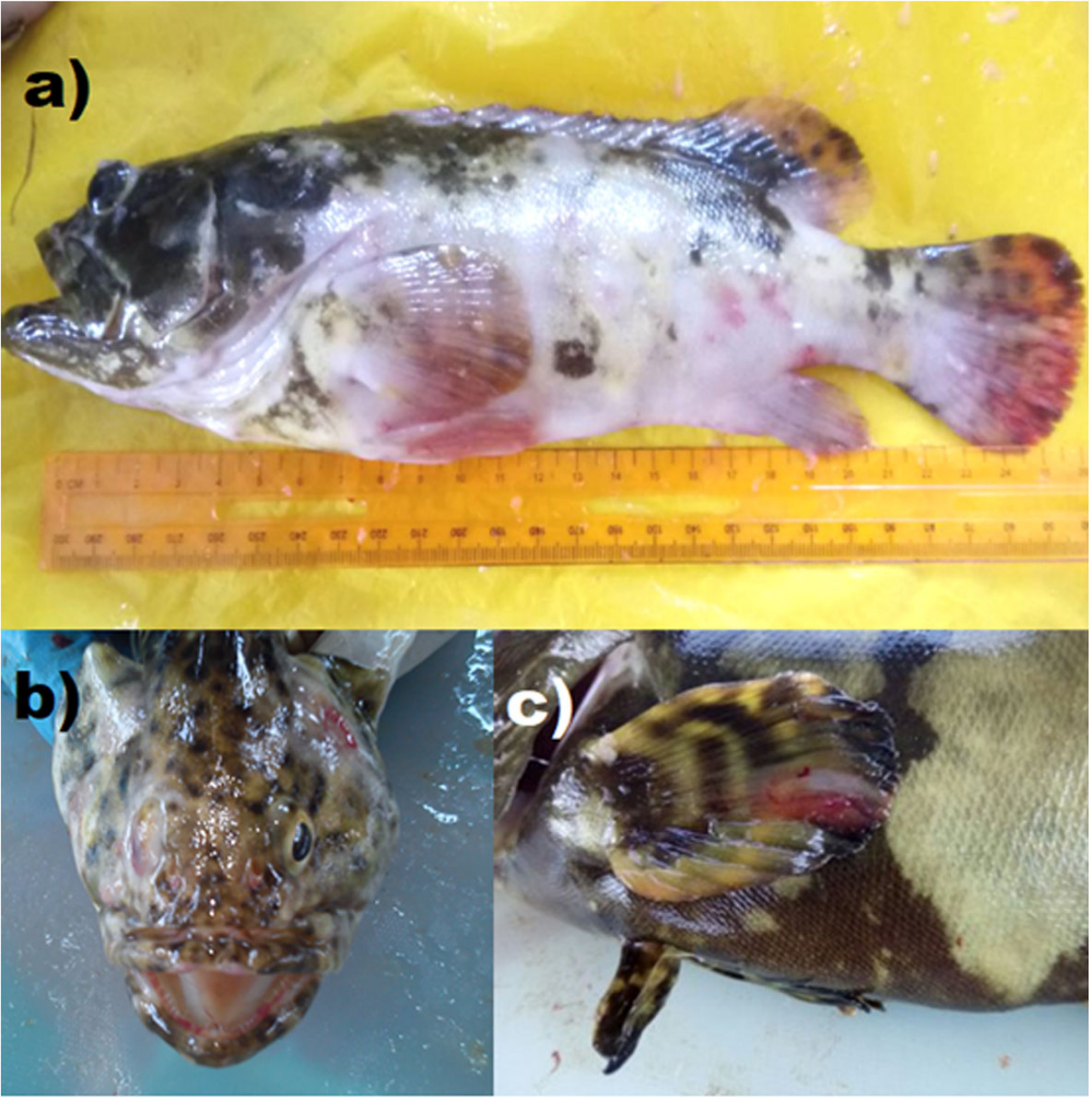
External lesions of vibriosis observed on groupers including; **a** discoloration with lesion on the skin and fins, **b** loss on the left side of fish eye, **c** haemorrhagic on the pectoral fin

**Fig. 2:**
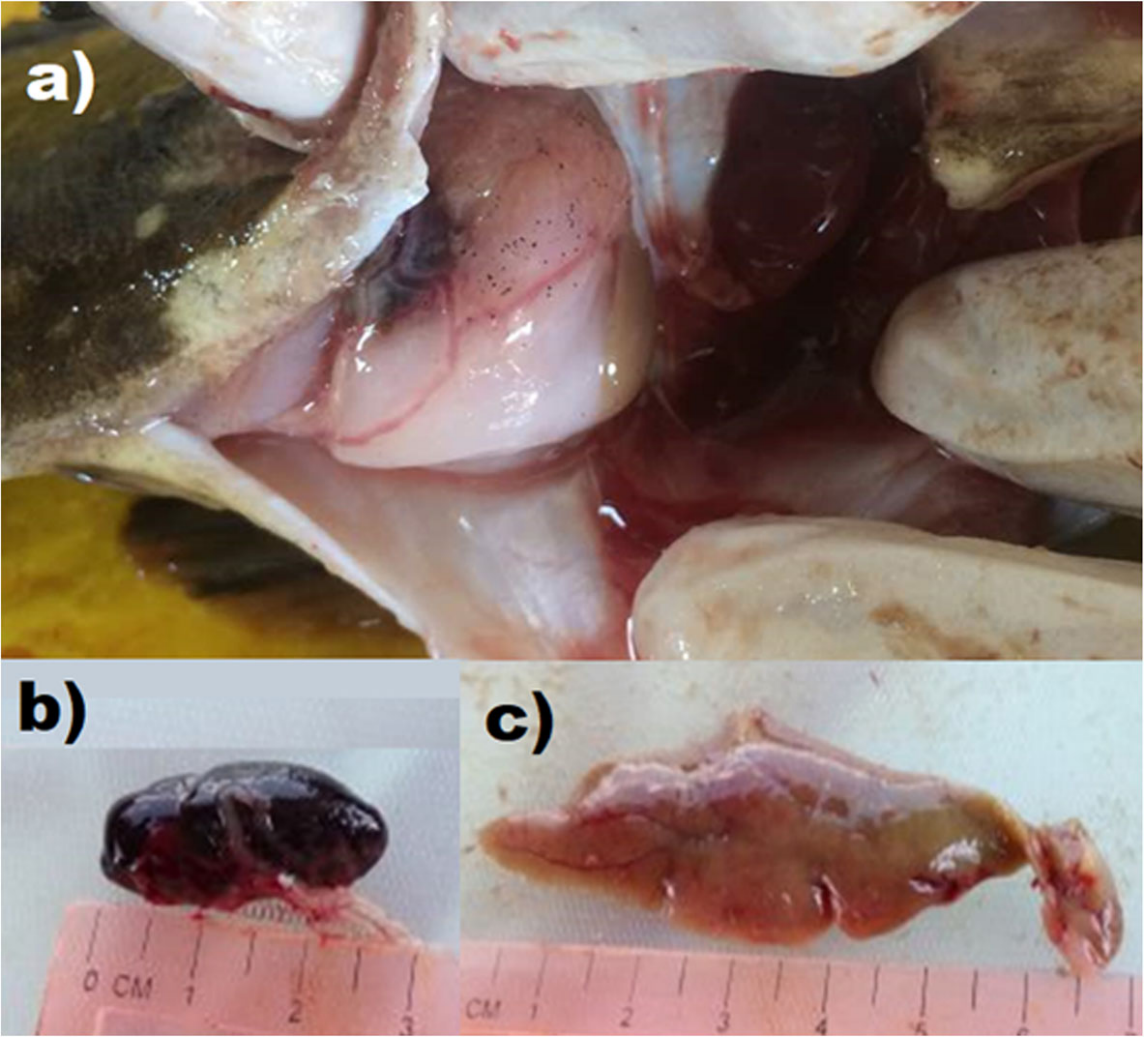
Internal appearance of grouper infected by *Vibrio* spp. showing; **a** blood-tinged ascetic fluid, **b** enlargement of spleen (splenomegaly), **c** pale discolouration of liver

The external lesions of vibriosis observed included ulcers on the skin, fins and mouth, corneal opacity, pop-eye and loss of one eyes. In advanced stage, affected fish showed discoloration or haemorrhagic skin (Fig. 1). Approximately 85% of the unhealthy and 30% of the healthy groupers had the external lesions. Upon dissection, examinations of the internal organs revealed 70% of the groupers had pale liver, 28% had soft and enlarged spleen, 14% with excessive ascetic fluids and less than 5% developed haemorrhagic liver and kidney with rotten organs (Fig. 2).

### Prevalence of *Vibrio* spp.

A total of 380 suspected *Vibrio* strains were isolated based on the color of the colonies (green or yellow) that appeared on the thio-sulphate citrate bile salt sucrose (TCBS) agar. They were isolated from 195 (72%) out of 270 groupers collected from nine farms in Peninsular Malaysia. Among them, 67 (18%) isolates were from Pulau Langkawi, Kedah, 66 (17%) from Pulau Ketam, Selangor, 58 (15%) from Kuala Gula, Perak, 54 (14%) from Port Dickson, Negeri Sembilan, 31 (8%) from Kota Bharu, Kelantan, 27 (7%) from Banting, Selangor, 27 (7%) from Kukup Laut, Johor, 26 (7%) from Jerteh, Terengganu, and 24 (6%) from Bukit Mertajam, Penang (Table 1).

**Table 1:** The prevalence of *Vibrio* spp. isolated from groupers in each farm

State	Sampling area	No. of groupers infected with *Vibrio*	No. of *Vibrio* strains isolated	Organs	Species of *Vibrio* based on phylogenetic tree analysis
Kedah	Pulau Langkawi	29/30	67	Liver: 23, Spleen: 23, Kidney: 21	*V. communis* (45), *V. mytili* (7), *V. parahaemolyticus* (5), *V. vulnificus* (5), *V. rotiferianus* (3), *V. alginolyticus* (1), *Vibrio* sp. (1)
Penang	Bukit Mertajam	15/30	24	Liver: 2, Spleen: 14, Kidney: 8	*V. communis* (11), *V. vulnificus* (5), *V. tubiashii* (4), *V. harveyi* (2), *Vibrio* sp. (2)
Perak	Kuala Gula	25/30	58	Liver: 21, Spleen: 20, Kidney: 17	*V.parahaemolyticus* (22), *V. alginolyticus* (21), *V. campbellii* (6), *V. vulnificus* (5), *V. communis* (3), *V. rotiferianus* (1)
Kelantan	Kota Bharu	21/30	31	Liver: 13, Spleen: 9, Kidney: 9	*V.parahaemolyticus* (19), *V. campbellii* (2), *V. harveyi* (2), *V. mytili* (1), *V. alginolyticus* (1), *Vibrio* sp. (6)
Terengganu	Jerteh	17/30	26	Liver: 9, Spleen: 11, Kidney: 6	*V. communis* (14), *V. vulnificus* (11), *Vibrio* sp. (1)
Selangor	Pulau Ketam	24/30	66	Liver: 19, Spleen: 23, Kidney: 24	*V. alginolyticus* (34), *V.parahaemolyticus* (18), *V. communis* (12), *V. diabolicus* (1), *Vibrio* sp. (1)
	Banting	19/30	27	Liver: 11, Spleen: 8, Kidney: 8	*V.parahaemolyticus* (15), *V. communis* (9), *V. vulnificus* (3)
Negeri Sembilan	Port Dickson	28/30	54	Liver: 25, Spleen: 17, Kidney: 12	*V. alginolyticus* (13), *V. vulnificus* (11), *V.parahaemolyticus* (10), *V. furnissii* (7), *V. communis* (2), *V. fluvialis* (1), *V. campbellii* (1)
Johor	Kukup Laut	17/30	27	Liver: 6, Spleen: 16, Kidney: 5	*V. vulnificus* (12), *V. rotiferianus* (7), *V. parahaemolyticus* (6), *V. communis* (1), *V. harveyi* (1)
		195/270 (72%)	380	Liver: 129 (34%), Spleen: 141 (37%), Kidney: 110 (29%)	

**Fig. 3:**
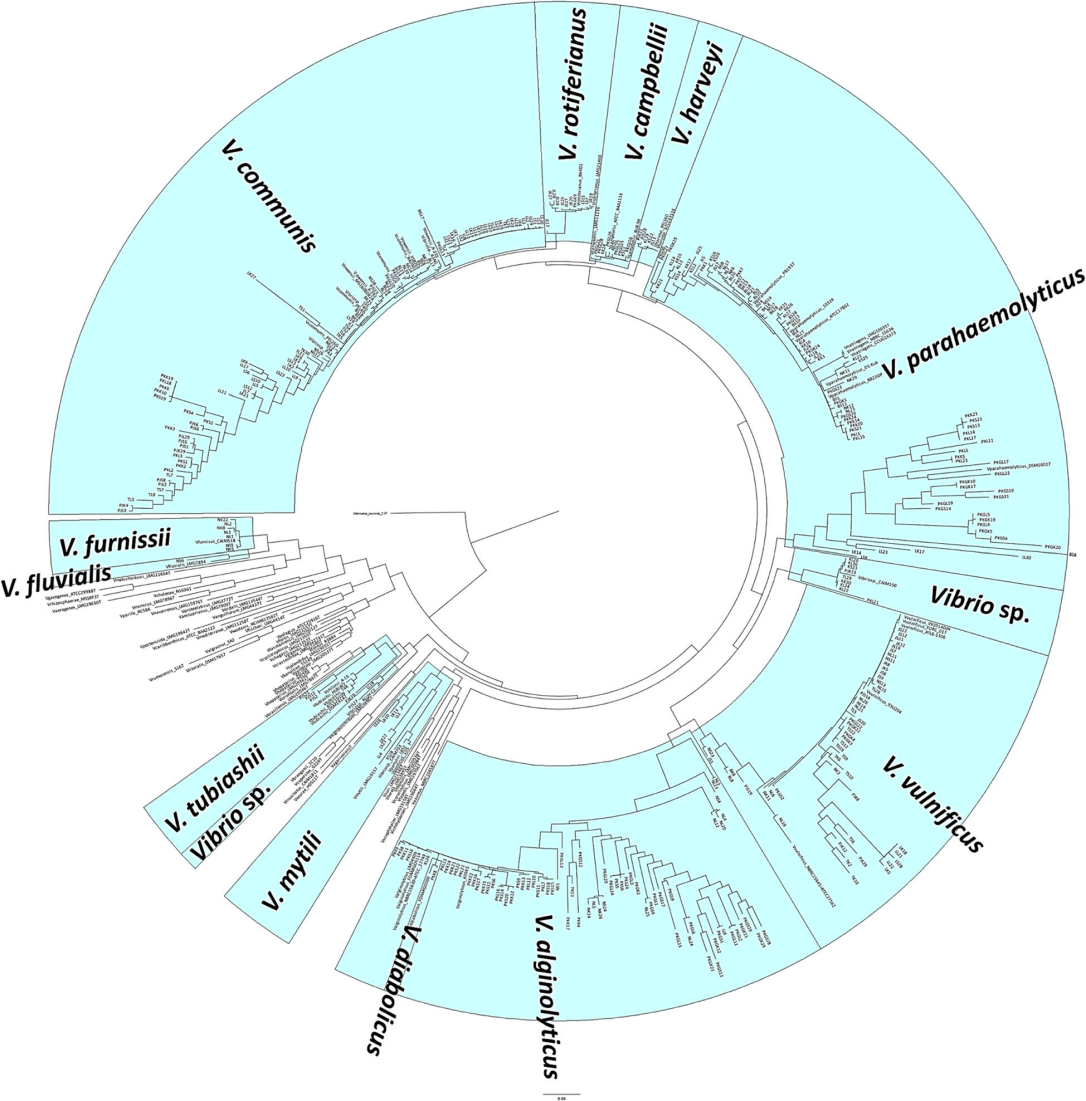
Phylogenetic tree of *pyrH* gene sequences was inferred by using the ML method based on GTR + G model. Bootstrap values greater than 50% confidences were shown at branching points (percentages of 1000 replicates). The analysis involved 380 *Vibrio* isolate sequences and 102 reference sequences of *Vibrio* spp. (GenBank database). *P. damsela* subsp. *piscicida* strain D121 have been added as outgroup. Clusters highlighted in blue comprise the well-identified strains

All 380 isolates were Gram negative with biochemical characteristics of *Vibrio* spp. (Table 2). They were also *pyrH-*positive, producing the 440 bp band. Using phylogenetic analysis of the *pyrH* sequences, 13 *Vibrio* species were identified (Table 3, Fig. 3). The sequences reported have been deposited in the GenBank nucleotide sequence databases (accession numbers MN253135-MN253478) (See Additional file 1 for details).

**Table 2:** Identification of *Vibrio* spp. based on culture method, Gram stain and biochemical tests

Species	No. of isolates	TCBS	Gram stain	LDC	ONPG	Oxidase	Catalase	TSI
*V. communis*	106	Yellow	Gram negative	+	–	+	+	A/A, no gas, no H_2_S
*V. parahemolyticus*	95	Green	Gram negative	+	–	+	+	A/A, no gas, no H_2_S; K/A, no gas, no H_2_S
*V. alginolyticus*	70	Yellow	Gram negative	+	–	+	+	A/A, no gas, no H_2_S
*V. vulnificus*	52	Green	Gram negative	+	–	+	+	A/A, no gas, no H_2_S
*V. rotiferianus*	11	Green	Gram negative	–	–	+	+	A/A, no gas, no H_2_S
*V. campbellii*	9	Green	Gram negative	–	–	+	+	A/A, no gas, no H_2_S
*V. mytili*	8	Green	Gram negative	–	–	+	+	A/A, no gas, no H_2_S
*V. furnissii*	7	Yellow	Gram negative	–	–	+	+	A/A, no gas, no H_2_S
*V. harveyi*	5	Yellow	Gram negative	+	–	+	+	A/A, no gas, no H_2_S
*V. tubiashii*	4	Yellow	Gram negative	–	–	+	+	A/A, no gas, no H_2_S
*V. fluvialis*	1	Green	Gram negative	–	–	+	+	A/A, no gas, no H_2_S
*V. diabolicus*	1	Yellow	Gram negative	–	–	+	+	A/A, no gas, no H_2_S
*Vibrio* sp.	11	Yellow	Gram negative	–	–	+	+	A/A, no gas, no H_2_S

*TCBS* thio-sulphate citrate bile salt sucrose, *LDC* lysine decarboxylase, *ONPG* O-nitrophenyl-beta-D-galactosifase, *TSI* triple sugar iron, +: Positive, −: Negative, *A/A* Acidic slant/Acidic butt, *K/A* Alkaline slant/Acidic butt

**Table 3:** List of reference sequences that related with *Vibrio* isolated from grouper in Malaysia

No	Species	No. of isolates	Reference sequences obtained from the GenBank database		
			Accession no	*Vibrio* spp.	Strain
1	*V. communis*	106	KC871657.1	*V. communis*	PEL26G
			KC871668.1	*V. communis*	PEL4D
			JX401895.1	*V. communis*	10G9
			GU078692.1	*V. communis*	R-40901
2	*V. parahemolyticus*	95	CP022243.1	*V. parahaemolyticus*	PB1937
			CP026041.1	*V. parahaemolyticus*	10,329
			CP014046.2	*V. parahaemolyticus*	ATCC 17802
			CP006004.1	*V. parahaemolyticus*	O1:Kuk
			CP003972.1	*V. parahaemolyticus*	BB22OP
			MG932062.1	*V. parahaemolyticus*	DSM 10027
3	*V. alginolyticus*	70	JN408273.1	*V. alginolyticus*	ATCC 17749
			CP014045.1	*V. alginolyticus*	FDAARGOS 114
			CP017919.1	*V. alginolyticus*	K09K1
			GU266285.1	*V. alginolyticus*	LMG 4409
4	*V. vulnificus*	52	CP019320.1	*V. vulnificus*	VV2014DJH
			CP012881.1	*V. vulnificus*	ATCC 27562
			CP012739.1	*V. vulnificus*	FORC_017
			CP014049.2	*V. vulnificus*	ATL6–1306
			CP009261.1	*V. vulnificus*	93 U204
5	*V. rotiferianus*	11	CP018312.1	*V. rotiferianus*	B64D1
			EF596722.1	*V. rotiferianus*	LMG21460
6	*V. campbellii*	9	EF596641.1	*V. campbellii*	LMG11216
			CP006605.1	*V. campbellii*	ATCC_BAA1116
			CP026315.1	*V. campbellii*	BoB-90
7	*V. mytili*	8	GU266287.1	*V. mytili*	LMG19157
8	*V. furnissii*	7	JF316672.1	*V. furnissii*	CAIM 518
9	*V. harveyi*	5	KC871684.1	*V. harveyi*	PEL36D
			CP025537.1	*V. harveyi*	ATCC 43516
10	*V. tubiashii*	4	LN998049.1	*V. tubiashii*	HLBLW2
			CP009345.1	*V. tubiashii*	ATCC19109
			GU186317.1	*V. tubiashii*	74 K
			MG932064.1	*V. tubiashii*	DSM19142
11	*V. fluvialis*	1	JN426808.1	*V.fluvialis*	LMG7894
12	*V. diabolicus*	1	CP014049.1	*V. diabolicus*	FDAARGOS 96
13	*Vibrio* sp.	11	EF394938.1	*Vibrio* sp.	RLUH-CZ
			JF739405.1	*Vibrio* sp.	CAIM 190

The 380 *Vibrio* strains were successfully isolated and identified from 195 groupers. From the phylogenetic analysis, 110 (56%) groupers were infected with one species of *Vibrio,* 77 (39%) were infected with two different species of *Vibrio,* eight (4%) were infected with three different species of *Vibrio*. Of the 380 *Vibrio* strains, 106 (28%) were *V. communis,* 95 (25%) were *V. parahaemolyticus,* 70 (19%) were *V. alginolyticus,* 52 (14%) were *V. vulnificus,* 11(3%) were *V. rotiferianus* and *Vibrio* sp. and less than 3% were *V. campbellii, V. mytili, V. furnissii, V. harveyi, V. tubiashii, V. fluvialis* and *V. diabolicus.* Based on the sampling farms, *V. communis* was the most prominent isolated in Pulau Langkawi (67%), Jerteh (52%) and Bukit Mertajam (46%). *Vibrio parahaemolyticus* was dominant in Kota Bharu (61%) and Banting (56%). *Vibrio alginolyticus* was prominent in Pulau Ketam (52%), while in Kuala Gula farm, *V. parahaemolyticus* and *V. alginolyticus* were prominent at 38 and 36%,*,* respectively*. Vibrio Alginolyticus* (24%)*, V. vulnificus* (20%) and *V. parahaemolyticus* (19%) were prominent in Port Dickson farm. In addition, *V. vulnificus* (44%) was also dominant in Kukup Laut.

### Antimicrobial susceptibility profile

Antimicrobial susceptibility profile of the 380 *Vibrio* strains revealed 369 (97%) isolated were resistant to at least one antibiotic. Ninety-eight (27%) isolates were resistant to four antibiotics, followed by 82 (22%), 67 (18%), 66 (18%), 45 (12%), 10 (3%) and 1 (0.3%) isolates were resistant to 2, 5, 3, 1, 6 and 7 antibiotics, respectively. A total of 11 (3%) isolates were susceptible to all antibiotics tested.

**Fig. 4:**
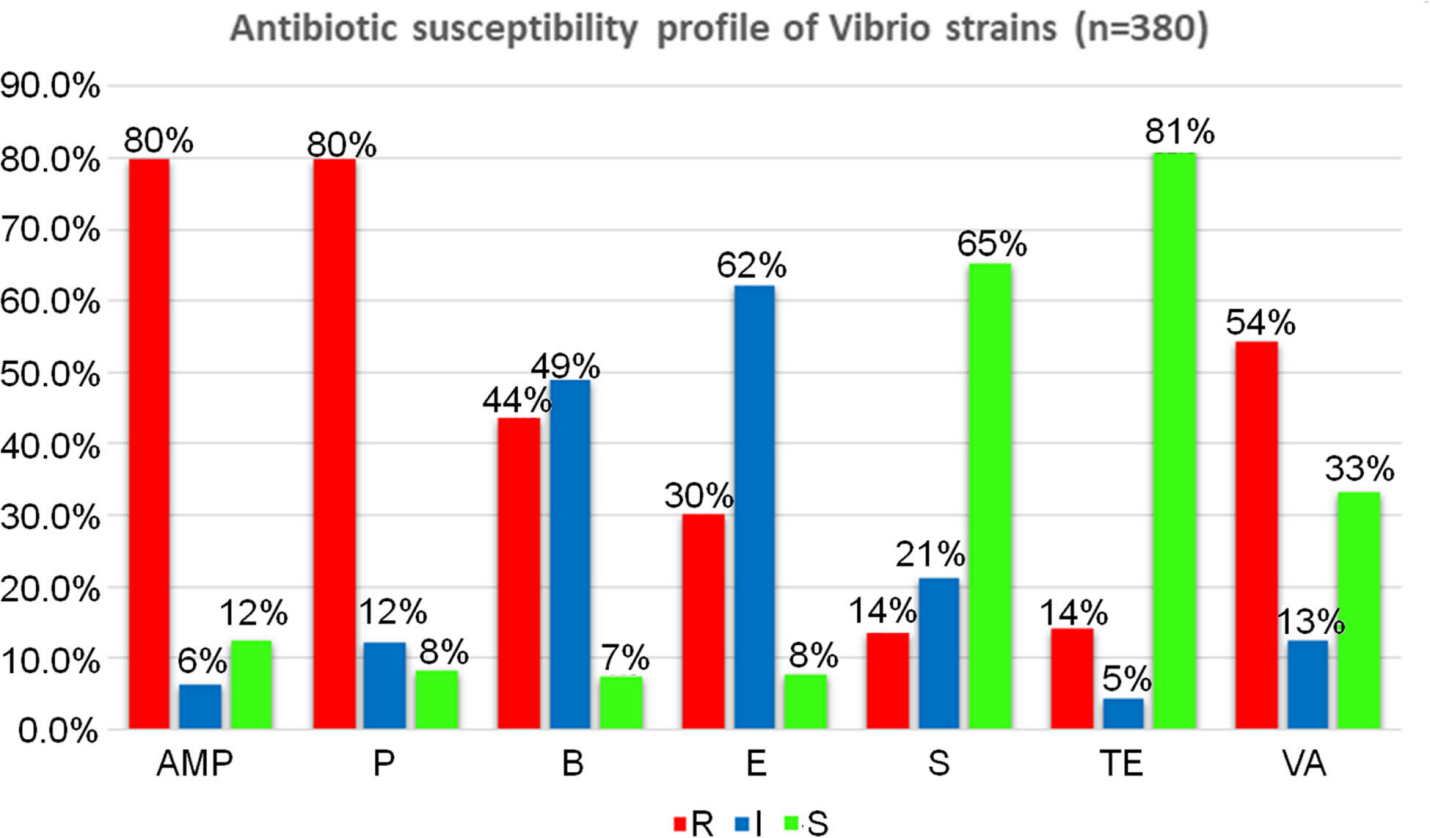
Level of antibiotic resistance profiles to the seven drugs tested. Amp: Ampicillin, P: Penicillin G, B: Bacitracin, E: Erythromycin, S: Streptomycin, TE: Tetracycline, VA: Vancomycin

A total of 303 (82%) *Vibrio* isolates were highly resistant to penicillin G and ampicillin, where 206 (56%), 166 (45%) and 115 (31%) isolates showed moderate resistant to vancomycin, bacitracin and erythromycin, respectively. Meanwhile, 54 (15%) isolates were resistant to tetracycline and 52 (14%) to streptomycin. Besides, 309 (84%) isolates were highly susceptible to tetracycline, 248 (67%) isolates to streptomycin and 126 (34%) isolates to vancomycin (Fig. 4).

### Antimicrobial susceptibility profile of the *Vibrio* spp.

The antimicrobial susceptibility profiles of the 13 identified species of *Vibrio* is summarised in Table 4. Most of the *Vibrio* spp. were highly resistant to ampicillin and penicillin G particularly *V. mytili* (100%), *V. tubiashii* (100%), *V. diabolicus* (100%), *V. fluvialis* (100%), *Vibrio* sp. (100%), *V. furnissii* (71–100%), *V. communis* (92–97%), *V. harveyi* (80%),*V. parahaemolyticus* (77–84%), *V. vulnificus* (64%), *V. alginolyticus* (61–79%) and *V. campbellii* (56%). Surprisingly, only 27% of the *V. rotiferianus* isolates were susceptible to ampicillin and penicillin G.

**Table 4:** Antibiotic resistance profiles based on the *Vibrio* spp.

*Vibrio* species	No of isolates	AMP			P			B			E			S			TE			VA		
		R	I	S	R	I	S	R	I	S	R	I	S	R	I	S	R	I	S	R	I	S
*V. communis*	106	100	5	1	103	2	1	60	43	3	49	49	8	25	46	35	16	1	89	74	14	18
*V. parahaemolyticus*	95	73	7	15	80	7	8	36	53	6	18	71	6	5	9	81	8	6	81	54	6	35
*V. alginolyticus*	70	55	7	8	43	19	8	17	48	5	6	59	5	1	5	64	9	3	58	25	4	41
*V. vulnificus*	52	33	8	11	33	13	6	29	19	4	20	28	4	15	7	30	13	3	36	28	12	12
*V. rotiferianus*	11	3	0	8	3	4	4	4	2	5	10	0	1	0	3	8	1	0	10	7	1	3
*V. campbellii*	9	5	0	4	5	0	4	6	3	0	2	7	0	0	1	8	1	0	8	2	1	6
*V. mytili*	8	8	0	0	8	0	0	0	8	0	7	1	0	0	7	1	0	3	5	7	1	0
*V. furnissii*	7	5	2	0	7	0	0	3	1	3	0	5	2	0	0	7	5	1	1	4	2	1
*V. harveyi*	5	4	1	0	4	1	0	2	2	1	1	3	1	2	0	3	0	0	5	1	2	2
*V. tubiashii*	4	4	0	0	4	0	0	4	0	0	1	3	0	2	0	2	0	0	4	0	0	4
*V. diabolicus*	1	1	0	0	1	0	0	0	1	0	0	1	0	0	0	1	0	0	1	0	0	1
*V. fluvialis*	1	1	0	0	1	0	0	1	0	0	0	0	1	0	0	1	1	0	0	1	0	0
Other *Vibrio* sp.	11	11	0	0	11	0	0	4	6	1	1	9	1	3	2	6	0	0	11	3	5	3

*R* resistance, *I* intermediate, *S* susceptibility, *AMP* ampicillin, *P* penicillin G, *B* bacitracin, *E* erythromycin, *S* streptomycin, *TE* tetracycline, *VA* vancomycin

When tested with bacitracin, more than 50% *V. communis, V. vulnificus, V. tubiashii, V. fluvialis* and *V. campbellii* showed resistance pattern. The remaining eight *Vibrio* spp. showed intermediate and susceptible to bacitracin. In contrast, *V. rotiferianus* (91%) and *V. mytili* (88%) were highly resistant to erythromycin, while the remaining 11 *Vibrio* spp. showed intermediate and susceptible. High resistance of *Vibrio* spp. against vancomycin were observed among *V. fluvialis* (100%), *V. mytili* (88%)*, V. communis* (70%), *V. rotiferianus* (64%), *V. parahaemolyticus* (57%)*, V. furnissii* (57%) and *V. vulnificus* (54%). The other six *Vibrio* spp. (64–100%) showed intermediate and susceptible to vancomycin.

On the other hand, more than 80% isolates of nine *Vibrio* spp. were susceptible to tetracycline including *V. alginolyticus, V. campbellii, V. communis, V. diabolicus, V. harveyi, V. parahaemolyticus, V. rotiferianus, V. tubiashii* and *V. vulnificus.* In addition, 100% of *V. furnissii, V. fluvialis* and *V. diabolicus* were found susceptible to streptomycin, followed by 91% of *V. alginolyticus,* 89% of *V. campbellii,* 85% of *V. parahaemolyticus* and 73% of *V. rotiferianus.*

### Plasmid profiles of *Vibrio* spp.

Among the 380 *Vibrio* isolates tested, 147 (39%) isolates harboured plasmid with molecular weight of more than 10 kb (Table 5) and 98 (67%) of them were resistant to four or more antibiotics. All *V. diabolicus,* 57% of *V. communis,* 46% of *V. rotiferianus,* 40% of *V. harveyi,* 37% of *V. vulnificus,* 34% of *V. parahaemolyticus,* 33% of *V. campbellii* and 29% of *V. alginolyticus* isolates were harboured plasmid. Meanwhile, less than 25% of *V. tubiashii*, *V. furnissii, V. mytili* and *Vibrio* sp. isolates harboured plasmid.

**Table 5:** The antibiotic resistance profile patterns of *Vibrio* spp. before and after plasmid curing

Species	Strain number	Before plasmid curing	No of plasmid	After plasmid curing	No of plasmid
		Antibiotic profiles		Antibiotic profiles	
*V. alginolyticus*	PKGK2	Amp/ E/ VA	1	B	Lost
*V. alginolyticus*	PKGL9, PKGL12, PKS18	Amp/ P	1	Amp	Lost
*V. alginolyticus*	PKS3	Amp/ P	1	Amp/ P	Lost
*V. alginolyticus*	PKL7, PKL13	Amp/ P	1	No resistance	Lost
*V. alginolyticus*	PKGS1, PKS12	Amp/ P/ VA	1	Amp	Lost
*V. alginolyticus*	PKGK21	Amp/ P/ VA	1	Amp/ VA	Lost
*V. alginolyticus*	PKS15	Amp/ P/ VA	1	No resistance	Lost
*V. alginolyticus*	NL3	Amp/ P/ B	1	Amp/ P/ B	Lost
*V. alginolyticus*	PKGL1	Amp/ P/ B/ E	1	Amp/ P	Lost
*V. alginolyticus*	LL6	Amp/ P/ B/ E/ VA	1	Amp/ P	Lost
*V. alginolyticus*	NS4	Amp/ P/ B/ TE/ VA	1	VA	Lost
*V. alginolyticus*	NL5	Amp/ P/ B/ TE/ VA	1	Amp/ P/ B	Lost
*V. alginolyticus*	PKGL15	Amp/ P/ B/ VA	1	No resistance	Lost
*V. alginolyticus*	NK26	Amp/ P/ B/ VA	1	Amp/ P/ B	Lost
*V. alginolyticus*	PKGS29	E/ VA	1	E/ VA	Lost
*V. alginolyticus*	PKK15	P/ VA	1	No resistance	Lost
*V. campbellii*	PKGL21	Amp/ P	1	Amp	Lost
*V. campbellii*	PKGL28	Amp/ P/ B	1	B	Lost
*V. campbellii*	NS30	Amp/ P/ B/ TE/ VA	1	Amp/ P/ B	Lost
*V. communis*	PKS1, PKL18, PKGL20	Amp/ P	1	Amp	Lost
*V. communis*	LS22	Amp/ P/ B/ E/ VA	1	No resistance	Lost
*V. communis*	LK27	Amp/ P/ B/ E/ S/ TE/ VA	1	Amp/ TE	Lost
*V. communis*	LL7, LS10, LS12	Amp/ P/ B/ E/ S/ VA	1	Amp/ P	Lost
*V. communis*	LL9, LK1	Amp/ P/ B/ E/ TE/ VA	1	Amp/ P	Lost
*V. communis*	LS21	Amp/ P/ B/ E/ TE/ VA	1	Amp/ P/ TE	Lost
*V. communis*	TL11, LK4, LK16	Amp/ P/ B/ E/ VA	1	Amp	Lost
*V. communis*	LK3, LS6, LK6, LK9, LL10, LL17, LS1, LS3, LL13, LK13, LL19, LL29, LK29	Amp/ P/ B/ E/ VA	1	Amp/ P	Lost
*V. communis*	LS13	Amp/ P/ B/ E/ VA	1	No resistance	Lost
*V. communis*	PJL2, PJS5	Amp/ P/ B/ S	1	Amp/ P/ S	Lost
*V. communis*	PJK4	Amp/ P/ B/ S	1	Amp/ P/ B	Lost
*V. communis*	PJS8, PJL29	Amp/ P/ B/ S	1	Amp/ P	Lost
*V. communis*	NS12	Amp/ P/ B/ TE	1	P/ B	Lost
*V. communis*	NS14	Amp/ P/ B/ TE	1	No resistance	Lost
*V. communis*	NL30	Amp/ P/ B/ TE/ VA	1	No resistance	Lost
*V. communis*	NS28	Amp/ P/ B/ TE/ VA	1	Amp/ P/ B	Lost
*V. communis*	TL3	Amp/ P/ B/ VA	1	Amp	Lost
*V. communis*	PKGL13	Amp/ P/ B/ VA	1	Amp/ P/ B	Lost
*V. communis*	PKL3; PKK3	Amp/ P/ E/ S	1	Amp	Lost
*V. communis*	LK21, LL15	Amp/ P/ E/ S/ VA	1	Amp	Lost
*V. communis*	LL5, LS17, LS25	Amp/ P/ E/ VA	1	Amp	Lost
*V. communis*	LL3, LL8, LS19, LL28	Amp/ P/ E/ VA	1	Amp/ P	Lost
*V. communis*	PKS2	Amp/ P/ S	1	Amp	Lost
*V. communis*	BS2	Amp/ P/ VA	1	Amp/ VA	Lost
*V. communis*	PKS4, BL17	Amp/ P/ VA	1	Amp	Lost
*V. communis*	BS18	Amp/ P/ VA	1	Amp/ P	Lost
*V. communis*	PKL2, PKK6, PKS19, BS14	Amp/ P/ VA	1	No resistance	Lost
*V. diabolicus*	PKK8	Amp/ P	1	No resistance	Lost
*V. furnissii*	NL2	Amp/ P/ TE/ VA	1	No resistance	Lost
*V. harveyi*	PJK21, PJS28	Amp/ P/ B/ S	1	Amp/ P	Lost
*V. mytili*	LS23	Amp/ P/ E/ VA	1	Amp	Lost
*V. parahaemolyticus*	PKL11	Amp/ P	1	No resistance	Lost
*V. parahaemolyticus*	PKGS23	Amp/ P/ VA	1	VA	Lost
*V. parahaemolyticus*	PKGS11	Amp/ P/ B/ E	1	Amp/ P/ B/ E	Lost
*V. parahaemolyticus*	LL23	Amp/ P/ B/ E/ VA	1	Amp	Lost
*V. parahaemolyticus*	LL30	Amp/ P/ B/ E/ VA	1	Amp/ P	Lost
*V. parahaemolyticus*	PKGS6	Amp/ P/ B/ E/ VA	1	Amp/ P/ B/ E	Lost
*V. parahaemolyticus*	PKGL19	Amp/ P/ B/ E/ VA	1	Amp/ B/ VA	Lost
*V. parahaemolyticus*	PKGK24	Amp/ P/ B/ E/ VA	1	No resistance	Lost
*V. parahaemolyticus*	NK6	Amp/ P/ B/ TE	1	Amp/ P/ B	Lost
*V. parahaemolyticus*	NS27	Amp/ P/ B/ TE/ VA	1	P/ B/ VA	Lost
*V. parahaemolyticus*	PKGL17	Amp/ P/ B/ VA	1	Amp/ P/ B/ VA	Lost
*V. parahaemolyticus*	PKGL22	Amp/ P/ B/ VA	1	Amp/ B/ VA	Lost
*V. parahaemolyticus*	BL4, BS11, BK12, BL21	Amp/ P/ B/ VA	1	Amp/ P	Lost
*V. parahaemolyticus*	PKS27, BS28, BK28	Amp/ P/ B/ VA	1	Amp	Lost
*V. parahaemolyticus*	PKK14	Amp/ P/ B/ VA	1	No resistance	Lost
*V. parahaemolyticus*	PKGK19	Amp/ P/ E	1	Amp/ P	Lost
*V. parahaemolyticus*	NK29	Amp/ P/ TE	1	Amp/ P	Lost
*V. parahaemolyticus*	PKK7, BS8	Amp/ P/ VA	1	Amp	Lost
*V. parahaemolyticus*	PKL14	Amp/ P/ VA	1	No resistance	Lost
*V. parahaemolyticus*	PKL17	Amp/ P/ VA	1	Amp/ P	Lost
*V. parahaemolyticus*	PKK24	Amp/ P/ VA	1	P	Lost
*V. parahaemolyticus*	PKGK17	E/ VA	1	E	Lost
*V. parahaemolyticus*	PKGL4	P/ B/ VA	1	P/ B/ VA	Lost
*V. parahaemolyticus*	PKGL5, PKL1	P/ B/ VA	1	No resistance	Lost
*V. parahaemolyticus*	PKK23	P/ S	1	No resistance	Lost
*V. rotiferianus*	LS7	Amp/ P/ B/ E/ VA	1	Amp/ P	Lost
*V. rotiferianus*	LS15	Amp/ P/ E/ VA	1	Amp/ P	Lost
*V. rotiferianus*	LK19	Amp/ P/ B/ E/ VA	1	Amp	Lost
*V. rotiferianus*	PKGK9	B/ E/ VA	1	No resistance	Lost
*V. rotiferianus*	JS28	B/ E/ TE/ VA	1	TE/ VA	Lost
*V. tubiashii*	PJK19	Amp/ P/ B/ S	1	Amp/ P/ B	Lost
*V. vulnificus*	LK5, LS28	Amp/ P/ E/ VA	1	Amp	Lost
*V. vulnificus*	LL21	Amp/ P/ E/ S/ VA	1	Amp	Lost
*V. vulnificus*	PJK12	Amp/ P/ B	1	Amp/ P/ B	Lost
*V. vulnificus*	LK18, TS22	Amp/ P/ B/ E/ S/ VA	1	Amp/ P	Lost
*V. vulnificus*	TL5, TS6	Amp/ P/ B/ E/ S/ VA	1	No resistance	Lost
*V. vulnificus*	PJK8	Amp/ P/ B/ S	1	Amp/ P/ B	Lost
*V. vulnificus*	PJS16	Amp/ P/ B/ S	1	Amp/ P	Lost
*V. vulnificus*	NL15	Amp/ P/ B/ TE/ VA	1	Amp/ P/ B	Lost
*V. vulnificus*	TS19	Amp/ P/ B/ VA	1	Amp	Lost
*V. vulnificus*	TK2	Amp/ P/ E/ VA	1	Amp	Lost
*V. vulnificus*	PKGK3	Amp/ P/ VA	1	Amp/ P/ VA	Lost
*V. vulnificus*	BL8	Amp/ P/ VA	1	Amp	Lost
*V. vulnificus*	JS9	B/ E/ S/ TE/ VA	1	B/ E	Lost
*V. vulnificus*	JS19	E/ S/ TE/ VA	1	E/ VA	Lost
*V. vulnificus*	PJS19	P/ B	1	P	Lost
*V. vulnificus*	BK3	P/ S	1	No resistance	Lost
*Vibrio* spp.	LL18	Amp/ P/ B/ E/ VA	1	Amp/ P	Lost
*Vibrio* spp.	PKL21	Amp/ P/ B/ S/ VA	1	No resistance	Lost

*AMP* ampicillin, *P* penicillin G, *B* bacitracin, *E* erythromycin, *S* streptomycin, *TE* tetracycline, *VA* vancomycin

Following plasmid curing test, all isolates lost their plasmid DNA with 139 (95%) isolates showed altered resistance phenotype towards antibiotics. However, the isolates were remained resistant to either one or all antibiotics after plasmid curing, whereby 72% isolates remained resistance to ampicillin, 46% to penicillin G, 16% to bacitracin, 8% to vancomycin, 4% to erythromycin, 2%to tetracycline and 1% to streptomycin.

### Multiplex antibiotic resistance (MAR) index

Overall, the mean MAR index value for *Vibrio* isolates was 0.44, with 85% showed MAR index value of more than 0.2. The most frequent MAR index for *Vibrio* spp. was 0.57, indicating that the isolates were resistance to four different antibiotics. In addition, the high MAR index value was observed among *V. fluvialis* (0.71), *V. tubiashii* (0.61), *V. communis* (0.57) and *V. mytili* (0.54). The other *Vibrio* spp. had MAR index value between 0.28 and 0.47, such as *V. furnissii* (0.47), *V. vulnificus* (0.45), *Vibrio* sp. (0.42), *V. harveyi* (0.4), *V. parahaemolyticus* (0.4), *V. rotiferianus* (0.3), *V campbellii* (0.3), *V. diabolicus* (0.29) and *V. alginolyticus* (0.28).

## Discussion

Grouper (*Epinephelus* spp.) has great commercial value worldwide including Malaysia due to high market price. Previous study reported that the production of grouper has increased, particularly in China, Indonesia, Philippines, Mexico and Pakistan [4]. However, high stocking density and poor handling of fish trigger disease outbreaks and mortality. In fact, two third of diseases reported in grouper was due to infection by *Vibrio* [12].

This study was successfully isolated 380 *Vibrio* bacteria from liver, spleen and/or kidney of 195 (72%) groupers. The liver, spleen and kidney were known as vital organs for *Vibrio* infection [29]. In fact, Li et al. [38] had shown significant increased of *Vibrio* in spleen and kidney paralleled with the decline in macrophage phagocytosis of the infected fish. In addition, histology observation showed *Vibrio* was multiplied extensively in the kidney, liver and spleen of the infected fish [17].

The phylogenetic analysis of *pyrH* sequences revealed that 97% of the strains were clustered into 12 distinct species, with 3% strains were clustered into *Vibrio* sp. Among these 12 *Vibrio* species, *V. communis, V. parahaemolyticus, V. alginolyticus* and *V. vulnificus* were highly isolated from groupers. It seemed that the *pyrH* gene could effectively distinguished the species level of *Vibrio* including *V. communis,* which currently being described as *Vibrio* spp. [11]. Thus, the *pyrH* gene is a good phylo marker of *Vibrio* and a good discriminatory target at species level [48, 55, 59]. In addition, these findings are in agreement with previous studies that reported high presence of *V. alginolyticus, V. vulnificus* and *V. parahaemolyticus* within cultured tiger grouper (*Epinephelus fuscoguttatus*) in deep sea cage and other aquatic animals in Malaysian costal area [1, 19].

The antibiotic susceptibility test found that they were resistant to ampicillin, penicillin G and vancomycin, highly susceptible to tetracycline and streptomycin and intermediate against bacitracin and erythromycin. In fact, 64% of the *Vibrio* isolates were resistance to at least three or more antibiotics. These findings were similar with a previous study reported that 68% of the *Vibrio* isolates were resistance to at least three or more antibiotics [65].

Based on MAR index, the isolates have been continuously exposed to antibiotics since the mean value calculated among 380 isolates was 0.44 [23]. There were also 85% isolates having MAR index value of more than 0.2, which indicate high risk of contamination with potentially hazardous to human health [62]. This is in agreement with previous studies done in Malaysian aquaculture [44, 52, 68].

The antibiotic susceptibility profiles obtained in current study clearly indicate that tetracycline and streptomycin remained highly effective against *Vibrio* spp., including *V. communis, V. parahaemolyticus, V. alginolyticus, V. vulnificus* and *V. rotiferianus.* This was supported by previous studies on the effectiveness of both antibiotics for the treatment against *Vibrio* spp. in Malaysia [24, 44]. In addition, many studies have proven that *V. parahaemolyticus* isolated from fish and other aquatic animals was susceptible to tetracycline and streptomycin [24, 32, 35, 44, 46, 47, 61, 66].

This study also found that 70 and 56% of *Vibrio* isolates were intermediate and susceptible against erythromycin and bacitracin, respectively, while 30% of *Vibrio* isolates mainly *V. rotiferianus* and *V. mytili* were highly resistance against erythromycin. According to Kumar et al. [32], *Vibrio* spp. isolated from seafood samples from coastal India were resistant to ampicillin, penicillin and erythromycin, while 44% of the *Vibrio* isolates were found resistance to bacitracin. This is slightly less compared to 98% in a study by Sahilah et al. [51] who studied the resistance to bacitracin among *V. parahaemolyticus* in cockle. The discrepancies regarding the resistance of *Vibrio* to antibiotic could possibly be due to geographical variation or difference in test methodology [36].

In this study, *Vibrio* spp. showed high resistance toward ampicillin and penicillin G. Previous reports showed resistance of both antibiotics in *Vibrio* are not a new phenomenon. Zanetti et al. [67] reported that *V. parahaemolyticus, V. vulnificus* and *V. alginolyticus* isolated from seawater were highly resistance to ampicillin. Another study reported that 81% of *V. parahaemolyticus* isolated from oyster were resistance to ampicillin [24]. Similarly, *V. parahaemolyticus* isolated from croaker fish (*P. senegalensis*) and blue crab (*Callinectes sapidus*) at Lagos Lagoon, Nigeria, showed resistance to ampicillin [46]. In China, 79.6% of *V. parahaemolyticus* isolated from fish, shrimp and oyster were resistant to ampicillin [65].

In addition, Vaseeharan et al. [64] reported the emergence of resistant *Vibrio* strains against ampicillin and penicillin in India. Over 80% of *V. harveyi* from fish in Italy showed resistant to ampicillin, amoxicillin and erythromycin [56]. The findings were also in agreement with studies done all around world and Malaysia [2, 5, 18, 52, 61]. Emergence of high resistance *Vibrio* strains against ampicillin and penicillin was related with the extensive used of both antibiotics and could influence the disease management in aquaculture system [21]. Thus, both ampicillin and penicillin are ineffective for treatment of *Vibrio* infection [61]. Instead of ampicillin and penicillin, seven out of 13 *Vibrio* spp. were found to be highly resistant against vancomycin. This finding was consistent with a previous study in Selangor, Malaysia, that reported by Noorlis et al. [44]. In addition, a study done in South Korea showed that all *V. parahaemolyticus* isolated from oysters were resistance to ampicillin and vancomycin [30].

The plasmid profiling revealed low occurrence of plasmid (39%), indicating that the resistance genes were of chromosomal mediated. Manjusha and Sarita [41] also revealed that 21 (70%) out of 30 *Vibrio* isolates did not exhibit plasmids but still resistance to all antibiotics. In addition, previous studies on *Vibrio* found no correlation between resistance to the antibiotics and the presence of plasmid [16, 67]. On the other hand, this study found that 67% isolates with plasmid were resistance to more than four antibiotics, indicating that the presence of plasmids might enhanced the virulence and antibiotic resistance [16, 49].

This study also revealed that the resistance to all antibiotics especially to ampicillin, penicillin G, bacitracin and vancomycin was related to the chromosome since the isolates remained resistant to these antibiotics after plasmid curing. Similar results were demonstrated in other studies by Reboucas et al. [50] and Costa et al. [14]. A study reported that the β-lactamase involved in ampicillin resistance was found to be chromosomally encoded in *V. harveyi* [28]. Thus, the antibiotic resistance genes in *Vibrio* spp. isolated from grouper were found in both plasmid and chromosome.

## Conclusion

In conclusion, our findings represent a comprehensive report on the antibiotic resistance profiles and plasmid curing of *Vibrio* spp. isolated from groupers in Malaysia. The vancomycin, bacitracin and erythromycin resistance patterns suggested that treatment of vibriosis with these antibiotics need to be reconsidered. By reducing the usage of these antibiotics may consequence the decrease in antibiotic resistance. Hence, continuous monitoring of susceptibility of *Vibrio* strains to antibiotics is necessary to ensure the best treatment and combat drug resistance among them.

## Methods

### Sampling of groupers

A total of 210 hybrid grouper (*Epinephelus fuscoguttatus* (♀) × *E. lanceolatus* (♂) and 60 green groupers (*E. suillus*) were obtained from nine farms that were located in different geographical regions of Peninsular Malaysia (Table 6). Fish were obtained during the period between December 2016 and September 2017. Thirty fish were randomly collected from each farm and the size of fish varies ranging between 14 and 580 g in weight, and between 10 and 31 cm in length. Any clinical signs and gross lesions of vibriosis were observed and documented.

**Table 6:** List of farms

	Farm	Location	GPS Location
1	Widad Agrofarm Sdn. Bhd.	Pulau Langkawi, Kedah	6^o^14’38.04″N, 99^o^57’11.88″E
2	Weng Teik Shrimp Farm	Bukit Mertajam, Penang	5^o^20’33.972″N, 100^o^26’36.96″E
3	Ain Aquaculture Sdn. Bhd.	Kota Bharu, Kelantan	6^o^7’59.808″N, 102^o^14’18.96″E
4	Perniagaan Johari	Besut, Terengganu	5^o^34’15.852″N, 102^o^31’8.795″E
5	Aqua Hub Sdn. Bhd.	Kuala Gula, Perak	4^o^59’51.203″N, 100^o^24’18.032″E
6	KS Aquaculture Sdn. Bhd.	Pulau Ketam, Selangor	3^o^2’9.348″N, 101^o^14’34.799″E
7	Oasis Long Diann Bio-Tech Sdn. Bhd.	Banting, Selangor	2^o^49’12.216″N, 101^o^30’56.404″E
8	Aqua Genesis Sdn. Bhd.	Port Dickson, Negeri Sembilan	2^o^32’13.848″N, 101^o^48’21.6″E
9	Smart Objectives Sdn. Bhd.	Kukup Laut, Johor	1^o^25’22.8″N, 103^o^26’32.999″E

Euthanasia and dissection of fish was performed at the sampling sites. Fish was euthanized in 0.2% of tricaine methanesulfonate (Western Chemical Industries, Mumbai, India). Fish was dissected for the collection of liver, kidney and spleen. These organs were immersed separately in 1× phosphate buffered saline (PBS) (Merck, New Jersey, USA). The samples were kept in ice and transported to the laboratory for processing on the same day.

### Isolation of *Vibrio* from liver, spleen and kidney

The liver, spleen and kidney of the groupers were separately homogenized using stomacher for 1 min. The homogenized sample was then streaked on thio-sulphate citrate bile salt sucrose (TCBS) agar (Difco, Michigan, USA) and incubated at 30 °C for 16 h. A single colony of bacteria suspected of *Vibrio* was incubated in tryptic soy broth (TSB) (Difco) with 1.5% normal saline (Merck) and incubated at 30 °C for 16 h. Alternate steps between TCBS and TSB containing 1.5% normal saline were performed until a pure colony of *Vibrio* was obtained. A pure isolate was inoculated into semi-solid nutrient agar and TSB with 20% glycerol, incubated at 30 °C for 16 h and then stored until further analysis.

### Identification of *Vibrio* spp. using gram stain, biochemical tests, *pyrH*-PCR assay and sequencing

All pure colonies were subjected to Gram staining (Becton Dickinson, New Jersey, USA) and biochemical tests (triple sugar iron (TSI), oxidase, catalase, O-nitrophenyl-beta-D-galactosifase (ONPG) and lysine decarboxylase (LDC) (Oxoid, Hampshire, UK) for identification of the *Vibrio* spp. [7, 27].

Genomic DNA of pure colonies were extracted using the GeneJET Genomic DNA Purification Kit (Thermo Fisher Scientific, Massachusetts, USA) according to the manufacturer’s protocol. The genomic DNA was subjected to PCR amplification using *pyrH* primers; *pyrH*_F (5′-GAT CGT ATG GCT CAA GAA G-3′) and *pyrH*_R (5′-TAG GCA TTT TGT GGT CAC G-3′) [10]. The PCR reactions were performed in a final volume of 50 μL containing 1× PCR buffer, 3 mM MgCl_2_, 200 uM dNTPs, 0.5 pmol of each primer, 2.5 U *Taq* polymerase and 50 ng of template DNA (Promega, Wisconsin, USA). The *pyrH* cycle condition was an initial denaturation at 95 °C for 5 s, followed by 33 cycles of 95 °C for 1 min; 59 °C for 2 min 15 s and 72 °C for 1 min 15 s, and a final extension of 72 °C for 10 s. The amplification was performed in an Eppendorf Mastercycler Nexus Thermal Cycler (Eppendorf, Hamburg, Germany).

Direct sequencing of purified PCR products was performed on sense strands (First Base, Kuala Lumpur, Malaysia). Phylogenetic analysis was conducted using MEGA version 7.0 [33]. The phylogenetic construction of *pyrH* genes of *Vibrio* isolates and reference sequences (obtained from GenBank database) was inferred using the Maximum Likelihood (ML) method based on the General Time Reversible (GTR) model and 1000 rapid bootstrap inferences [43].

### Antibiotic susceptibility test

The *Vibrio* isolates were assessed for their antibiotic susceptibility by disc diffusion method as described by Devi et al. [16]. Seven antibiotics (Thermo Fisher Scientific, Massachusetts, USA) were used, which included tetracycline 30 μg (TE), ampicillin 10 μg (AMP), penicillin G 10 μg (P), streptomycin 10 μg (S), erythromycin 15 μg (E), vancomycin 30 μg (VA) and bacitracin 10 μg (B).

*Vibrio* suspension of approximately 1 × 10^8^ CFU/mL was inoculated by lawn on Muller-Hinton agar (MHA) (Difco) using a cotton swab. The antibiotic discs were then placed 15 mm away from the edge of the plates to prevent overlapping of the zones of inhibition. After incubation at 37 °C for 24 h, the diameter of inhibition zone was measured. Strain was then regarded as resistance, intermediate or susceptible based on guidelines of the Clinical and Laboratory Standards Institute (CLSI) [13].

### Plasmid profiling

A total of 2.5 mL of bacterial culture from TSB supplemented with 1.5% normal saline was centrifuged at 12000×g for 3 min. A *Vibrio* isolate was purified using GeneJet Plasmid Purification kit according to the manufacturer’s protocol (Thermo Fisher Scientific)). The supernatant containing plasmid was kept at -20 °C until used. Presence of plasmid was detected using the agarose gel (1% w/v) electrophoresis (Bio-Rad Laboratories, California, USA).

### Plasmid curing

*Vibrio* isolates that harboured plasmid were treated with the acridine orange (AO) (Thermo Fisher Scientific) following modifications of the methods by Letchumanan et al. [37]. A single colony of bacterial isolate from TCBS agar was grown on Tryptone Soy Broth (TSB) supplemented with 1.5% NaCl and 0.2 mg/mL AO. Bacterial culture was incubated at 37 °C for 24 h under constant agitation. After treatment with curing agent, the agarose gel (1% w/v) electrophoresis was performed to detect for the presence of plasmid. In addition, to verify changes in resistance profiles, the antibiotic susceptibility test was again performed as described previously.

### Multiplex antibiotic resistance index

Multiplex antibiotic resistance (MAR) index was calculated based on the ratio of resistance antibiotics to the total number of antibiotics to which the isolates are exposed to [31]. The MAR index provides an accurate estimation about the origin of contamination [18].

## Supplementary information

**Additional file 1.** The sequences of oligonucleotide used in this study.

## Data Availability

The datasets used and/or analysed during the current study are available from the corresponding author on reasonable request.

## Abbreviations

A/A: Acidic slant/Acidic butt
Amp: Ampicillin
AO: Acridine orange
B: Bacitracin
E: Erythromycin
EtBr: Ethidium bromide
GTR: General Time Reversible
K/A: Alkaline slant/Acidic butt
LDC: Lysine decarboxylase
MAR: Multiple antibiotic resistance
MHA: Muller-Hinton agar
ML: Maximum Likelihood
ONPG: O-nitrophenyl-beta-D-galactosidase
P: Penicillin G
PBS: Phosphate buffered saline
S: Streptomycin
SDS: Sodium dodecyl sulphate
Spp: Species
TCBS: Thio-sulphate citrate bile salt sucrose
TE: Tetracycline
TSB: Tryptone soy broth
TSI: Triple sugar iron
VA: Vancomycin
