# Supplementary material for: Prevalence, antimicrobial susceptibility and plasmid profiling of Vibrio spp. isolated from cultured groupers in Peninsular Malaysia
Source: BMC Microbiol. 2019 Nov 11;19:251. doi: 10.1186/s12866-019-1624-2 (PMC6849203; doi:10.1186/s12866-019-1624-2)
Supplement: Supplementary file 1 — Additional file 1. The sequences of oligonucleotide used in this study. [file 12866_2019_1624_MOESM1_ESM.docx]

**Additional file 1**

The sequences of oligonucleotide used in this study

| **Name** | **Organism** | **Sequence (5’ to 3’)** | **GenBank Accession Number** |
| --- | --- | --- | --- |
| LL06 | *Vibrio alginolyticus* | GGTGGCGGTAACTTGTTCCGTGGTGCAGGTCTTGCTGAAGCTGGTATGAACCGCGTAGTAGGTGACCACATGGGAATGCTTGCAACAGTAATGAATGGCCTTGCGATGCGTGATGCTCTTCACCGTGCTTACGTAAATGCTCGCGTTATGTCTGCAATCCCTCTTAAAGGTGTATGTGACGACTACAATTGGGCTGATGCTATCCGTGAATTGCGCCAGGGACGCGTAGTAATCTTCAGCGCTGGTACGGGTAACCCTTTCTTTACAACGGATTCTGCTGCTTGCTTACGCGGTATCGAAATTGAAGCGGACGTGGTCTTAAAAGCGACAAAGGTGGATGGGGTATTTACGGCTGACCCTGTAGCAAACCCAGACGCAGAACTTTATGACAAACTATCCTATGCAGAGGTCCTGGATAAAGAACTTAAAGTAATGGACTTA | MN253135 |
| PKGL01 | *Vibrio alginolyticus* | GGTGGCGGTAACTTGTTCCGTGGTGCAGGTCTAGCTGAAGCTGGTATGAACCGCGTAGTGGGTGACCACATGGGTATGCTTGCTACAGTAATGAATGGCCTAGCGATGCGTGATGCTCTTCACCGTGCTTACGTAAATGCGCGCGTTATGTCTGCAATCCCTCTAAAAGGCGTATGTGACGACTATAATTGGGCTGATGCTATCCGCGAATTGAGGCAGGGCCGAGTTGTCATCTTCTCTGCTGGTACTGGTAACCCTTTCTTTACAACGGATTCTGCTGCTTGCTTAAGAGGTATCGAAATAGAAGCTGATGTCGTACTAAAAGCGACAAAGGTGGATGGGGTATTTACTGCTGACCCTGTAGCAAACCCAGACGCAGAATTATATGATAAGTTATCTTATGCAGAAGTCCTGGATAAAGAACTTAAAGTAATGGACTTA | MN253136 |
| PKGS01 | *Vibrio alginolyticus* | GGGGGGGGGAACCTTTTTCGCGGTGCAGGTCTAGCTGAAGCTGGTATGAACCGCGTAGTGGGTGACCACATGGGTATGCTTGCAACAGTAATGAATGGCCTAGCGATGCGTGATGCTCTTCACCGTGCTTACGTAAATGCTCGCGTTATGTCTGCAATCCCTCTAAAAGGTGTATGTGACGACTACAATTGGGCTGATGCTATCCGTGAACTTAGACAAGGCAGAGTTGTCATCTTCTCTGCTGGTACGGGTAACCCTTTCTTTACAACGGATTCTGCTGCTTGCTTAAGGGGTATCGAAATAGAAGCTGACGTGGTGCTTAAAGCGACAAAGGTGGATGGGGTATTTACGGCTGACCCAGTAGCCAATCCCGACGCCGAACTTTATGATAAGCTGAGTTATGCCGAGGTCCTGGATAAAGAACTTAAAGTAATGGACTTA | MN253137 |
| PKGL02 | *Vibrio alginolyticus* | GGTGGCGGTAACTTGTTCCGTGGTGCAGGTCTAGCTGAAGCTGGTATGAACCGCGTAGTGGGTGACCACATGGGTATGCTTGCTACAGTAATGAATGGCCTAGCGATGCGTGATGCTCTTCACCGTGCTTACGTAAATGCTCGCGTTATGTCTGCAATTCCTCTTAAAGGCGTATGTGACGACTACAATTGGGCTGATGCTATCCGCGAACTTCGCCAAGGCCGAGTTGTCATCTTCTCTGCTGGTACTGGTAACCCTTTCTTTACAACGGATTCTGCTGCTTGTTTACGTGGTATCGAAATTGAAGCGGATGTCGTACTAAAAGCGACAAAGGTGGACGGGGTATTTACGGCTGACCCTGTAGCAAACCCAGACGCAGAACTGTATGACAAATTAAGCTATGCCGAGGTCCTGGATAAAGAACTTAAAGTAATGGACTTA | MN253138 |
| PKGK02 | *Vibrio alginolyticus* | GGTGGCGGTAACTTGTTCCGTGGTGCAGGTCTAGCTGAAGCTGGTATGAACCGCGTAGTTGGTGACCACATGGGTATGCTTGCTACAGTAATGAATGGCCTAGCGATGCGTGATGCTCTTCACCGTGCATACGTAAATGCTCGCGTTATGTCTGCAATCCCTCTAAAAGGCGTATGTGACGACTATAATTGGGCTGATGCGATCCGTGAACTTAGACAAGGCAGAGTTGTCATCTTCTCTGCTGGTACGGGTAACCCTTTCTTTACAACGGATTCTGCTGCTTGTTTAAGAGGTATCGAAATAGAAGCGGATGTCGTACTAAAAGCGACAAAGGTGGATGGGGTATTTACGGCAGACCCTGTAGCAAACCCAGACGCAGAGCTGTACGACAAATTAAGCTATGCCGAGGTCCTGGATAAGGAACTTAAAGTAATGGACTTA | MN253139 |
| PKGL03 | *Vibrio alginolyticus* | GGTGGCGGTAACTTGTTCCGTGGTGCAGGTCTAGCTGAAGCTGGTATGAACCGCGTAGTGGGTGACCACATGGGTATGCTTGCTACAGTAATGAATGGCCTAGCGATGCGTGATGCTCTTCACCGTGCTTACGTAAATGCTCGCGTTATGTCTGCAATCCCTCTAAAAGGCGTATGTGACGACTACAATTGGGCTGATGCTATCCGCGAACTTCGCCAAGGCCGAGTTGTCATCTTCTCTGCTGGTACTGGTAACCCTTTCTTTACAACGGATTCTGCTGCTTGTTTACGTGGTATCGAAATTGAAGCGGATGTCGTACTAAAAGCGACAAAGGTGGATGGGGTATTTACTGCTGACCCTGTAGCAAACCCAGACGCAGAACTGTATGACAAATTAAGCTATGCCGAGGTCCTGGATAAAGAACTTAAAGTAATGGACTTA | MN253140 |
| PKGL06 | *Vibrio alginolyticus* | GGTGGCGGTAACTTGTTCCGTGGTGCAGGTCTAGCTGAAGCTGGTATGAACCGCGTAGTGGGTGACCACATGGGTATGCTTGCTACAGTAATGAATGGCCTAGCGATGCGTGATGCTCTTCACCGTGCTTACGTAAATGCTCGCGTGATGTCTGCAATTCCTCTAAAAGGCGTATGTGACGACTACAATTGGGCTGATGCTATCCGCGAACTTCGCCAAGGCCGAGTTGTCATCTTCTCTGCTGGTACTGGTAACCCTTTCTTTACAACGGATAGCGCTGCTTGTTTACGTGGTATCGAAATTGAAGCGGATGTCGTACTAAAAGCGACAAAGGTGGATGGGGTATTTACGGCTGACCCTGTAGCAAACCCAGACGCAGAACTGTATGACAAACTATCCTATGCCGAGGTCCTGGACAAGGAACTTAAAGTAATGGACTTA | MN253141 |
| PKGL09 | *Vibrio alginolyticus* | GGTGGCGGTAACTTGTTCCGTGGTGCAGGTCTAGCTGAAGCTGGTATGAACCGCGTAGTGGGTGACCACATGGGTATGCTTGCTACAGTAATGAATGGCCTAGCGATGCGTGATGCTCTTCACCGTGCTTACGTAAATGCTCGCGTGATGTCTGCAATTCCTCTAAAAGGCGTATGTGACGACTACAATTGGGCTGATGCTATCCGCGAACTTCGCCAAGGCCGAGTTGTCATCTTCTCTGCTGGTACTGGTAACCCTTTCTTTACAACGGATTCTGCTGCTTGTTTACGTGGTATCGAAATTGAAGCGGATGTCGTACTTAAAGCGACAAAGGTCGATGGGGTATTTACGGCTGACCCTGTAGCAAACCCAGACGCAGAACTGTATGACAAATTAAGCTATGCCGAGGTCCTGGATAAAGAACTTAAAGTAATGGACTTA | MN253142 |
| PKGS09 | *Vibrio alginolyticus* | GGTGGCGGTAACTTGTTCCGTGGTGCAGGTCTAGCTGAAGCTGGTATGAACCGCGTAGTGGGTGACCACATGGGTATGCTTGCTACAGTAATGAATGGCCTAGCGATGCGTGATGCTCTTCACCGTGCTTACGTAAATGCTCGCGTAATGTCTGCAATTCCTCTAAAAGGCGTATGTGACGACTACAATTGGGCTGATGCTATCCGCGAACTTCGCCAAGGCCGAGTTGTCATCTTCTCTGCTGGTACTGGTAACCCTTTCTTTACAACGGATTCTGCTGCTTGTTTACGTGGTATCGAAATTGAAGCGGATGTCGTACTAAAAGCGACAAAGGTGGACGGGGTATTTACGGCTGACCCTGTAGCAAACCCAGACGCAGAACTGTATGACAAATTAAGCTATGCCGAGGTCCTGGATAAAGAACTTAAAGTAATGGACTTA | MN253143 |
| PKGL11 | *Vibrio alginolyticus* | GGTGGCGGTAACTTGTTCCGTGGTGCAGGTCTAGCTGAAGCTGGTATGAACCGCGTAGTGGGTGACCACATGGGTATGCTTGCTACAGTAATGAATGGCCTAGCGATGCGTGATGCTCTTCACCGTGCTTACGTAAATGCTCGCGTTATGTCTGCAATTCCTCTTAAAGGCGTATGTGACGACTACAATTGGGCTGATGCTATCCGCGAACTTCGCCAAGGCCGAGTTGTCATCTTCTCTGCTGGTACTGGTAACCCTTTCTTTACAACGGATTCTGCTGCTTGTTTACGTGGTATCGAAATTGAAGCCGATGTCGTACTAAAAGCGACAAAGGTGGACGGGGTATTTACGGCTGACCCTGTAGCAAACCCAGACGCAGAACTGTATGACAAATTAAGCTATGCCGAGGTCCTGGATAAAGAACTTAAAGTAATGGACTTA | MN253144 |
| PKGK11 | *Vibrio alginolyticus* | GGTGGCGGTAACTTGTTCCGTGGTGCAGGTCTTGCTGAAGCTGGTATGAACCGCGTTGTAGGTGACCACATGGGTATGCTTGCTACAGTTATGAATGGCCTAGCGATGCGTGATGCACTCCATAGAGCATACGTAAATGCTCGCGTAATGTCTGCAATTCCTCTAAAAGGCGTATGTGACGACTACAATTGGGCTGATGCTATCCGCGAACTTCGCCAAGGCCGAGTTGTCATCTTCTCTGCTGGTACTGGTAACCCTTTCTTTACAACGGATTCTGCTGCTTGTTTACGTGGTATCGAAATTGAAGCGGATGTCGTACTAAAAGCGACAAAGGTGGATGGGGTATTCACTGCTGACCCCGTAGCAAACCCCGACGCAGAACTGTATGACAAATTAAGCTATGCCGAGGTCCTGGATAAAGAACTTAAAGTAATGGACTTA | MN253145 |
| PKGL12 | *Vibrio alginolyticus* | GGTGGCGGTAACTTGTTCCGTGGTGCAGGTCTAGCTGAAGCTGGTATGAACCGCGTAGTAGGTGACCACATGGGTATGCTTGCTACAGTAATGAATGGCCTAGCGATGCGTGATGCTCTTCACCGTGCATACGTAAATGCTCGCGTTATGTCTGCAATTCCTCTAAAAGGCGTATGTGACGACTACAATTGGGCTGATGCTATCCGTGAGCTTCGCCAAGGCCGAGTTGTCATCTTCTCTGCTGGTACTGGTAACCCGTTTTTTACTACGGATTCTGCTGCTTGTTTACGTGGTATCGAAATTGAAGCGGATGTCGTACTAAAAGCGACAAAAGTTGATGGGGTATTTACTGCTGACCCTGTAGCAAACCCAGACGCAGAGCTGTATGATAAGCTTTCTTATGCAGAAGTTCTGGATAAAGAGCTGAAAGTAATGGACTTA | MN253146 |
| PKGS12 | *Vibrio alginolyticus* | GGTGGCGGTAACTTGTTCCGTGGTGCAGGTCTTGCTGAAGCTGGTATGAACCGCGTAGTTGGTGACCACATGGGTATGCTTGCTACAGTAATGAATGGCCTAGCGATGCGTGATGCTCTTCACCGTGCTTACGTAAATGCTCGCGTTATGTCTGCAATCCCTCTAAAAGGTGTATGTGACGACTACAATTGGGCTGATGCTATCCGCGAACTTCGCCAAGGCCGAGTTGTCATCTTCTCTGCTGGTACTGGTAACCCGTTCTTTACAACGGATTCTGCTGCTTGTTTACGTGGTATCGAAATTGAAGCGGATGTCGTACTAAAAGCGACAAAGGTTGATGGGGTATTTACTGCTGACCCTGTAGCAAACCCAGATGCAGAACTGTATGATAAGCTTTCTTATGCGGAAGTTCTGGATAAAGAGCTGAAAGTAATGGACTTA | MN253147 |
| PKGK12 | *Vibrio alginolyticus* | GGTGGGGGGAATCTTTTTCGGGGTGCAGGTCTTGCTGAAGCTGGTATGAACCGCGTAGTAGGTGACCACATGGGTATGCTTGCAACAGTAATGAATGGCCTAGCGATGCGTGATGCTCTTCACCGTGCTTACGTAAATGCTCGCGTGATGTCTGCAATTCCTCTAAAAGGTGTATGTGACGACTACAATTGGGCTGATGCTATCCGCGAACTTAGGCAAGGCCGTGTTGTCATCTTCTCTGCTGGTACGGGTAACCCTTTCTTTACCACAGATTCTGCTGCTTGCTTACGTGGTATCGAAATTGAAGCCGACGTCGTTCTTAAAGCGACAAAGGTAGATGGGGTATTTACGGCTGACCCTGTAGCAAACCCAGATGCAGAACTGTATGATAAGCTTTCTTATGCGGAAGTTCTGGATAAAGAGCTGAAAGTAATGGACTTA | MN253148 |
| PKGS13 | *Vibrio alginolyticus* | GGCGGTGGCAACTTGTTCCGTGGTGCAGGTCTTGCTGAAGCTGGTATGAACCGCGTAGTAGGTGACCACATGGGTATGCTTGCTACAGTAATGAATGGCCTAGCGATGCGTGATGCTCTTCACCGTGCTTACGTAAATGCTCGCGTAATGTCTGCAATTCCTCTAAAAGGCGTATGTGACGACTACAATTGGGCTGATGCTATCCGCGAACTTAGGCAAGGCAGAGTTGTCATCTTCTCTGCTGGTACGGGAAACCCTTTTTTTACTACAGATTCTGCTGCTTGCTTACGTGGTATCGAAATTGAAGCTGACGTAGTACTAAAAGCGACAAAGGTGGACGGGGTATTTACGGCTGACCCTGTAGCAAACCCAGACGCAGAACTGTATGACAAATTAAGCTATGCCGAGGTCCTGGATAAAGAACTTAAAGTAATGGACTTA | MN253149 |
| PKGL15 | *Vibrio alginolyticus* | GGTGGCGGTAACTTGTTCCGTGGTGCAGGTCTAGCTGAAGCTGGTATGAACCGCGTAGTAGGTGACCACATGGGTATGCTTGCTACAGTAATGAATGGCCTAGCGATGCGTGATGCTCTTCACCGTGCTTACGTAAATGCTCGCGTAATGTCTGCAATTCCTCTAAAAGGCGTATGTGACGACTACAATTGGGCTGATGCTATCCGCGAACTTCGCCAAGGCCGAGTTGTCATCTTCTCTGCTGGAACGGGTAACCCTTTTTTTACGACAGATTCTGCTGCTTGCTTACGTGGTATCGAAATTGAAGCTGATGTCGTACTTAAAGCGACAAAGGTCGACGGGGTATTTACGGCTGACCCTGTAGCAAACCCAGATGCAGAACTGTATGATAAGCTTTCTTATGCGGAGGTTCTGGATAAAGAGCTGAAAGTAATGGACTTA | MN253150 |
| PKGS17 | *Vibrio alginolyticus* | GGTGGCGGTAACTTGTTCCGTGGTGCAGGTCTAGCTGAAGCTGGTATGAACCGCGTAGTGGGTGACCACATGGGTATGCTTGCTACAGTAATGAATGGCCTAGCGATGCGTGATGCTCTTCACCGTGCTTACGTAAATGCTCGCGTGATGTCTGCAATTCCTCTAAAAGGCGTATGTGACGACTACAATTGGGCTGATGCTATCCGCGAACTTCGCCAAGGCCGAGTTGTCATCTTCTCTGCTGGTACTGGTAACCCTTTCTTTACAACGGATTCTGCTGCTTGCTTACGTGGTATCGAAATTGAAGCTGACGTCGTACTAAAAGCGACAAAGGTGGATGGGGTATTTACGGCTGACCCTGTAGCAAACCCAGATGCAGAACTGTATGATAAGCTTTCTTATGCAGAGGTTCTGGATAAAGAGCTGAAAGTAATGGACTTA | MN253151 |
| PKGK21 | *Vibrio alginolyticus* | GGCGGCGGTAACTTGTTCCGTGGTGCAGGTCTTGCTGAAGCTGGTATGAACCGCGTAGTAGGTGACCACATGGGTATGCTTGCAACAGTAATGAATGGCCTAGCGATGCGTGATGCTCTTCACCGTGCTTACGTAAATGCTCGCGTGATGTCTGCAATTCCTCTAAAAGGTGTATGTGACGACTACAATTGGGCTGATGCTATCCGCGAACTTCGCCAAGGCCGAGTTGTCATCTTCTCTGCTGGTACTGGTAACCCTTTTTTTACTACTGATTCTGCTGCTTGCTTACGTGGTATCGAAATTGAAGCTGACGTCGTACTAAAAGCGACAAAGGTGGATGGGGTATTTACGGCTGACCCTGTAGCAAACCCAGATGCAGAACTGTATGATAAGCTTTCTTATGCAGAAGTTCTGGATAAAGAGCTGAAAGTAATGGACTTA | MN253152 |
| PKGS28 | *Vibrio alginolyticus* | GGCGGCGGTAACTTGTTCCGTGGTGCAGGTCTTGCTGAAGCTGGTATGAACCGCGTAGTAGGTGACCACATGGGTATGCTTGCTACAGTAATGAATGGCCTAGCGATGCGTGATGCTCTTCACCGTGCTTACGTAAATGCTCGCGTAATGTCTGCAATTCCTCTAAAAGGCGTATGTGACGACTACAATTGGGCTGATGCTATCCGCGAACTTCGCCAAGGCCGAGTTGTCATCTTCTCTGCTGGTACTGGTAACCCATTCTTCACTACAGATTCTGCTGCTTGCTTACGTGGTATCGAAATTGAAGCTGACGTCGTACTAAAAGCGACAAAGGTGGATGGGGTATTTACGGCTGACCCTGTAGCAAACCCAGATGCAGAACTGTATGATAAGCTTTCTTATGCAGAGGTTCTGGATAAAGAGCTGAAAGTAATGGACTTA | MN253153 |
| PKGL29 | *Vibrio alginolyticus* | GGTGGCGGTAACTTGTTCCGTGGTGCAGGTCTAGCTGAAGCTGGTATGAACCGCGTAGTGGGTGACCACATGGGTATGCTTGCAACAGTAATGAATGGCCTAGCGATGCGTGATGCTCTTCACCGTGCATACGTAAATGCTCGCGTTATGTCTGCAATTCCTCTTAAAGGCGTATGTGACGACTACAATTGGGCTGATGCTATCCGCGAACTTCGCCAAGGCCGAGTTGTCATCTTCTCTGCTGGTACTGGTAACCCATTCTTTACTACAGATTCTGCTGCTTGTTTACGTGGTATCGAAATTGAAGCGGATGTCGTACTAAAAGCGACAAAGGTGGATGGGGTATTTACTGCTGACCCTGTAGCCAACCCAGACGCCGAACTGTATGACAAATTAAGCTATGCCGAGGTCCTGGATAAAGAACTTAAAGTAATGGACTTA | MN253154 |
| PKGS29 | *Vibrio alginolyticus* | GGTGGCGGTAACTTGTTCCGTGGTGCAGGTCTTGCTGAAGCTGGTATGAACCGCGTAGTAGGTGACCACATGGGTATGCTTGCAACAGTAATGAATGGCCTAGCGATGCGTGATGCTCTTCACCGTGCTTACGTAAATGCTCGCGTTATGTCTGCAATTCCTCTAAAAGGTGTATGTGACGACTACAATTGGGCTGATGCTATCCGCGAACTTCGCCAAGGCCGAGTTGTCATCTTCTCTGCTGGTACTGGAAACCCATTCTTCACTACAGATTCTGCTGCTTGTTTACGTGGTATCGAAATTGAAGCGGATGTCGTACTAAAAGCGACAAAGGTGGATGGGGTATTTACTGCTGACCCTGTAGCCAACCCAGACGCCGAACTGTATGACAAATTAAGCTATGCCGAGGTCCTGGATAAAGAACTTAAAGTAATGGACTTA | MN253155 |
| PKGK29 | *Vibrio alginolyticus* | GGTGGCGGTAACTTGTTCCGTGGTGCAGGTCTTGCTGAAGCTGGTATGAACCGCGTAGTAGGTGACCACATGGGTATGCTTGCTACAGTAATGAATGGCCTAGCGATGCGTGATGCTCTTCACCGTGCTTACGTAAATGCTCGCGTAATGTCTGCAATTCCTCTAAAAGGCGTATGTGACGACTACAATTGGGCTGATGCTATCCGCGAACTTCGCCAAGGCCGAGTTGTCATCTTCTCTGCAGGTACTGGTAACCCATTCTTCACTACAGATTCTGCTGCTTGTTTACGTGGTATCGAAATTGAAGCGGATGTCGTACTAAAAGCGACAAAGGTGGATGGGGTATTTACTGCTGACCCTGTTGCCAACCCAGACGCCGAACTGTATGACAAATTAAGCTATGCCGAGGTCCTGGATAAAGAGCTCAAAGTAATGGACTTA | MN253156 |
| KL26 | *Vibrio alginolyticus* | GGTGGCGGTAACTTGTTCCGTGGTGCAGGCCTAGCTGAAGCTGGTATGAACCGCGTAGTTGGTGACCACATGGGTATGCTTGCTACAGTAATGAATGGCCTAGCGATGCGTGATGCTCTTCACCGTGCATACGTAAATGCGCGCGTTATGTCTGCAATCCCTCTAAAAGGCGTATGTGACGACTATAATTGGGCTGATGCGATCCGTGAGCTTCGCCAAGGCCGAGTTGTCATCTTCTCTGCTGGTACTGGTAACCCGTTCTTTACAACGGATTCTGCTGCTTGTTTACGTGGTATCGAAATTGAAGCGGATGTTGTACTAAAAGCGACGAAAGTTGATGGTGTATTTACTGCTGACCCTGTAGCAAACCCAGACGCAGAGCTGTATGATAAGCTATCTTATGCAGAAGTTCTGGATAAAGAGCTGAAAGTTATGGACTTG | MN253157 |
| PKS03 | *Vibrio alginolyticus* | GGTGGCGGTAACTTGTTCCGTGGTGCAGGCCTAGCTGAAGCTGGTATGAACCGCGTAGTTGGTGACCACATGGGTATGCTTGCTACAGTAATGAATGGCCTAGCGATGCGTGATGCTCTTCACCGTGCATACGTAAATGCGCGCGTTATGTCTGCAATCCCTCTAAAAGGCGTATGTGACGACTATAATTGGGCTGATGCGATCCGTGAGCTTCGCCAAGGCCGAGTTGTCATCTTCTCTGCTGGTACTGGTAACCCGTTCTTTACAACGGATTCTGCTGCTTGTTTACGTGGTATCGAAATTGAAGCCGATGTCGTACTAAAAGCGACAAAAGTTGATGGTGTATTTACTGCTGACCCTGTAGCAAACCCAGACGCAGAGCTGTATGATAAGCTATCTTATGCAGAAGTTCTGGATAAAGAGCTGAAAGTTATGGATTTG | MN253158 |
| PKK04 | *Vibrio alginolyticus* | GGTGGCGGTAACTTGTTCCGTGGTGCAGGTCTAGCTGAAGCTGGTATGAACCGCGTAGTTGGTGACCACATGGGTATGCTTGCTACAGTAATGAATGGCCTAGCGATGCGTGATGCTCTTCACCGTGCTTACGTTAATGCTCGCGTTATGTCTGCAATCCCTCTTAAAGGCGTGTGTGATGACTATAATTGGGCTGATGCTATCCGAGAGCTTCGCCAAGGCCGAGTTGTCATCTTCTCTGCTGGTACTGGTAACCCGTTCTTTACAACGGATTCTGCTGCTTGTTTACGTGGTATCGAAATTGAAGCCGATGTCGTACTAAAAGCGACAAAAGTTGATGGTGTATTTACTGCTGACCCTGTAGCAAACCCAGACGCAGAGCTGTATGATAAGCTATCTTATGCAGAAGTTCTGGATAAAGAGCTGAAAGTTATGGATTTG | MN253159 |
| PKS05 | *Vibrio alginolyticus* | GGTGGCGGTAACTTGTTCCGTGGTGCAGGTCTAGCTGAAGCAGGTATGAACCGCGTAGTTGGTGACCACATGGGTATGCTTGCTACAGTAATGAATGGCCTAGCGATGCGTGATGCTCTTCACCGTGCTTACGTAAATGCTCGCGTTATGTCTGCAATTCCTCTAAAAGGTGTATGTGACGACTATAATTGGGCTGATGCTATCCGCGAACTTCGCCAAGGCCGAGTTGTCATCTTCTCTGCTGGTACTGGTAACCCTTTCTTTACAACGGATTCTGCTGCTTGCTTACGTGGTATCGAAATTGAAGCGGATGTCGTGCTAAAAGCGACAAAGGTGGATGGGGTATTCACTGCTGACCCTGTAGCAAACCCAGACGCAGAGCTGTATGATAAGCTATCTTATGCAGAAGTTCTGGATAAAGAGCTGAAAGTTATGGATTTG | MN253160 |
| PKL06 | *Vibrio alginolyticus* | GGTGGCGGTAACTTGTTCCGTGGTGCAGGTCTAGCTGAAGCAGGTATGAACCGCGTAGTTGGTGACCACATGGGTATGCTAGCTACAGTAATGAATGGCCTAGCGATGCGTGATGCTCTTCACCGCGCATACGTAAATGCTCGCGTTATGTCTGCAATTCCTCTAAAAGGTGTATGTGACGACTATAATTGGGCTGATGCTATCCGCGAACTTCGCCAAGGCCGAGTTGTCATCTTCTCTGCTGGTACTGGTAACCCTTTCTTTACAACGGATTCTGCTGCTTGCTTACGTGGTATCGAAATTGAAGCGGATGTCGTACTAAAAGCGACAAAGGTGGATGGGGTATTTACTGCTGACCCTGTAGCAAACCCAGACGCAGAGTTGTATGATAAGTTATCTTATGCTGAAGTTCTGGATAAAGAGCTGAAAGTTATGGATTTG | MN253161 |
| PKS06 | *Vibrio alginolyticus* | GGTGGCGGTAACTTGTTCCGTGGTGCAGGCCTAGCTGAAGCTGGTATGAACCGCGTAGTTGGTGACCACATGGGTATGCTTGCTACAGTAATGAATGGCCTAGCGATGCGTGATGCTCTTCACCGTGCATACGTAAATGCGCGCGTTATGTCTGCAATCCCTCTAAAAGGCGTATGTGACGACTATAATTGGGCTGATGCGATCCGTGAGCTTCGCCAAGGCCGAGTTGTCATCTTCTCTGCTGGTACTGGTAACCCGTTCTTTACAACGGATTCTGCTGCTTGTTTACGTGGTATCGAAATTGAAGCGGATGTTGTACTAAAAGCGACGAAAGTTGATGGTGTATTTACTGCTGACCCTGTAGCAAACCCAGACGCAGAACTGTATGATAAGCTATCTTATGCAGAAGTTCTGGATAAAGAGCTGAAAGTTATGGACTTG | MN253162 |
| PKL07 | *Vibrio alginolyticus* | GGTGGCGGTAACTTGTTCCGTGGTGCAGGCCTAGCTGAAGCTGGTATGAACCGCGTAGTTGGTGACCACATGGGTATGCTTGCTACAGTAATGAATGGCCTAGCGATGCGTGATGCTCTTCACCGTGCATACGTAAATGCGCGCGTTATGTCTGCAATCCCTCTAAAAGGCGTATGTGACGACTATAATTGGGCTGATGCGATCCGTGAGCTTCGCCAAGGCCGAGTTGTCATCTTCTCTGCTGGTACTGGTAACCCGTTCTTTACAACGGATTCTGCTGCTTGTTTACGTGGTATCGAAATTGAAGCGGATGTCGTACTAAAAGCGACGAAAGTTGATGGTGTATTTACTGCTGACCCTGTAGCAAACCCAGACGCAGAGCTGTATGATAAGCTATCTTATGCAGAAGTTCTGGATAAAGAGCTGAAAGTTATGGACTTG | MN253163 |
| PKS08 | *Vibrio alginolyticus* | GGTGGCGGTAACTTGTTCCGTGGTGCAGGCCTAGCTGAAGCTGGTATGAACCGCGTAGTTGGTGACCACATGGGTATGCTTGCTACAGTAATGAATGGCCTAGCGATGCGTGATGCTCTTCACCGTGCATACGTAAATGCGCGCGTTATGTCTGCAATCCCTCTAAAAGGCGTATGTGACGACTATAATTGGGCTGATGCGATCCGTGAGCTTCGCCAAGGCCGAGTTGTCATCTTCTCTGCTGGTACTGGTAACCCGTTCTTTACAACGGATTCTGCTGCTTGTTTACGTGGTATCGAAATTGAAGCGGATGTTGTACTAAAAGCGACGAAAGTTGATGGTGTATTTACTGCTGACCCTGTAGCAAACCCAGACGCAGAGCTGTATGATAAGCTATCTTATGCAGAAGTTCTGGATAAAGAGCTGAAAGTTATGGACTTG | MN253164 |
| PKS09 | *Vibrio alginolyticus* | GGTGGCGGTAACTTGTTCCGTGGTGCAGGCCTAGCTGAAGCTGGTATGAACCGCGTAGTTGGTGACCACATGGGTATGCTTGCTACAGTAATGAATGGCCTAGCGATGCGTGATGCTCTTCACCGTGCATACGTAAATGCGCGCGTTATGTCTGCAATCCCTCTAAAAGGCGTATGTGACGACTATAATTGGGCTGATGCGATCCGTGAGCTTCGCCAAGGCCGAGTTGTCATCTTCTCTGCTGGTACTGGTAACCCGTTCTTTACAACGGATTCTGCTGCTTGTTTACGTGGTATCGAAATTGAAGCGGATGTTGTACTAAAAGCGACGAAAGTTGATGGTGTATTTACTGCTGACCCTGTAGCAAACCCAGACGCAGAGCTGTATGATAAGCTATCTTATGCAGAAGTTCTGGATAAAGAGCTGAAAGTTATGGACTTG | MN253165 |
| PKK09 | *Vibrio alginolyticus* | GGTGGCGGTAACTTGTTCCGTGGTGCAGGCCTAGCTGAAGCTGGTATGAACCGCGTAGTTGGTGACCACATGGGTATGCTTGCTACAGTAATGAATGGCCTAGCGATGCGTGATGCTCTTCACCGTGCATACGTAAATGCGCGCGTTATGTCTGCAATCCCTCTAAAAGGCGTATGTGACGACTATAATTGGGCAGATGCGATCCGTGAGCTTCGCCAAGGCCGAGTTGTCATCTTCTCTGCTGGTACTGGTAACCCGTTCTTTACAACGGATTCTGCTGCTTGTTTACGTGGTATCGAAATTGAAGCGGATGTTGTACTAAAAGCGACGAAAGTTGATGGTGTATTTACTGCTGACCCTGTTGCAAACCCAGACGCAGAGCTGTATGATAAGCTATCTTATGCAGAAGTTCTGGATAAAGAGCTGAAAGTTATGGACTTG | MN253166 |
| PKL10 | *Vibrio alginolyticus* | GGTGGCGGTAACTTGTTCCGTGGTGCAGGCCTAGCTGAAGCTGGTATGAACCGCGTAGTTGGTGACCACATGGGTATGCTTGCTACAGTAATGAATGGCCTAGCGATGCGTGATGCTCTTCACCGTGCATACGTAAATGCGCGCGTTATGTCTGCAATCCCTCTAAAAGGCGTATGTGACGACTATAATTGGGCTGATGCGATCCGTGAGCTTCGCCAAGGCCGAGTTGTCATCTTCTCTGCTGGTACTGGTAACCCGTTCTTTACAACGGATTCTGCTGCTTGTTTACGTGGTATCGAAATTGAAGCGGATGTCGTACTAAAAGCGACGAAAGTTGATGGTGTATTTACTGCTGACCCTGTAGCAAACCCAGACGCAGAGCTGTATGATAAGCTATCTTATGCAGAAGTTCTGGATAAAGAGCTGAAAGTTATGGACTTG | MN253167 |
| PKS11 | *Vibrio alginolyticus* | GGTGGCGGTAACTTGTTCCGTGGTGCAGGCCTAGCTGAAGCTGGTATGAACCGCGTAGTTGGTGACCACATGGGTATGCTTGCTACAGTAATGAATGGCCTAGCGATGCGTGATGCTCTTCACCGTGCATACGTAAATGCGCGCGTTATGTCTGCAATCCCTCTAAAAGGCGTATGTGACGACTATAATTGGGCTGATGCGATCCGTGAGCTTCGCCAAGGCCGAGTTGTCATCTTCTCTGCTGGTACTGGTAACCCGTTCTTTACAACGGATTCTGCTGCTTGTTTACGTGGTATCGAAATTGAAGCGGATGTCGTACTAAAAGCGACGAAAGTTGATGGTGTATTTACTGCTGACCCTGTAGCAAACCCAGACGCAGAACTGTATGATAAGCTATCTTATGCAGAAGTTCTGGATAAAGAGCTGAAAGTTATGGACTTG | MN253168 |
| PKK11 | *Vibrio alginolyticus* | GGTGGCGGTAACTTGTTCCGTGGTGCAGGCCTAGCTGAAGCTGGTATGAACCGCGTAGTTGGTGACCACATGGGTATGCTTGCTACAGTAATGAATGGCCTAGCGATGCGTGATGCTCTTCACCGTGCATACGTAAATGCGCGCGTTATGTCTGCAATCCCTCTAAAAGGCGTATGTGACGACTATAATTGGGCTGATGCGATCCGTGAGCTTCGCCAAGGCCGAGTTGTCATCTTCTCTGCTGGTACTGGTAACCCGTTCTTTACAACGGATTCTGCTGCTTGTTTACGTGGTATCGAAATTGAAGCGGATGTTGTACTAAAGGCAACGAAAGTTGATGGTGTATTTACTGCTGACCCTGTAGCAAACCCCGACGCGGAGCTGTATGATAAATTAAGCTATGCAGAAGTTCTGGACAAAGAGCTGAAAGTTATGGACTTG | MN253169 |
| PKL12 | *Vibrio alginolyticus* | GGTGGCGGTAACTTGTTCCGTGGTGCAGGCCTAGCTGAAGCTGGTATGAACCGCGTAGTTGGTGACCACATGGGTATGCTTGCTACAGTAATGAATGGCCTAGCGATGCGTGATGCTCTTCACCGTGCATACGTAAATGCGCGCGTTATGTCTGCAATCCCTCTAAAAGGCGTATGTGACGACTATAATTGGGCTGATGCGATCCGTGAGCTTCGCCAAGGCCGAGTTGTCATCTTCTCTGCTGGTACTGGTAACCCGTTCTTTACAACGGATTCTGCTGCTTGTTTACGTGGTATCGAAATTGAAGCGGATGTCGTACTAAAAGCGACGAAAGTTGATGGTGTATTTACTGCTGACCCTGTAGCAAACCCAGACGCAGAGCTGTATGATAAGCTATCTTATGCAGAAGTTCTGGATAAAGAGCTGAAAGTTATGGACTTG | MN253170 |
| PKS12 | *Vibrio alginolyticus* | GGTGGCGGTAACTTGTTCCGTGGTGCAGGCCTAGCTGAAGCTGGTATGAACCGCGTAGTTGGTGACCACATGGGTATGCTTGCTACAGTAATGAATGGCCTAGCGATGCGTGATGCTCTTCACCGTGCATACGTAAATGCGCGCGTTATGTCTGCAATCCCTCTAAAAGGCGTATGTGACGACTATAATTGGGCTGATGCGATCCGTGAGCTTCGCCAAGGCCGAGTTGTCATCTTCTCTGCTGGTACTGGTAACCCGTTCTTTACAACGGATTCTGCTGCTTGTTTACGTGGTATCGAAATTGAAGCGGATGTTGTACTAAAAGCGACGAAAGTTGATGGTGTATTTACTGCTGACCCTGTAGCGAACCCAGACGCAGAGCTGTATGATAAGCTATCTTATGCAGAAGTTCTGGATAAAGAGCTGAAAGTTATGGACTTG | MN253171 |
| PKK12 | *Vibrio alginolyticus* | GGTGGCGGTAACTTGTTCCGTGGTGCAGGCCTAGCTGAAGCTGGTATGAACCGCGTAGTTGGTGACCACATGGGTATGCTTGCTACAGTAATGAATGGCCTAGCGATGCGTGATGCTCTTCACCGTGCATACGTAAATGCGCGCGTTATGTCTGCAATCCCTCTAAAAGGCGTATGTGACGACTATAATTGGGCTGATGCGATCCGTGAGCTTCGCCAAGGCCGAGTTGTCATCTTCTCTGCTGGTACTGGTAACCCGTTCTTTACAACGGATTCTGCTGCTTGTTTACGTGGTATCGAAATTGAAGCGGATGTTGTACTAAAAGCGACGAAAGTTGATGGTGTATTTACTGCTGACCCTGTAGCAAACCCAGACGCAGAGCTGTACGATAAGCTATCTTATGCAGAAGTTCTGGATAAAGAGCTGAAAGTTATGGACTTG | MN253172 |
| PKL13 | *Vibrio alginolyticus* | GGTGGCGGTAACTTGTTCCGTGGTGCAGGCCTAGCTGAAGCTGGTATGAACCGCGTAGTTGGTGACCACATGGGTATGCTTGCTACAGTAATGAATGGCCTAGCGATGCGTGATGCTCTTCACCGTGCATACGTAAATGCGCGCGTTATGTCTGCAATCCCTCTAAAAGGCGTATGTGACGACTATAATTGGGCTGATGCGATCCGTGAGCTTCGCCAAGGCCGAGTTGTCATCTTCTCTGCTGGTACTGGTAACCCGTTCTTTACAACGGATTCTGCTGCTTGTTTACGTGGTATCGAAATTGAAGCGGATGTCGTACTAAAAGCGACGAAAGTTGATGGTGTATTTACTGCTGACCCTGTAGCAAACCCAGACGCAGAGCTGTATGATAAGCTATCTTATGCAGAAGTTCTGGATAAAGAGCTGAAAGTTATGGACTTG | MN253173 |
| PKK13 | *Vibrio alginolyticus* | GGTGGCGGTAACTTGTTCCGTGGTGCAGGCCTAGCTGAAGCTGGTATGAACCGCGTAGTTGGTGACCACATGGGTATGCTGGCAACAGTAATGAATGGCCTTGCGATGCGTGATGCTCTTCACCGCGCATACGTAAATGCGCGCGTTATGTCTGCAATCCCTCTAAAAGGCGTATGTGACGACTATAATTGGGCTGATGCGATCCGTGAGCTTCGCCAAGGCCGAGTTGTCATCTTCTCTGCTGGTACTGGTAACCCGTTCTTTACAACGGATTCTGCTGCTTGTTTACGTGGTATCGAAATTGAAGCGGATGTCGTACTAAAAGCGACGAAAGTTGATGGTGTATTTACTGCTGACCCTGTAGCAAACCCAGACGCAGAGCTGTATGATAAGCTATCTTATGCAGAAGTTCTGGATAAAGAGCTGAAAGTTATGGACTTG | MN253174 |
| PKS14 | *Vibrio alginolyticus* | GGTGGCGGTAACTTGTTCCGTGGTGCAGGCCTTGCTGAAGCTGGTATGAACCGCGTAGTTGGTGACCACATGGGTATGCTGGCAACAGTAATGAATGGCCTTGCGATGCGTGATGCTCTTCACCGCGCATACGTAAATGCGCGCGTTATGTCTGCAATCCCTCTAAAAGGCGTATGTGACGACTATAATTGGGCTGATGCGATCCGTGAGCTTCGCCAAGGCCGAGTTGTCATCTTCTCTGCTGGTACTGGTAACCCGTTCTTTACAACGGATTCTGCTGCTTGTTTACGTGGTATCGAAATTGAAGCGGATGTCGTACTAAAAGCGACGAAAGTTGATGGTGTATTTACTGCTGACCCTGTAGCAAACCCAGACGCAGAGCTGTATGATAAGCTATCTTATGCAGAAGTTCTGGATAAAGAGCTGAAAGTTATGGACTTG | MN253175 |
| PKS15 | *Vibrio alginolyticus* | GGTGGCGGTAACTTGTTCCGTGGTGCAGGCCTAGCTGAAGCTGGTATGAACCGCGTAGTTGGTGACCACATGGGTATGCTTGCTACAGTAATGAATGGCCTAGCGATGCGCGATGCTCTTCACCGTGCATACGTAAATGCGCGCGTTATGTCTGCAATCCCTCTAAAAGGCGTATGTGACGACTATAATTGGGCTGATGCGATCCGTGAGCTTCGCCAAGGCCGAGTTGTCATCTTCTCTGCTGGTACTGGTAACCCGTTCTTTACAACGGATTCTGCTGCTTGTTTACGTGGTATCGAAATTGAAGCGGATGTCGTACTTAAAGCGACGAAAGTTGATGGTGTATTTACTGCTGACCCTGTTGCAAACCCAGACGCAGAGTTGTATGATAAGCTATCTTATGCAGAAGTTCTGGATAAAGAGCTCAAAGTTATGGACTTG | MN253176 |
| PKK15 | *Vibrio alginolyticus* | GGTGGCGGTAACTTGTTCCGTGGTGCAGGCCTAGCTGAAGCTGGTATGAACCGCGTAGTTGGTGACCACATGGGTATGCTTGCTACAGTAATGAATGGCCTAGCGATGCGTGATGCTCTTCACCGTGCATACGTAAATGCGCGCGTTATGTCTGCAATCCCTCTAAAAGGGGTATGTGACGACTATAATTGGGCTGATGCGATTCGGGAGCTTCGCCAAGGCCGAGTTGTCATCTTCTCTGCTGGTACTGGTAACCCGTTCTTTACAACGGATTCTGCTGCTTGTTTACGTGGTATCGAAATTGAAGCGGATGTTGTACTAAAAGCGACGAAAGTTGATGGTGTATTTACTGCTGACCCTGTAGCAAACCCAGACGCAGAGCTGTATGATAAGCTATCTTATGCAGAAGTTCTGGATAAAGAGCTCAAAGTTATGGACTTG | MN253177 |
| PKL16 | *Vibrio alginolyticus* | GGTGGCGGTAACTTGTTCCGTGGTGCAGGCCTAGCTGAAGCTGGTATGAACCGCGTAGTTGGTGACCACATGGGTATGCTTGCTACAGTAATGAATGGCCTAGCGATGCGTGATGCTCTTCACCGTGCATACGTAAATGCGCGCGTTATGTCTGCAATCCCTCTAAAAGGCGTATGTGACGACTATAATTGGGCTGATGCGATCCGTGAGCTTCGCCAAGGCCGAGTTGTCATCTTCTCTGCTGGTACTGGTAACCCGTTCTTTACAACGGATTCTGCTGCTTGTTTACGTGGTATCGAAATTGAAGCGGATGTTGTACTAAAAGCGACGAAAGTTGATGGTGTATTTACTGCTGACCCTGTAGCAAACCCAGACGCAGAGCTGTATGATAAGCTATCTTATGCAGAAGTTCTGGATAAAGAGCTGAAAGTTATGGACTTG | MN253178 |
| PKS16 | *Vibrio alginolyticus* | GGTGGCGGTAACTTGTTCCGTGGTGCAGGCCTAGCTGAAGCTGGTATGAACCGCGTAGTTGGTGACCACATGGGTATGCTTGCTACAGTAATGAATGGCCTAGCGATGCGTGATGCTCTTCACCGTGCATACGTAAATGCGCGCGTTATGTCTGCAATCCCTCTAAAAGGCGTATGTGACGACTATAATTGGGCTGATGCGATCCGTGAGCTTCGCCAAGGCCGAGTTGTCATCTTCTCTGCTGGTACTGGTAACCCGTTCTTTACAACGGATTCTGCTGCTTGTTTACGTGGTATCGAAATTGAAGCGGATGTTGTACTAAAAGCGACGAAAGTTGATGGTGTATTTACTGCTGACCCTGTAGCAAACCCAGACGCAGAGCTGTATGATAAGCTATCTTATGCAGAAGTTCTGGATAAAGAGCTGAAAGTTATGGACTTG | MN253179 |
| PKK16 | *Vibrio alginolyticus* | GGTGGCGGTAACTTGTTCCGTGGTGCAGGCCTAGCTGAAGCTGGTATGAACCGCGTAGTTGGTGACCACATGGGTATGCTTGCTACAGTAATGAATGGCCTAGCGATGCGTGATGCTCTTCATCGTGCTTACGTAAATGCGCGCGTTATGTCTGCAATCCCTCTAAAAGGCGTATGTGACGACTATAATTGGGCTGATGCGATCCGTGAGCTTCGCCAAGGCCGAGTTGTCATCTTCTCTGCTGGTACTGGTAACCCGTTCTTTACAACGGATTCTGCTGCTTGTTTACGTGGTATCGAAATTGAAGCGGATGTTGTACTAAAAGCAACAAAAGTTGATGGTGTATTTACTGCTGACCCTGTAGCAAACCCAGACGCAGAGCTGTATGATAAGCTATCTTATGCAGAAGTTCTGGATAAAGAGCTGAAAGTTATGGACTTG | MN253180 |
| PKS17 | *Vibrio alginolyticus* | GGTGGCGGTAACTTGTTCCGTGGTGCAGGCCTAGCTGAAGCTGGTATGAACCGCGTAGTTGGTGACCACATGGGAATGCTTGCTACAGTAATGAATGGCCTAGCGATGCGTGATGCTCTTCACCGTGCATACGTAAATGCGCGCGTTATGTCTGCAATCCCTCTAAAAGGCGTATGTGACGACTATAATTGGGCTGATGCGATCCGTGAGCTTCGCCAAGGCCGAGTTGTCATCTTCTCTGCTGGTACTGGTAACCCGTTCTTTACAACGGATTCTGCTGCTTGTTTACGTGGTATCGAAATAGAAGCGGATGTTGTACTAAAAGCGACGAAAGTTGATGGTGTATTTACTGCTGACCCTGTAGCAAACCCTGATGCAGAGCTGTATGATAAGCTATCTTATGCAGAAGTTCTGGATAAAGAGCTGAAAGTTATGGACTTG | MN253181 |
| PKK17 | *Vibrio alginolyticus* | GGTGGCGGTAACTTGTTCCGTGGTGCAGGTCTTGCTGAAGCTGGTATGAACCGCGTAGTTGGTGACCACATGGGTATGCTTGCTACAGTAATGAATGGCCTAGCGATGCGCGATGCTCTTCACCGTGCATACGTTAATGCGCGTGTTATGTCTGCCATTCCTCTAAAAGGCGTATGTGACGACTATAATTGGGCTGATGCGATCCGTGAACTTCGCCAAGGCCGGGTTGTCATCTTCTCTGCTGGTACGGGTAACCCGTTCTTTACAACGGATTCTGCTGCTTGCCTACGTGGTATTGAAATCGAAGCGGACGTTGTACTTAAAGCGACAAAGGTTGATGGGGTATTTACTGCTGACCCTGTAGCCAACCCAGACGCAGAGCTGTATGATAAGCTATCTTACGCCGAAGTTCTGGATAAAGAGCTGAAAGTGATGGACTTG | MN253182 |
| PKS18 | *Vibrio alginolyticus* | GGTGGCGGTAACTTGTTCCGTGGTGCAGGCCTAGCTGAAGCTGGTATGAACCGCGTAGTTGGTGACCACATGGGTATGCTTGCTACAGTAATGAATGGCCTAGCGATGCGTGATGCTCTTCACCGTGCATACGTAAATGCGCGCGTTATGTCTGCAATCCCTCTAAAAGGCGTATGTGACGACTATAATTGGGCTGATGCGATCCGTGAGCTTCGCCAAGGCCGAGTTGTCATCTTCTCTGCTGGTACTGGTAACCCGTTCTTTACAACGGATTCTGCTGCTTGTTTACGTGGTATCGAAATTGAAGCGGATGTCGTACTAAAAGCGACGAAAGTTGATGGTGTATTTACTGCTGACCCTGTAGCAAACCCAGACGCAGAGCTGTATGATAAGCTATCTTATGCAGAAGTTCTGGATAAAGAGCTGAAAGTTATGGACTTG | MN253183 |
| PKK18 | *Vibrio alginolyticus* | GGTGGCGGTAACTTGTTCCGTGGTGCAGGCCTAGCTGAAGCTGGTATGAACCGCGTAGTTGGTGACCACATGGGTATGCTTGCTACAGTAATGAATGGCCTAGCGATGCGTGATGCTCTTCACCGTGCATACGTAAATGCGCGCGTTATGTCTGCAATCCCTCTAAAAGGCGTATGTGACGACTATAATTGGGCTGATGCGATCCGTGAGCTTCGCCAAGGCCGAGTTGTCATCTTCTCTGCTGGTACTGGTAACCCGTTCTTTACAACGGATTCTGCTGCTTGTTTACGTGGTATCGAAATTGAAGCGGATGTCGTACTAAAAGCGACGAAAGTTGATGGTGTATTTACTGCTGACCCTGTAGCAAACCCAGACGCAGAGCTGTATGATAAGCTATCTTATGCAGAAGTTCTGGATAAAGAGCTGAAAGTTATGGACTTG | MN253184 |
| PKS20 | *Vibrio alginolyticus* | GGTGGCGGTAACTTGTTCCGTGGTGCAGGCCTAGCTGAGGCTGGTATGAACCGCGTAGTTGGTGACCACATGGGTATGCTTGCTACAGTAATGAATGGCCTAGCGATGCGTGATGCTCTTCACCGTGCATACGTAAATGCGCGCGTTATGTCTGCAATCCCTCTCAAAGGCGTATGTGACGACTATAACTGGGCTGATGCGATCCGAGAGCTTCGCCAAGGCCGAGTTGTCATCTTCTCTGCTGGTACTGGTAACCCGTTCTTTACAACGGATTCTGCTGCTTGTTTACGTGGTATCGAAATCGAAGCGGATGTTGTACTAAAGGCGACGAAAGTTGACGGTGTATTTACTGCTGACCCTGTTGCAAACCCAGACGCAGAGCTGTATGATAAGCTATCTTATGCAGAAGTTCTGGATAAAGAGCTCAAAGTTATGGACTTG | MN253185 |
| PKK21 | *Vibrio alginolyticus* | GGTGGCGGTAACTTGTTCCGTGGTGCAGGCCTAGCTGAAGCTGGTATGAACCGCGTAGTTGGTGACCACATGGGTATGCTTGCTACAGTAATGAATGGCCTAGCGATGCGTGATGCTCTTCACCGTGCATACGTAAATGCGCGCGTTATGTCTGCAATCCCTCTAAAAGGCGTATGTGACGACTATAATTGGGCTGATGCGATTCGCGAGCTTCGCCAAGGCCGAGTTGTCATCTTCTCTGCTGGTACTGGTAACCCGTTCTTTACAACGGATTCTGCTGCTTGTTTACGTGGTATCGAAATCGAAGCGGATGTTGTACTAAAGGCGACGAAAGTTGACGGTGTATTTACTGCTGACCCTGTTGCAAACCCAGACGCAGAGCTGTATGATAAGCTATCTTATGCAGAAGTTCTGGATAAAGAGCTCAAAGTTATGGACTTG | MN253186 |
| PKL22 | *Vibrio alginolyticus* | GGTGGCGGTAACTTGTTCCGTGGTGCAGGCCTAGCTGAAGCTGGTATGAACCGCGTAGTTGGTGACCACATGGGTATGCTTGCTACAGTAATGAATGGCCTAGCGATGCGTGATGCTCTTCACCGTGCATACGTAAATGCGCGCGTTATGTCTGCAATCCCTCTAAAAGGCGTATGTGACGACTATAATTGGGCTGATGCGATCCGTGAGCTTCGCCAAGGCCGAGTTGTCATCTTCTCTGCTGGTACTGGGAATCCCTTTTTTACAACGGATTCTGCTGCTTGTTTACGTGGTATCGAAATTGAAGCGGATGTCGTACTAAAAGCGACGAAAGTCGATGGTGTATTTACTGCTGACCCTGTTGCAAACCCAGACGCAGAGCTGTATGATAAGCTATCTTATGCAGAAGTTCTGGATAAAGAGCTCAAAGTTATGGACTTG | MN253187 |
| PKS22 | *Vibrio alginolyticus* | GGTGGCGGTAACTTGTTCCGTGGTGCAGGCCTAGCTGAAGCTGGTATGAACCGCGTAGTTGGTGACCACATGGGTATGCTTGCTACAGTAATGAATGGCCTAGCGATGCGTGATGCTCTTCACCGTGCATACGTAAATGCGCGCGTTATGTCTGCAATCCCTCTAAAAGGCGTATGTGACGACTATAATTGGGCTGATGCGATCCGTGAGCTTCGCCAAGGCCGAGTTGTCATCTTCTCTGCTGGTACTGGGAATCCCTTTTTTACAACGGATTCTGCTGCTTGTTTACGTGGTATCGAAATTGAAGCGGATGTCGTACTAAAAGCGACGAAAGTCGATGGTGTATTTACTGCTGACCCTGTTGCAAACCCAGACGCAGAGCTGTATGATAAGCTATCTTATGCAGAAGTTCTGGATAAAGAGCTCAAAGTTATGGACTTG | MN253188 |
| PKK22 | *Vibrio alginolyticus* | GGTGGCGGTAACTTGTTCCGTGGTGCAGGCCTAGCTGAAGCTGGTATGAACCGCGTAGTTGGTGACCACATGGGTATGCTTGCTACAGTAATGAATGGCCTAGCGATGCGTGATGCTCTTCACCGTGCATACGTAAATGCGCGCGTTATGTCTGCAATCCCTCTAAAAGGCGTATGTGACGACTATAATTGGGCTGATGCGATCCGTGAGCTTCGCCAAGGCCGAGTTGTCATCTTCTCTGCTGGTACTGGGAATCCCTTTTTTACAACGGATTCTGCTGCTTGTTTACGTGGTATCGAAATTGAAGCGGATGTAGTACTAAAAGCGACGAAAGTCGATGGTGTATTTACTGCTGACCCTGTTGCAAATCCAGACGCAGAGCTGTATGATAAGCTATCTTATGCAGAAGTTCTGGATAAAGAGCTCAAAGTTATGGACTTG | MN253189 |
| PKL24 | *Vibrio alginolyticus* | GGTGGCGGTAACTTGTTCCGTGGTGCAGGTCTAGCTGAAGCAGGTATGAACCGCGTAGTTGGTGACCACATGGGTATGCTTGCTACAGTAATGAATGGCCTAGCGATGCGTGATGCTCTTCACCGTGCTTACGTAAATGCTCGCGTTATGTCTGCAATTCCTCTAAAAGGTGTATGTGACGACTATAATTGGGCTGATGCTATCCGCGAACTTCGCCAAGGCCGAGTTGTCATCTTCTCTGCTGGTACTGGTAACCCTTTCTTTACAACGGATTCTGCTGCTTGCTTACGTGGTATCGAAATTGAAGCGGATGTCGTACTTAAAGCGACGAAAGTTGATGGTGTATTTACTGCTGACCCTGTTGCAAACCCAGACGCAGAGTTGTATGATAAGCTATCTTATGCAGAAGTTCTGGATAAAGAGCTCAAAGTTATGGACTTG | MN253190 |
| PKS24 | *Vibrio alginolyticus* | GGTGGCGGTAACTTGTTCCGTGGTGCAGGTCTAGCTGAAGCAGGTATGAACCGCGTAGTTGGTGACCACATGGGTATGCTTGCTACAGTAATGAATGGCCTAGCGATGCGTGATGCTCTTCACCGTGCTTACGTAAATGCTCGCGTTATGTCTGCAATTCCTCTAAAAGGTGTATGTGACGACTATAATTGGGCTGATGCTATCCGCGAACTTCGCCAAGGCCGAGTTGTCATCTTCTCTGCTGGTACTGGTAACCCTTTCTTTACAACGGATTCTGCTGCTTGCTTACGTGGTATCGAAATTGAAGCGGATGTCGTACTAAAAGCGACAAAGGTGGATGGGGTATTTACTGCTGACCCTGTTGCAAACCCAGACGCAGAGTTGTATGATAAGCTATCTTATGCAGAAGTTCTGGATAAAGAGCTCAAAGTTATGGACTTG | MN253191 |
| NS01 | *Vibrio alginolyticus* | GGTGGTGGTAACTTGTTCCGTGGTGCAGGTCTAGCCGAGGCTGGTATGAACCGCGTAGTGGGTGACCACATGGGTATGCTTGCTACAGTAATGAATGGCCTAGCGATGCGTGATGCTCTGCACCGTGCATACGTAAATGCTCGCGTGATGTCTGCAATTCCTCTCAAAGGCGTATGTGACGACTACAATTGGGCTGATGCAATTCGTGAACTTCGCCAAGGCCGAGTGGTCATCTTCTCGGCTGGCACTGGCAACCCATTTTTTACAACGGATTCTGCTGCTTGTCTGCGTGGTATCGAAATTGAAGCGGATGTAGTTCTAAAAGCAACAAAAGTGGATGGCGTTTTTACTGCTGACCCGGTAGCCAACCCTGACGCAGAGTTGTATGATAAGCTATCTTATGCAGAAGTTCTGGATAAAGAGCTCAAAGTTATGGACTTG | MN253192 |
| NL03 | *Vibrio alginolyticus* | GGTGGTGGTAACTTGTTCCGTGGTGCAGGTCTAGCCGAGGCTGGTATGAACCGCGTAGTGGGTGACCACATGGGTATGCTTGCTACAGTAATGAATGGCCTAGCGATGCGTGATGCTCTGCACCGTGCATACGTAAATGCTCGCGTGATGTCTGCAATTCCTCTCAAAGGCGTATGTGACGACTACAATTGGGCTGATGCAATTCGTGAACTTCGCCAAGGCCGAGTGGTCATCTTCTCGGCTGGTACTGGCAACCCATTCTTTACAACGGATTCTGCTGCTTGTCTGCGTGGTATCGAAATTGAAGCGGATGTAGTTCTAAAAGCAACAAAAGTGGATGGCGTTTTTACTGCTGACCCGGTAGCCAACCCTGACGCAGAGTTGTATGATAAGCTATCTTATGCAGAAGTTCTGGATAAAGAGCTCAAAGTTATGGACTTG | MN253193 |
| NL04 | *Vibrio alginolyticus* | GGTGGTGGTAACTTGTTCCGTGGTGCAGGTCTAGCTGAAGCTGGTATGAACCGCGTAGTGGGTGACCACATGGGTATGCTTGCTACAGTAATGAATGGCCTAGCGATGCGTGATGCTCTTCACCGTGCTTACGTAAATGCTCGCGTAATGTCTGCAATTCCTCTAAAAGGTGTATGTGACGACTACAATTGGGCTGATGCTATCCGCGAACTTCGCCAAGGCCGAGTGGTCATCTTCTCTGCTGGTACTGGTAACCCATTCTTCACAACGGATTCTGCTGCTTGTTTACGTGGTATCGAAATTGAAGCGGATGTCGTACTAAAAGCGACAAAGGTTGATGGTGTATTTACTGCTGACCCTGTAGCAAACCCAGATGCAGAGTTGTATGATAAGCTATCTTATGCAGAAGTTCTGGATAAAGAGCTCAAAGTTATGGACTTG | MN253194 |
| NS04 | *Vibrio alginolyticus* | GGTGGCGGTAACTTGTTCCGTGGTGCAGGTCTAGCTGAAGCTGGTATGAACCGCGTAGTGGGTGACCACATGGGTATGCTTGCAACAGTAATGAATGGCCTAGCGATGCGTGATGCTCTTCACCGTGCTTACGTAAATGCTCGCGTGATGTCTGCAATTCCTCTTAAAGGTGTATGTGACGACTACAATTGGGCTGATGCTATCCGCGAACTTCGCCAAGGCCGAGTTGTCATCTTCTCTGCTGGTACTGGTAACCCATTTTTCACAACGGATTCTGCTGCTTGTTTACGTGGTATCGAAATTGAAGCGGATGTCGTACTAAAAGCGACAAAGGTTGATGGTGTATTTACTGCTGACCCTGTAGCAAACCCAGATGCAGAGTTGTATGATAAGCTATCTTATGCAGAAGTTCTGGATAAAGAGCTCAAAGTTATGGACTTG | MN253195 |
| NL05 | *Vibrio alginolyticus* | GGTGGCGGTAACTTGTTCCGTGGTGCAGGTCTTGCTGAGGCTGGTATGAACCGCGTAGTGGGTGACCACATGGGTATGCTTGCTACAGTAATGAATGGCCTAGCGATGCGTGATGCTCTTCACCGTGCATACGTAAATGCGCGCGTAATGTCTGCAATTCCTCTAAAAGGCGTATGTGACGACTACAATTGGGCTGATGCAATCCGCGAACTTCGCCAAGGCCGAGTTGTCATCTTCTCTGCTGGTACTGGTAACCCGTTCTTCACAACGGATTCTGCTGCTTGTTTACGTGGTATCGAAATTGAAGCGGATGTCGTACTAAAAGCGACAAAGGTGGATGGGGTATTTACTGCTGACCCCGTTGCAAACCCAGATGCAGAGTTGTATGATAAGCTATCTTATGCAGAAGTTCTGGATAAAGAGCTCAAAGTTATGGACTTG | MN253196 |
| NL12 | *Vibrio alginolyticus* | GGCGGCGGTAACTTGTTCCGTGGTGCAGGTCTAGCTGAAGCTGGTATGAACCGCGTAGTGGGTGACCACATGGGTATGCTTGCTACAGTAATGAATGGCCTAGCGATGCGTGATGCTCTTCACCGTGCTTACGTAAATGCTCGCGTAATGTCTGCAATTCCGCTAAAAGGTGTATGTGACGACTACAATTGGGCTGATGCTATCCGCGAACTTCGCCAAGGCCGAGTGGTCATCTTCTCTGCTGGTACTGGTAACCCATTCTTCACAACGGATTCTGCTGCTTGTTTACGTGGTATCGAAATTGAAGCGGATGTCGTACTAAAAGCGACAAAGGTTGATGGTGTATTTACTGCTGACCCTGTAGCAAACCCAGACGCAGAGTTGTATGATAAGCTATCTTATGCAGAAGTTCTGGATAAAGAGCTCAAAGTTATGGACTTG | MN253197 |
| NL13 | *Vibrio alginolyticus* | GGTGGTGGTAACTTGTTCCGTGGTGCAGGTCTAGCCGAAGCTGGTATGAACCGCGTAGTGGGTGACCACATGGGTATGCTTGCTACAGTAATGAATGGCCTAGCGATGCGTGATGCTCTGCACCGTGCATACGTAAATGCTCGCGTGATGTCTGCAATTCCTCTCAAAGGCGTATGTGACGACTACAATTGGGCTGATGCAATTCGTGAACTTCGCCAAGGGCGAGTGGTCATCTTCTCGGCTGGCACTGGCAACCCATTTTTTACAACGGATTCTGCTGCTTGTCTGCGTGGTATCGAAATTGAAGCGGATGTAGTTCTAAAAGCAACAAAAGTGGATGGCGTTTTTACTGCTGACCCGGTAGCCAACCCTGACGCAGAGTTGTATGATAAGCTATCTTATGCAGAAGTTCTGGATAAAGAGCTCAAAGTTATGGACTTG | MN253198 |
| NL20 | *Vibrio alginolyticus* | GGTGGTGGTAACTTGTTCCGTGGTGCAGGTCTTGCAGAAGCGGGTATGAACCGCGTAGTGGGTGACCACATGGGTATGCTTGCTACAGTAATGAATGGCCTAGCGATGCGTGATGCTCTTCACCGTGCTTACGTAAATGCTCGCGTAATGTCTGCAATTCCTCTAAAAGGTGTATGTGACGACTACAATTGGGCTGATGCTATCCGCGAACTTCGCCAAGGCCGAGTGGTCATCTTCTCTGCTGGTACTGGTAACCCATTCTTCACAACGGATTCTGCTGCTTGTTTACGTGGTATCGAAATTGAAGCGGATGTCGTACTAAAAGCGACAAAGGTTGATGGTGTATTTACTGCTGACCCTGTAGCAAACCCAGACGCAGAGTTGTATGATAAGCTATCTTATGCAGAAGTTCTGGATAAAGAGCTCAAAGTTATGGACTTG | MN253199 |
| NL24 | *Vibrio alginolyticus* | GGTGGCGGTAACTTGTTCCGTGGTGCTGGTCTAGCGGAAGCTGGTATGAACCGTGTTGTGGGTGACCACATGGGTATGCTAGCTACAGTAATGAATGGCCTAGCGATGCGTGATGCTCTTCACCGTGCTTACGTAAATGCTCGCGTGATGTCTGCAATTCCTCTAAAAGGCGTATGTGACGACTACAATTGGGCTGATGCTATCCGCGAACTTCGCCAAGGCCGAGTTGTCATCTTCTCTGCTGGTACTGGTAACCCTTTCTTTACAACGGATTCTGCTGCTTGTTTACGTGGTATCGAAATTGAAGCGGATGTAGTACTAAAAGCGACGAAAGTCGATGGTGTATTTACTGCTGACCCTGTTGCAAATCCAGACGCAGAGCTGTATGATAAGCTATCTTATGCAGAAGTTCTGGATAAAGAGCTCAAAGTTATGGACTTG | MN253200 |
| NS24 | *Vibrio alginolyticus* | GGTGGCGGTAACTTGTTCCGTGGTGCAGGTCTTGCAGAAGCTGGTATGAACCGCGTAGTGGGTGACCACATGGGTATGCTTGCTACAGTAATGAATGGCCTAGCGATGCGTGATGCTCTTCACCGTGCATACGTAAATGCGCGCGTAATGTCTGCAATTCCTCTAAAAGGCGTATGTGACGACTACAATTGGGCTGATGCAATCCGCGAACTTCGCCAAGGCCGAGTTGTCATCTTCTCTGCTGGTACTGGTAACCCGTTCTTCACAACGGATTCTGCTGCTTGCTTACGTGGTATCGAAATTGAAGCGGATGTCGTACTAAAAGCGACAAAGGTGGATGGGGTATTTACTGCTGACCCTGTAGCAAACCCAGACGCAGAGTTGTATGATAAGCTATCTTATGCAGAAGTTCTGGATAAAGAGCTCAAAGTTATGGACTTG | MN253201 |
| NK24 | *Vibrio alginolyticus* | GGTGGCGGTAACTTGTTCCGTGGTGCAGGTCTTGCTGAAGCTGGTATGAACCGCGTAGTGGGTGACCACATGGGTATGCTTGCTACAGTAATGAATGGCCTAGCGATGCGTGATGCTCTTCACCGTGCATACGTAAATGCGCGCGTAATGTCTGCAATTCCTCTAAAAGGCGTATGTGACGACTACAATTGGGCTGATGCAATCCGCGAACTTCGCCAAGGCCGAGTTGTCATCTTCTCTGCTGGTACTGGTAACCCGTTCTTCACAACGGATTCTGCTGCTTGTTTACGTGGTATCGAAATTGAAGCGGATGTCGTACTAAAAGCGACAAAGGTGGATGGGGTATTTACTGCTGACCCTGTAGCAAACCCAGACGCAGAGTTGTATGATAAGCTATCTTATGCAGAAGTTCTGGATAAAGAGCTCAAAGTTATGGACTTG | MN253202 |
| NL25 | *Vibrio alginolyticus* | GGTGGCGGTAACTTGTTCCGTGGTGCAGGTCTAGCTGAAGCTGGTATGAACCGCGTAGTGGGTGACCACATGGGTATGCTTGCTACAGTAATGAATGGCCTAGCGATGCGTGATGCTCTTCACCGTGCTTACGTAAATGCTCGCGTAATGTCTGCAATTCCTCTAAAAGGCGTATGTGACGACTACAATTGGGCTGATGCTATCCGCGAACTTCGCCAAGGCCGAGTTGTCATCTTCTCTGCTGGTACTGGTAACCCCTTCTTCACAACGGATTCTGCTGCTTGTTTACGTGGTATCGAAATTGAAGCGGATGTCGTACTAAAAGCGACAAAGGTTGATGGTGTATTTACTGCTGACCCTGTAGCAAACCCAGACGCAGAGTTGTATGATAAGCTATCTTATGCAGAAGTTCTGGATAAAGAGCTCAAAGTTATGGACTTG | MN253203 |
| NK26 | *Vibrio alginolyticus* | GGTGGCGGTAACTTGTTCCGTGGTGCAGGTCTTGCTGAAGCTGGTATGAACCGCGTAGTAGGCGACCACATGGGTATGCTTGCTACAGTAATGAATGGCCTAGCGATGCGTGATGCTCTTCACCGTGCATACGTAAATGCGCGCGTAATGTCTGCAATTCCTCTAAAAGGCGTATGTGACGACTACAATTGGGCTGATGCTATTCGCGAACTTCGCCAGGGCCGAGTTGTCATCTTCTCTGCTGGTACTGGTAACCCGTTCTTCACAACGGATTCTGCTGCTTGTTTACGTGGTATCGAAATTGAAGCGGATGTCGTACTAAAAGCGACAAAGGTGGATGGGGTATTTACTGCTGACCCTGTAGCAAACCCAGACGCAGAGTTGTATGATAAGCTATCTTATGCAGAAGTTCTGGATAAAGAGCTCAAAGTTATGGACTTG | MN253204 |
| PKGL21 | *V. campbellii* | GGCGGTGGTAACCTGTTCCGTGGTGCTGGTCTTGCGGAAGCGGGTATGAACCGTGTAGTAGGCGACCACATGGGTATGCTTGCAACAGTAATGAACGGTCTAGCAATGCGTGATGCACTTCACCGTGCTTACGTAAACGCTCGTGTAATGTCTGCAATTCCTCTAAAAGGCGTGTGTGACGACTACAATTGGGCAGACGCTATCCGCGAACTTCGCCAAGGCCGTGTAGTAATCTTCTCTGCAGGTACTGGTAACCCATTCTTCACTACAGATTCAGCAGCGTGTCTACGCGGTATCGAAATCGAAGCTGACGTAGTGCTAAAAGCAACGAAAGTTGATGGCGTATTTACTGCTGACCCAGTAGCAAACCCAGACGCAGAGCTGTATGATAAGCTATCTTACAATGAAGTTCTGGATAAAGAACTGAAAGTAATGGACTTG | MN253205 |
| PKGL25 | *V. campbellii* | GGCGGTGGTAACCTGTTCCGTGGTGCTGGTCTTGCGGAAGCGGGTATGAACCGTGTAGTAGGCGACCACATGGGTATGCTTGCAACGGTAATGAACGGTCTAGCAATGCGTGATGCACTTCACCGTGCTTACGTAAACGCTCGTGTAATGTCTGCAATTCCTCTAAAAGGCGTGTGTGACGACTACAATTGGGCAGACGCTATCCGCGAACTTCGCCAAGGCCGTGTAGTAATCTTCTCTGCAGGTACTGGTAACCCATTCTTCACTACAGATTCAGCAGCGTGTCTACGCGGTATCGAAATCGAAGCTGACGTAGTGCTAAAAGCAACGAAAGTTGATGGCGTATTTACTGCTGACCCAGTAGCAAACCCAGACGCAGAGCTGTATGATAAGCTATCTTACAATGAAGTTCTGGATAAAGAACTGAAAGTAATGGACTTG | MN253206 |
| PKGS25 | *V. campbellii* | GGCGGTGGTAACCTGTTCCGTGGTGCTGGTCTTGCGGAAGCGGGTATGAACCGTGTAGTAGGCGACCACATGGGTATGCTTGCAACGGTAATGAACGGTCTAGCAATGCGTGATGCACTTCACCGTGCTTACGTAAACGCTCGTGTAATGTCTGCAATTCCTCTAAAAGGCGTGTGTGACGACTACAATTGGGCAGACGCTATCCGCGAACTTCGCCAAGGCCGTGTAGTAATCTTCTCTGCAGGTACTGGTAACCCATTCTTCACTACAGATTCAGCAGCGTGTCTACGCGGTATCGAAATCGAAGCTGACGTAGTGCTAAAAGCAACGAAAGTTGATGGCGTATTTACTGCTGACCCAGTAGCAAACCCAGACGCAGAGCTGTATGATAAGCTATCTTACAATGAAGTTCTGGATAAAGAACTGAAAGTAATGGACTTG | MN253207 |
| PKGL26 | *V. campbellii* | GGCGGTGGTAACCTGTTCCGTGGTGCTGGTCTTGCGGAAGCGGGTATGAACCGTGTAGTAGGCGACCACATGGGTATGCTTGCAACGGTAATGAACGGTCTAGCAATGCGTGATGCACTTCACCGTGCTTACGTAAACGCTCGTGTAATGTCTGCAATTCCTCTAAAAGGCGTGTGTGACGACTACAATTGGGCAGACGCTATCCGCGAACTTCGCCAAGGCCGTGTAGTAATCTTCTCTGCAGGTACTGGTAACCCATTCTTCACTACAGATTCAGCAGCGTGTCTACGCGGTATCGAAATCGAAGCTGACGTAGTGCTAAAAGCAACGAAAGTTGATGGCGTATTTACTGCTGACCCAGTAGCAAACCCAGACGCAGAGCTTTATGATAAGCTATCTTACAATGAAGTTTTGGATAAAGAACTGAAAGTAATGGACTTG | MN253208 |
| PKGS26 | *V. campbellii* | GGCGGTGGTAACCTGTTCCGTGGTGCTGGTCTTGCGGAAGCGGGTATGAACCGTGTAGTAGGCGACCACATGGGTATGCTTGCAACGGTAATGAACGGTCTAGCAATGCGTGATGCACTTCACCGTGCTTACGTAAACGCTCGTGTAATGTCTGCAATTCCTCTAAAAGGCGTGTGTGACGACTACAATTGGGCAGACGCTATCCGCGAACTTCGCCAAGGCCGTGTAGTAATCTTCTCTGCAGGTACTGGTAACCCATTCTTCACTACAGATTCAGCAGCGTGTCTACGCGGTATTGAAATCGAAGCTGACGTGGTGCTAAAAGCAACGAAAGTTGATGGCGTATTTACTGCTGACCCAGTAGCCAACCCAGACGCTGAGCTGTATGATAAGCTATCTTACAATGAAGTTCTGGATAAAGAACTCAAAGTTATGGACTTG | MN253209 |
| PKGL28 | *V. campbellii* | GGCGGTGGTAACCTGTTCCGTGGTGCTGGTCTTGCGGAAGCGGGTATGAACCGTGTAGTAGGCGACCACATGGGTATGCTTGCAACAGTAATGAACGGTCTAGCAATGCGTGATGCACTTCACCGTGCTTACGTAAACGCTCGTGTAATGTCTGCAATTCCTCTAAAAGGCGTGTGTGACGACTACAATTGGGCAGACGCTATCCGCGAACTTCGCCAAGGCCGTGTAGTAATCTTCTCTGCAGGTACTGGTAACCCATTCTTCACTACAGATTCAGCAGCGTGTCTACGCGGTATCGAAATCGAAGCTGACGTAGTGCTAAAAGCAACGAAAGTTGATGGCGTATTTACTGCTGACCCAGTAGCAAACCCAGACGCAGAGCTGTATGATAAGCTATCTTACAATGAAGTTCTGGATAAAGAACTGAAAGTAATGGACTTG | MN253210 |
| KS11 | *V. campbellii* | GGTGGTGGTAACCTGTTCCGTGGTGCTGGTCTTGCTGAAGCTGGTATGAACCGTGTAGTGGGTGACCACATGGGTATGCTTGCTACAGTAATGAACGGTCTAGCAATGCGTGATGCTCTTCACCGTGCTTACGTAAATGCTCGCGTAATGTCTGCAATTCCTCTAAAAGGCGTGTGTGACGACTACAATTGGGCTGATGCTATCCGCGAACTTCGCCAAGGCCGTGTTGTAATCTTCTCTGCAGGTACTGGTAACCCATTCTTCACTACAGATTCTGCTGCGTGTCTACGCGGTATCGAAATCGAAGCTGACGTAGTGCTAAAAGCAACGAAAGTTGATGGCGTATTTACTGCTGACCCAGTAGCAAACCCAGACGCAGAGCTGTATGATAAGCTATCTTACAATGAAGTTCTGGATAAAGAACTGAAAGTAATGGACTTG | MN253211 |
| KK24 | *V. campbellii* | GGCGGTGGTAACCTGTTCCGTGGTGCTGGTCTTGCGGAAGCGGGTATGAACCGTGTAGTAGGCGACCACATGGGTATGCTTGCAACAGTAATGAACGGTCTAGCAATGCGTGATGCACTTCACCGTGCTTACGTAAACGCTCGTGTAATGTCTGCAATTCCTCTAAAAGGCGTGTGTGACGACTACAATTGGGCAGACGCTATCCGCGAACTTCGCCAAGGCCGTGTAGTAATCTTCTCTGCAGGTACTGGTAACCCATTCTTCACTACAGATTCAGCAGCGTGTCTACGCGGTATCGAAATCGAAGCTGACGTAGTGCTAAAAGCAACGAAAGTTGATGGCGTATTTACTGCTGACCCAGTAGCAAACCCAGACGCAGAGCTGTATGATAAGCTATCTTACAATGAAGTTCTGGATAAAGAACTGAAAGTAATGGACTTG | MN253212 |
| NS30 | *V. campbellii* | GGCGGTGGTAACCTGTTCCGTGGTGCTGGTCTTGCTGAGGCGGGTATGAACCGTGTAGTAGGCGACCACATGGGTATGCTTGCAACGGTAATGAACGGTCTAGCAATGCGTGATGCACTTCACCGTGCTTACGTAAACGCTCGTGTAATGTCTGCAATTCCTCTAAAAGGCGTGTGTGACGACTACAATTGGGCAGACGCTATCCGCGAACTTCGCCAAGGCCGTGTAGTAATCTTCTCTGCAGGTACTGGTAACCCATTCTTCACTACAGATTCAGCAGCGTGTCTACGCGGTATCGAAATCGAAGCTGACGTAGTGCTAAAAGCAACGAAAGTTGATGGCGTATTTACTGCTGACCCAGTAGCAAACCCAGACGCAGAGCTGTATGATAAGCTATCTTACAATGAAGTTCTGGATAAAGAACTGAAAGTAATGGACTTG | MN253213 |
| LS01 | *Vibrio communis* | GGCGGTGGTAACCTTTTCCGTGGTGCAGGTCTTGCAGAAGCGGGTATGAACCGCGTAGTAGGCGACCACATGGGTATGCTTGCAACAGTAATGAACGGTCTAGCAATGCGTGATGCACTTCACCGTGCTTATGTAAACGCTCGCGTAATGTCTGCAATTCCGCTAAAAGGTGTGTGTGACGATTACAACTGGGCAGACGCTATCCGCGAACTTCGCCAAGGCCGTGTAGTGATCTTCTCTGCAGGTACAGGTAACCCATTCTTTACGACGGATTCAGCAGCTTGTCTACGTGGTATCGAAATCGAAGCTGACGTAGTTCTAAAAGCGACAAAAGTTGATGGTGTATTTACTGCTGACCCAGTAGCAAACCCAGACGCAGAGCTGTATGATAAGCTATCTTACACTGAAATTCTGGATAAGGAACTGAAAGTAATGGACTTG | MN253214 |
| LK01 | *Vibrio communis* | GGCGGTGGTAACTTGTTCCGTGGTGCAGGTCTTGCAGAAGCGGGTATGAACCGCGTAGTAGGCGACCACATGGGTATGCTTGCAACAGTAATGAACGGTCTAGCAATGCGTGATGCACTTCACCGTGCTTATGTAAACGCTCGCGTAATGTCTGCAATTCCGCTAAAAGGTGTGTGTGACGATTACAACTGGGCAGACGCTATCCGCGAACTTCGCCAAGGCCGTGTAGTGATCTTCTCTGCAGGTACAGGTAACCCATTCTTTACGACGGATTCAGCAGCTTGTCTACGTGGTATCGAAATCGAAGCTGACGTAGTTCTAAAAGCGACAAAAGTTGATGGTGTATTTACTGCTGACCCAGTGGCAAACCCAGACGCAGAGCTGTATGATAAGCTATCTTACACAGAAATTCTGGATAAAGAATTGAAGGTAATGGACTTG | MN253215 |
| LL02 | *Vibrio communis* | GGCGGTGGTAACTTGTTCCGTGGTGCAGGTCTTGCAGAAGCGGGTATGAACCGCGTAGTAGGCGACCACATGGGTATGCTTGCAACAGTAATGAACGGTCTAGCAATGCGTGATGCACTTCACCGTGCTTATGTAAACGCTCGCGTAATGTCTGCAATTCCGCTAAAGGGTGTGTGTGACGATTACAACTGGGCAGACGCTATCCGCGAACTTCGCCAAGGCCGTGTAGTGATCTTCTCTGCAGGTACAGGTAACCCATTCTTTACGACGGATTCAGCAGCTTGTCTACGTGGTATTGAAATTGAAGCTGACGTAGTTCTAAAAGCGACAAAAGTTGATGGTGTATTTACTGCTGACCCAGTGGCAAACCCAGACGCAGAGCTGTACGATAAACTATCTTACACTGAAATTCTGGATAAAGAATTGAAGGTAATGGACTTG | MN253216 |
| LS02 | *Vibrio communis* | GGCGGTGGTAACTTGTTCCGTGGTGCAGGTCTTGCAGAAGCGGGTATGAACCGCGTAGTAGGCGACCACATGGGTATGCTTGCAACAGTAATGAACGGTCTAGCAATGCGTGATGCACTTCACCGTGCTTATGTAAACGCTCGCGTAATGTCTGCAATTCCGCTAAAAGGTGTGTGTGACGATTACAACTGGGCAGACGCTATCCGCGAACTTCGCCAAGGCCGTGTAGTGATCTTCTCTGCAGGTACAGGTAACCCATTCTTTACGACGGATTCAGCAGCTTGTCTACGTGGTATCGAAATCGAAGCTGACGTAGTTCTAAAAGCGACAAAAGTTGATGGTGTATTTACTGCTGACCCAGTAGCAAACCCAGACGCAGAGCTGTATGATAAGCTATCTTACACTGAAATTCTGGATAAAGAATTGAAAGTAATGGACTTG | MN253217 |
| LK02 | *Vibrio communis* | GGTGGTGGTAACCTCTTTCGCGGTGCTGGTCTTGCAGAAGCTGGTATGAACCGCGTAGTAGGCGACCACATGGGTATGCTTGCAACAGTAATGAACGGTCTAGCAATGCGTGACGCACTTCACCGTGCTTATGTAAACGCTCGCGTGATGTCTGCAATTCCGCTAAAAGGTGTGTGTGACGATTACAATTGGGCAGACGCTATCCGCGAACTTCGCCAAGGCCGTGTAGTTATCTTCTCTGCAGGTACTGGTAACCCATTCTTCACGACAGATTCTGCAGCGTGTCTACGCGGCATCGAAATCGAAGCTGACGTAGTTCTAAAAGCAACGAAAGTTGATGGTGTATTTACTGCTGACCCAGTAGCAAACCCAGACGCAGAGCTGTATGATAAGCTATCTTACACTGAAATTCTGGATAAGGAACTGAAAGTAATGGACTTG | MN253218 |
| LL03 | *Vibrio communis* | GGCGGTGGTAACTTGTTCCGTGGTGCAGGTCTTGCAGAAGCGGGTATGAACCGCGTAGTAGGCGACCACATGGGTATGCTTGCAACAGTAATGAACGGTCTAGCAATGCGTGATGCACTTCACCGTGCTTATGTAAACGCTCGCGTAATGTCTGCAATTCCGCTAAAAGGTGTGTGTGACGATTACAACTGGGCAGACGCTATCCGCGAACTTCGCCAAGGCCGTGTAGTGATCTTCTCTGCAGGTACAGGTAACCCATTCTTTACGACGGATTCAGCAGCTTGTCTACGTGGTATCGAAATCGAAGCTGACGTAGTTCTAAAAGCGACAAAAGTTGATGGTGTATTTACTGCTGACCCAGTAGCAAACCCAGACGCAGAGCTGTACGATAAGCTTTCTTACACTGAAATTCTGGATAAGGAACTGAAAGTAATGGACTTG | MN253219 |
| LS03 | *Vibrio communis* | GGCGGTGGTAACTTGTTCCGTGGTGCAGGTCTTGCAGAAGCGGGTATGAACCGCGTAGTAGGCGACCACATGGGTATGCTTGCAACAGTAATGAACGGTCTAGCAATGCGTGATGCACTTCACCGTGCTTATGTAAACGCTCGCGTAATGTCTGCAATTCCGCTAAAAGGTGTGTGTGACGATTACAACTGGGCAGACGCTATCCGCGAACTTAGGCAAGGCCGTGTAGTGATCTTCTCTGCAGGTACAGGAAACCCATTCTTTACGACGGATTCAGCAGCTTGTCTACGTGGTATCGAAATCGAAGCTGATGTTGTTCTAAAAGCGACAAAAGTTGACGGTGTATTTACTGCTGACCCAGTAGCAAACCCAGACGCAGAGCTGTACGACAAACTATCTTACACTGAAATTCTGGATAAGGAACTGAAAGTAATGGACTTG | MN253220 |
| LK03 | *Vibrio communis* | GGCGGTGGTAACTTGTTCCGTGGTGCAGGTCTTGCAGAAGCGGGTATGAACCGCGTAGTAGGCGACCACATGGGTATGCTTGCAACAGTAATGAACGGTCTAGCAATGCGTGATGCACTTCACCGTGCTTATGTAAACGCTCGCGTAATGTCTGCAATTCCGCTAAAAGGTGTGTGTGACGACTACAACTGGGCAGACGCTATCCGCGAACTTCGCCAAGGCCGTGTAGTGATCTTCTCTGCAGGTACAGGTAATCCATTCTTTACGACGGATTCAGCAGCTTGTCTACGTGGTATCGAAATCGAAGCTGACGTAGTACTAAAAGCGACAAAAGTTGATGGCGTATTTACTGCTGACCCAGTAGCAAACCCAGACGCAGAGCTGTACGATAAGCTTTCTTACACTGAAATTCTGGATAAGGAACTGAAAGTAATGGACTTG | MN253221 |
| LK04 | *Vibrio communis* | GGCGGTGGTAACTTGTTCCGTGGTGCAGGTCTTGCAGAAGCGGGTATGAACCGCGTAGTAGGCGACCACATGGGTATGCTTGCAACAGTAATGAACGGTCTAGCAATGCGTGATGCACTTCACCGTGCTTATGTAAACGCTCGCGTAATGTCTGCAATTCCGCTAAAAGGTGTGTGTGACGATTACAACTGGGCAGACGCTATACGCGAACTTCGCCAGGGCCGTGTAGTGATCTTCTCTGCAGGTACAGGTAACCCATTCTTTACGACGGATTCAGCAGCTTGTCTACGTGGTATCGAAATCGAAGCTGACGTAGTTCTAAAAGCGACAAAAGTAGATGGTGTATTTACTGCTGACCCAGTAGCAAACCCAGACGCAGAGCTGTATGATAAGCTATCTTACACAGAAATTCTGGATAAAGAATTGAAAGTAATGGACTTG | MN253222 |
| LL05 | *Vibrio communis* | GGCGGTGGTAACTTGTTCCGTGGTGCAGGTCTTGCAGAAGCGGGTATGAACCGAGTAGTAGGCGACCACATGGGTATGCTTGCAACAGTAATGAACGGTCTAGCAATGCGTGATGCACTTCACCGTGCTTATGTAAACGCTCGCGTAATGTCTGCAATTCCGCTAAAAGGTGTGTGTGACGATTATAATTGGGCAGACGCTATCCGCGAACTTCGCCAAGGCCGTGTAGTTATCTTCTCTGCAGGTACTGGTAACCCATTCTTCACTACAGATTCAGCAGCGTGTCTACGCGGCATCGAAATCGAAGCTGACGTAGTTCTAAAAGCAACGAAAGTTGATGGTGTATTTACTGCTGACCCAGTAGCCAACCCAGACGCAGAGCTGTATGATAAGCTATCTTACACTGAAATCCTGGATAAGGAATTAAAAGTAATGGACTTG | MN253223 |
| LS05 | *Vibrio communis* | GGCGGTGGTAACTTGTTCCGTGGTGCAGGTCTTGCAGAAGCGGGTATGAACCGCGTAGTAGGCGACCACATGGGTATGCTTGCAACAGTAATGAACGGTCTAGCAATGCGTGATGCACTTCACCGTGCTTATGTAAACGCTCGCGTAATGTCTGCAATTCCGCTAAAAGGTGTGTGTGACGATTACAATTGGGCAGACGCTATCCGCGAACTTCGCCAAGGCCGTGTAGTTATCTTCTCTGCAGGTACTGGTAACCCATTCTTCACTACAGATTCAGCAGCGTGTCTACGCGGCATCGAAATCGAAGCTGACGTAGTTCTAAAAGCAACGAAAGTTGATGGTGTATTTACTGCTGACCCAGTAGCAAACCCAGACGCAGAGCTGTATGATAAGCTATCTTACACTGAAATTCTGGATAAAGAATTGAAAGTAATGGACTTG | MN253224 |
| LS06 | *Vibrio communis* | GGCGGTGGTAACCTGTTCCGTGGTGCTGGTCTTGCAGAGGCTGGTATGAACCGCGTAGTGGGTGACCACATGGGTATGCTTGCAACAGTAATGAATGGTCTAGCGATGCGTGATGCACTTCACCGTGCTTACGTAAATGCTCGCGTGATGTCTGCAATTCCGCTTAAAGGTGTGTGTGACGACTACAATTGGGCAGACGCTATCCGCGAACTTCGCCAAGGCCGCGTGGTTATCTTCTCTGCAGGTACTGGTAACCCATTCTTCACTACTGATTCTGCGGCGTGTCTACGCGGCATCGAAATCGAAGCTGACGTAGTTCTAAAAGCAACGAAAGTTGATGGTGTATTTACTGCTGACCCAGTAGCCAACCCAGACGCAGAACTGTATGATAAGCTATCTTACACTGAAATTCTGGATAAAGAATTGAAAGTAATGGACTTG | MN253225 |
| LK06 | *Vibrio communis* | GGCGGTGGTAACTTGTTCCGTGGTGCTGGTCTTGCAGAGGCTGGTATGAACCGCGTAGTAGGTGACCACATGGGTATGCTTGCAACAGTAATGAACGGTCTAGCAATGCGTGATGCACTTCACCGTGCTTATGTAAATGCTCGCGTGATGTCTGCAATTCCGCTTAAAGGTGTGTGTGACGACTACAATTGGGCAGACGCTATCCGCGAACTTCGCCAAGGCCGTGTGGTTATCTTCTCTGCAGGTACTGGTAACCCATTCTTCACTACTGATTCTGCAGCTTGTCTACGCGGCATCGAAATCGAAGCTGACGTAGTTCTAAAAGCAACGAAAGTTGATGGTGTATTTACTGCTGATCCAGTAGCTAACCCAGACGCAGAACTGTATGATAAGCTATCTTACACTGAAATTCTGGATAAAGAATTGAAAGTAATGGACTTG | MN253226 |
| LL07 | *Vibrio communis* | GGCGGTGGTAACTTGTTCCGTGGTGCAGGTCTTGCAGAAGCTGGTATGAACCGCGTAGTAGGTGACCACATGGGTATGCTTGCAACAGTAATGAATGGTCTAGCAATGCGTGACGCACTTCACCGTGCTTACGTAAATGCTCGCGTGATGTCTGCAATTCCGCTTAAAGGTGTGTGTGACGACTACAATTGGGCAGACGCTATCCGCGAACTTCGCCAAGGCCGTGTGGTTATCTTCTCTGCAGGTACTGGTAACCCATTCTTCACTACAGACTCTGCGGCTTGTCTACGCGGCATCGAAATCGAAGCTGACGTAGTTCTAAAAGCAACGAAAGTTGATGGTGTATTTACTGCAGATCCAGTAGCAAACCCAGATGCAGAACTGTATGATAAGCTATCTTACACTGAAATCCTGGATAAAGAATTGAAAGTAATGGACTTG | MN253227 |
| LL08 | *Vibrio communis* | GGCGGTGGTAACTTGTTCCGTGGTGCAGGTCTTGCAGAAGCGGGTATGAACCGCGTAGTAGGCGACCACATGGGTATGCTTGCAACAGTAATGAACGGTCTAGCAATGCGTGATGCACTTCACCGTGCTTATGTAAACGCTCGCGTAATGTCTGCAATTCCGCTAAAAGGTGTGTGTGACGATTACAACTGGGCAGACGCTATCCGCGAACTTCGCCAAGGCCGTGTAGTGATCTTCTCTGCAGGTACAGGTAACCCATTCTTTACGACGGATTCAGCAGCTTGTCTACGTGGTATCGAAATCGAAGCTGACGTAGTTCTAAAAGCGACAAAAGTTGATGGTGTATTTACTGCTGACCCAGTAGCAAACCCAGACGCAGAGCTGTATGATAAGCTATCTTACACTGAAATTCTGGATAAAGAATTGAAAGTAATGGACTTG | MN253228 |
| LL09 | *Vibrio communis* | GGCGGTGGTAACCTGTTCCGTGGTGCTGGTCTTGCAGAAGCTGGTATGAACCGCGTAGTAGGCGACCACATGGGTATGCTTGCAACAGTAATGAACGGTCTAGCAATGCGTGACGCACTTCACCGTGCTTACGTAAACGCTCGCGTAATGTCTGCAATTCCGCTAAAAGGTGTGTGTGACGACTACAATTGGGCAGACGCTATCCGCGAACTTCGCCAAGGCCGTGTGGTTATCTTCTCTGCAGGTACTGGTAACCCATTCTTCACGACAGATTCTGCGGCGTGTCTACGCGGCATCGAAATCGAAGCTGACGTAGTTCTAAAAGCAACGAAAGTTGATGGTGTATTTACTGCTGACCCAGTAGCTAACCCAGACGCAGAACTGTATGATAAGCTATCTTACACTGAAATTCTGGATAAAGAATTGAAAGTAATGGACTTG | MN253229 |
| LK09 | *Vibrio communis* | GGCGGTGGTAACCTGTTCCGTGGTGCAGGTCTTGCAGAAGCTGGTATGAACCGCGTAGTAGGCGACCACATGGGTATGCTTGCAACAGTAATGAACGGTCTAGCAATGCGTGACGCACTTCACCGTGCTTATGTAAACGCTCGCGTGATGTCTGCAATTCCGCTTAAAGGTGTGTGTGACGATTACAATTGGGCAGACGCTATCCGCGAACTTCGCCAAGGCCGTGTGGTTATCTTCTCTGCAGGTACTGGTAACCCATTCTTCACTACAGATTCTGCAGCGTGTCTACGCGGCATCGAAATCGAAGCTGACGTAGTTCTAAAAGCAACGAAAGTTGATGGTGTATTTACTGCTGACCCAGTAGCAAACCCAGACGCAGAACTGTATGATAAGCTATCTTACACTGAAATTCTGGATAAAGAATTGAAAGTAATGGACTTG | MN253230 |
| LL10 | *Vibrio communis* | GGCGGTGGTAACCTGTTCCGTGGTGCAGGTCTTGCAGAGGCTGGTATGAACCGCGTAGTGGGTGACCACATGGGTATGCTTGCAACAGTAATGAATGGTCTAGCAATGCGTGACGCACTTCACCGTGCTTACGTAAACGCTCGCGTGATGTCTGCAATTCCGCTAAAAGGTGTGTGTGACGACTACAATTGGGCAGACGCTATCCGCGAACTTCGCCAAGGCCGTGTAGTTATCTTCTCTGCAGGTACTGGTAACCCATTCTTCACGACAGATTCAGCAGCGTGTCTACGCGGCATCGAAATCGAAGCTGACGTAGTTCTAAAAGCAACGAAAGTTGATGGTGTATTTACTGCTGACCCAGTAGCAAACCCAGACGCAGAGCTGTATGATAAGCTATCTTACACTGAAATTCTGGATAAAGAATTGAAAGTAATGGACTTG | MN253231 |
| LS10 | *Vibrio communis* | GGCGGTGGTAACCTGTTCCGTGGTGCTGGTCTTGCAGAAGCTGGTATGAACCGCGTAGTAGGTGACCACATGGGTATGCTTGCAACAGTAATGAATGGTCTAGCAATGCGTGACGCACTTCACCGTGCTTACGTAAATGCTCGCGTGATGTCTGCAATTCCGCTTAAAGGTGTGTGTGACGACTACAATTGGGCAGACGCTATCCGCGAACTTCGCCAAGGCCGTGTGGTTATCTTCTCTGCAGGTACTGGTAACCCATTCTTCACGACAGATTCTGCCGCCTGTCTACGCGGCATCGAAATCGAAGCTGACGTAGTTCTAAAAGCAACGAAAGTTGATGGTGTATTTACTGCTGACCCAGTAGCAAACCCAGACGCAGAACTGTATGATAAGCTATCTTACACTGAAATTCTGGATAAAGAATTGAAAGTAATGGACTTG | MN253232 |
| LL11 | *Vibrio communis* | GGCGGTGGTAACCTGTTCCGTGGTGCTGGTCTTGCAGAAGCTGGTATGAACCGCGTAGTGGGTGACCACATGGGTATGCTTGCAACAGTAATGAATGGTCTAGCAATGCGTGATGCACTTCACCGTGCTTACGTAAATGCTCGCGTGATGTCTGCAATTCCGCTTAAAGGTGTGTGTGACGACTACAATTGGGCAGACGCTATCCGCGAACTTCGCCAAGGTCGTGTGGTTATCTTCTCTGCAGGTACTGGTAACCCATTCTTCACTACTGATTCTGCGGCTTGTCTACGTGGTATCGAAATTGAAGCTGACGTAGTTCTAAAAGCTACCAAAGTTGACGGGGTATTTACTGCTGACCCAGTAGCAAACCCCGATGCAGAACTGTATGATAAGCTATCTTACACTGAAATTCTGGATAAAGAATTGAAAGTAATGGACTTG | MN253233 |
| LS12 | *Vibrio communis* | GGCGGTGGTAACCTGTTCCGTGGTGCAGGTCTTGCAGAAGCTGGTATGAACCGCGTAGTAGGTGACCACATGGGTATGCTTGCAACAGTAATGAATGGTCTAGCAATGCGTGACGCACTTCACCGTGCTTACGTAAATGCTCGCGTGATGTCTGCAATTCCGCTTAAAGGTGTGTGTGACGACTACAATTGGGCAGACGCTATCCGCGAACTTCGCCAAGGCCGTGTGGTTATCTTCTCTGCAGGTACTGGTAACCCATTCTTCACTACAGATTCTGCAGCATGTCTACGTGGTATCGAAATTGAAGCTGACGTAGTTCTAAAAGCAACCAAAGTTGACGGGGTATTTACTGCTGACCCAGTAGCAAACCCAGACGCAGAACTGTATGATAAGCTATCTTACACTGAAATTCTGGATAAAGAATTGAAAGTAATGGACTTG | MN253234 |
| LL13 | *Vibrio communis* | GGCGGTGGTAACTTGTTCCGTGGTGCAGGTCTTGCAGAAGCGGGTATGAACCGCGTAGTAGGCGACCACATGGGTATGCTTGCAACAGTAATGAACGGTCTAGCAATGCGTGATGCACTTCACCGTGCTTATGTAAACGCTCGCGTAATGTCTGCAATTCCGCTAAAAGGTGTGTGTGACGATTACAACTGGGCAGACGCTATCCGCGAACTTCGCCAAGGCCGTGTAGTGATCTTCTCTGCAGGTACAGGTAACCCATTCTTTACGACGGATTCAGCAGCTTGTCTACGTGGTATCGAAATCGAAGCTGACGTAGTTCTAAAAGCGACAAAAGTTGATGGTGTATTTACTGCTGACCCAGTAGCAAACCCAGACGCAGAGCTGTATGATAAGCTATCTTACACAGAAATTCTGGATAAAGAATTGAAAGTAATGGACTTG | MN253235 |
| LS13 | *Vibrio communis* | GGCGGTGGTAACTTGTTCCGTGGTGCTGGTCTTGCAGAAGCTGGTATGAACCGCGTAGTAGGCGACCACATGGGTATGCTTGCAACAGTAATGAACGGTCTAGCAATGCGTGACGCACTTCACCGTGCTTATGTAAACGCTCGCGTGATGTCTGCAATTCCGCTAAAAGGTGTGTGTGACGATTACAATTGGGCAGACGCTATCCGCGAACTTCGCCAAGGCCGTGTAGTTATCTTCTCTGCAGGTACTGGTAACCCATTCTTCACGACAGATTCTGCAGCGTGTCTACGCGGCATCGAAATCGAAGCTGACGTAGTTCTAAAAGCAACGAAAGTTGATGGTGTATTTACTGCTGACCCAGTAGCAAACCCAGACGCAGAGCTGTATGATAAGCTATCTTACACAGAAATTCTGGATAAAGAATTGAAAGTAATGGACTTG | MN253236 |
| LK13 | *Vibrio communis* | GGCGGTGGTAACTTGTTCCGTGGTGCAGGTCTTGCAGAAGCGGGTATGAACCGCGTAGTAGGCGACCACATGGGTATGCTTGCAACAGTAATGAACGGTCTAGCAATGCGTGATGCACTTCACCGTGCTTATGTAAACGCTCGCGTAATGTCTGCAATTCCGCTAAAAGGTGTGTGTGACGATTACAACTGGGCAGACGCTATCCGCGAACTTCGCCAAGGCCGTGTAGTGATCTTCTCTGCAGGTACAGGTAACCCATTCTTTACGACGGATTCAGCAGCTTGTCTACGTGGTATCGAAATCGAAGCTGACGTAGTTCTAAAAGCGACAAAAGTTGATGGTGTATTTACTGCTGACCCAGTAGCAAACCCAGACGCAGAGCTGTATGATAAGCTATCTTACACAGAAATTCTGGATAAAGAATTGAAAGTAATGGACTTG | MN253237 |
| LS14 | *Vibrio communis* | GGCGGTGGTAACTTGTTCCGTGGTGCAGGTCTTGCAGAAGCGGGTATGAACCGCGTAGTAGGCGACCACATGGGTATGCTTGCAACAGTAATGAACGGTCTAGCAATGCGTGATGCACTTCACCGTGCTTATGTAAACGCTCGCGTAATGTCTGCAATTCCGCTAAAAGGTGTGTGTGACGATTACAACTGGGCAGACGCTATCCGCGAACTTCGCCAAGGCCGTGTAGTGATCTTCTCTGCAGGTACAGGTAACCCATTCTTTACGACGGATTCAGCAGCTTGTCTACGTGGTATCGAAATCGAAGCTGACGTAGTTCTAAAAGCGACAAAAGTTGATGGTGTATTTACTGCTGACCCAGTAGCAAACCCAGACGCAGAGCTGTATGATAAGCTATCTTACACTGAAATTCTGGATAAAGAATTGAAAGTAATGGACTTG | MN253238 |
| LL15 | *Vibrio communis* | GGCGGTGGTAACTTGTTCCGTGGTGCAGGTCTTGCAGAAGCGGGTATGAACCGCGTAGTAGGCGACCACATGGGTATGCTTGCAACAGTAATGAACGGTCTAGCAATGCGTGATGCACTTCACCGTGCTTATGTAAACGCTCGCGTAATGTCTGCAATTCCGCTAAAAGGTGTGTGTGACGATTACAACTGGGCAGACGCTATCCGCGAACTTCGCCAAGGCCGTGTAGTGATCTTCTCTGCAGGTACAGGTAACCCATTCTTTACGACGGATTCAGCAGCTTGTCTACGTGGTATCGAAATCGAAGCTGACGTAGTTCTAAAAGCGACAAAAGTTGATGGTGTATTTACTGCTGACCCAGTAGCAAACCCAGACGCAGAGCTGTATGATAAGCTATCTTACACTGAAATTCTGGATAAAGAATTGAAAGTAATGGACTTG | MN253239 |
| LK15 | *Vibrio communis* | GGCGGTGGTAACTTGTTCCGTGGTGCAGGTCTTGCAGAAGCGGGTATGAACCGCGTAGTAGGCGACCACATGGGTATGCTTGCAACAGTAATGAACGGTCTAGCAATGCGTGATGCACTTCACCGTGCTTATGTAAACGCTCGCGTAATGTCTGCAATTCCGCTAAAAGGTGTGTGTGACGATTACAACTGGGCAGACGCTATCCGCGAACTTCGCCAAGGCCGTGTAGTGATCTTCTCTGCAGGTACAGGTAACCCATTCTTTACGACGGATTCAGCAGCTTGTCTACGTGGTATCGAAATCGAAGCTGACGTAGTTCTAAAAGCGACAAAAGTTGATGGTGTATTTACTGCTGACCCAGTAGCAAACCCAGACGCAGAGCTGTATGATAAGCTATCTTACACTGAAATTCTGGATAAAGAATTGAAAGTAATGGACTTG | MN253240 |
| LK16 | *Vibrio communis* | GGCGGTGGTAACTTGTTCCGTGGTGCAGGTCTTGCAGAAGCGGGTATGAACCGCGTAGTAGGCGACCACATGGGTATGCTTGCAACAGTAATGAACGGTCTAGCAATGCGTGATGCACTTCACCGTGCTTATGTAAACGCTCGCGTAATGTCTGCAATTCCGCTAAAAGGTGTGTGTGACGATTACAACTGGGCAGACGCTATCCGCGAACTTCGCCAAGGCCGTGTCGTGATCTTCTCTGCAGGTACAGGTAACCCATTCTTTACGACGGATTCAGCAGCTTGTCTACGTGGTATCGAAATCGAAGCTGACGTAGTTCTAAAAGCGACAAAAGTTGATGGTGTATTTACTGCTGACCCAGTAGCAAACCCAGACGCAGAGCTGTATGATAAGCTATCTTACACTGAAATTCTGGATAAAGAATTGAAAGTAATGGACTTG | MN253241 |
| LL17 | *Vibrio communis* | GGGGGTGGTAACCTGTTCCGTGGTGCTGGTCTTGCAGAGGCTGGTATGAACCGCGTAGTAGGCGACCACATGGGTATGCTTGCAACAGTAATGAACGGTCTAGCAATGCGTGATGCACTTCACCGTGCTTATGTAAACGCTCGCGTGATGTCTGCAATTCCGCTAAAAGGTGTGTGTGACGACTACAATTGGGCAGACGCTATCCGCGAACTTCGCCAAGGCCGTGTGGTTATCTTCTCTGCAGGTACTGGTAACCCATTCTTCACTACTGATTCTGCAGCTTGTCTACGCGGCATCGAAATCGAAGCTGACGTAGTTCTAAAAGCAACCAAAGTTGATGGTGTATTTACTGCTGACCCAGTAGCAAACCCAGACGCAGAGCTGTATGATAAGCTATCTTACACTGAAATTCTGGATAAAGAATTGAAAGTAATGGACTTG | MN253242 |
| LS17 | *Vibrio communis* | GGCGGTGGTAACTTGTTCCGTGGTGCAGGTCTTGCAGAAGCGGGTATGAACCGCGTAGTAGGCGACCACATGGGTATGCTTGCAACAGTAATGAACGGTCTAGCAATGCGTGATGCACTTCACCGTGCTTATGTAAACGCTCGCGTAATGTCTGCAATTCCGCTAAAAGGTGTGTGTGACGATTACAACTGGGCAGACGCTATCCGCGAACTTCGCCAAGGCCGTGTCGTGATCTTCTCTGCAGGTACAGGTAACCCATTCTTTACGACGGATTCAGCAGCTTGTCTACGTGGTATCGAAATCGAAGCTGACGTAGTTCTAAAAGCGACAAAAGTTGATGGTGTATTTACTGCTGACCCAGTAGCAAACCCAGACGCAGAGCTGTATGATAAGCTATCTTACACTGAAATTCTGGATAAAGAATTGAAAGTAATGGACTTG | MN253243 |
| LL19 | *Vibrio communis* | GGCGGTGGTAACTTGTTCCGTGGTGCAGGTCTTGCAGAAGCGGGTATGAACCGCGTAGTAGGCGACCACATGGGTATGCTTGCAACAGTAATGAACGGTCTAGCAATGCGTGATGCACTTCACCGTGCTTATGTAAACGCTCGCGTAATGTCTGCAATTCCGCTAAAAGGTGTGTGTGACGATTACAACTGGGCAGACGCTATCCGCGAACTTCGCCAAGGCCGTGTAGTGATCTTCTCTGCAGGTACAGGTAACCCATTCTTTACGACGGATTCAGCAGCTTGTCTACGTGGTATCGAAATCGAAGCTGACGTAGTTCTAAAAGCGACAAAAGTTGATGGTGTATTTACTGCTGACCCAGTAGCAAACCCAGACGCAGAGCTGTATGATAAGCTATCTTACACAGAAATTCTGGATAAAGAATTGAAAGTAATGGACTTG | MN253244 |
| LS19 | *Vibrio communis* | GGCGGTGGTAACTTGTTCCGTGGTGCAGGTCTTGCAGAAGCGGGTATGAACCGCGTAGTAGGCGACCACATGGGTATGCTTGCAACAGTAATGAACGGTCTAGCAATGCGTGATGCACTTCACCGTGCTTATGTAAACGCTCGCGTAATGTCTGCAATTCCGCTAAAAGGTGTGTGTGACGATTACAACTGGGCAGACGCTATCCGCGAACTTCGCCAAGGCCGTGTAGTGATCTTCTCTGCAGGTACAGGTAACCCATTCTTTACGACGGATTCAGCAGCTTGTCTACGTGGTATCGAAATCGAAGCTGACGTAGTTCTAAAAGCGACAAAAGTTGATGGTGTATTTACTGCTGACCCAGTAGCAAACCCAGACGCAGAGCTGTATGATAAGCTATCTTACACAGAAATTCTGGATAAAGAATTGAAAGTAATGGACTTG | MN253245 |
| LS20 | *Vibrio communis* | GGCGGTGGTAACTTGTTCCGTGGTGCAGGTCTTGCAGAAGCGGGTATGAACCGCGTAGTAGGCGACCACATGGGTATGCTTGCAACAGTAATGAACGGTCTAGCAATGCGTGATGCACTTCACCGTGCTTATGTAAACGCTCGCGTAATGTCTGCAATTCCGCTAAAAGGTGTGTGTGACGATTACAACTGGGCAGACGCTATCCGCGAACTTCGCCAAGGCCGTGTAGTGATCTTCTCTGCAGGTACAGGTAACCCATTCTTTACGACGGATTCAGCAGCTTGTCTACGTGGTATCGAAATCGAAGCTGACGTAGTTCTAAAAGCGACAAAAGTTGATGGTGTATTTACTGCTGACCCAGTAGCAAACCCAGACGCAGAGCTGTATGATAAGCTATCTTACACTGAAATTCTGGATAAAGAATTGAAAGTAATGGACTTG | MN253246 |
| LS21 | *Vibrio communis* | GGCGGTGGTAACTTGTTCCGTGGTGCAGGTCTTGCAGAAGCGGGTATGAACCGCGTAGTAGGCGACCACATGGGTATGCTTGCAACAGTAATGAACGGTCTAGCAATGCGTGATGCACTTCACCGTGCTTATGTAAACGCTCGCGTAATGTCTGCAATTCCGCTAAAAGGTGTGTGTGACGATTACAACTGGGCAGACGCTATCCGCGAACTTCGCCAAGGCCGTGTCGTGATCTTCTCTGCAGGTACAGGTAACCCATTCTTTACGACGGATTCAGCAGCTTGTCTACGTGGTATCGAAATCGAAGCTGACGTAGTTCTAAAAGCGACAAAAGTTGATGGTGTATTTACTGCTGACCCAGTAGCAAACCCAGACGCAGAGCTGTATGATAAGCTATCTTACACTGAAATTCTGGATAAAGAATTGAAAGTAATGGACTTG | MN253247 |
| LK21 | *Vibrio communis* | GGCGGTGGTAACTTGTTCCGTGGTGCAGGTCTTGCAGAAGCTGGTATGAACCGCGTAGTAGGTGACCACATGGGTATGCTTGCAACAGTAATGAATGGTCTAGCAATGCGTGACGCACTTCACCGTGCTTACGTAAATGCTCGCGTGATGTCTGCAATTCCGCTTAAAGGTGTGTGTGACGACTACAATTGGGCAGACGCTATCCGCGAACTTCGCCAAGGCCGTGTGGTTATCTTCTCTGCAGGTACTGGTAACCCATTCTTTACGACGGATTCAGCAGCTTGTCTACGTGGTATCGAAATCGAAGCTGACGTAGTTCTAAAAGCGACAAAAGTTGATGGTGTATTTACTGCTGACCCAGTAGCAAACCCAGACGCAGAGCTGTATGATAAGCTATCTTACACTGAAATTCTGGATAAAGAATTGAAAGTAATGGACTTG | MN253248 |
| LS22 | *Vibrio communis* | GGCGGTGGTAACTTGTTCCGTGGTGCTGGTCTTGCAGAAGCTGGTATGAACCGCGTAGTAGGTGACCACATGGGTATGCTTGCAACAGTAATGAACGGTCTAGCAATGCGTGATGCACTTCACCGTGCTTACGTAAACGCTCGCGTGATGTCTGCAATTCCGCTAAAAGGTGTGTGTGACGACTACAATTGGGCAGACGCTATCCGCGAACTTCGCCAAGGCCGTGTGGTTATCTTCTCTGCAGGTACTGGTAACCCATTCTTCACGACAGATTCTGCGGCGTGTCTACGCGGCATCGAAATCGAAGCTGACGTAGTTCTAAAAGCAACAAAAGTTGATGGTGTATTTACTGCTGACCCAGTAGCAAACCCAGACGCAGAACTGTATGATAAGCTATCTTACACTGAAATTCTGGATAAAGAATTGAAAGTAATGGACTTG | MN253249 |
| LK23 | *Vibrio communis* | GGCGGTGGTAACTTGTTCCGTGGTGCAGGTCTTGCAGAAGCGGGTATGAACCGCGTAGTAGGCGACCACATGGGTATGCTTGCAACAGTAATGAACGGTCTAGCAATGCGTGATGCACTTCACCGTGCTTATGTAAACGCTCGCGTAATGTCTGCAATTCCGCTAAAAGGTGTGTGTGACGATTACAACTGGGCAGACGCTATCCGCGAACTTCGCCAAGGCCGTGTAGTGATCTTCTCTGCAGGTACAGGTAACCCATTCTTTACGACGGATTCAGCAGCTTGTCTACGTGGTATCGAAATCGAAGCTGACGTAGTTCTAAAAGCGACAAAAGTTGATGGTGTATTTACTGCTGACCCAGTAGCAAACCCAGACGCAGAGCTGTATGATAAGCTATCTTACACTGAAATTCTGGATAAAGAATTGAAAGTAATGGACTTG | MN253250 |
| LL24 | *Vibrio communis* | GGCGGTGGTAACTTGTTCCGTGGTGCAGGTCTTGCAGAAGCGGGTATGAACCGCGTAGTAGGCGACCACATGGGTATGCTTGCAACAGTAATGAACGGTCTAGCAATGCGTGATGCACTTCACCGTGCTTATGTAAACGCTCGCGTAATGTCTGCAATTCCGCTAAAAGGTGTGTGTGACGATTACAACTGGGCAGACGCTATCCGCGAACTTCGCCAAGGCCGTGTAGTGATCTTCTCTGCAGGTACAGGTAACCCATTCTTTACGACGGATTCAGCAGCTTGTCTACGTGGTATCGAAATCGAAGCTGACGTAGTTCTAAAAGCGACAAAAGTTGATGGTGTATTTACTGCTGACCCAGTAGCAAACCCAGACGCAGAGCTGTATGATAAGCTATCTTACACAGAAATTCTGGATAAAGAATTGAAAGTAATGGACTTG | MN253251 |
| LS25 | *Vibrio communis* | GGCGGTGGTAACTTGTTCCGTGGTGCAGGTCTTGCAGAAGCGGGTATGAACCGCGTAGTAGGCGACCACATGGGTATGCTTGCAACAGTAATGAACGGTCTAGCAATGCGTGATGCACTTCACCGTGCTTATGTAAACGCTCGCGTAATGTCTGCAATTCCGCTAAAAGGTGTGTGTGACGATTACAACTGGGCAGACGCTATCCGCGAACTTCGCCAAGGCCGTGTAGTGATCTTCTCTGCAGGTACAGGTAACCCATTCTTTACGACGGATTCAGCAGCTTGTCTACGTGGTATCGAAATCGAAGCTGACGTAGTTCTAAAAGCGACAAAAGTTGATGGTGTATTTACTGCTGACCCAGTAGCAAACCCAGACGCAGAGCTGTATGATAAGCTATCTTACACTGAAATTCTGGATAAAGAATTGAAAGTAATGGACTTG | MN253252 |
| LK27 | *Vibrio communis* | GGCGGTGGTAACTTGTTCCGTGGTGCAGGTCTTGCAGAAGCAGGTATGAACCGCGTAGTAGGCGACCACATGGGTATGCTTGCAACAGTAATGAACGGTCTAGCAATGCGTGACGCACTTCACCGTGCTTATGTAAACGCTCGCGTAATGTCTGCAATTCCGCTAAAAGGTGTGTGTGACGATTACAATTGGGCAGACGCTATCCGCGAACTTCGCCAAGGCCGTGTAGTTATCTTCTCTGCAGGTACCGGTAACCCATTCTTTACGACGGATTCAGCAGCTTGTCTACGTGGTATCGAAATCGAAGCTGACGTAGTTCTAAAAGCGACAAAAGTTGATGGTGTATTTACTGCTGACCCAGTAGCAAACCCAGACGCAGAGCTGTATGATAAGCTATCTTACACTGAAATTCTGGATAAAGAATTGAAAGTAATGGACTTG | MN253253 |
| LL28 | *Vibrio communis* | GGCGGTGGTAACTTGTTCCGTGGTGCAGGTCTTGCAGAAGCGGGTATGAACCGCGTAGTAGGCGACCACATGGGTATGCTTGCAACAGTAATGAACGGTCTAGCAATGCGTGATGCACTTCACCGTGCTTATGTAAACGCTCGCGTAATGTCTGCAATTCCGCTAAAAGGTGTGTGTGACGATTACAACTGGGCAGACGCTATCCGCGAACTTCGCCAAGGCCGTGTAGTGATCTTCTCTGCAGGTACAGGTAACCCATTCTTTACGACGGATTCAGCAGCTTGTCTACGTGGTATCGAAATCGAAGCTGACGTAGTTCTAAAAGCGACAAAAGTTGATGGTGTATTTACTGCTGACCCAGTAGCAAACCCAGACGCAGAGCTGTATGATAAGCTATCTTACACTGAAATTCTGGATAAAGAATTGAAAGTAATGGACTTG | MN253254 |
| LL29 | *Vibrio communis* | GGCGGTGGTAACTTGTTCCGTGGTGCAGGTCTTGCAGAAGCGGGTATGAACCGCGTAGTAGGCGACCACATGGGTATGCTTGCAACAGTAATGAACGGTCTAGCAATGCGTGATGCACTTCACCGTGCTTATGTAAACGCTCGCGTAATGTCTGCAATTCCGCTAAAAGGTGTGTGTGACGATTACAACTGGGCAGACGCTATCCGCGAACTTCGCCAAGGCCGTGTAGTGATCTTTTCTGCAGGTACAGGTAACCCATTCTTTACGACGGATTCAGCAGCTTGTCTACGTGGGATCGAAATCGAAGCTGACGTTGTTCTAAAAGCGACAAAAGTTGATGGTGTATTTACTGCTGACCCAGTAGCAAACCCAGACGCAGAGCTGTATGATAAGCTATCTTACACTGAAATTCTGGATAAAGAATTGAAAGTAATGGACTTG | MN253255 |
| LS29 | *Vibrio communis* | GGCGGTGGTAACTTGTTCCGTGGTGCAGGTCTTGCAGAAGCGGGTATGAACCGCGTAGTAGGCGACCACATGGGTATGCTTGCAACAGTAATGAACGGTCTAGCAATGCGTGATGCACTTCACCGTGCTTATGTAAACGCTCGCGTAATGTCTGCAATTCCGCTAAAAGGTGTGTGTGACGATTACAACTGGGCAGACGCTATCCGCGAACTTCGCCAAGGCCGTGTAGTGATCTTTTCTGCAGGTACAGGTAACCCATTCTTTACGACGGATTCAGCAGCTTGTCTACGTGGGATTGAAATCGAAGCTGACGTTGTTCTAAAAGCGACAAAAGTTGATGGTGTATTTACTGCTGACCCAGTTGCAAACCCAGACGCAGAGCTGTATGATAAGCTATCTTACACTGAAATTCTGGATAAAGAATTGAAAGTAATGGACTTG | MN253256 |
| LK29 | *Vibrio communis* | GGCGGTGGTAACTTGTTCCGTGGTGCAGGTCTTGCAGAAGCGGGTATGAACCGCGTAGTAGGCGACCACATGGGTATGCTTGCAACAGTAATGAACGGTCTAGCAATGCGTGATGCACTTCACCGTGCTTATGTAAACGCTCGCGTAATGTCTGCAATTCCGCTAAAAGGTGTGTGTGACGATTACAACTGGGCAGACGCTATCCGCGAACTTCGCCAAGGCCGTGTAGTGATCTTCTCTGCAGGTACAGGTAACCCATTCTTTACGACGGATTCAGCAGCTTGTCTACGTGGTATCGAAATCGAAGCTGACGTAGTTCTAAAAGCGACAAAAGTTGATGGTGTATTTACTGCTGACCCAGTAGCAAACCCAGACGCAGAGCTGTATGATAAGCTATCTTACACTGAAATTCTGGATAAAGAATTGAAAGTAATGGACTTG | MN253257 |
| LS30 | *Vibrio communis* | GGCGGTGGTAACTTGTTCCGTGGTGCAGGTCTTGCAGAAGCGGGTATGAACCGCGTAGTAGGCGACCACATGGGTATGCTTGCAACAGTAATGAACGGTCTAGCAATGCGTGATGCACTTCACCGTGCTTATGTAAACGCTCGCGTAATGTCTGCAATTCCGCTAAAAGGTGTGTGTGACGATTACAACTGGGCAGACGCTATCCGCGAACTTCGCCAAGGCCGTGTAGTGATCTTCTCTGCAGGTACAGGTAACCCATTCTTTACGACGGATTCAGCAGCTTGTCTACGTGGTATCGAAATCGAAGCCGACGTGGTCCTTAAAGCAACAAAAGTTGATGGTGTATTTACTGCTGACCCAGTAGCAAACCCAGACGCAGAGCTGTATGACAAGCTATCTTACACTGAGATTCTGGACAAAGAATTGAAAGTAATGGACTTG | MN253258 |
| PJS01 | *Vibrio communis* | GGCGGTGGTAACTTGTTCCGTGGTGCAGGTCTTGCAGAAGCTGGTATGAACCGCGTAGTGGGTGACCACATGGGTATGCTTGCAACAGTAATGAACGGTCTAGCAATGCGTGATGCACTTCACCGTGCTTATGTAAACGCTCGCGTAATGTCTGCAATTCCGCTTAAAGGTGTGTGTGACGACTACAATTGGGCAGACGCTATCCGCGAACTTCGCCAAGGCCGTGTGGTTATCTTCTCTGCAGGTACTGGTAACCCATTCTTCACTACTGATTCTGCGGCTTGTCTACGCGGCATTGAAATTGAAGCTGACGTCGTTCTGAAGGCTACCAAAGTTGATGGTGTATTTACTGCTGACCCAGTAGCAAACCCAGACGCAGAGCTGTATGATAAGCTATCTTACACTGAAATTCTGGATAAAGAATTGAAAGTAATGGACTTG | MN253259 |
| PJL02 | *Vibrio communis* | GGCGGTGGTAACCTGTTCCGTGGTGCTGGTCTTGCAGAAGCTGGTATGAACCGCGTAGTGGGTGACCACATGGGTATGCTTGCAACAGTAATGAATGGTCTAGCAATGCGTGATGCACTTCACCGTGCTTATGTAAATGCTCGCGTGATGTCTGCAATTCCGCTTAAAGGTGTGTGTGACGATTACAATTGGGCAGACGCTATCCGCGAACTTCGCCAAGGCCGTGTGGTTATCTTCTCTGCAGGTACTGGTAACCCATTCTTCACTACTGATTCTGCGGCTTGTCTACGCGGCATTGAAATTGAAGCTGATGTCGTTCTGAAGGCTACCAAAGTTGATGGTGTATTTACTGCTGACCCAGTAGCAAACCCAGACGCAGAGCTGTATGATAAGCTATCTTACACTGAAATTCTGGATAAAGAATTGAAAGTAATGGACTTG | MN253260 |
| PJS03 | *Vibrio communis* | GGGGGTGGTAACCTGTTCCGTGGTGCAGGTCTAGCAGAAGCTGGTATGAACCGCGTAGTGGGTGACCACATGGGTATGCTTGCAACAGTAATGAATGGTCTAGCAATGCGTGATGCACTTCACCGTGCTTATGTAAATGCTCGCGTGATGTCTGCAATTCCGCTTAAAGGTGTGTGTGATGACTACAATTGGGCAGACGCTATCCGCGAACTTCGCCAAGGCCGTGTGGTTATCTTCTCTGCAGGTACTGGTAACCCATTCTTCACTACTGATTCTGCGGCTTGTCTACGCGGCATTGAAATTGAAGCTGATGTCGTTCTGAAGGCTACCAAAGTTGATGGTGTATTTACTGCTGACCCAGTAGCAAACCCAGACGCAGAGCTGTATGATAAGCTATCTTACACTGAAATTCTGGATAAAGAATTGAAAGTAATGGACTTG | MN253261 |
| PJK04 | *Vibrio communis* | GGGGGTGGTAACTTGTTCCGTGGTGCAGGTCTTGCAGAAGCTGGTATGAACCGCGTAGTGGGTGACCACATGGGTATGCTTGCAACAGTAATGAATGGTCTAGCAATGCGTGATGCACTTCACCGTGCTTATGTAAATGCTCGCGTGATGTCTGCAATTCCGCTTAAAGGTGTGTGTGATGACTACAATTGGGCAGACGCTATCCGCGAACTTCGCCAAGGCCGTGTGGTTATCTTCTCTGCAGGTACTGGTAACCCATTCTTCACTACTGATTCTGCGGCTTGTCTACGCGGCATTGAAATTGAAGCTGATGTCGTCCTAAAAGCCACCAAAGTTGATGGTGTATTTACTGCTGACCCAGTAGCAAACCCAGACGCAGAGCTGTATGATAAGCTATCTTACACTGAAATTCTGGATAAAGAATTGAAAGTAATGGACTTG | MN253262 |
| PJS05 | *Vibrio communis* | GGGGGGGGGAACCTGTTCCGTGGTGCTGGTCTTGCAGAAGCTGGTATGAACCGCGTAGTGGGTGATCACATGGGTATGCTTGCAACAGTAATGAACGGTCTAGCAATGCGTGATGCACTTCACCGTGCTTATGTAAACGCTCGCGTAATGTCTGCAATTCCGCTTAAAGGTGTGTGTGACGACTACAATTGGGCAGACGCTATCCGCGAACTTCGCCAAGGCCGTGTGGTTATCTTCTCTGCAGGTACTGGTAACCCATTCTTCACTACTGATTCTGCGGCTTGTCTACGCGGCATTGAAATTGAAGCTGACGTAGTTCTCAAAGCTACCAAGGTTGATGGTGTATTTACTGCTGACCCAGTAGCAAACCCAGACGCAGAGCTGTATGATAAGCTATCTTACACTGAAATTCTGGATAAAGAATTGAAAGTAATGGACTTG | MN253263 |
| PJS06 | *Vibrio communis* | GGCGGTGGTAACCTGTTCCGTGGTGCAGGTCTTGCAGAAGCTGGTATGAACCGCGTAGTGGGTGACCACATGGGTATGCTTGCAACAGTAATGAACGGTCTAGCAATGCGTGATGCACTTCACCGTGCTTATGTAAATGCTCGCGTGATGTCTGCAATTCCGCTTAAAGGTGTGTGTGACGATTACAATTGGGCAGACGCTATCCGCGAACTTCGCCAAGGCCGTGTGGTTATCTTCTCTGCAGGTACTGGTAACCCATTCTTCACTACTGATTCTGCGGCGTGTCTGCGCGGCATCGAAATAGAGGCAGATGTAGTTCTAAAAGCAACCAAAGTCGATGGTGTATTTACTGCTGACCCAGTAGCAAACCCAGACGCAGAGCTGTATGATAAGCTATCTTACACTGAAATTCTGGATAAAGAATTGAAAGTAATGGACTTG | MN253264 |
| PJK06 | *Vibrio communis* | GGCGGTGGTAACCTGTTCCGTGGTGCAGGTCTTGCAGAAGCTGGTATGAACCGCGTAGTGGGTGACCACATGGGTATGCTTGCAACAGTTATGAACGGTCTAGCAATGCGTGATGCACTTCACCGTGCTTATGTGAATGCTCGCGTAATGAGCGCTATTCCGCTAAAAGGTGTGTGTGACGATTACAATTGGGCAGACGCTATCCGCGAACTTCGCCAAGGCCGTGTGGTTATCTTCTCTGCAGGTACTGGAAACCCATTCTTCACTACTGATTCTGCGGCTTGTCTACGCGGCATTGAAATTGAAGCTGACGTAGTTCTCAAAGCTACCAAGGTTGATGGTGTATTTACTGCTGACCCAGTAGCAAACCCAGACGCAGAGCTGTATGATAAGCTATCTTACACTGAAATTCTGGATAAAGAATTGAAAGTAATGGACTTG | MN253265 |
| PJS08 | *Vibrio communis* | GGGGGTGGTAACCTGTTCCGTGGTGCAGGTCTAGCAGAAGCTGGTATGAACCGCGTAGTGGGTGACCACATGGGTATGCTTGCAACAGTAATGAATGGTTTAGCAATGCGTGATGCACTTCACCGTGCTTATGTAAATGCTCGCGTTATGTCTGCAATTCCGCTTAAAGGTGTGTGTGACGATTACAATTGGGCAGACGCTATCCGCGAACTTCGCCAAGGCCGTGTGGTTATCTTCTCTGCAGGTACTGGTAACCCATTCTTCACTACTGATTCTGCGGCTTGTCTACGCGGCATTGAAATTGAAGCTGATGTCGTCCTAAAAGCCACCAAAGTTGATGGTGTATTTACTGCTGACCCAGTAGCAAACCCAGACGCAGAGCTGTATGATAAGCTATCTTACACTGAAATTCTGGATAAAGAATTGAAAGTAATGGACTTG | MN253266 |
| PJS23 | *Vibrio communis* | GGCGGTGGTAACTTGTTCCGTGGTGCAGGTCTTGCAGAAGCGGGTATGAACCGCGTAGTAGGCGACCACATGGGTATGCTTGCAACAGTAATGAACGGTCTAGCAATGCGTGACGCACTTCACCGTGCGTATGTAAACGCTCGCGTAATGTCTGCAATTCCGCTAAAAGGTGTGTGTGACGATTACAATTGGGCAGACGCTATCCGCGAACTTCGCCAAGGCCGTGTAGTTATCTTCTCAGCAGGTACCGGTAACCCATTCTTCACTACAGATTCAGCAGCGTGTCTACGCGGCATCGAAATCGAAGCTGACGTAGTTCTAAAAGCAACGAAAGTTGATGGTGTATTTACTGCAGACCCAGTAGCAAACCCAGACGCAGAGCTGTATGATAAGCTATCTTACACTGAAATTCTGGATAAAGAATTGAAAGTAATGGACTTG | MN253267 |
| PJL29 | *Vibrio communis* | GGCGGTGGTAACCTGTTCCGTGGTGCTGGTCTTGCAGAAGCTGGTATGAACCGCGTAGTGGGTGACCACATGGGTATGCTTGCAACAGTAATGAACGGTCTAGCAATGCGTGATGCACTTCACCGTGCTTATGTAAACGCTCGCGTAATGTCTGCAATTCCGCTTAAAGGTGTGTGTGACGACTACAATTGGGCAGACGCTATCCGCGAACTTCGCCAAGGCCGTGTGGTTATCTTCTCTGCAGGTACTGGTAACCCATTCTTCACTACTGATTCTGCGGCTTGTCTACGCGGCATTGAAATTGAAGCTGACGTCGTTCTGAAGGCTACCAAAGTTGATGGTGTATTTACTGCTGACCCAGTAGCAAACCCAGACGCAGAGCTGTATGATAAGCTATCTTACACTGAAATTCTGGATAAAGAATTGAAAGTAATGGACTTG | MN253268 |
| PJK29 | *Vibrio communis* | GGCGGTGGTAACCTGTTCCGTGGTGCTGGTCTTGCAGAAGCTGGTATGAACCGCGTAGTGGGTGACCACATGGGTATGCTTGCAACAGTAATGAACGGTCTAGCAATGCGTGATGCACTTCACCGTGCTTACGTAAACGCTCGCGTAATGTCTGCAATTCCGCTTAAAGGTGTGTGTGACGACTACAATTGGGCAGACGCTATCCGCGAACTTCGCCAAGGCCGTGTGGTTATCTTCTCTGCAGGTACTGGTAACCCATTCTTCACTACTGATTCTGCGGCTTGTCTACGCGGCATTGAAATTGAAGCTGACGTCGTTCTGAAGGCTACCAAAGTTGATGGTGTATTTACTGCTGACCCAGTAGCAAACCCAGACGCAGAGCTGTATGATAAGCTATCTTACACTGAAATTCTGGATAAAGAATTGAAAGTAATGGACTTG | MN253269 |
| PKGS05 | *Vibrio communis* | GGCGGTGGTAACTTGTTCCGTGGTGCAGGTCTTGCAGAAGCGGGTATGAACCGCGTAGTAGGCGACCACATGGGTATGCTTGCAACAGTAATGAACGGTCTAGCAATGCGTGACGCACTTCACCGTGCTTATGTAAACGCTCGCGTAATGTCTGCAATTCCGCTAAAAGGTGTGTGTGACGATTACAATTGGGCAGACGCTATCCGCGAACTTCGCCAAGGCCGTGTAGTTATCTTCTCTGCAGGTACTGGTAACCCATTCTTCACTACAGATTCAGCAGCGTGTCTACGCGGTATCGAAATCGAAGCTGACGTAGTTCTAAAAGCGACGAAAGTTGATGGTGTATTTACTGCTGACCCAGTAGCAAACCCAGACGCAGAGCTGTATGATAAGCTATCTTACACTGAAATTCTGGATAAAGAATTGAAAGTAATGGACTTG | MN253270 |
| PKGL13 | *Vibrio communis* | GGCGGTGGTAACTTGTTCCGTGGTGCAGGTCTTGCAGAAGCGGGTATGAACCGCGTAGTAGGCGACCACATGGGTATGCTTGCAACAGTAATGAACGGTCTAGCAATGCGTGATGCACTTCACCGTGCTTATGTAAACGCTCGCGTAATGTCTGCAATTCCGCTAAAAGGTGTGTGTGACGATTACAACTGGGCAGACGCTATCCGCGAACTTCGCCAAGGCCGTGTAGTGATCTTCTCTGCAGGTACTGGTAACCCATTCTTTACGACAGATTCAGCAGCTTGTCTACGTGGTATCGAAATCGAAGCTGACGTAGTTCTAAAAGCGACGAAAGTTGATGGTGTATTTACTGCTGACCCAGTAGCAAACCCAGACGCAGAGCTGTATGATAAGCTATCTTACACTGAAATTCTGGATAAAGAATTGAAAGTAATGGACTTG | MN253271 |
| PKGL20 | *Vibrio communis* | GGCGGTGGTAACTTGTTCCGTGGTGCAGGTCTTGCAGAAGCGGGTATGAACCGCGTAGTAGGCGACCACATGGGTATGCTTGCAACAGTAATGAACGGTCTAGCAATGCGTGACGCACTTCACCGTGCTTATGTAAACGCTCGCGTAATGTCTGCAATTCCGCTAAAAGGTGTGTGTGACGATTACAATTGGGCAGACGCTATCCGCGAACTTCGCCAAGGCCGTGTAGTTATCTTCTCTGCAGGTACTGGTAACCCATTCTTCACTACAGATTCAGCAGCGTGTCTACGCGGTATCGAAATCGAAGCTGACGTAGTTCTAAAAGCAACGAAAGTTGATGGTGTATTTACTGCTGACCCAGTAGCAAACCCAGACGCAGAGCTGTATGATAAGCTATCTTACACTGAAATTCTGGATAAAGAATTGAAAGTAATGGACTTG | MN253272 |
| TS01 | *Vibrio communis* | GGCGGTGGTAACCTGTTCCGTGGTGCAGGTCTTGCAGAAGCGGGTATGAACCGCGTAGTAGGCGACCACATGGGTATGCTTGCAACAGTAATGAACGGTCTAGCAATGCGTGACGCACTTCACCGTGCTTATGTAAACGCTCGCGTAATGTCTGCAATTCCGCTAAAAGGTGTGTGTGACGATTACAATTGGGCAGACGCTATCCGCGAACTTCGCCAAGGCCGTGTAGTTATCTTCTCTGCAGGTACTGGTAACCCTTTCTTCACGACAGATTCAGCAGCGTGTCTACGCGGCATCGAAATCGAAGCTGACGTTGTTCTAAAAGCAACGAAAGTTGATGGTGTATTTACCGCTGACCCAGTGGCCAATCCAGACGCAGAGCTGTATGATAAACTTTCTTACACTGAAATTCTTGATAAAGAACTGAAAGTAATGGACTTG | MN253273 |
| TK01 | *Vibrio communis* | GGCGGTGGTAACTTGTTCCGTGGTGCAGGTCTTGCAGAAGCGGGTATGAACCGCGTAGTAGGCGACCACATGGGTATGCTTGCAACAGTAATGAACGGTCTAGCAATGCGTGATGCACTTCACCGTGCTTATGTAAACGCTCGCGTAATGTCTGCAATTCCGCTAAAAGGTGTGTGTGACGATTACAACTGGGCAGACGCTATCCGCGAACTTCGCCAAGGCCGTGTAGTGATCTTCTCTGCAGGTACAGGTAACCCATTCTTTACGACGGATTCAGCAGCTTGTCTACGTGGTATCGAAATCGAAGCTGACGTAGTTCTAAAAGCGACAAAAGTTGATGGTGTATTTACTGCTGACCCAGTAGCAAACCCAGACGCAGAGCTGTATGATAAGCTATCTTACACTGAAATTCTGGATAAAGAATTGAAAGTAATGGACTTG | MN253274 |
| TL02 | *Vibrio communis* | GGCGGTGGTAACCTGTTCCGTGGTGCAGGTCTTGCAGAAGCGGGTATGAACCGCGTAGTAGGCGACCACATGGGTATGCTTGCAACAGTAATGAACGGTCTAGCAATGCGTGATGCACTTCACCGTGCTTATGTAAACGCTCGCGTAATGTCTGCAATTCCGCTAAAAGGTGTGTGTGACGATTACAACTGGGCAGACGCTATCCGCGAACTTCGCCAAGGCCGTGTAGTGATCTTCTCTGCAGGTACTGGTAACCCATTCTTTACGACAGATTCAGCAGCTTGTCTACGTGGTATCGAAATCGAAGCTGACGTAGTTCTAAAAGCGACAAAAGTTGATGGTGTATTTACTGCTGACCCAGTAGCAAACCCAGACGCAGAGCTGTATGATAAGCTATCTTACACTGAAATTCTGGATAAAGAATTGAAAGTAATGGACTTG | MN253275 |
| TS02 | *Vibrio communis* | GGCGGTGGTAACCTGTTCCGTGGTGCAGGTCTTGCAGAAGCGGGTATGAACCGCGTAGTAGGCGACCACATGGGTATGCTTGCAACAGTAATGAACGGTCTAGCAATGCGTGACGCACTTCACCGTGCTTATGTAAACGCTCGCGTAATGTCTGCAATTCCGCTAAAAGGTGTGTGTGACGATTACAATTGGGCAGACGCTATCCGCGAACTTCGCCAAGGCCGTGTAGTTATCTTCTCTGCAGGTACTGGTAACCCATTCTTCACGACAGATTCAGCAGCGTGTCTACGCGGCATCGAAATCGAAGCTGACGTAGTTCTAAAAGCAACGAAAGTTGATGGTGTATTTACTGCTGACCCAGTAGCAAACCCAGACGCAGAGCTGTATGATAAGCTATCTTACACTGAAATTCTGGATAAAGAATTGAAAGTAATGGACTTG | MN253276 |
| TL03 | *Vibrio communis* | GGTGGTGGTAATCTGTTCCGTGGTGCTGGTCTTGCAGAAGCTGGTATGAACCGCGTAGTGGGTGACCACATGGGTATGCTTGCAACAGTAATGAATGGTCTAGCAATGCGTGATGCACTTCACCGTGCTTATGTAAATGCTCGCGTAATGTCTGCAATTCCGCTTAAAGGTGTGTGTGACGATTACAATTGGGCAGACGCTATCCGCGAACTTCGCCAAGGCCGTGTGGTTATCTTCTCTGCAGGTACTGGTAACCCATTCTTCACTACTGATTCTGCGGCTTGCCTACGCGGCATTGAAATTGAAGCTGACGTCGTTCTGAAGGCTACCAAAGTTGATGGTGTATTTACTGCTGACCCAGTAGCAAACCCAGACGCAGAGCTGTATGATAAGCTATCTTACACTGAAATTCTGGATAAAGAATTGAAAGTAATGGACTTG | MN253277 |
| TS04 | *Vibrio communis* | GGCGGTGGTAACCTGTTCCGTGGTGCAGGTCTTGCAGAAGCGGGTATGAACCGCGTAGTAGGCGACCACATGGGTATGCTTGCAACAGTAATGAACGGTCTAGCAATGCGTGACGCACTTCACCGTGCTTATGTAAACGCTCGCGTAATGTCTGCAATTCCGCTAAAAGGTGTGTGTGACGATTACAATTGGGCAGACGCTATCCGCGAACTTCGCCAAGGCCGTGTAGTTATCTTCTCTGCAGGTACTGGTAACCCATTCTTCACGACAGATTCAGCAGCGTGTCTACGCGGCATCGAAATCGAAGCTGACGTAGTTCTAAAAGCAACGAAAGTTGATGGTGTATTTACTGCTGACCCAGTAGCAAACCCAGACGCAGAGCTGTATGATAAGCTATCTTACACTGAAATTCTGGATAAAGAATTGAAAGTAATGGACTTG | MN253278 |
| TK05 | *Vibrio communis* | GGCGGTGGTAACCTGTTCCGTGGTGCAGGTCTTGCAGAAGCGGGTATGAACCGCGTAGTAGGCGACCACATGGGTATGCTTGCAACAGTAATGAACGGTCTAGCAATGCGTGACGCACTTCACCGTGCTTATGTAAACGCTCGCGTAATGTCTGCAATTCCGCTAAAAGGTGTGTGTGACGATTACAATTGGGCAGACGCTATCCGCGAACTTCGCCAAGGCCGTGTAGTTATCTTCTCTGCAGGTACTGGTAACCCATTCTTCACGACAGATTCAGCAGCGTGTCTACGCGGCATCGAAATCGAAGCTGACGTAGTTCTAAAAGCAACGAAAGTTGATGGTGTATTTACTGCTGACCCAGTAGCAAACCCAGACGCAGAGCTGTATGATAAGCTATCTTACACTGAAATTCTGGATAAAGAATTGAAAGTAATGGACTTG | MN253279 |
| TL06 | *Vibrio communis* | GGCGGTGGTAACCTGTTCCGTGGTGCAGGTCTTGCAGAAGCGGGTATGAACCGCGTAGTAGGCGACCACATGGGTATGCTTGCAACAGTAATGAACGGTCTAGCAATGCGTGACGCACTTCACCGTGCTTATGTAAACGCTCGCGTAATGTCTGCAATTCCGCTAAAAGGTGTGTGTGACGATTACAATTGGGCAGACGCTATCCGCGAACTTCGCCAAGGCCGTGTAGTTATCTTCTCTGCAGGTACTGGTAACCCATTCTTCACGACAGATTCAGCAGCGTGTCTACGCGGCATCGAAATCGAAGCTGACGTAGTTCTAAAAGCAACGAAAGTTGATGGTGTATTTACTGCTGACCCAGTAGCAAACCCAGACGCAGAGCTGTATGATAAGCTATCTTACACTGAAATTCTGGATAAAGAATTGAAAGTAATGGACTTG | MN253280 |
| TL07 | *Vibrio communis* | GGCGGTGGCAACCTGTTCCGTGGTGCTGGTCTTGCAGAAGCTGGTATGAACCGCGTAGTGGGTGACCACATGGGTATGCTTGCAACAGTAATGAACGGTCTAGCAATGCGTGATGCACTTCACCGTGCTTATGTAAATGCTCGCGTGATGTCTGCAATTCCGCTTAAAGGTGTGTGTGATGACTACAATTGGGCAGACGCTATCCGCGAACTTCGCCAAGGCCGTGTGGTTATCTTCTCTGCAGGTACTGGTAACCCATTCTTCACTACTGATTCTGCGGCTTGTCTACGCGGCATTGAAATTGAAGCTGACGTAGTTCTAAAAGCAACGAAAGTTGATGGTGTATTTACTGCTGACCCAGTAGCAAACCCAGACGCAGAGCTGTATGATAAGCTATCTTACACTGAAATTCTGGATAAAGAATTGAAAGTAATGGACTTG | MN253281 |
| TS07 | *Vibrio communis* | GGCGGTGGCAACCTGTTCCGTGGTGCTGGTCTTGCAGAAGCTGGTATGAACCGCGTAGTGGGTGACCACATGGGTATGCTTGCAACAGTAATGAATGGTCTAGCAATGCGTGATGCACTTCACCGTGCTTATGTAAATGCTCGCGTGATGTCTGCAATTCCGCTTAAAGGTGTGTGTGACGATTACAATTGGGCAGACGCTATCCGCGAACTTCGCCAAGGCCGTGTGGTTATCTTCTCTGCAGGTACTGGTAACCCATTCTTCACTACTGATTCTGCGGCTTGTCTACGCGGGATTGAAATTGAAGCTGACGTAGTTCTAAAAGCAACGAAAGTTGATGGTGTATTTACTGCTGACCCAGTAGCAAACCCAGACGCAGAGCTGTATGATAAGCTATCTTACACTGAAATTCTGGATAAAGAATTGAAAGTAATGGACTTG | MN253282 |
| TS08 | *Vibrio communis* | GGCGGTGGTAACCTGTTCCGTGGTGCAGGTCTTGCAGAAGCGGGTATGAACCGCGTAGTAGGCGACCACATGGGTATGCTTGCAACAGTAATGAACGGTCTAGCAATGCGTGACGCACTTCACCGTGCTTATGTAAACGCTCGCGTAATGTCTGCAATTCCGCTAAAAGGTGTGTGTGACGATTACAATTGGGCAGACGCTATCCGCGAACTTCGCCAAGGCCGTGTAGTTATCTTCTCTGCAGGTACTGGTAACCCATTCTTCACGACAGATTCAGCAGCGTGTCTACGCGGCATCGAAATCGAAGCTGACGTAGTTCTAAAAGCAACGAAAGTTGATGGTGTATTTACTGCTGACCCAGTAGCAAACCCAGACGCAGAACTGTATGATAAGCTATCTTACACTGAAATTCTGGATAAAGAATTGAAAGTAATGGACTTG | MN253283 |
| TL09 | *Vibrio communis* | GGCGGTGGTAACCTGTTCCGTGGTGCTGGTCTTGCAGAAGCTGGTATGAACCGCGTAGTGGGTGACCACATGGGTATGCTTGCAACAGTAATGAATGGTCTAGCAATGCGTGATGCACTTCACCGTGCTTATGTAAATGCTCGCGTGATGTCTGCAATTCCGCTTAAAGGTGTGTGTGATGACTACAATTGGGCAGACGCTATCCGCGAACTTCGCCAAGGCCGTGTGGTTATCTTCTCTGCAGGTACTGGTAACCCATTCTTCACGACAGATTCAGCAGCGTGTCTACGCGGCATCGAAATCGAAGCTGACGTAGTTCTAAAAGCAACGAAAGTTGATGGTGTATTTACTGCTGACCCAGTAGCAAACCCAGACGCAGAACTGTATGATAAGCTATCTTACACTGAAATTCTGGATAAAGAATTGAAAGTAATGGACTTG | MN253284 |
| TL11 | *Vibrio communis* | GGCGGTGGTAACCTGTTCCGTGGTGCAGGTCTTGCAGAAGCGGGTATGAACCGCGTAGTAGGCGACCACATGGGTATGCTTGCAACAGTAATGAACGGTCTAGCAATGCGTGATGCACTTCACCGTGCTTATGTAAACGCTCGCGTAATGTCTGCAATTCCGCTAAAAGGTGTGTGTGACGATTACAACTGGGCAGACGCTATCCGCGAACTTCGCCAAGGCCGTGTAGTTATCTTCTCTGCAGGTACTGGTAACCCATTCTTCACGACAGATTCAGCAGCGTGTCTACGCGGCATCGAAATCGAAGCTGACGTAGTTCTAAAAGCAACGAAAGTTGATGGTGTATTTACTGCTGACCCAGTAGCAAACCCAGACGCAGAACTGTATGATAAGCTATCTTACACTGAAATTCTGGATAAAGAATTGAAAGTAATGGACTTG | MN253285 |
| TK30 | *Vibrio communis* | GGCGGTGGTAACCTGTTCCGTGGTGCAGGTCTTGCAGAAGCGGGTATGAACCGCGTAGTGGGCGACCACATGGGTATGCTTGCAACAGTAATGAACGGTCTAGCAATGCGTGACGCACTTCACCGTGCTTATGTAAACGCTCGCGTAATGTCTGCAATTCCGCTAAAAGGTGTGTGTGACGATTACAATTGGGCAGACGCTATCCGCGAACTTCGCCAAGGCCGTGTAGTTATCTTCTCTGCAGGTACTGGTAACCCATTCTTCACGACAGATTCTGCTGCGTGTCTACGCGGCATCGAAATCGAAGCCGACGTAGTTCTAAAAGCAACGAAAGTTGATGGTGTATTTACTGCTGACCCAGTAGCTAACCCAGACGCAGAACTGTATGATAAGCTATCTTACACTGAAATTCTGGATAAAGAATTGAAAGTAATGGACTTG | MN253286 |
| BS02 | *Vibrio communis* | GGCGGTGGTAACCTGTTCCGTGGTGCAGGTCTTGCAGAAGCGGGTATGAACCGCGTAGTAGGCGACCACATGGGTATGCTTGCAACAGTAATGAACGGTCTAGCAATGCGTGACGCACTTCACCGTGCTTATGTAAACGCTCGCGTAATGTCTGCAATTCCGCTAAAAGGTGTGTGTGACGATTACAACTGGGCAGACGCTATCCGCGAACTTCGCCAAGGCCGTGTAGTTATCTTCTCTGCAGGTACTGGTAACCCATTCTTCACGACAGATTCTGCTGCGTGTCTACGCGGCATCGAAATCGAAGCTGACGTAGTACTAAAAGCAACGAAAGTTGATGGTGTATTTACTGCTGACCCAGTAGCTAACCCAGACGCAGAACTGTATGATAAGCTATCTTACACTGAAATTCTGGATAAAGAATTGAAAGTAATGGACTTG | MN253287 |
| BK05 | *Vibrio communis* | GGCGGTGGTAACTTGTTCCGTGGTGCAGGTCTTGCAGAAGCGGGTATGAACCGCGTAGTAGGCGACCACATGGGTATGCTTGCAACAGTAATGAACGGTCTAGCAATGCGTGACGCACTTCACCGTGCTTATGTAAACGCTCGCGTAATGTCTGCAATTCCGCTAAAAGGTGTGTGTGACGATTACAATTGGGCAGACGCTATCCGCGAACTTCGCCAAGGCCGTGTAGTTATCTTCTCTGCAGGTACTGGTAACCCATTCTTCACGACAGATTCTGCTGCGTGTCTACGCGGCATCGAAATCGAAGCTGACGTAGTTCTAAAAGCAACGAAAGTTGATGGTGTATTTACTGCTGACCCAGTAGCTAACCCAGACGCAGAACTGTATGATAAGCTATCTTACACTGAAATTCTGGATAAAGAATTGAAAGTAATGGACTTG | MN253288 |
| BL06 | *Vibrio communis* | GGCGGTGGTAACTTGTTCCGTGGTGCAGGTCTTGCAGAAGCGGGTATGAACCGCGTAGTAGGCGACCACATGGGTATGCTTGCAACAGTAATGAACGGTCTAGCAATGCGTGACGCACTTCACCGTGCTTATGTAAACGCTCGCGTAATGTCTGCAATTCCGCTAAAAGGTGTGTGTGACGATTACAATTGGGCAGACGCTATCCGCGAACTTCGCCAAGGCCGTGTAGTTATCTTCTCTGCAGGTACTGGTAACCCATTCTTCACGACAGATTCAGCAGCGTGTCTACGCGGCATCGAAATCGAAGCTGACGTAGTTCTAAAAGCAACGAAAGTTGATGGTGTATTTACTGCTGACCCAGTAGCAAACCCAGACGCAGAGCTGTATGATAAGCTATCTTACACTGAAATTCTGGATAAAGAATTGAAAGTAATGGACTTG | MN253289 |
| BL14 | *Vibrio communis* | GGCGGTGGTAACCTGTTCCGTGGTGCAGGTCTTGCAGAAGCGGGTATGAACCGCGTAGTAGGCGACCACATGGGTATGCTTGCAACAGTAATGAACGGTCTAGCAATGCGTGACGCACTTCACCGTGCTTATGTAAACGCTCGCGTAATGTCTGCAATTCCGCTAAAAGGTGTGTGTGACGATTACAATTGGGCAGACGCTATCCGCGAACTTCGCCAAGGCCGTGTAGTTATCTTCTCTGCAGGTACTGGTAACCCATTCTTCACGACAGATTCAGCAGCGTGTCTACGCGGTATCGAAATCGAAGCTGACGTAGTTCTAAAAGCAACGAAAGTTGATGGTGTATTTACTGCTGACCCAGTAGCAAACCCAGACGCAGAGCTGTATGATAAGCTATCTTACACTGAAATTCTGGATAAAGAATTGAAAGTAATGGACTTG | MN253290 |
| BS14 | *Vibrio communis* | GGCGGTGGTAACTTGTTCCGTGGTGCAGGTCTTGCAGAAGCGGGTATGAACCGCGTAGTAGGCGACCACATGGGTATGCTTGCAACAGTAATGAACGGTCTAGCAATGCGTGACGCACTTCACCGTGCTTATGTAAACGCTCGCGTAATGTCTGCAATTCCGCTAAAAGGTGTGTGTGACGATTACAACTGGGCAGACGCTATCCGCGAACTTCGCCAAGGCCGTGTAGTTATCTTCTCTGCAGGTACTGGTAACCCATTCTTCACGACAGATTCAGCAGCGTGTCTACGCGGTATCGAAATCGAAGCTGACGTAGTTCTAAAAGCAACGAAAGTTGATGGTGTATTTACTGCTGACCCAGTAGCAAACCCAGACGCAGAGCTGTATGATAAGCTATCTTACACTGAAATTCTGGATAAAGAATTGAAAGTAATGGACTTG | MN253291 |
| BL15 | *Vibrio communis* | GGCGGTGGTAACTTGTTCCGTGGTGCAGGTCTTGCAGAAGCGGGTATGAACCGCGTAGTAGGCGACCACATGGGTATGCTTGCAACAGTAATGAACGGTCTAGCAATGCGTGACGCACTTCACCGTGCTTATGTAAACGCTCGCGTAATGTCTGCAATTCCGCTAAAAGGTGTGTGTGACGATTACAACTGGGCAGACGCTATCCGCGAACTTCGCCAAGGCCGTGTAGTTATCTTCTCTGCAGGTACTGGTAACCCATTCTTCACGACAGATTCAGCAGCGTGTCTACGCGGTATCGAAATCGAAGCTGACGTAGTTCTAAAAGCAACGAAAGTTGATGGTGTATTTACTGCTGACCCAGTAGCAAACCCAGACGCAGAGCTGTATGATAAGCTATCTTACACTGAAATTCTGGATAAAGAATTGAAAGTAATGGACTTG | MN253292 |
| BL17 | *Vibrio communis* | GGCGGTGGTAACCTGTTCCGTGGTGCAGGTCTTGCAGAAGCGGGTATGAACCGCGTAGTAGGCGACCACATGGGTATGCTTGCAACAGTAATGAACGGTCTAGCAATGCGTGATGCACTTCACCGTGCTTATGTAAACGCTCGCGTAATGTCTGCAATTCCGCTAAAAGGTGTGTGTGACGATTACAATTGGGCAGACGCTATCCGCGAACTTCGCCAAGGCCGTGTAGTTATCTTCTCTGCAGGTACTGGTAACCCATTCTTCACAACAGATTCAGCAGCGTGTCTACGCGGTATCGAAATCGAAGCTGACGTAGTTCTAAAAGCAACGAAAGTTGATGGTGTATTTACAGCTGACCCAGTAGCAAACCCAGACGCAGAGCTGTATGATAAGCTATCTTACACTGAAATTCTGGATAAAGAATTGAAAGTAATGGACTTG | MN253293 |
| BS18 | *Vibrio communis* | GGCGGTGGTAACTTGTTCCGTGGTGCAGGTCTTGCAGAAGCGGGTATGAACCGCGTAGTAGGCGACCACATGGGTATGCTTGCAACAGTAATGAACGGTCTAGCAATGCGTGACGCACTTCACCGTGCTTATGTAAACGCTCGCGTAATGTCTGCAATTCCGCTAAAAGGTGTGTGTGACGATTACAATTGGGCAGACGCTATCCGCGAACTTCGCCAAGGCCGTGTAGTTATCTTCTCTGCAGGTACTGGTAACCCATTCTTCACGACAGATTCAGCAGCGTGTCTACGCGGCATCGAAATCGAAGCTGACGTAGTTCTAAAAGCAACGAAAGTTGATGGTGTATTTACTGCTGACCCAGTAGCAAACCCAGACGCAGAGCTGTATGATAAGCTATCTTACACTGAAATTCTGGATAAAGAATTGAAAGTAATGGACTTG | MN253294 |
| BK25 | *Vibrio communis* | GGCGGTGGTAACTTGTTCCGTGGTGCAGGTCTTGCAGAAGCGGGTATGAACCGCGTAGTAGGCGACCACATGGGTATGCTTGCAACAGTAATGAACGGTCTAGCAATGCGTGATGCACTTCACCGTGCTTATGTAAACGCTCGCGTAATGTCTGCAATTCCGCTAAAAGGTGTGTGTGACGATTACAATTGGGCAGACGCTATCCGCGAACTTCGCCAAGGCCGTGTAGTTATCTTCTCTGCAGGTACTGGTAACCCATTCTTCACTACAGATTCAGCAGCGTGTCTACGCGGCATCGAAATCGAAGCTGACGTAGTTCTAAAAGCAACGAAAGTTGATGGTGTATTTACTGCTGACCCAGTAGCAAACCCAGACGCAGAGCTGTATGATAAGCTATCTTACACTGAAATTCTGGATAAAGAATTGAAAGTAATGGACTTG | MN253295 |
| PKS01 | *Vibrio communis* | GGCGGTGGTAACCTGTTCCGTGGTGCTGGTCTTGCAGAAGCTGGTATGAACCGCGTAGTGGGTGACCACATGGGTATGCTTGCAACAGTAATGAACGGTCTAGCAATGCGTGACGCACTTCACCGTGCTTATGTAAACGCTCGCGTAATGTCTGCAATTCCGCTTAAAGGTGTGTGTGACGATTACAATTGGGCAGACGCTATCCGCGAACTTCGCCAAGGCCGTGTGGTTATCTTCTCTGCAGGTACTGGTAACCCATTCTTCACGACTGATTCTGCGGCTTGTCTACGCGGCATTGAAATAGAAGCTGACGTAGTTCTAAAAGCAACGAAAGTTGATGGTGTATTTACTGCTGACCCAGTAGCAAACCCAGACGCAGAGCTGTATGATAAGCTATCTTACACTGAAATTCTGGATAAAGAATTGAAAGTAATGGACTTG | MN253296 |
| PKL02 | *Vibrio communis* | GGCGGTGGTAACCTGTTCCGTGGTGCAGGTCTTGCAGAAGCTGGTATGAACCGCGTAGTGGGCGACCACATGGGTATGCTTGCAACAGTAATGAACGGTCTAGCAATGCGTGACGCACTTCACCGTGCTTATGTAAACGCTCGCGTAATGTCTGCAATTCCGCTTAAAGGTGTGTGTGACGATTACAATTGGGCAGACGCTATCCGCGAACTTCGCCAAGGCCGTGTGGTTATCTTCTCTGCAGGTACTGGTAACCCATTCTTCACTACTGATTCTGCGGCTTGTCTACGCGGCATTGAAATAGAGGCTGACGTAGTTCTAAAAGCAACGAAAGTTGATGGTGTATTTACTGCTGACCCAGTAGCAAACCCAGACGCAGAGCTGTATGATAAGCTATCTTACACTGAAATTCTGGATAAAGAATTGAAAGTAATGGACTTG | MN253297 |
| PKS02 | *Vibrio communis* | GGCGGTGGTAACCTGTTCCGTGGTGCTGGTCTTGCAGAAGCTGGTATGAACCGCGTAGTGGGTGACCACATGGGTATGCTTGCAACAGTAATGAACGGTCTAGCAATGCGTGACGCACTTCACCGTGCTTATGTAAATGCTCGCGTGATGTCTGCAATTCCGCTTAAAGGTGTGTGTGATGACTACAATTGGGCAGACGCTATCCGCGAACTTCGCCAAGGCCGTGTGGTTATCTTCTCTGCAGGTACTGGTAACCCATTCTTCACGACTGATTCTGCGGCTTGTCTACGCGGCATTGAAATTGAAGCTGATGTAGTTCTAAAAGCAACGAAAGTTGATGGTGTATTTACTGCTGACCCAGTAGCAAACCCAGACGCAGAGCTGTATGATAAGCTATCTTACACTGAAATTCTGGATAAAGAATTGAAAGTAATGGACTTG | MN253298 |
| PKK02 | *Vibrio communis* | GGCGGTGGTAACCTGTTCCGTGGTGCTGGTCTTGCAGAAGCTGGTATGAACCGCGTAGTGGGTGACCACATGGGTATGCTTGCAACAGTAATGAACGGTCTAGCAATGCGTGACGCACTTCACCGTGCTTATGTAAACGCTCGCGTAATGTCTGCAATTCCGCTTAAAGGTGTGTGTGACGATTACAATTGGGCAGACGCTATCCGCGAACTTCGCCAAGGCCGTGTGGTTATCTTCTCTGCAGGTACTGGTAACCCATTCTTCACGACTGATTCTGCGGCTTGTCTACGCGGCATTGAAATTGAAGCTGACGTAGTTCTAAAAGCAACGAAAGTTGATGGTGTATTTACTGCTGACCCAGTAGCAAACCCAGACGCAGAGCTGTATGATAAGCTATCTTACACTGAAATTCTGGATAAAGAATTGAAAGTAATGGACTTG | MN253299 |
| PKL03 | *Vibrio communis* | GGCGGTGGTAACCTGTTCCGTGGTGCTGGTCTTGCAGAAGCTGGTATGAACCGCGTAGTGGGTGACCACATGGGTATGCTTGCAACAGTAATGAACGGTCTAGCAATGCGTGACGCACTTCACCGTGCTTATGTAAACGCTCGCGTAATGTCTGCAATTCCGCTTAAAGGTGTGTGTGACGATTATAATTGGGCAGACGCTATCCGCGAACTTCGCCAAGGCCGGGTGGTTATCTTCTCTGCAGGTACTGGTAACCCATTCTTCACGACTGATTCTGCGGCTTGTCTACGCGGCATCGAAATTGAAGCTGACGTAGTTCTAAAAGCAACGAAAGTTGATGGTGTATTTACTGCTGACCCAGTAGCAAACCCAGACGCAGAGCTGTATGATAAGCTATCTTACACTGAAATTCTGGATAAAGAATTGAAAGTAATGGACTTG | MN253300 |
| PKK03 | *Vibrio communis* | GGCGGTGGTAACCTGTTCCGTGGTGCAGGTCTTGCAGAAGCTGGTATGAACCGCGTAGTGGGCGACCACATGGGTATGCTTGCAACAGTAATGAACGGTCTAGCAATGCGTGACGCACTTCACCGTGCTTATGTAAACGCTCGCGTGATGTCTGCAATTCCGCTTAAAGGTGTGTGTGACGATTACAATTGGGCAGACGCTATCCGCGAACTTCGCCAAGGCCGTGTGGTTATCTTCTCTGCAGGTACTGGTAACCCATTCTTCACGACTGATTCTGCGGCTTGTCTACGCGGCATTGAAATTGAAGCTGACGTAGTTCTAAAAGCAACGAAAGTTGATGGTGTATTTACTGCTGACCCAGTAGCAAACCCAGACGCAGAGCTGTATGATAAGCTATCTTACACTGAAATTCTGGATAAAGAATTGAAAGTAATGGACTTG | MN253301 |
| PKS04 | *Vibrio communis* | GGGGGTGGTAACCTGTTCCGTGGTGCAGGTCTTGCAGAAGCTGGTATGAACCGCGTAGTGGGTGACCACATGGGTATGCTTGCAACAGTAATGAATGGTCTAGCAATGCGTGATGCACTTCACCGTGCTTATGTAAACGCTCGCGTGATGTCTGCAATTCCGCTTAAAGGTGTGTGTGATGACTACAATTGGGCAGACGCTATCCGCGAACTTCGCCAAGGCCGTGTGGTTATCTTCTCTGCAGGTACTGGTAACCCATTCTTCACGACTGATTCTGCAGCGTGTCTACGCGGCATTGAAATTGAAGCTGACGTAGTTCTAAAAGCAACGAAAGTTGATGGTGTATTTACTGCTGACCCAGTAGCAAACCCAGACGCAGAGCTGTATGATAAGCTATCTTACACTGAAATTCTGGATAAAGAATTGAAAGTAATGGACTTG | MN253302 |
| PKK06 | *Vibrio communis* | GGCGGTGGTAACCTGTTCCGTGGTGCAGGTCTTGCAGAAGCTGGAATGAACCGCGTAGTGGGTGACCACATGGGTATGCTTGCAACAGTAATGAATGGCCTAGCGATGCGTGATGCACTTCACCGTGCTTACGTAAACGCTCGCGTGATGTCTGCAATTCCGCTTAAAGGTGTGTGTGATGACTACAATTGGGCAGACGCTATCCGCGAACTTCGCCAAGGCCGTGTGGTTATCTTCTCTGCAGGTACTGGTAACCCATTCTTCACTACTGATTCCGCGGCGTGTCTACGTGGCATCGAAATCGAAGCTGACGTAGTACTAAAAGCAACGAAAGTTGATGGTGTATTTACTGCTGACCCAGTAGCAAACCCAGACGCAGAGCTGTATGATAAGCTATCTTACACTGAAATTCTGGATAAAGAATTGAAAGTAATGGACTTG | MN253303 |
| PKK10 | *Vibrio communis* | GGGGGTGGTAACCTGTTCCGTGGTGCAGGTCTTGCAGAAGCTGGTATGAACCGCGTAGTGGGTGACCACATGGGTATGCTTGCAACAGTAATGAATGGCCTAGCGATGCGTGATGCACTTCACCGTGCTTATGTAAACGCTCGCGTGATGTCTGCAATTCCGCTTAAAGGTGTGTGTGACGACTACAATTGGGCAGACGCTATCCGCGAACTTCGCCAAGGCCGTGTGGTTATCTTCTCTGCAGGTACTGGTAACCCATTCTTCACTACTGATTCCGCAGCGTGTCTACGTGGCATCGAAATCGAAGCTGACGTAGTACTAAAAGCAACGAAAGTTGATGGTGTATTTACTGCTGACCCAGTAGCAAACCCAGACGCAGAGCTGTATGATAAGCTATCTTACACTGAAATTCTGGATAAAGAATTGAAAGTAATGGACTTG | MN253304 |
| PKL18 | *Vibrio communis* | GGCGGTGGTAACCTGTTTCGCGGGGCAGGTCTTGCAGAAGCTGGTATGAACCGCGTAGTGGGTGACCACATGGGTATGCTTGCAACAGTAATGAATGGCCTAGCGATGCGTGATGCACTTCACCGTGCTTACGTAAACGCTCGCGTGATGTCTGCAATTCCGCTTAAAGGTGTGTGTGATGACTACAATTGGGCAGACGCTATCCGCGAACTTCGCCAAGGCCGTGTGGTTATCTTCTCTGCAGGTACTGGTAACCCATTCTTCACTACTGATTCCGCAGCGTGTCTACGCGGCATCGAAATCGAAGCTGATGTAGTACTAAAAGCAACGAAAGTTGATGGTGTATTTACTGCTGACCCAGTAGCAAACCCAGACGCAGAGCTGTATGATAAGCTATCTTACACTGAAATTCTGGATAAAGAATTGAAAGTAATGGACTTG | MN253305 |
| PKS19 | *Vibrio communis* | GGCGGTGGTAACCTGTTTCGCGGTGCAGGTCTTGCAGAAGCTGGTATGAACCGCGTCGTAGGCGACCACATGGGTATGCTTGCAACAGTGATGAATGGCCTAGCGATGCGTGATGCACTTCACCGTGCTTACGTAAACGCTCGCGTGATGTCTGCAATTCCGCTTAAAGGTGTGTGTGATGACTACAATTGGGCAGACGCTATCCGCGAACTTCGCCAAGGCCGTGTGGTTATCTTCTCTGCAGGTACTGGTAACCCATTCTTCACTACTGATTCCGCAGCGTGTCTACGCGGCATCGAAATCGAAGCTGATGTAGTACTAAAAGCAACGAAAGTTGATGGTGTATTTACTGCTGACCCAGTAGCAAACCCAGACGCAGAGCTGTATGATAAGCTATCTTACACTGAAATTCTGGATAAAGAATTGAAAGTAATGGACTTG | MN253306 |
| PKK19 | *Vibrio communis* | GGGGGTGGTAACCTGTTCCGTGGTGCAGGTCTTGCAGAAGCTGGTATGAACCGCGTAGTGGGTGACCACATGGGTATGCTTGCAACAGTAATGAATGGCCTAGCGATGCGTGATGCACTTCACCGTGCTTACGTAAACGCTCGCGTGATGTCTGCAATTCCGCTTAAAGGTGTGTGTGATGACTACAATTGGGCAGACGCTATCCGCGAACTTCGCCAAGGCCGTGTGGTTATCTTCTCTGCAGGTACTGGTAACCCATTCTTCACTACTGATTCCGCAGCGTGTCTACGCGGCATCGAAATCGAAGCTGATGTAGTACTAAAAGCAACGAAAGTTGATGGTGTATTTACTGCTGACCCAGTAGCAAACCCAGACGCAGAGCTGTATGATAAGCTATCTTACACTGAAATTCTGGATAAAGAATTGAAAGTAATGGACTTG | MN253307 |
| NL09 | *Vibrio communis* | GGCGGTGGTAACCTGTTCCGTGGTGCAGGTCTTGCAGAAGCGGGTATGAACCGCGTAGTAGGCGACCACATGGGTATGCTTGCAACAGTAATGAACGGTCTAGCAATGCGTGACGCACTTCACCGTGCTTATGTAAACGCTCGCGTAATGTCTGCAATTCCGCTAAAAGGTGTGTGTGACGATTACAACTGGGCAGACGCTATCCGCGAACTTCGCCAAGGCCGTGTAGTTATCTTCTCTGCAGGTACTGGTAACCCATTCTTCACTACAGATTCAGCAGCGTGTCTACGCGGTATCGAAATCGAAGCTGACGTAGTTCTAAAAGCAACGAAAGTTGATGGTGTATTTACTGCTGACCCAGTAGCAAACCCAGACGCAGAACTGTATGATAAGCTATCTTACACTGAAATTCTGGATAAAGAATTGAAAGTAATGGACTTG | MN253308 |
| NS12 | *Vibrio communis* | GGCGGTGGTAACCTGTTCCGTGGTGCAGGTCTTGCTGAAGCGGGTATGAACCGCGTAGTAGGCGACCACATGGGTATGCTTGCAACAGTAATGAATGGTCTAGCGATGCGTGACGCACTTCACCGTGCTTACGTAAACGCTCGCGTAATGTCTGCAATTCCGCTAAAAGGTGTGTGTGACGATTACAACTGGGCAGACGCTATCCGCGAACTTCGCCAAGGCCGTGTAGTTATCTTCTCTGCAGGTACTGGTAACCCATTCTTCACGACAGATTCAGCAGCGTGTCTACGCGGCATCGAAATCGAAGCTGACGTAGTTCTAAAAGCAACGAAAGTTGATGGTGTATTTACTGCTGACCCAGTAGCAAACCCAGACGCAGAGCTGTATGATAAGCTATCTTACACTGAAATTCTGGATAAAGAATTGAAAGTAATGGACTTG | MN253309 |
| NS14 | *Vibrio communis* | GGTGGTGGTAACTTGTTCCGTGGTGCAGGTCTTGCAGAAGCGGGTATGAACCGCGTAGTAGGCGACCACATGGGTATGCTTGCAACAGTAATGAACGGTCTAGCAATGCGTGACGCACTTCACCGTGCTTATGTAAACGCTCGCGTAATGTCTGCAATTCCGCTAAAAGGTGTGTGTGACGATTACAACTGGGCAGACGCTATCCGCGAACTTCGCCAAGGCCGTGTAGTTATCTTCTCTGCAGGTACTGGTAACCCATTCTTCACTACAGATTCAGCAGCGTGTCTACGCGGTATCGAAATCGAAGCTGACGTAGTTCTAAAAGCAACGAAAGTTGATGGTGTATTTACTGCTGACCCAGTAGCAAACCCAGACGCAGAGCTGTATGATAAGCTATCTTACACTGAAATTCTGGATAAAGAATTGAAAGTAATGGACTTG | MN253310 |
| NS17 | *Vibrio communis* | GGCGGTGGTAACCTGTTCCGTGGTGCAGGTCTTGCAGAAGCGGGTATGAACCGCGTAGTAGGCGACCACATGGGTATGCTTGCAACAGTAATGAACGGTCTAGCAATGCGTGACGCACTTCACCGTGCTTATGTAAACGCTCGCGTAATGTCTGCAATTCCGCTAAAAGGTGTGTGTGACGATTACAATTGGGCAGACGCTATCCGCGAACTTCGCCAAGGCCGTGTAGTTATCTTCTCTGCAGGTACTGGTAACCCATTCTTCACGACAGATTCTGCTGCTTGCCTACGTGGTATTGAGATTGAAGCGGATGTAGTTCTAAAAGCGACTAAAGTGGATGGCGTATTTACTGCTGACCCGGTAGCCAACCCAGACGCAGAGCTGTATGATAAGCTTTCTTACACCGAAATTCTTGATAAAGAACTGAAAGTGATGGACTTG | MN253311 |
| NL22 | *Vibrio communis* | GGCGGTGGTAACCTGTTCCGTGGTGCAGGTCTTGCAGAAGCGGGTATGAACCGCGTAGTAGGCGACCACATGGGTATGCTTGCAACAGTAATGAACGGTCTAGCAATGCGTGACGCACTTCACCGTGCTTATGTAAACGCTCGCGTAATGTCTGCAATTCCGCTAAAAGGTGTGTGTGACGATTACAATTGGGCAGACGCTATCCGCGAACTTCGCCAAGGCCGTGTAGTTATCTTCTCTGCAGGTACTGGTAACCCATTCTTCACTACAGATTCAGCAGCGTGTCTACGCGGTATCGAAATCGAAGCTGACGTAGTTCTAAAAGCAACGAAAGTTGATGGTGTATTTACTGCTGACCCAGTAGCAAACCCAGACGCAGAGCTGTATGATAAGCTATCTTACACTGAAATTCTGGATAAAGAATTGAAAGTAATGGACTTG | MN253312 |
| NK23 | *Vibrio communis* | GGCGGTGGTAACCTGTTCCGTGGTGCAGGTCTTGCAGAAGCGGGTATGAACCGCGTAGTAGGCGACCACATGGGTATGCTTGCAACAGTAATGAACGGTCTAGCAATGCGTGACGCACTTCACCGTGCTTATGTAAACGCTCGCGTAATGTCTGCAATTCCGCTAAAAGGTGTGTGTGACGATTACAATTGGGCAGACGCTATCCGCGAACTTCGCCAAGGCCGTGTAGTTATCTTCTCTGCAGGTACTGGTAACCCATTCTTCACGACAGATTCAGCAGCGTGTCTACGCGGCATCGAAATCGAAGCTGACGTAGTTCTAAAAGCAACGAAAGTTGATGGGGTATTTACTGCTGACCCAGTAGCAAACCCAGACGCAGAGCTGTATGATAAGCTATCTTACACTGAAATTCTGGATAAAGAATTGAAAGTAATGGACTTG | MN253313 |
| NS26 | *Vibrio communis* | GGTGGTGGTAACCTGTTCCGTGGTGCAGGTCTTGCGGAAGCGGGTATGAACCGCGTAGTAGGCGACCACATGGGTATGCTTGCAACAGTAATGAACGGTCTAGCAATGCGTGACGCACTTCACCGTGCTTATGTAAACGCTCGCGTAATGTCTGCAATTCCGCTAAAAGGTGTGTGTGACGACTACAATTGGGCAGACGCTATCCGCGAACTTCGCCAAGGCCGTGTAGTAATCTTCTCTGCAGGTACTGGTAACCCATTCTTCACGACAGATTCAGCAGCGTGTCTACGCGGCATCGAAATCGAAGCTGACGTAGTTCTAAAAGCAACGAAAGTTGATGGTGTATTTACTGCTGACCCAGTAGCAAACCCAGACGCAGAGCTGTATGATAAGCTATCTTACACTGAAATTCTGGATAAAGAATTGAAAGTGATGGACTTG | MN253314 |
| NK27 | *Vibrio communis* | GGCGGTGGTAACCTGTTCCGTGGTGCAGGTCTTGCAGAAGCGGGTATGAACCGCGTAGTAGGCGACCACATGGGTATGCTTGCAACAGTAATGAACGGTCTAGCAATGCGTGACGCACTTCACCGTGCTTATGTAAACGCTCGCGTAATGTCTGCAATTCCGCTAAAAGGTGTGTGTGACGATTACAACTGGGCAGACGCTATCCGCGAACTTCGCCAAGGCCGTGTAGTTATCTTCTCTGCAGGTACTGGTAACCCATTCTTCACTACAGATTCAGCAGCGTGTCTACGCGGTATCGAAATCGAAGCTGACGTAGTTCTAAAAGCAACGAAAGTTGATGGTGTATTTACAGCTGACCCAGTAGCAAACCCAGACGCAGAGCTGTATGATAAGCTATCTTACACTGAAATTCTGGATAAAGAATTGAAAGTAATGGACTTG | MN253315 |
| NL28 | *Vibrio communis* | GGCGGTGGTAACCTGTTCCGTGGTGCAGGTCTTGCAGAAGCGGGTATGAACCGCGTAGTAGGCGACCACATGGGTATGCTTGCAACAGTAATGAACGGTCTAGCAATGCGTGACGCACTTCACCGTGCTTATGTAAACGCTCGCGTAATGTCTGCAATTCCGCTAAAAGGTGTGTGTGACGATTACAATTGGGCAGACGCTATCCGCGAACTTCGCCAAGGCCGTGTAGTAATCTTCTCTGCAGGTACCGGTAACCCATTCTTCACGACAGATTCAGCAGCGTGTCTACGCGGCATCGAAATCGAAGCTGACGTTGTTCTAAAAGCAACGAAAGTTGATGGTGTATTTACTGCTGACCCAGTAGCAAACCCAGACGCAGAGCTGTATGATAAGCTATCTTACACTGAAATTCTGGATAAAGAATTGAAAGTGATGGACTTG | MN253316 |
| NS28 | *Vibrio communis* | GGCGGTGGTAACTTGTTCCGTGGTGCAGGTCTAGCAGAAGCTGGTATGAACCGCGTAGTAGGCGACCACATGGGTATGCTTGCTACAGTAATGAATGGCCTAGCAATGCGTGATGCACTTCACCGTGCTTATGTAAACGCTCGCGTAATGTCTGCAATTCCGCTAAAAGGTGTGTGTGACGATTACAATTGGGCAGACGCTATCCGCGAACTTCGCCAAGGCCGTGTAGTTATCTTCTCAGCAGGTACCGGTAACCCATTCTTCACTACAGATTCAGCAGCGTGTCTACGCGGCATCGAAATCGAAGCTGACGTAGTTCTAAAAGCAACGAAAGTTGATGGTGTATTTACTGCAGACCCAGTAGCAAACCCAGACGCAGAGCTGTATGATAAGCTATCTTACACTGAAATTCTGGATAAAGAATTGAAAGTGATGGACTTG | MN253317 |
| NL30 | *Vibrio communis* | GGCGGTGGTAACTTGTTCCGTGGTGCAGGTCTTGCAGAAGCGGGTATGAACCGCGTAGTAGGCGACCACATGGGTATGCTTGCAACAGTAATGAACGGTCTAGCAATGCGTGACGCACTTCACCGTGCTTATGTAAACGCTCGCGTAATGTCTGCAATTCCGCTAAAAGGTGTGTGTGACGATTACAACTGGGCAGACGCTATCCGCGAACTTCGCCAAGGCCGTGTAGTTATCTTCTCTGCAGGTACTGGTAACCCATTCTTCACGACAGATTCAGCAGCGTGTCTACGCGGTATCGAAATCGAAGCTGACGTAGTTCTAAAAGCAACGAAAGTTGATGGTGTATTTACTGCTGACCCAGTAGCAAACCCAGACGCAGAGCTGTATGATAAGCTATCTTACACTGAAATTCTGGATAAAGAATTGAAAGTAATGGACTTG | MN253318 |
| JL01 | *Vibrio communis* | GGCGGTGGTAACCTGTTCCGTGGTGCAGGTCTTGCAGAAGCGGGTATGAACCGCGTAGTAGGCGACCACATGGGTATGCTTGCAACAGTAATGAACGGTCTAGCAATGCGTGACGCACTTCACCGTGCTTATGTAAACGCTCGCGTAATGTCTGCAATTCCGCTAAAAGGTGTGTGTGACGATTACAATTGGGCAGACGCTATCCGCGAACTTCGCCAAGGCCGTGTAGTTATCTTCTCTGCAGGTACTGGTAACCCATTCTTCACGACAGATTCAGCAGCGTGTCTACGCGGCATCGAAATCGAAGCTGACGTAGTTCTAAAAGCAACGAAAGTTGATGGTGTATTTACTGCTGACCCAGTAGCAAACCCAGACGCAGAGCTGTATGATAAGCTATCTTACACTGAAATTCTGGATAAAGAATTGAAAGTAATGGACTTG | MN253319 |
| PKK08 | *Vibrio diabolicus* | GGTGGCGGTAACTTGTTCCGTGGTGCAGGCCTAGCTGAAGCTGGTATGAACCGCGTAGTTGGTGACCACATGGGTATGCTTGCTACAGTAATGAATGGCCTAGCGATGCGCGATGCTCTTCACCGTGCATACGTGAATGCGCGCGTTATGTCTGCCATTCCTCTAAAAGGCGTATGTGACGACTATAATTGGGCTGATGCGATCCGTGAGCTTCGCCAAGGCCGTGTTGTCATCTTCTCTGCTGGTACGGGTAACCCGTTCTTTACAACGGATTCTGCTGCTTGCCTACGTGGTATCGAAATTGAAGCGGATGTCGTACTAAAAGCGACGAAAGTTGATGGTGTATTTACTGCTGACCCTGTAGCAAACCCAGACGCAGAGCTGTATGATAAGCTATCTTATGCAGAAGTTCTGGATAAAGAGCTGAAAGTTATGGACTTG | MN253320 |
| NS06 | *Vibrio fluvialis* | GGTGGTGGTAACTTGTTCCGTGGTGCCGGTCTGGCAGCAGCTGGTATGAACCGCGTCGTGGGTGATCACATGGGTATGCTGGCAACAGTAATGAATGGTCTGGCCATGCGTGATGCGCTGCACCGTGCGTACGTGAACGCACGTGTGATGTCTGCGATTCCTCTGAAAGGCGTGTGTGACGATTACAACTGGGCAGATGCTATCAGCCAACTGCGCCAAGGTCGCGTGGTGATTTTCTCCGCTGGTACAGGCAACCCATTCTTTACTACCGATTCTGCAGCCTGTCTGCGTGGTATCGAAATCGAAGCAGATGTAGTTCTCAAAGCGACTAAAGTAGACGGCGTATATTCTGCAGACCCGGTAGCCAACCCAGACGCACAACTGTATGATAAGCTCGCATACAACGATGTACTTGATAAAGAATTGAAAGTGATGGATTTG | MN253321 |
| NL01 | *Vibrio furnissii* | GGTGGTGGTAACTTGTTCCGTGGCGCAGGCCTAGCCGCTGCTGGTATGAACCGTGTTGTGGGTGACCACATGGGTATGTTGGCAACAGTAATGAATGGCTTGGCAATGCGTGATGCACTGCACCGTGCTTACGTGAACGCACGTGTGATGTCTGCCATTCCTCTCAAAGGTGTGTGTGACGATTACAATTGGGCCGATGCGATTAGCCAACTACGCCAAGGTCGTGTGGTCATTTTCTCGGCGGGTACAGGCAACCCATTCTTTACAACCGATTCAGCAGCTTGTCTGCGCGGTATCGAAATTGAAGCGGACGTAGTTCTCAAAGCAACAAAAGTAGACGGCGTTTACAGTGCTGACCCGGTAGCCAACCCTGACGCACAACTGTATGATAAGCTCGCGTACAACGATGTACTTGATAAAGAATTGAAAGTGATGGATTTG | MN253322 |
| NK01 | *Vibrio furnissii* | GGTGGTGGTAACTTGTTCCGTGGCGCAGGCCTAGCCGCTGCTGGTATGAACCGTGTTGTGGGTGACCACATGGGGATGTTGGCAACAGTAATGAATGGCTTGGCAATGCGTGATGCACTGCACCGTGCTTACGTGAACGCACGTGTGATGTCTGCCATTCCTCTCAAAGGTGTGTGTGACGATTACAATTGGGCCGATGCGATTAGCCAACTACGCCAAGGTCGTGTGGTCATTTTCTCGGCGGGTACAGGCAACCCATTCTTTACAACCGATTCAGCAGCTTGTCTGCGCGGTATCGAAATTGAAGCGGACGTAGTTCTCAAAGCAACAAAAGTAGACGGCGTTTACAGTGCTGACCCGGTAGCCAACCCTGACGCACAACTGTATGATAAGCTCGCATACAACGATGTACTTGATAAAGAATTGAAAGTGATGGATTTG | MN253323 |
| NL02 | *Vibrio furnissii* | GGTGGTGGTAACTTGTTCCGTGGCGCAGGCCTAGCCGCTGCTGGTATGAACCGTGTTGTGGGTGACCACATGGGGATGTTGGCAACAGTAATGAATGGCTTGGCAATGCGTGATGCACTGCACCGTGCTTACGTGAACGCACGTGTGATGTCTGCCATTCCTCTCAAAGGTGTGTGTGACGATTACAATTGGGCCGATGCGATTAGCCAACTACGCCAAGGTCGTGTGGTCATTTTCTCGGCGGGTACAGGCAACCCATTCTTTACAACCGATTCAGCAGCTTGTCTGCGCGGTATCGAAATTGAAGCGGACGTAGTTCTCAAAGCAACAAAAGTAGACGGCGTTTACAGTGCTGACCCGGTAGCCAACCCTGACGCACAACTGTATGATAAGCTCGCATACAACGATGTACTTGATAAAGAATTGAAAGTGATGGATTTG | MN253324 |
| NS05 | *Vibrio furnissii* | GGTGGTGGTAACTTGTTCCGTGGCGCAGGCCTAGCCGCTGCTGGTATGAACCGTGTTGTGGGTGACCACATGGGGATGTTGGCAACAGTAATGAATGGCTTGGCAATGCGTGATGCACTGCACCGTGCTTATGTGAACGCACGTGTGATGTCTGCCATTCCTCTCAAAGGTGTGTGTGACGATTACAATTGGGCCGATGCGATTAGCCAACTACGCCAAGGCCGTGTGGTCATTTTCTCGGCGGGTACAGGCAACCCATTCTTTACCACCGATTCAGCAGCTTGTCTGCGCGGTATCGAAATTGAAGCGGACGTAGTTCTCAAAGCGACAAAAGTAGACGGCGTTTACAGTGCTGACCCGGTAGCCAACCCTGACGCACAACTGTATGATAAGCTCGCATACAACGATGTACTTGATAAAGAATTGAAAGTGATGGATTTG | MN253325 |
| NK05 | *Vibrio furnissii* | GGTGGTGGTAACTTGTTCCGTGGCGCAGGCCTAGCCGCTGCTGGTATGAACCGTGTTGTGGGTGACCACATGGGGATGTTGGCAACAGTAATGAATGGCTTGGCAATGCGTGATGCACTGCACCGTGCTTATGTGAACGCACGTGTGATGTCTGCCATTCCTCTCAAAGGTGTGTGTGACGATTACAATTGGGCCGATGCGATTAGCCAACTACGCCAAGGCCGTGTGGTCATTTTCTCGGCGGGTACAGGCAACCCATTCTTTACCACCGATTCAGCAGCTTGTCTGCGCGGTATCGAAATTGAAGCGGACGTAGTTCTCAAAGCGACAAAAGTAGACGGCGTTTACAGTGCTGACCCGGTAGCCAACCCTGACGCACAACTGTATGATAAGCTCGCATACAACGATGTACTTGATAAAGAATTGAAAGTGATGGATTTG | MN253326 |
| NK08 | *Vibrio furnissii* | GGTGGTGGTAACTTGTTCCGTGGCGCAGGCCTAGCCGCTGCTGGTATGAACCGTGTTGTGGGTGACCACATGGGTATGTTGGCAACAGTAATGAATGGCTTGGCAATGCGTGATGCACTGCACCGTGCTTACGTGAACGCACGTGTGATGTCTGCCATTCCTCTCAAAGGTGTGTGTGACGATTACAATTGGGCCGATGCGATTAGCCAACTACGCCAAGGTCGTGTGGTCATTTTCTCGGCGGGTACAGGCAACCCATTCTTTACAACCGATTCAGCAGCTTGTCTGCGCGGTATCGAAATTGAAGCGGACGTAGTTCTCAAAGCAACAAAAGTAGACGGCGTTTACTCAGCGGACCCGGTAGCCAACCCTGACGCACAACTGTATGATAAACTCGCATACAACGATGTACTTGATAAAGAATTGAAAGTGATGGATTTG | MN253327 |
| NK22 | *Vibrio furnissii* | GGTGGTGGTAACTTGTTCCGTGGCGCAGGCCTAGCCGCTGCTGGTATGAACCGTGTTGTGGGTGACCACATGGGGATGTTGGCAACAGTAATGAATGGCTTGGCAATGCGTGATGCACTGCACCGTGCTTACGTGAACGCACGTGTGATGTCTGCCATTCCTCTCAAAGGTGTGTGTGACGATTACAATTGGGCCGATGCGATTAGCCAACTACGCCAAGGTCGTGTGGTCATTTTCTCGGCGGGTACAGGCAACCCATTCTTTACAACCGATTCAGCAGCTTGTCTGCGCGGTATCGAAATTGAAGCGGACGTAGTTCTCAAAGCAACAAAAGTAGACGGCGTTTATAGTGCTGACCCGGTAGCCAACCCCGACGCACAACTGTATGATAAGCTCGCATACAACGATGTACTTGATAAAGAATTGAAAGTGATGGATTTG | MN253328 |
| PJK21 | *Vibrio harveyi* | GGTGGTGGTAACCTGTTCCGTGGCGCTGGTCTTGCGGAAGCTGGTATGAACCGCGTAGTAGGCGACCACATGGGTATGCTTGCAACGGTAATGAACGGTTTGGCAATGCGTGACGCACTTCACCGTGCATACGTAAACGCTCGTGTAATGTCTGCAATTCCTCTAAAAGGTGTGTGTGACGACTACAATTGGGCAGACGCTATCCGCGAACTTCGTCAAGGCCGAGTGGTAATCTTCTCTGCAGGTACTGGTAACCCATTCTTCACAACAGATTCAGCGGCGTGTCTACGTGGTATCGAAATTGAAGCTGACGTAGTTCTAAAAGCAACGAAAGTTGATGGCGTATTTACTGCAGACCCAGTAGCAAACCCAGACGCAGAGCTGTATGATAAGCTATCTTACGCCGAAGTACTGGATAAAGAACTGAAAGTAATGGACTTG | MN253329 |
| PJS28 | *Vibrio harveyi* | GGTGGTGGTAACCTGTTCCGTGGCGCTGGTCTTGCGGAAGCAGGTATGAACCGCGTAGTAGGCGACCACATGGGTATGCTTGCAACGGTAATGAACGGTTTGGCAATGCGTGACGCACTTCACCGTGCATACGTAAACGCTCGTGTAATGTCTGCAATTCCTCTAAAAGGTGTGTGTGACGACTACAATTGGGCAGACGCTATCCGCGAACTTCGTCAAGGCCGAGTGGTAATCTTCTCTGCAGGTACTGGTAACCCATTCTTCACAACAGATTCAGCGGCGTGTCTACGTGGTATCGAAATTGAAGCTGACGTAGTTCTAAAAGCAACGAAAGTTGATGGCGTATTTACTGCAGACCCAGTAGCAAACCCAGACGCAGAGCTGTATGATAAGCTATCTTACGCCGAAGTACTGGATAAAGAACTGAAAGTAATGGACTTG | MN253330 |
| KL19 | *Vibrio harveyi* | GGTGGTGGTAACCTGTTCCGTGGCGCTGGTCTTGCGGAAGCTGGTATGAACCGCGTAGTAGGCGACCACATGGGTATGCTTGCAACGGTAATGAACGGTTTGGCAATGCGTGACGCACTTCACCGTGCATACGTAAACGCTCGTGTAATGTCTGCAATTCCTCTAAAAGGTGTGTGTGACGACTACAATTGGGCAGACGCTATCCGCGAACTTCGTCAAGGCCGAGTGGTAATCTTCTCTGCAGGTACTGGTAACCCATTCTTCACAACAGATTCAGCGGCGTGTCTACGTGGTATCGAAATTGAAGCTGACGTAGTTCTAAAAGCAACGAAAGTTGATGGCGTATTTACTGCAGACCCAGTAGCAAACCCAGACGCAGAGCTGTATGATAAGCTATCTTACGCAGAAGTTCTGGATAAAGAGCTGAAAGTAATGGACTTG | MN253331 |
| KS22 | *Vibrio harveyi* | GGTGGTGGTAACCTGTTCCGTGGCGCTGGTCTTGCGGAAGCTGGTATGAACCGCGTAGTAGGCGACCACATGGGTATGCTTGCAACGGTAATGAACGGTTTGGCAATGCGTGACGCACTTCACCGTGCATACGTAAACGCTCGTGTAATGTCTGCAATTCCTCTAAAAGGTGTGTGTGACGACTACAATTGGGCAGACGCTATCCGCGAACTTCGTCAAGGCCGAGTGGTAATCTTCTCTGCAGGTACTGGTAACCCATTCTTCACAACAGATTCAGCGGCGTGTCTACGTGGTATCGAAATTGAAGCTGACGTAGTTCTAAAAGCAACGAAAGTTGATGGTGTATTTACTGCAGACCCAGTAGCAAACCCAGACGCAGAGCTGTATGATAAGCTATCTTACGCAGAAGTTCTGGATAAAGAGCTGAAAGTAATGGACTTG | MN253332 |
| JS11 | *Vibrio harveyi* | GGTGGTGGTAACCTGTTCCGTGGCGCTGGTCTTGCGGAAGCTGGTATGAACCGCGTAGTAGGCGACCACATGGGTATGCTTGCAACGGTAATGAACGGTTTGGCAATGCGTGACGCACTTCACCGTGCATACGTAAACGCTCGTGTAATGTCTGCAATTCCTCTAAAAGGTGTGTGTGACGACTACAATTGGGCAGACGCTATCCGCGAACTTCGTCAAGGCCGAGTGGTAATCTTCTCTGCAGGTACTGGTAACCCATTCTTCACAACAGATTCAGCGGCGTGTCTACGTGGTATCGAAATTGAAGCTGACGTAGTTCTAAAAGCAACGAAAGTTGATGGCGTATTTACTGCAGACCCAGTAGCAAACCCAGACGCAGAGCTGTATGATAAGCTATCTTACGCAGAAGTTCTGGATAAAGAGCTGAAAGTAATGGACTTG | MN253333 |
| LL01 | *Vibrio mytili* | GGTGGTGGTAACCTTTTCCGTGGCGCAGGCCTTGCTGAAGCGGGCATGAACCGTGTTGTGGGCGACCACATGGGTATGCTAGCAACAGTAATGAACGGCCTAGCTATGCGTGACGCACTGCACCGTGCATACGTAAATGCGCGTGTAATGTCTGCAATTCCTCTGAAAGGCGTATGTGACGACTACAATTGGGCTGATGCTATTCGCGAACTTCGCCAAGGTCGTGTTGTTATCTTCTCTGCTGGTACAGGTAACCCATTCTTCACAACAGATTCAGCGGCTTGTCTACGTGGCATTGAAATCGAAGCTGACGTAGTTCTAAAAGCGACAAAAGTAGACGGTGTATTTACTGCTGACCCTGTAGCAAACCCAGACGCAGAGCTGTATGATAAGCTGTCTTACGCAGAAGTTCTTGATAAAGAACTAAAAGTAATGGATTTG | MN253334 |
| LL04 | *Vibrio mytili* | GGTGGTGGTAACCTTTTCCGTGGTGCAGGCCTTGCTGAAGCGGGGATGAACCGCGTTGTAGGTGACCACATGGGTATGCTAGCGACAGTAATGAACGGCCTAGCTATGCGTGATGCACTACACCGTGCATACGTAAATGCTCGCGTAATGTCTGCAATTCCTCTAAAAGGCGTATGTGACGACTACAATTGGGCTGATGCAATTCGCGAACTTCGTCAAGGCCGTGTTGTTATCTTCTCAGCTGGTACGGGTAACCCATTCTTCACAACAGATTCTGCAGCGTGTCTACGTGGTATTGAGATCGAAGCTGACGTTGTTCTCAAAGCAACTAAGGTGGATGGTGTATTTACTGCTGACCCAGTAGCTAACCCAGACGCAGAGCTGTATGATAAGCTGTCTTACGCAGAAGTTCTTGATAAAGAACTAAAAGTAATGGATTTG | MN253335 |
| LK10 | *Vibrio mytili* | GGTGGTGGTAACCTTTTCCGTGGTGCAGGTCTTGCTGAAGCAGGTATGAACCGTGTTGTGGGCGACCACATGGGTATGCTTGCAACAGTAATGAACGGCCTAGCTATGCGTGATGCACTGCACCGTGCATATGTAAACGCTCGCGTAATGTCTGCAATTCCTCTGAAAGGCGTATGTGACGACTACAATTGGGCTGATGCTATTCGCGAACTTCGCCAAGGCCGTGTTGTTATCTTCTCTGCTGGTACTGGTAACCCATTCTTCACTACTGATTCAGCGGCTTGTCTACGTGGCATTGAAATTGAAGCTGACGTAGTTCTAAAAGCGACAAAAGTAGACGGTGTATTTACTGCTGACCCTGTAGCAAACCCAGACGCAGAGCTGTATGATAAGCTGTCTTACGCTGAAGTTCTTGATAAAGAACTAAAAGTAATGGATTTG | MN253336 |
| LK11 | *Vibrio mytili* | GGTGGTGGTAACCTTTTCCGTGGCGCAGGCCTTGCTGAAGCGGGCATGAACCGTGTTGTGGGCGACCACATGGGTATGCTAGCAACAGTAATGAACGGCCTAGCTATGCGTGACGCACTGCACCGTGCATACGTAAATGCGCGTGTAATGTCTGCAATTCCTCTGAAAGGCGTATGTGACGACTACAATTGGGCTGATGCTATTCGCGAACTTCGCCAAGGTCGTGTTGTTATCTTCTCTGCTGGTACAGGTAACCCATTCTTCACAACAGATTCAGCGGCTTGTCTACGTGGCATTGAAATTGAAGCTGACGTAGTTCTAAAAGCGACAAAAGTAGACGGTGTATTTACTGCTGACCCTGTAGCAAACCCAGACGCAGAGCTGTATGATAAGCTGTCTTACGCTGAAGTTCTTGATAAAGAGCTAAAAGTAATGGATTTG | MN253337 |
| LS16 | *Vibrio mytili* | GGTGGTGGTAACCTTTTCCGTGGTGCAGGTCTTGCAGAAGCTGGAATGAACCGTGTAGTGGGCGACCACATGGGTATGCTAGCAACAGTAATGAACGGCCTAGCTATGCGTGACGCACTGCACCGTGCTTACGTAAATGCTCGCGTAATGTCTGCAATTCCTCTGAAAGGTGTGTGTGACGACTACAATTGGGCTGATGCTATTCGCGAACTTCGCCAAGGTCGTGTAGTTATCTTCTCTGCTGGTACTGGTAACCCATTCTTCACAACAGATTCAGCGGCTTGTCTACGTGGCATTGAAATTGAAGCTGACGTAGTTCTAAAAGCGACAAAAGTTGACGGTGTATTTACTGCTGACCCTGTAGCAAACCCAGACGCAGAGCTGTATGATAAGCTGTCTTACGCTGAAGTTCTTGATAAAGAACTAAAAGTAATGGATTTG | MN253338 |
| LK22 | *Vibrio mytili* | GGTGGTGGTAACCTTTTCCGTGGTGCAGGCCTTGCTGAAGCGGGTATGAACCGCGTTGTAGGTGACCACATGGGCATGCTAGCGACAGTAATGAACGGCCTAGCTATGCGTGATGCACTACACCGTGCATACGTAAATGCTCGCGTAATGTCTGCAATTCCTCTAAAAGGCGTATGTGACGACTACAATTGGGCTGATGCAATTCGCGAACTTCGTCAAGGCCGTGTTGTTATCTTCTCAGCTGGTACGGGTAACCCATTCTTCACAACAGATTCTGCAGCGTGTCTACGTGGTATTGAGATCGAAGCGGACGTTGTTCTCAAAGCAACTAAGGTGGATGGTGTATTTACTGCTGACCCAGTAGCAAACCCAGACGCAGAGCTGTATGATAAACTTAGTTACGCAGAAGTTCTTGATAAAGAGCTAAAAGTAATGGATTTG | MN253339 |
| LS23 | *Vibrio mytili* | GGTGGTGGTAACCTTTTCCGTGGTGCAGGCCTTGCTGAAGCGGGGATGAACCGCGTTGTAGGTGACCACATGGGTATGCTAGCGACAGTAATGAACGGCCTAGCTATGCGTGATGCACTACACCGTGCATACGTAAATGCTCGCGTAATGTCTGCAATTCCTCTAAAAGGCGTATGTGACGACTACAATTGGGCTGATGCAATTCGCGAACTTCGTCAAGGCCGTGTTGTTATCTTCTCAGCTGGTACGGGTAACCCATTCTTCACAACAGATTCTGCAGCGTGTCTACGTGGTATTGAGATCGAAGCTGACGTTGTTCTCAAAGCAACTAAGGTGGATGGTGTATTTACTGCTGACCCAGTAGCAAACCCAGACGCAGAGCTGTATGATAAACTTAGTTACGCAGAAGTTCTTGATAAAGAGCTAAAAGTAATGGATTTG | MN253340 |
| KS28 | *Vibrio mytili* | GGTGGTGGTAACCTTTTCCGTGGTGCAGGCCTTGCTGAAGCTGGTATGAACCGTGTTGTGGGCGACCATATGGGTATGCTTGCTACAGTAATGAACGGTTTAGCAATGCGTGATGCTCTGCACCGTGCTTACGTAAATGCTCGCGTGATGTCTGCAATTCCTCTAAAAGGCGTATGTGACGACTACAATTGGGCTGATGCGATTCGCGAACTTCGCCAAGGCCGCGTAGTTATTTTCTCTGCAGGTACTGGTAACCCATTCTTCACTACTGATTCTGCTGCTTGCTTACGTGGTATCGAAATCGAAGCTGATGTGGTTCTAAAAGCAACCAAAGTGGATGGTGTATTTACCGCAGACCCAGTAGCAAACCCTGACGCAGAGCTGTATGATAAGCTGAGCTACGCTGAAGTTCTTGATAAAGAACTAAAAGTGATGGATTTA | MN253341 |
| PKGK01 | *Vibrio parahaemolyticus* | GGTGGCGGTAACCTGTTCCGTGGCGCTGGTCTTGCAGAAGCTGGTATGAACCGTGTAGTGGGTGACCACATGGGTATGCTTGCTACAGTAATGAACGGCCTTGCGATGCGTGACGCTCTTCACCGTGCATACGTAAACGCGCGCGTAATGTCTGCTATTCCTCTTAAAGGCGTATGTGACGACTACAATTGGGCTGATGCAATTCGCGAACTTCGTCAAGGCCGCGTTGTAATCTTCTCAGCAGGTACTGGTAACCCATTCTTCACAACAGACTCTGCTGCGTGTCTACGTGGTATCGAAATCGAAGCTGACGTAGTTCTAAAAGCAACGAAAGTTGATGGCGTATTTACTGCTGACCCAGTAGCAAACCCAGACGCAGAGCTGTATGATAAGCTATCTTACGCAGAAGTTCTGGATAAAGAGCTTAAAGTAATGGATTTG | MN253342 |
| PKGS11 | *Vibrio parahaemolyticus* | GGTGGCGGTAACCTGTTCCGTGGCGCTGGTCTTGCAGAAGCTGGTATGAACCGTGTAGTGGGTGACCACATGGGTATGCTTGCTACAGTAATGAACGGCCTTGCGATGCGTGACGCTCTTCACCGTGCATACGTAAATGCGCGCGTAATGTCTGCAATTCCTCTTAAAGGCGTATGTGATGACTACAATTGGGCTGATGCAATTCGCGAACTTCGTCAAGGCCGCGTTGTAATCTTCTCAGCAGGTACTGGTAACCCATTCTTCACAACAGACTCTGCTGCGTGTCTACGTGGTATCGAAATCGAAGCTGACGTAGTTCTAAAAGCAACGAAAGTTGATGGCGTATTTACTGCTGACCCAGTAGCAAACCCAGACGCAGAGCTGTATGATAAGCTATCTTACGCAGAAGTTCTGGATAAAGAGCTTAAAGTAATGGATTTG | MN253343 |
| PKGS20 | *Vibrio parahaemolyticus* | GGTGGCGGTAACCTGTTCCGTGGCGCTGGTCTTGCAGAAGCTGGTATGAACCGTGTAGTGGGTGACCACATGGGTATGCTTGCTACAGTAATGAACGGCCTTGCGATGCGTGACGCTCTTCACCGTGCATACGTAAACGCGCGCGTAATGTCTGCAATTCCTCTTAAAGGCGTATGTGACGACTACAATTGGGCTGATGCAATTCGCGAACTTCGTCAAGGCCGCGTTGTAATCTTCTCAGCAGGTACTGGTAACCCATTCTTCACAACAGACTCTGCTGCGTGTCTACGTGGTATCGAAATCGAAGCTGACGTAGTTCTAAAAGCAACGAAAGTTGATGGCGTATTTACTGCTGACCCAGTAGCAAACCCAGACGCAGAGCTGTATGATAAGCTATCTTACGCAGAAGTTCTGGATAAAGAGCTTAAAGTAATGGATTTG | MN253344 |
| PKGS23 | *Vibrio parahaemolyticus* | GGTGGCGGTAACCTGTTCCGTGGCGCTGGTCTTGCAGAAGCTGGTATGAACCGTGTAGTGGGTGACCACATGGGTATGCTTGCTACAGTAATGAACGGCCTTGCGATGCGTGACGCTCTTCACCGTGCATACGTAAACGCGCGCGTAATGTCTGCAATTCCTCTTAAAGGCGTATGTGACGACTACAATTGGGCTGATGCAATTCGCGAACTTCGTCAAGGCCGCGTTGTAATCTTCTCAGCAGGTACTGGTAACCCATTCTTCACAACAGACTCTGCTGCGTGTCTACGTGGTATCGAAATCGAAGCTGACGTAGTTCTAAAAGCAACGAAAGTTGATGGCGTATTTACTGCTGACCCAGTAGCAAACCCAGACGCAGAGCTGTATGATAAGCTATCTTACGCAGAAGTTCTGGATAAAGAGCTTAAAGTAATGGATTTG | MN253345 |
| PKGK24 | *Vibrio parahaemolyticus* | GGTGGCGGTAACCTGTTCCGTGGCGCTGGTCTTGCAGAAGCTGGTATGAACCGTGTAGTGGGTGACCACATGGGTATGCTTGCTACAGTAATGAACGGTCTTGCAATGCGTGACGCTCTTCACCGTGCATACGTAAATGCGCGCGTAATGTCTGCAATTCCTCTTAAAGGCGTATGTGACGACTACAATTGGGCTGATGCAATTCGCGAACTTCGTCAAGGCCGCGTTGTAATCTTCTCAGCAGGTACTGGTAACCCATTCTTCACAACAGACTCTGCTGCGTGTCTACGTGGTATCGAAATCGAAGCTGACGTAGTTCTAAAAGCAACGAAAGTTGATGGCGTATTTACTGCTGACCCAGTAGCAAACCCAGACGCAGAGCTGTATGATAAGCTATCTTACGCAGAAGTTCTGGATAAAGAGCTTAAAGTAATGGATTTG | MN253346 |
| KK03 | *Vibrio parahaemolyticus* | GGTGGCGGTAACCTGTTCCGTGGCGCTGGTCTTGCTGAAGCTGGTATGAACCGTGTAGTGGGTGACCACATGGGTATGCTTGCTACAGTAATGAACGGCCTAGCGATGCGTGACGCTCTTCACCGTGCATACGTAAATGCGCGCGTAATGTCTGCAATTCCTCTTAAAGGCGTATGTGACGACTACAATTGGGCTGATGCAATTCGCGAACTTCGTCAAGGCCGCGTTGTAATCTTCTCAGCAGGTACTGGTAACCCATTCTTCACAACAGACTCTGCTGCGTGTCTACGTGGTATCGAAATCGAAGCTGACGTAGTTCTAAAAGCAACGAAAGTTGATGGCGTATTTACTGCTGACCCAGTAGCAAACCCAGACGCAGAACTGTATGATAAGCTATCTTACGCAGAAGTTCTGGATAAAGAACTTAAAGTAATGGATTTG | MN253347 |
| KL04 | *Vibrio parahaemolyticus* | GGTGGCGGTAACCTGTTCCGTGGCGCTGGTCTTGCAGAAGCTGGTATGAACCGTGTAGTGGGTGACCACATGGGTATGCTTGCTACAGTAATGAACGGCCTTGCGATGCGTGACGCTCTTCACCGTGCATACGTAAATGCGCGCGTAATGTCTGCAATTCCTCTTAAAGGCGTATGTGACGACTACAATTGGGCTGATGCAATTCGCGAACTTCGTCAAGGCCGCGTTGTAATCTTCTCAGCAGGTACTGGTAACCCATTCTTCACAACAGACTCTGCTGCGTGTCTACGTGGTATCGAAATCGAAGCTGACGTAGTTCTAAAAGCAACGAAAGTTGATGGCGTATTTACTGCTGACCCAGTAGCAAACCCAGACGCAGAGCTGTATGATAAGCTATCTTACGCAGAAGTTCTGGATAAAGAGCTTAAAGTAATGGATTTG | MN253348 |
| KK10 | *Vibrio parahaemolyticus* | GGTGGCGGTAACCTGTTCCGTGGCGCTGGTCTTGCAGAAGCTGGTATGAACCGTGTAGTGGGTGACCACATGGGTATGCTTGCTACAGTAATGAACGGCCTTGCGATGCGTGACGCTCTTCACCGTGCATACGTAAATGCGCGCGTAATGTCTGCAATTCCTCTTAAAGGCGTATGTGACGACTACAATTGGGCTGATGCAATTCGCGAACTTCGTCAAGGCCGCGTTGTAATCTTCTCAGCAGGTACTGGTAACCCATTCTTCACAACAGACTCTGCTGCGTGTCTACGTGGTATCGAAATCGAAGCTGACGTAGTTCTAAAAGCAACGAAAGTTGATGGCGTATTTACTGCTGACCCAGTAGCAAACCCAGACGCAGAGCTGTATGATAAGCTATCTTACGCAGAAGTTCTGGATAAAGAACTTAAAGTAATGGATTTG | MN253349 |
| KL11 | *Vibrio parahaemolyticus* | GGTGGCGGTAACTTGTTCCGTGGCGCTGGTCTTGCTGAAGCTGGTATGAACCGTGTAGTGGGTGACCACATGGGTATGCTTGCTACAGTAATGAACGGCCTTGCGATGCGTGACGCTCTTCACCGTGCATATGTAAACGCGCGCGTAATGTCTGCAATTCCTCTTAAAGGCGTATGTGACGACTACAATTGGGCTGATGCAATTCGCGAACTTCGTCAAGGCCGCGTTGTAATCTTCTCAGCAGGTACTGGTAACCCATTCTTCACAACAGACTCTGCTGCTTGTCTACGTGGTATCGAAATCGAAGCTGACGTAGTTCTAAAAGCAACGAAAGTTGATGGCGTATTTACTGCTGACCCAGTAGCAAACCCAGACGCAGAGCTGTATGATAAGCTATCTTACGCAGAAGTTCTGGATAAAGAACTTAAAGTAATGGATTTG | MN253350 |
| KS12 | *Vibrio parahaemolyticus* | GGTGGCGGTAACCTGTTCCGTGGCGCTGGTCTTGCAGAAGCTGGTATGAACCGTGTAGTGGGTGACCACATGGGTATGCTTGCTACAGTAATGAACGGCCTTGCGATGCGTGACGCTCTTCACCGTGCATACGTAAATGCTCGCGTAATGTCTGCAATTCCTCTTAAAGGCGTATGTGACGACTACAATTGGGCTGATGCTATTCGCGAACTTCGTCAAGGCCGCGTTGTAATCTTCTCAGCAGGTACTGGTAACCCATTCTTCACAACAGACTCTGCTGCGTGTCTACGTGGTATCGAAATCGAAGCTGACGTAGTTCTAAAAGCAACGAAAGTTGATGGCGTATTTACTGCTGACCCAGTAGCAAACCCAGACGCAGAACTGTATGATAAGCTATCTTACGCAGAAGTTCTGGATAAAGAACTTAAAGTAATGGATTTG | MN253351 |
| KL13 | *Vibrio parahaemolyticus* | GGTGGTGGTAACTTGTTCCGTGGTGCTGGTCTTGCAGAAGCTGGTATGAACCGCGTAGTGGGTGACCACATGGGTATGCTTGCTACAGTAATGAACGGTCTTGCGATGCGTGACGCTCTTCATCGTGCATACGTAAATGCTCGCGTAATGTCTGCAATTCCTCTTAAAGGCGTATGTGACGACTACAATTGGGCTGATGCTATTCGCGAACTTCGTCAAGGCCGCGTTGTAATCTTCTCAGCAGGTACTGGTAACCCATTCTTCACAACAGACTCTGCTGCGTGTCTACGTGGTATCGAAATCGAAGCTGACGTAGTTCTAAAAGCAACGAAAGTTGATGGCGTATTTACTGCTGACCCAGTAGCAAACCCAGACGCAGAACTGTATGATAAGCTATCTTACGCAGAAGTTCTGGATAAAGAACTTAAAGTAATGGATTTG | MN253352 |
| KL14 | *Vibrio parahaemolyticus* | GGTGGTGGTAACTTGTTCCGTGGTGCTGGTCTTGCAGAAGCTGGTATGAACCGCGTAGTGGGTGACCACATGGGTATGCTTGCTACAGTAATGAACGGTCTTGCGATGCGTGATGCTCTTCACCGTGCATACGTAAATGCTCGCGTAATGTCTGCAATTCCTCTTAAAGGCGTATGTGACGACTACAATTGGGCTGATGCTATTCGCGAACTTCGTCAAGGCCGCGTTGTAATCTTCTCAGCAGGTACTGGTAACCCATTCTTCACAACAGACTCTGCTGCGTGTCTACGTGGTATCGAAATCGAAGCTGACGTAGTTCTAAAAGCAACGAAAGTTGATGGCGTATTTACTGCTGACCCAGTAGCAAACCCAGACGCAGAACTGTATGATAAGCTATCTTACGCAGAAGTTCTGGATAAAGAACTTAAAGTAATGGATTTG | MN253353 |
| KS14 | *Vibrio parahaemolyticus* | GGTGGCGGTAACCTGTTCCGTGGTGCTGGTCTTGCAGAAGCTGGTATGAACCGTGTAGTGGGTGACCACATGGGTATGCTTGCTACAGTAATGAACGGCCTAGCGATGCGTGATGCTCTTCACCGTGCATACGTAAATGCTCGCGTAATGTCTGCAATTCCTCTTAAAGGCGTATGTGACGACTACAATTGGGCTGATGCTATTCGCGAACTTCGCCAAGGCCGCGTTGTAATCTTCTCTGCAGGTACTGGTAACCCATTCTTCACTACAGATTCTGCTGCGTGTCTACGTGGTATCGAAATCGAAGCTGACGTAGTTCTAAAAGCAACGAAAGTTGATGGCGTATTTACTGCTGACCCAGTAGCAAACCCAGACGCAGAGCTGTATGATAAGCTATCTTACGCTGAAGTTCTGGATAAAGAACTTAAAGTAATGGACTTG | MN253354 |
| KK15 | *Vibrio parahaemolyticus* | GGTGGTGGTAACTTGTTCCGTGGTGCAGGTCTTGCAGAAGCTGGTATGAACCGCGTAGTGGGTGACCACATGGGTATGCTTGCTACAGTAATGAACGGTCTTGCGATGCGTGATGCTCTTCATCGTGCATACGTAAATGCTCGCGTAATGTCTGCAATTCCTCTTAAAGGCGTATGTGACGACTACAATTGGGCTGATGCTATTCGCGAACTTCGCCAAGGCCGCGTTGTAATCTTCTCAGCAGGTACTGGTAACCCATTCTTCACAACAGACTCTGCTGCGTGTCTACGTGGTATCGAAATCGAAGCTGACGTAGTTCTAAAAGCAACGAAAGTTGATGGCGTATTTACTGCTGACCCAGTAGCAAACCCAGACGCAGAACTGTATGATAAGCTATCTTACGCAGAAGTTCTGGATAAAGAACTTAAAGTAATGGATTTG | MN253355 |
| KS16 | *Vibrio parahaemolyticus* | GGTGGCGGTAACCTGTTCCGTGGCGCTGGTCTTGCTGAAGCTGGTATGAACCGTGTAGTGGGTGACCACATGGGTATGCTTGCTACAGTAATGAACGGCCTTGCGATGCGTGATGCTCTTCACCGTGCATACGTAAATGCTCGCGTAATGTCTGCAATTCCTCTTAAAGGCGTATGTGACGACTACAATTGGGCTGATGCTATTCGCGAACTTCGCCAAGGCCGCGTTGTAATCTTCTCAGCAGGTACTGGTAACCCATTCTTCACAACAGACTCTGCTGCGTGTCTACGTGGTATCGAAATCGAAGCTGACGTAGTTCTAAAAGCAACGAAAGTTGATGGCGTATTTACTGCTGACCCAGTAGCAAACCCAGACGCAGAACTGTATGATAAGCTATCTTACGCAGAAGTTCTGGATAAAGAACTTAAAGTAATGGATTTG | MN253356 |
| KL17 | *Vibrio parahaemolyticus* | GGTGGCGGTAACCTATTCCGTGGCGCTGGTCTTGCAGAAGCTGGTATGAACCGTGTAGTGGGTGACCACATGGGGATGCTTGCTACAGTAATGAACGGCCTTGCGATGCGTGACGCTCTTCACCGTGCATACGTAAATGCGCGCGTAATGTCTGCAATTCCTCTTAAAGGCGTATGTGATGACTACAATTGGGCTGATGCAATTCGCGAACTTCGTCAAGGCCGCGTTGTAATCTTCTCAGCAGGTACTGGTAACCCATTCTTCACAACAGACTCTGCTGCGTGTCTACGTGGTATCGAAATCGAAGCTGACGTAGTTCTAAAAGCAACGAAAGTTGATGGCGTATTTACTGCTGACCCAGTAGCAAACCCAGACGCAGAGCTGTATGATAAGCTATCTTACGCAGAAGTTCTGGATAAAGAGCTTAAAGTAATGGATTTG | MN253357 |
| KK17 | *Vibrio parahaemolyticus* | GGTGGCGGTAACCTGTTCCGTGGCGCTGGTCTTGCTGAAGCTGGTATGAACCGTGTAGTGGGTGACCACATGGGTATGCTTGCTACAGTAATGAATGGCCTTGCGATGCGTGATGCTCTTCACCGTGCATACGTAAATGCTCGCGTAATGTCTGCAATTCCTCTTAAAGGCGTATGTGACGACTACAATTGGGCTGATGCTATTCGCGAACTTCGCCAAGGCCGCGTTGTAATCTTCTCAGCAGGTACTGGTAACCCATTCTTCACAACAGATTCTGCTGCTTGTCTACGTGGTATCGAAATCGAAGCTGACGTAGTTCTAAAAGCAACAAAAGTTGATGGCGTATTTACTGCTGACCCAGTAGCAAACCCAGACGCAGAACTGTATGATAAGCTATCTTACGCTGAAGTTCTGGATAAAGAACTTAAAGTAATGGATTTG | MN253358 |
| KL18 | *Vibrio parahaemolyticus* | GGTGGCGGTAACCTGTTCCGTGGCGCTGGTCTTGCTGAAGCTGGTATGAACCGTGTAGTGGGTGACCACATGGGTATGCTTGCTACAGTAATGAACGGCCTTGCGATGCGTGACGCTCTTCACCGTGCATACGTAAATGCGCGCGTAATGTCTGCAATTCCTCTTAAAGGCGTATGTGACGACTACAATTGGGCTGATGCAATTCGCGAACTTCGTCAAGGCCGCGTTGTAATCTTCTCAGCAGGTACTGGTAACCCATTCTTCACAACAGACTCTGCTGCGTGTCTACGTGGTATCGAAATCGAAGCTGACGTAGTTCTAAAAGCAACGAAAGTTGATGGCGTATTTACTGCTGACCCAGTAGCAAACCCAGACGCAGAGCTGTATGATAAGCTATCTTACGCAGAAGTTCTGGATAAAGAGCTTAAAGTAATGGATTTG | MN253359 |
| KS20 | *Vibrio parahaemolyticus* | GGTGGCGGTAACCTGTTCCGTGGCGCTGGTCTTGCTGAAGCTGGTATGAACCGTGTAGTGGGTGACCACATGGGTATGCTTGCTACAGTAATGAACGGCCTTGCGATGCGTGACGCTCTTCACCGTGCATACGTAAATGCTCGCGTAATGTCTGCAATTCCTCTTAAAGGCGTATGTGACGACTACAATTGGGCTGATGCAATTCGCGAACTTCGCCAAGGCCGCGTTGTAATCTTCTCAGCAGGTACTGGTAACCCATTCTTCACAACAGACTCTGCTGCTTGTCTACGTGGTATCGAAATCGAAGCTGACGTAGTTCTAAAAGCAACGAAAGTTGATGGCGTATTTACTGCTGACCCAGTAGCAAACCCAGACGCAGAACTGTATGATAAGCTATCTTACGCAGAAGTTCTGGATAAAGAACTTAAAGTAATGGATTTG | MN253360 |
| KL21 | *Vibrio parahaemolyticus* | GGTGGCGGTAACCTGTTCCGTGGCGCTGGTCTTGCTGAAGCTGGTATGAACCGTGTAGTTGGTGACCACATGGGTATGCTTGCTACAGTAATGAACGGCCTTGCGATGCGTGACGCTCTTCACCGTGCATATGTAAATGCGCGCGTAATGTCTGCAATTCCTCTAAAAGGCGTATGTGACGACTACAATTGGGCTGATGCAATTCGCGAACTTCGTCAAGGCCGAGTTGTAATCTTCTCAGCAGGTACTGGTAACCCATTCTTCACAACAGACTCTGCTGCTTGTCTACGTGGTATCGAAATCGAAGCTGACGTAGTTCTAAAAGCAACGAAAGTTGATGGCGTATTTACTGCTGACCCCGTAGCAAACCCAGACGCAGAGCTGTATGATAAGCTATCTTACGCAGAAGTTCTGGATAAAGAACTTAAAGTAATGGATTTG | MN253361 |
| KK21 | *Vibrio parahaemolyticus* | GGCGGTGGTAACCTGTTCCGTGGTGCTGGTCTTGCGGAAGCGGGTATGAACCGTGTAGTAGGCGACCACATGGGTATGCTTGCAACGGTAATGAACGGTCTAGCAATGCGTGATGCACTTCACCGTGCTTACGTAAACGCTCGTGTAATGTCTGCAATTCCTCTAAAAGGCGTGTGTGACGACTACAATTGGGCAGACGCTATCCGCGAACTTCGCCAAGGCCGTGTAGTAATCTTCTCTGCAGGTACTGGTAACCCATTCTTCACTACAGATTCAGCAGCGTGTCTACGCGGTATCGAAATCGAAGCTGACGTAGTGCTAAAAGCAACGAAAGTTGATGGCGTATTTACTGCTGACCCAGTAGCAAACCCAGACGCAGAGCTGTATGATAAGCTATCTTACGCTGAAGTTCTGGATAAAGAACTGAAAGTAATGGACTTG | MN253362 |
| KS25 | *Vibrio parahaemolyticus* | GGTGGCGGTAACCTGTTCCGTGGCGCTGGTCTAGCAGAAGCTGGTATGAACCGCGTAGTGGGTGACCACATGGGTATGCTTGCTACAGTAATGAACGGCCTTGCGATGCGTGACGCTCTTCACCGTGCATATGTAAATGCGCGCGTAATGTCTGCAATTCCTCTAAAAGGCGTATGTGACGACTACAATTGGGCTGATGCAATTCGCGAACTTCGTCAAGGCCGAGTTGTAATCTTCTCAGCAGGTACTGGTAACCCATTCTTCACAACAGACTCTGCTGCTTGTCTACGTGGTATCGAAATTGAAGCTGACGTAGTTCTAAAAGCAACGAAAGTTGATGGCGTATTTACTGCTGACCCAGTAGCAAACCCAGACGCAGAGCTGTATGATAAGCTATCTTACGCAGAAGTTCTGGATAAAGAGCTTAAAGTAATGGATTTG | MN253363 |
| KK25 | *Vibrio parahaemolyticus* | GGTGGCGGTAACCTATTCCGTGGCGCTGGTCTTGCAGAAGCTGGTATGAACCGTGTAGTGGGTGACCACATGGGGATGCTTGCTACAGTAATGAACGGCCTTGCGATGCGTGACGCTCTTCACCGTGCATACGTAAATGCGCGCGTAATGTCTGCAATTCCTCTTAAAGGCGTATGTGATGACTACAATTGGGCTGATGCAATTCGCGAACTTCGTCAAGGCCGCGTTGTAATCTTCTCAGCAGGTACTGGTAACCCATTCTTCACAACAGACTCTGCTGCGTGTCTACGTGGTATCGAAATCGAAGCTGACGTAGTTCTAAAAGCAACGAAAGTTGATGGCGTATTTACTGCTGACCCAGTAGCAAACCCAGACGCAGAGCTGTATGATAAGCTATCTTACGCAGAAGTTCTGGATAAAGAGCTTAAAGTAATGGATTTG | MN253364 |
| KK26 | *Vibrio parahaemolyticus* | GGTGGCGGTAACCTATTCCGTGGCGCTGGTCTTGCAGAAGCTGGTATGAACCGTGTAGTGGGTGACCACATGGGGATGCTTGCTACAGTAATGAACGGCCTTGCGATGCGTGACGCTCTTCACCGTGCATACGTAAATGCGCGCGTAATGTCTGCAATTCCTCTTAAAGGCGTATGTGATGACTACAATTGGGCTGATGCAATTCGCGAACTTCGTCAAGGCCGCGTTGTAATCTTCTCAGCAGGTACTGGTAACCCATTCTTCACAACAGACTCTGCTGCGTGTCTACGTGGTATCGAAATCGAAGCTGACGTAGTTCTAAAAGCAACGAAAGTTGATGGCGTATTTACTGCTGACCCAGTAGCAAACCCAGACGCAGAGCTGTATGATAAGCTATCTTACGCAGAAGTTCTGGATAAAGAGCTTAAAGTAATGGATTTG | MN253365 |
| BS01 | *Vibrio parahaemolyticus* | GGTGGCGGTAACCTGTTCCGTGGCGCTGGTCTTGCAGAAGCTGGTATGAACCGTGTAGTGGGTGACCACATGGGTATGCTTGCTACAGTAATGAACGGCCTTGCGATGCGTGACGCTCTTCACCGTGCATACGTAAACGCGCGCGTAATGTCTGCAATTCCTCTTAAAGGCGTATGTGACGACTACAATTGGGCTGATGCAATTCGCGAACTTCGTCAAGGCCGCGTTGTAATCTTCTCAGCAGGTACTGGTAACCCATTCTTCACAACAGACTCTGCTGCGTGTCTACGTGGTATCGAAATCGAAGCTGACGTAGTTCTAAAAGCAACGAAAGTTGATGGCGTATTTACTGCTGACCCAGTAGCAAACCCAGACGCAGAGCTGTATGATAAGCTATCTTACGCAGAAGTTCTGGATAAAGAGCTTAAAGTAATGGATTTG | MN253366 |
| BL04 | *Vibrio parahaemolyticus* | GGTGGCGGTAACCTGTTCCGTGGCGCTGGTCTTGCAGAAGCTGGTATGAACCGTGTAGTGGGTGACCACATGGGTATGCTTGCTACAGTAATGAACGGCCTTGCGATGCGTGACGCTCTTCACCGTGCATACGTAAATGCGCGCGTAATGTCTGCAATTCCTCTTAAAGGCGTATGTGATGACTACAATTGGGCTGATGCAATTCGCGAACTTCGTCAAGGCCGCGTTGTAATCTTCTCAGCAGGTACTGGTAACCCATTCTTCACAACAGACTCTGCTGCGTGTCTACGTGGTATCGAAATCGAAGCTGACGTAGTTCTAAAAGCAACGAAAGTTGATGGCGTATTTACTGCTGACCCAGTAGCAAACCCAGACGCAGAGCTGTATGATAAGCTATCTTACGCAGAAGTTCTGGATAAAGAGCTTAAAGTAATGGATTTG | MN253367 |
| BL05 | *Vibrio parahaemolyticus* | GGTGGCGGTAACCTGTTCCGTGGCGCTGGTCTTGCAGAAGCTGGTATGAACCGTGTAGTGGGTGACCACATGGGTATGCTTGCTACAGTAATGAACGGCCTTGCGATGCGTGACGCTCTTCACCGTGCATACGTAAATGCGCGCGTAATGTCTGCAATCCCTCTTAAAGGCGTATGTGACGATTACAATTGGGCTGATGCAATTCGCGAACTTCGTCAAGGCCGCGTTGTAATCTTCTCAGCAGGTACTGGTAACCCATTCTTCACAACAGACTCTGCTGCGTGTCTACGTGGTATTGAAATCGAAGCTGACGTAGTTCTAAAAGCAACGAAAGTTGATGGCGTATTTACTGCTGACCCAGTAGCAAACCCAGACGCAGAGCTGTATGATAAGCTATCTTACGCAGAAGTTCTGGATAAAGAGCTTAAAGTAATGGATTTG | MN253368 |
| BL10 | *Vibrio parahaemolyticus* | GGTGGCGGTAACCTGTTCCGAGGCGCTGGTCTTGCAGAAGCTGGTATGAACCGTGTAGTGGGTGACCACATGGGTATGCTTGCTACAGTAATGAACGGCCTTGCGATGCGTGACGCTCTTCACCGTGCATACGTAAATGCGCGCGTAATGTCTGCAATTCCTCTTAAAGGCGTATGTGACGATTACAATTGGGCTGATGCAATTCGCGAACTTCGTCAAGGCCGCGTTGTAATCTTCTCAGCAGGTACTGGTAACCCATTCTTCACAACAGACTCTGCTGCGTGTCTACGTGGTATTGAAATCGAAGCTGACGTAGTTCTAAAAGCAACGAAAGTTGATGGCGTATTTACTGCTGACCCAGTAGCAAACCCAGACGCAGAGCTGTATGATAAGCTATCTTACGCAGAAGTTCTGGATAAAGAGCTTAAAGTAATGGATTTG | MN253369 |
| BS10 | *Vibrio parahaemolyticus* | GGTGGCGGTAACCTGTTCCGTGGCGCTGGTCTTGCAGAAGCTGGTATGAACCGTGTAGTGGGTGACCACATGGGTATGCTTGCTACAGTAATGAACGGCCTTGCGATGCGTGACGCTCTTCACCGTGCATACGTAAATGCGCGCGTAATGTCTGCAATTCCTCTTAAAGGCGTATGTGACGATTACAATTGGGCTGATGCAATTCGCGAACTTCGTCAAGGCCGCGTTGTAATCTTCTCAGCAGGCACTGGTAACCCATTCTTCACAACAGACTCTGCTGCGTGTCTACGTGGTATCGAAATCGAAGCTGACGTAGTTCTAAAAGCAACGAAAGTTGATGGCGTATTTACTGCTGACCCAGTAGCAAACCCAGACGCAGAGCTGTATGATAAGCTATCTTACGCAGAAGTTCTGGATAAAGAGCTTAAAGTAATGGATTTG | MN253370 |
| BS11 | *Vibrio parahaemolyticus* | GGTGGCGGTAACCTGTTCCGTGGCGCTGGTCTTGCAGAAGCTGGTATGAACCGTGTAGTGGGTGACCACATGGGTATGCTTGCTACAGTAATGAACGGCCTTGCGATGCGTGACGCTCTTCACCGTGCATACGTAAACGCGCGCGTAATGTCTGCAATTCCTCTTAAAGGCGTATGTGACGACTACAATTGGGCTGATGCAATTCGCGAACTTCGTCAAGGCCGCGTTGTAATCTTCTCAGCAGGTACTGGTAACCCATTCTTCACAACAGACTCTGCTGCTTGTCTACGTGGTATCGAAATCGAAGCTGACGTAGTTCTAAAAGCAACGAAAGTTGATGGCGTATTTACTGCTGACCCAGTAGCAAACCCAGACGCAGAGCTGTATGATAAGCTATCTTACGCAGAAGTTCTGGATAAAGAGCTTAAAGTAATGGATTTG | MN253371 |
| BK12 | *Vibrio parahaemolyticus* | GGTGGCGGTAACCTGTTCCGTGGCGCTGGTCTTGCAGAAGCTGGTATGAACCGTGTAGTGGGTGACCACATGGGTATGCTTGCTACAGTAATGAACGGCCTTGCGATGCGTGACGCTCTTCACCGTGCATACGTAAATGCGCGCGTAATGTCTGCAATTCCTCTTAAAGGCGTATGTGACGATTACAATTGGGCTGATGCAATTCGCGAACTTCGTCAAGGCCGCGTTGTAATCTTCTCAGCAGGCACTGGTAACCCATTCTTCACAACAGACTCTGCTGCGTGTCTACGTGGTATCGAAATCGAAGCTGACGTAGTTCTAAAAGCAACGAAAGTTGATGGCGTATTTACTGCTGACCCAGTAGCAAACCCAGACGCAGAGCTGTATGATAAGCTATCTTACGCAGAAGTTCTGGATAAAGAGCTTAAAGTAATGGATTTG | MN253372 |
| BK13 | *Vibrio parahaemolyticus* | GGTGGCGGTAACCTGTTCCGTGGCGCTGGTCTTGCAGAAGCTGGTATGAACCGTGTAGTGGGTGACCACATGGGTATGCTTGCTACAGTAATGAACGGCCTTGCGATGCGTGACGCTCTTCACCGTGCATACGTAAATGCGCGCGTAATGTCTGCAATTCCTCTTAAAGGCGTATGTGACGATTACAATTGGGCTGATGCAATTCGCGAACTTCGTCAAGGCCGCGTTGTAATCTTCTCAGCAGGCACTGGTAACCCATTCTTCACAACAGACTCTGCTGCGTGTCTACGTGGTATCGAAATCGAAGCTGACGTAGTTCTAAAAGCAACGAAAGTTGATGGCGTATTTACTGCTGACCCAGTAGCAAACCCAGACGCAGAGCTGTATGATAAGCTATCTTACGCAGAAGTTCTGGATAAAGAGCTTAAAGTAATGGATTTG | MN253373 |
| BK18 | *Vibrio parahaemolyticus* | GGTGGCGGTAACCTGTTCCGTGGCGCTGGTCTTGCAGAAGCTGGTATGAACCGTGTAGTGGGTGACCACATGGGTATGCTTGCTACAGTAATGAACGGCCTTGCGATGCGTGACGCTCTTCACCGTGCATACGTAAATGCGCGCGTAATGTCTGCAATTCCTCTTAAAGGCGTATGTGATGACTACAATTGGGCTGATGCAATTCGCGAACTTCGTCAAGGCCGCGTTGTAATCTTCTCAGCAGGTACTGGTAACCCATTCTTCACAACAGACTCTGCTGCGTGTCTACGTGGTATCGAAATCGAAGCTGACGTAGTTCTAAAAGCAACGAAAGTTGATGGCGTATTTACTGCTGACCCAGTAGCAAACCCAGACGCAGAGCTGTATGATAAGCTATCTTACGCAGAAGTTCTGGATAAAGAGCTTAAAGTAATGGATTTG | MN253374 |
| BL18 | *Vibrio parahaemolyticus* | GGTGGCGGTAACCTGTTCCGTGGCGCTGGTCTTGCAGAAGCTGGTATGAACCGTGTAGTGGGTGACCACATGGGTATGCTTGCTACAGTAATGAACGGCCTTGCGATGCGTGACGCTCTTCACCGTGCATACGTAAATGCGCGCGTAATGTCTGCAATTCCTCTTAAAGGCGTATGTGACGATTACAATTGGGCTGATGCAATTCGCGAACTTCGTCAAGGCCGCGTTGTAATCTTCTCAGCAGGCACTGGTAACCCATTCTTCACAACAGACTCTGCTGCGTGTCTACGTGGTATCGAAATCGAAGCTGACGTAGTTCTAAAAGCAACGAAAGTTGATGGCGTATTTACTGCTGACCCAGTAGCAAACCCAGACGCAGAGCTGTATGATAAGCTATCTTACGCAGAAGTTCTGGATAAAGAGCTTAAAGTAATGGATTTG | MN253375 |
| BL20 | *Vibrio parahaemolyticus* | GGTGGCGGTAACCTGTTCCGTGGCGCTGGTCTTGCAGAAGCTGGTATGAATCGTGTAGTGGGTGACCACATGGGTATGCTTGCTACAGTAATGAACGGCCTTGCGATGCGTGACGCTCTTCACCGTGCATACGTAAATGCGCGCGTAATGTCTGCAATTCCTCTTAAAGGCGTATGTGACGATTACAATTGGGCTGATGCAATTCGCGAACTTCGTCAAGGCCGCGTTGTAATCTTCTCAGCAGGCACTGGTAACCCATTCTTCACAACAGACTCTGCTGCGTGTCTACGTGGTATCGAAATCGAAGCTGACGTAGTTCTAAAAGCAACGAAAGTTGATGGCGTATTTACTGCTGACCCAGTAGCAAACCCAGACGCAGAGCTGTATGATAAGCTATCTTACGCAGAAGTTCTGGATAAAGAGCTTAAAGTAATGGATTTG | MN253376 |
| BL21 | *Vibrio parahaemolyticus* | GGTGGCGGTAACCTGTTCCGTGGCGCTGGTCTTGCAGAAGCTGGTATGAACCGTGTAGTGGGTGACCACATGGGTATGCTTGCTACAGTAATGAACGGCCTTGCGATGCGTGACGCTCTTCACCGTGCATACGTAAATGCGCGCGTAATGTCTGCAATTCCTCTTAAAGGCGTATGTGACGATTACAATTGGGCTGATGCAATTCGCGAACTTCGTCAAGGCCGCGTTGTAATCTTCTCAGCAGGCACTGGTAACCCATTCTTCACAACAGACTCTGCTGCGTGTCTACGTGGTATCGAAATCGAAGCTGACGTAGTTCTAAAAGCAACGAAAGTTGATGGCGTATTTACTGCTGACCCAGTAGCAAACCCAGACGCAGAGCTGTATGATAAGCTATCTTACGCAGAAGTTCTGGATAAAGAGCTTAAAGTAATGGATTTG | MN253377 |
| BS28 | *Vibrio parahaemolyticus* | GGTGGCGGTAACCTGTTCCGTGGCGCTGGTCTTGCAGAAGCTGGTATGAACCGTGTAGTGGGTGACCACATGGGTATGCTTGCTACAGTAATGAACGGCCTTGCGATGCGTGACGCTCTTCACCGTGCATACGTAAATGCGCGCGTAATGTCTGCAATTCCTCTTAAAGGCGTATGTGATGACTACAATTGGGCTGATGCAATTCGCGAACTTCGTCAAGGCCGCGTTGTAATCTTCTCAGCAGGTACTGGTAACCCATTCTTCACAACAGACTCTGCTGCGTGTCTACGTGGTATCGAAATCGAAGCTGACGTAGTTCTAAAAGCAACGAAAGTTGATGGCGTATTTACTGCTGACCCAGTAGCAAACCCAGACGCAGAGCTGTATGATAAGCTATCTTACGCAGAAGTACTGGATAAAGAGCTAAAAGTAATGGATTTG | MN253378 |
| BK28 | *Vibrio parahaemolyticus* | GGTGGCGGTAACCTGTTCCGTGGCGCTGGTCTTGCAGAAGCTGGTATGAACCGTGTAGTGGGTGACCACATGGGTATGCTTGCTACAGTAATGAACGGCCTTGCGATGCGTGACGCTCTTCACCGTGCATACGTAAATGCGCGCGTAATGTCTGCAATTCCTCTTAAAGGCGTATGTGATGACTACAATTGGGCTGATGCAATTCGCGAACTTCGTCAAGGCCGCGTTGTAATCTTCTCAGCAGGTACTGGTAACCCATTCTTCACAACAGACTCTGCTGCCTGTCTACGTGGTATCGAAATCGAAGCTGACGTAGTTCTAAAAGCAACGAAAGTTGATGGCGTATTTACTGCTGACCCAGTAGCAAACCCAGACGCAGAGCTGTATGATAAGCTATCTTACGCAGAAGTACTGGATAAAGAGCTAAAAGTAATGGATTTG | MN253379 |
| PKL01 | *Vibrio parahaemolyticus* | GGTGGCGGTAACCTGTTCCGTGGCGCTGGTCTTGCAGAAGCTGGTATGAACCGTGTAGTGGGTGACCACATGGGTATGCTTGCTACAGTAATGAACGGCCTTGCGATGCGTGACGCTCTTCACCGTGCATACGTAAACGCGCGCGTAATGTCTGCAATTCCTCTTAAAGGTGTATGTGACGACTACAATTGGGCTGATGCAATTCGCGAACTTCGTCAAGGCCGCGTTGTAATCTTCTCAGCAGGTACTGGTAACCCATTCTTCACCACAGACTCTGCTGCGTGTCTACGTGGTATCGAAATCGAAGCTGACGTAGTTCTAAAAGCAACGAAAGTTGATGGCGTATTTACTGCTGACCCAGTAGCAAACCCAGACGCAGAGCTGTATGATAAGCTATCTTACGCAGAGGTTCTGGATAAAGAGCTTAAAGTAATGGATTTG | MN253380 |
| PKK01 | *Vibrio parahaemolyticus* | GGTGGCGGTAACCTGTTCCGTGGCGCTGGTCTTGCAGAAGCTGGTATGAACCGTGTAGTGGGTGACCACATGGGTATGCTTGCTACAGTAATGAACGGCCTTGCGATGCGTGACGCTCTACACCGTGCATACGTAAATGCGCGCGTAATGTCTGCAATTCCTCTTAAAGGCGTATGTGACGATTACAATTGGGCTGATGCAATTCGCGAACTTCGTCAAGGCCGCGTTGTAATCTTCTCAGCAGGCACTGGTAACCCATTCTTCACAACAGACTCTGCTGCGTGTCTACGTGGTATCGAAATCGAAGCTGACGTAGTTCTAAAAGCAACGAAAGTTGATGGCGTATTTACTGCTGACCCAGTAGCAAACCCAGACGCAGAGCTGTATGATAAGCTATCTTACGCAGAAGTTCTGGATAAAGAGCTTAAAGTAATGGATTTG | MN253381 |
| PKS07 | *Vibrio parahaemolyticus* | GGTGGCGGTAACCTGTTCCGTGGCGCTGGTCTTGCAGAAGCTGGTATGAACCGTGTAGTGGGTGACCACATGGGTATGCTTGCTACAGTAATGAACGGCCTTGCGATGCGTGACGCTCTTCACCGTGCATACGTAAATGCGCGCGTAATGTCTGCAATTCCTCTTAAAGGCGTATGTGACGATTACAATTGGGCTGATGCAATTCGCGAACTTCGTCAAGGCCGCGTTGTAATCTTCTCAGCAGGCACTGGTAACCCATTCTTCACAACAGACTCTGCTGCGTGTCTACGTGGTATCGAAATCGAAGCTGACGTAGTTCTAAAAGCAACGAAAGTTGATGGCGTATTTACTGCTGACCCAGTAGCAAACCCAGACGCAGAGCTGTATGATAAGCTATCTTACGCAGAAGTTCTGGATAAAGAGCTTAAAGTAATGGATTTG | MN253382 |
| PKK07 | *Vibrio parahaemolyticus* | GGTGGCGGTAACCTGTTCCGTGGCGCTGGTCTTGCAGAAGCTGGTATGAACCGTGTAGTGGGTGACCACATGGGTATGCTTGCTACAGTAATGAACGGCCTTGCGATGCGTGACGCTCTTCACCGTGCATACGTAAATGCGCGCGTAATGTCTGCAATCCCTCTTAAAGGCGTATGTGACGATTACAATTGGGCTGATGCAATTCGCGAACTTCGTCAAGGCCGCGTTGTAATCTTCTCAGCAGGTACTGGTAACCCATTCTTCACAACAGACTCTGCTGCGTGTCTACGTGGTATTGAAATCGAAGCTGACGTAGTTCTAAAAGCAACGAAAGTTGATGGCGTATTTACTGCTGACCCAGTAGCAAACCCAGACGCAGAGCTGTATGATAAGCTATCTTACGCAGAAGTTCTGGATAAAGAGCTTAAAGTAATGGATTTG | MN253383 |
| PKK14 | *Vibrio parahaemolyticus* | GGTGGCGGTAACCTGTTCCGTGGCGCTGGTCTTGCAGAAGCTGGTATGAACCGTGTAGTGGGTGACCACATGGGTATGCTTGCTACAGTAATGAACGGCCTTGCGATGCGTGACGCTCTTCATAGAGCGTACGTAAACGCGCGCGTAATGTCTGCAATTCCTCTTAAAGGTGTATGTGACGACTACAATTGGGCTGATGCAATTCGCGAACTTCGTCAAGGCCGCGTTGTAATCTTCTCAGCAGGTACTGGTAACCCATTCTTCACCACAGACTCTGCTGCGTGTCTACGTGGTATCGAAATCGAAGCTGACGTAGTTCTAAAAGCAACGAAAGTTGATGGCGTATTTACTGCTGACCCAGTAGCAAACCCAGACGCAGAGCTGTATGATAAGCTATCTTACGCAGAGGTTCTGGATAAAGAGCTTAAAGTAATGGATTTG | MN253384 |
| PKL15 | *Vibrio parahaemolyticus* | GGTGGCGGTAACCTGTTCCGTGGCGCTGGTCTTGCAGAAGCTGGTATGAACCGTGTAGTGGGTGACCACATGGGTATGCTTGCTACAGTAATGAACGGCCTTGCGATGCGTGACGCTCTTCATAGAGCGTACGTAAACGCGCGCGTCATGTCTGCAATTCCTCTTAAAGGTGTATGTGACGACTACAATTGGGCTGATGCAATTCGCGAACTTCGTCAAGGCCGCGTTGTAATCTTCTCAGCAGGTACTGGTAACCCATTCTTCACCACAGACTCTGCTGCGTGTCTACGTGGTATCGAAATCGAAGCTGACGTAGTTCTAAAAGCAACGAAAGTTGATGGCGTATTTACTGCTGACCCAGTAGCAAACCCAGACGCAGAGCTGTATGATAAGCTATCTTACGCAGAGGTTCTGGATAAAGAGCTTAAAGTAATGGATTTG | MN253385 |
| PKK20 | *Vibrio parahaemolyticus* | GGTGGCGGTAACCTGTTCCGTGGCGCTGGTCTTGCAGAAGCTGGTATGAACCGTGTAGTGGGTGACCACATGGGTATGCTTGCTACAGTAATGAACGGCCTTGCGATGCGTGACGCTCTTCACCGTGCATACGTAAACGCGCGCGTAATGTCTGCAATTCCTCTTAAAGGTGTATGTGACGACTACAATTGGGCTGATGCAATTCGCGAACTTCGTCAAGGCCGCGTTGTAATCTTCTCAGCAGGTACTGGTAACCCATTCTTCACCACAGACTCTGCTGCGTGTCTACGTGGTATCGAAATCGAAGCTGACGTAGTTCTAAAAGCAACGAAAGTTGATGGCGTATTTACTGCTGACCCAGTAGCAAACCCAGACGCAGAGCTGTATGATAAGCTATCTTACGCTGAGGTTCTGGATAAAGAGCTTAAAGTAATGGATTTG | MN253386 |
| PKS21 | *Vibrio parahaemolyticus* | GGTGGCGGTAACCTGTTCCGTGGCGCTGGTCTTGCAGAAGCTGGTATGAACCGTGTAGTGGGTGACCACATGGGTATGCTTGCTACAGTAATGAACGGCCTTGCGATGCGTGACGCTCTTCACCGTGCATACGTAAACGCGCGCGTAATGTCTGCAATTCCTCTTAAAGGTGTATGTGACGACTACAATTGGGCTGATGCAATTCGCGAACTTCGTCAAGGCCGCGTTGTAATCTTCTCAGCAGGTACTGGTAACCCATTCTTCACCACAGACTCTGCTGCGTGTCTACGTGGTATCGAAATCGAAGCTGACGTAGTTCTAAAAGCAACGAAAGTTGATGGCGTATTTACTGCTGACCCAGTAGCAAACCCAGACGCAGAGCTGTATGATAAGCTATCTTACGCTGAGGTTCTGGATAAAGAGCTTAAAGTAATGGATTTG | MN253387 |
| PKK24 | *Vibrio parahaemolyticus* | GGTGGCGGTAACCTGTTCCGTGGCGCTGGTCTTGCAGAAGCTGGTATGAACCGTGTAGTGGGTGACCACATGGGTATGCTTGCTACAGTAATGAACGGCCTTGCGATGCGTGACGCTCTTCACCGTGCATACGTAAACGCGCGCGTAATGTCTGCAATTCCTCTTAAAGGTGTATGTGACGACTACAATTGGGCTGATGCAATTCGCGAACTTCGTCAAGGCCGCGTTGTAATCTTCTCAGCAGGTACTGGTAACCCATTCTTCACCACAGACTCTGCTGCGTGTCTACGTGGGATCGAAATCGAAGCTGACGTAGTTCTAAAAGCAACGAAAGTTGATGGCGTATTTACTGCTGACCCAGTAGCAAACCCAGACGCAGAGCTGTATGATAAGCTATCTTACGCTGAGGTTCTGGATAAAGAGCTTAAAGTAATGGATTTG | MN253388 |
| NK06 | *Vibrio parahaemolyticus* | GGTGGCGGTAACCTGTTCCGTGGCGCTGGTCTTGCAGAAGCTGGTATGAACCGTGTAGTGGGTGACCACATGGGTATGCTTGCTACAGTAATGAACGGCCTTGCGATGCGTGACGCTCTTCACCGTGCATACGTAAATGCGCGCGTAATGTCTGCAATTCCTCTTAAAGGTGTATGTGACGACTACAATTGGGCTGATGCAATTCGCGAACTTCGTCAAGGCCGCGTTGTAATCTTCTCAGCAGGTACTGGTAACCCATTCTTCACCACAGACTCTGCTGCGTGTCTACGTGGGATCGAAATCGAAGCTGACGTAGTTCTAAAAGCAACGAAAGTTGATGGCGTATTTACTGCTGACCCAGTAGCAAACCCAGACGCAGAGCTGTATGATAAGCTATCTTACGCTGAGGTTCTGGATAAAGAGCTTAAAGTAATGGATTTG | MN253389 |
| NL07 | *Vibrio parahaemolyticus* | GGTGGCGGTAACCTGTTCCGTGGCGCTGGTCTTGCAGAAGCTGGTATGAACCGTGTAGTGGGTGACCACATGGGTATGCTTGCTACAGTAATGAACGGCCTTGCGATGCGTGACGCTCTTCACCGTGCATACGTAAATGCGCGCGTAATGTCTGCAATTCCTCTTAAAGGTGTATGTGACGACTACAATTGGGCTGATGCAATTCGCGAACTTCGTCAAGGCCGCGTTGTAATCTTCTCAGCAGGTACTGGTAACCCATTCTTCACCACAGACTCTGCTGCGTGTCTACGTGGGATCGAAATCGAAGCTGACGTAGTTCTAAAAGCAACGAAAGTTGATGGCGTATTTACTGCTGACCCAGTAGCAAACCCAGACGCAGAGCTGTATGATAAGCTATCTTACGCTGAGGTTCTGGATAAAGAGCTTAAAGTAATGGATTTG | MN253390 |
| NS08 | *Vibrio parahaemolyticus* | GGTGGCGGTAACCTGTTCCGTGGCGCTGGTCTTGCAGAAGCTGGTATGAACCGTGTAGTGGGTGACCACATGGGTATGCTTGCTACAGTAATGAACGGCCTTGCGATGCGTGACGCTCTTCACCGTGCATACGTAAATGCGCGCGTAATGTCTGCAATTCCTCTTAAAGGTGTATGTGACGACTACAATTGGGCTGATGCAATTCGCGAACTTCGTCAAGGCCGCGTTGTAATCTTCTCAGCAGGTACTGGTAACCCATTCTTCACCACAGACTCTGCTGCGTGTCTACGTGGGATCGAAATCGAAGCTGACGTAGTTCTAAAAGCAACGAAAGTTGATGGCGTATTTACTGCTGACCCAGTAGCAAACCCAGACGCAGAGCTGTATGATAAGCTATCTTACGCTGAGGTTCTGGATAAAGAGCTTAAAGTAATGGATTTG | MN253391 |
| NK12 | *Vibrio parahaemolyticus* | GGTGGCGGTAACCTGTTCCGTGGCGCTGGTCTTGCAGAAGCTGGTATGAACCGTGTAGTGGGTGACCACATGGGTATGCTTGCTACAGTAATGAACGGCCTTGCGATGCGTGACGCTCTTCACCGTGCATACGTAAACGCGCGCGTAATGTCTGCAATTCCTCTTAAAGGCGTATGTGACGACTACAATTGGGCTGATGCAATTCGCGAACTTCGTCAAGGCCGCGTTGTAATCTTCTCAGCAGGTACTGGTAACCCATTCTTCACAACAGACTCTGCTGCGTGTCTACGTGGTATCGAAATCGAAGCTGACGTAGTTCTCAAAGCAACGAAAGTTGATGGCGTATTTACTGCTGACCCAGTAGCAAACCCAGACGCAGAGCTGTATGATAAGCTATCTTACGCAGAAGTTCTGGATAAAGAGCTTAAAGTAATGGATTTG | MN253392 |
| NL17 | *Vibrio parahaemolyticus* | GGTGGCGGTAACCTGTTCCGTGGCGCTGGTCTTGCAGAAGCTGGTATGAACCGTGTAGTGGGTGACCACATGGGTATGCTTGCTACAGTAATGAACGGCCTTGCGATGCGTGACGCTCTTCACCGTGCATACGTAAATGCGCGCGTAATGTCTGCAATTCCTCTTAAAGGTGTATGTGACGACTACAATTGGGCTGATGCAATTCGCGAACTTCGTCAAGGCCGCGTTGTAATCTTCTCAGCAGGTACTGGTAACCCATTCTTCACCACAGACTCTGCTGCGTGTCTACGTGGGATCGAAATCGAAGCTGACGTAGTTCTAAAAGCAACGAAAGTTGATGGCGTATTTACTGCTGACCCAGTAGCAAACCCAGACGCAGAGCTGTATGATAAGCTATCTTACGCTGAGGTTCTGGATAAAGAGCTTAAAGTAATGGATTTG | MN253393 |
| NS19 | *Vibrio parahaemolyticus* | GGTGGCGGTAACCTGTTCCGTGGCGCTGGTCTTGCAGAAGCTGGTATGAACCGTGTAGTGGGTGACCACATGGGTATGCTTGCTACAGTAATGAACGGCCTTGCGATGCGTGACGCTCTTCACCGTGCATACGTAAATGCGCGCGTAATGTCTGCAATTCCTCTTAAAGGCGTATGTGACGACTACAATTGGGCTGATGCAATTCGCGAACTTCGTCAAGGCCGCGTTGTAATCTTCTCAGCAGGTACTGGTAACCCATTCTTCACAACAGACTCTGCTGCGTGTCTACGTGGTATCGAAATCGAAGCTGACGTAGTTCTAAAAGCAACGAAAGTTGATGGCGTATTTACTGCTGACCCAGTAGCAAACCCAGACGCAGAGCTGTATGATAAGCTATCTTACGCTGAGGTTCTGGATAAAGAGCTTAAAGTAATGGATTTG | MN253394 |
| NL23 | *Vibrio parahaemolyticus* | GGTGGCGGTAACCTGTTCCGTGGCGCTGGTCTTGCAGAAGCTGGTATGAACCGTGTAGTGGGTGACCACATGGGTATGCTTGCTACAGTAATGAACGGCCTTGCGATGCGTGACGCTCTTCACCGTGCATACGTAAACGCGCGCGTAATGTCTGCAATTCCTCTTAAAGGCGTATGTGACGACTACAATTGGGCTGATGCAATTCGCGAACTTCGTCAAGGCCGCGTTGTAATCTTCTCAGCAGGTACTGGTAACCCATTCTTCACAACAGACTCTGCTGCGTGTCTACGTGGTATCGAAATCGAAGCTGACGTAGTTCTAAAAGCAACGAAAGTTGATGGCGTATTTACTGCTGACCCAGTAGCAAACCCAGACGCAGAGCTGTATGATAAGCTATCTTACGCAGAAGTTCTGGATAAAGAGCTGAAAGTAATGGATTTG | MN253395 |
| NL27 | *Vibrio parahaemolyticus* | GGTGGCGGTAACCTGTTCCGTGGCGCTGGTCTTGCAGAAGCTGGTATGAACCGTGTAGTGGGTGACCACATGGGTATGCTTGCTACAGTAATGAACGGCCTTGCGATGCGTGACGCTCTTCACCGTGCATACGTAAATGCGCGCGTAATGTCTGCAATTCCTCTTAAAGGCGTATGTGACGACTACAATTGGGCTGATGCAATTCGCGAACTTCGTCAAGGCCGCGTTGTAATCTTCTCAGCAGGTACTGGTAACCCATTCTTCACAACAGACTCTGCTGCGTGTCTACGTGGTATCGAAATCGAAGCTGACGTAGTTCTAAAAGCAACGAAAGTTGATGGCGTATTTACTGCTGACCCAGTAGCAAACCCAGACGCAGAGCTGTATGATAAGCTATCTTACGCAGAAGTTCTTGATAAAGAGCTTAAAGTAATGGATTTG | MN253396 |
| NS27 | *Vibrio parahaemolyticus* | GGTGGCGGTAACCTGTTCCGTGGCGCTGGTCTTGCAGAAGCTGGTATGAACCGTGTAGTGGGTGACCACATGGGTATGCTTGCTACAGTAATGAACGGCCTTGCGATGCGTGACGCTCTTCACCGTGCATACGTAAATGCGCGCGTAATGTCTGCAATTCCTCTTAAAGGCGTATGTGATGACTACAATTGGGCTGATGCAATTCGCGAACTTCGTCAAGGCCGCGTTGTAATCTTCTCAGCAGGTACTGGTAACCCATTCTTCACAACAGACTCTGCTGCGTGTCTACGTGGTATCGAAATCGAAGCTGACGTAGTTCTAAAAGCAACGAAAGTTGATGGCGTATTTACTGCTGACCCAGTAGCAAACCCAGACGCAGAGCTGTATGATAAGCTATCTTACGCAGAAGTTCTGGATAAAGAGCTTAAAGTAATGGATTTG | MN253397 |
| NK29 | *Vibrio parahaemolyticus* | GGCGGTGGTAACCTGTTCCGTGGCGCGGGTCTTGCAGAAGCGGGTATGAACCGCGTAGTAGGCGACCACATGGGGATGCTTGCTACAGTAATGAACGGCCTTGCGATGCGTGACGCTCTTCACCGTGCTTATGTAAACGCTCGCGTAATGTCTGCAATTCCGCTAAAAGGCGTATGTGACGACTACAATTGGGCAGATGCAATTCGCGAACTTCGTCAAGGCCGCGTTGTAATCTTCTCAGCAGGTACTGGTAACCCATTCTTCACAACAGACTCTGCTGCGTGTCTACGTGGTATCGAAATCGAAGCTGACGTAGTTCTAAAAGCAACGAAAGTTGATGGCGTATTTACTGCTGACCCAGTAGCAAACCCAGACGCAGAGCTGTATGATAAGCTATCTTACGCAGAAGTTCTGGATAAAGAGCTTAAAGTAATGGATTTG | MN253398 |
| JL05 | *Vibrio parahaemolyticus* | GGTGGCGGTAACCTGTTCCGTGGCGCTGGTCTTGCAGAAGCTGGTATGAACCGTGTAGTGGGTGACCACATGGGTATGCTTGCTACAGTAATGAACGGCCTAGCGATGCGTGACGCTCTTCACCGTGCATACGTAAATGCGCGCGTAATGTCTGCAATTCCTCTTAAAGGCGTATGTGACGACTACAATTGGGCTGATGCTATTCGCGAACTTCGTCAAGGCCGCGTTGTAATCTTCTCAGCAGGTACTGGTAACCCATTCTTCACAACAGACTCTGCTGCGTGTCTACGTGGTATCGAAATCGAAGCTGACGTAGTTCTAAAAGCAACGAAAGTTGATGGCGTATTTACTGCTGACCCAGTAGCAAACCCAGACGCAGAGCTGTATGATAAGCTATCTTACGCAGAAGTTCTGGATAAAGAACTTAAAGTAATGGATTTG | MN253399 |
| JS17 | *Vibrio parahaemolyticus* | GGTGGCGGTAACCTGTTCCGTGGTGCTGGTCTTGCTGAAGCTGGTATGAACCGTGTAGTGGGTGACCACATGGGTATGCTTGCTACAGTAATGAACGGCCTTGCGATGCGTGACGCTCTTCACCGTGCATACGTAAATGCGCGCGTAATGTCTGCAATTCCTCTTAAAGGCGTATGTGACGACTACAATTGGGCTGATGCTATTCGCGAACTTCGTCAAGGCCGCGTTGTAATCTTCTCAGCAGGTACTGGTAACCCATTCTTCACAACAGACTCTGCTGCGTGTCTACGTGGTATCGAAATCGAAGCTGACGTAGTTCTAAAAGCAACGAAAGTTGATGGCGTATTTACTGCTGACCCAGTAGCAAACCCAGACGCAGAGCTGTATGATAAGCTATCTTACGCAGAAGTTCTGGATAAAGAACTTAAAGTAATGGATTTG | MN253400 |
| JL24 | *Vibrio parahaemolyticus* | GGTGGCGGTAACCTGTTCCGTGGCGCTGGTCTTGCAGAAGCTGGTATGAACCGTGTAGTGGGTGACCACATGGGTATGCTTGCTACAGTAATGAACGGCCTTGCGATGCGTGACGCTCTTCACCGTGCATACGTAAATGCGCGCGTAATGTCTGCAATTCCTCTTAAAGGCGTATGTGACGACTACAATTGGGCTGATGCAATTCGCGAACTTCGTCAAGGCCGCGTTGTAATCTTCTCAGCAGGTACTGGTAACCCATTCTTCACAACAGACTCTGCTGCGTGTCTACGTGGTATCGAAATCGAAGCTGACGTAGTTCTAAAAGCAACGAAAGTTGATGGCGTATTTACTGCTGACCCAGTAGCAAACCCAGACGCAGAGCTGTATGATAAGCTATCTTACGCAGAAGTTCTGGATAAAGAACTTAAAGTAATGGATTTG | MN253401 |
| JS24 | *Vibrio parahaemolyticus* | GGTGGCGGTAACCTTTTCCGTGGCGCTGGTCTTGCAGAAGCTGGTATGAACCGTGTAGTGGGTGACCACATGGGTATGCTTGCTACAGTAATGAACGGCCTTGCGATGCGTGACGCTCTTCACCGTGCATACGTAAATGCGCGCGTAATGTCTGCAATTCCTCTTAAAGGCGTATGTGACGACTACAATTGGGCTGATGCAATTCGCGAACTTCGTCAAGGCCGCGTTGTAATCTTCTCAGCAGGTACTGGTAACCCATTCTTCACAACAGACTCTGCTGCGTGTCTACGTGGTATCGAAATCGAAGCTGACGTAGTTCTAAAAGCAACGAAAGTTGATGGCGTATTTACTGCTGACCCAGTAGCAAACCCAGACGCAGAGCTGTATGATAAGCTATCTTACGCAGAAGTTCTGGATAAAGAGCTTAAAGTAATGGATTTG | MN253402 |
| JL25 | *Vibrio parahaemolyticus* | GGTGGCGGTAACCTGTTCCGTGGCGCTGGTCTTGCTGAAGCTGGTATGAACCGTGTAGTGGGTGACCACATGGGTATGCTTGCTACAGTAATGAACGGCCTTGCGATGCGTGACGCTCTTCACCGTGCATACGTAAATGCGCGCGTAATGTCTGCAATTCCTCTTAAAGGCGTATGTGACGACTACAATTGGGCTGATGCAATTCGCGAACTTCGTCAAGGCCGCGTTGTAATCTTCTCAGCAGGTACTGGTAACCCATTCTTCACAACAGATTCTGCTGCTTGTCTACGTGGTATCGAAATCGAAGCTGACGTAGTTCTAAAAGCAACGAAAGTTGATGGCGTATTTACTGCTGACCCAGTAGCAAACCCAGACGCAGAGCTGTATGATAAGCTATCTTACGCAGAAGTTCTGGATAAAGAACTTAAAGTAATGGATTTG | MN253403 |
| JS25 | *Vibrio parahaemolyticus* | GGTGGCGGTAACTTGTTCCGTGGTGCTGGTCTTGCAGAAGCTGGTATGAACCGTGTAGTGGGTGACCACATGGGTATGCTTGCTACAGTAATGAATGGCCTTGCGATGCGTGATGCTCTTCACCGTGCATACGTAAATGCGCGCGTAATGTCTGCAATTCCTCTTAAAGGCGTATGTGACGACTACAATTGGGCTGATGCTATTCGCGAACTTCGCCAAGGCCGCGTTGTAATCTTCTCTGCTGGTACTGGTAACCCATTCTTTACAACAGATTCTGCTGCCTGTCTACGTGGTATCGAAATTGAAGCTGACGTAGTTCTAAAAGCAACGAAAGTTGATGGCGTATTTACTGCTGACCCAGTAGCCAACCCAGACGCAGAGCTGTATGATAAGCTATCTTACGCCGAAGTTCTTGATAAAGAACTTAAAGTAATGGATTTG | MN253404 |
| LS07 | *Vibrio rotiferianus* | GGCGGCGGTAACCTATTCCGTGGTGCTGGTCTAGCTGAAGCGGGCATGAACCGTGTTGTGGGTGACCACATGGGTATGCTAGCAACAGTAATGAACGGTCTAGCAATGCGTGATGCACTTCATCGCGCATACGTAAACGCTCGTGTAATGTCTGCAATTCCGCTAAAAGGTGTGTGTGACGATTACAATTGGGCAGATGCTATTCGTGAACTTCGTCAAGGCCGTGTAGTGATCTTCTCTGCAGGTACAGGTAACCCATTCTTCACGACGGATTCAGCAGCTTGTCTACGTGGTATCGAAATCGAAGCTGACGTAGTTCTAAAAGCGACAAAAGTTGATGGTGTATTTACTGCTGACCCAGTAGCAAACCCAGACGCAGAGCTGTATGATAAGCTATCTTACACTGAAATTCTGGATAAAGAATTGAAAGTAATGGACTTG | MN253405 |
| LS15 | *Vibrio rotiferianus* | GGCGGCGGTAACCTATTCCGTGGTGCTGGTCTAGCTGAAGCGGGCATGAACCGTGTTGTGGGTGACCACATGGGTATGCTAGCAACAGTAATGAACGGTCTAGCAATGCGTGATGCACTTCATCGCGCATACGTAAACGCTCGTGTAATGTCTGCAATTCCGCTAAAAGGTGTGTGTGACGATTACAATTGGGCAGATGCTATTCGTGAACTTCGTCAAGGCCGTGTAGTTATCTTCTCTGCAGGTACAGGTAACCCATTCTTTACGACGGATTCAGCAGCTTGTCTACGTGGTATCGAAATCGAAGCTGACGTAGTTCTAAAAGCGACAAAAGTTGATGGTGTATTTACTGCTGACCCAGTAGCAAACCCAGACGCAGAGCTGTATGATAAGCTATCTTACACTGAAATTCTGGATAAAGAATTGAAAGTAATGGACTTG | MN253406 |
| LK19 | *Vibrio rotiferianus* | GGCGGCGGTAACCTATTCCGTGGTGCTGGTCTAGCTGAAGCAGGCATGAACCGTGTTGTGGGTGACCACATGGGTATGCTAGCAACAGTAATGAACGGTCTAGCAATGCGTGATGCACTTCATCGCGCATACGTAAACGCTCGTGTAATGTCTGCAATTCCGCTAAAAGGTGTGTGTGACGATTACAATTGGGCAGACGCTATTCGTGAACTTCGTCAAGGCCGTGTAGTGATCTTCTCTGCAGGTACAGGTAACCCATTCTTCACGACGGATTCAGCAGCTTGTCTACGTGGTATCGAAATCGAAGCTGACGTAGTTCTAAAAGCGACAAAAGTTGATGGTGTATTTACTGCTGACCCAGTAGCAAACCCAGACGCAGAGCTGTATGATAAGCTATCTTACACTGAAATTCTGGATAAAGAATTGAAAGTAATGGACTTG | MN253407 |
| PKGK09 | *Vibrio rotiferianus* | GGCGGCGGTAACCTATTCCGTGGTGCTGGTCTAGCTGAAGCGGGCATGAACCGTGTTGTGGGTGACCACATGGGTATGCTAGCAACAGTAATGAACGGTCTAGCAATGCGTGATGCACTTCATCGCGCATACGTAAACGCTCGTGTAATGTCTGCAATTCCGCTAAAAGGTGTGTGTGACGATTACAATTGGGCAGATGCTATTCGTGAACTTCGTCAAGGCCGTGTAGTGATCTTCTCTGCAGGTACAGGTAACCCATTCTTTACGACGGATTCAGCAGCGTGTCTACGTGGTATCGAAATCGAAGCTGACGTAGTTCTAAAAGCGACAAAAGTTGATGGTGTATTTACTGCTGACCCAGTAGCAAACCCAGACGCAGAGCTGTATGATAAGCTATCTTACACTGAAATTCTGGATAAAGAATTGAAAGTAATGGACTTG | MN253408 |
| JL17 | *Vibrio rotiferianus* | GGCGGCGGTAACCTGTTCCGTGGTGCTGGTCTTGCTGAAGCGGGTATGAACCGTGTAGTGGGTGACCACATGGGTATGCTTGCAACAGTAATGAACGGTCTAGCAATGCGTGATGCACTTCATCGTGCTTACGTAAACGCTCGCGTAATGTCTGCAATTCCGCTAAAAGGTGTGTGTGACGATTACAATTGGGCAGATGCTATTCGCGAACTTCGTCAAGGCCGTGTAGTTATCTTCTCTGCAGGTACTGGTAACCCATTCTTCACGACAGATTCTGCTGCGTGTCTACGTGGTATCGAAATCGAAGCTGACGTAGTTCTAAAAGCAACGAAAGTTGATGGTGTATTTACTGCTGACCCAGTAGCAAACCCAGACGCAGAGCTGTATGATAAGCTATCTTACACTGAAATTCTGGATAAAGAATTGAAAGTAATGGACTTG | MN253409 |
| JS26 | *Vibrio rotiferianus* | GGCGGCGGTAACCTATTCCGTGGTGCTGGTCTAGCTGAAGCGGGCATGAACCGTGTTGTGGGTGACCACATGGGTATGCTAGCAACAGTAATGAACGGTCTAGCAATGCGTGATGCACTTCATCGCGCATACGTAAACGCTCGTGTAATGTCTGCAATTCCGCTAAAAGGTGTGTGTGACGATTACAATTGGGCAGATGCTATTCGTGAACTTCGTCAAGGCCGTGTAGTGATCTTCTCTGCAGGTACAGGTAACCCATTCTTTACGACGGATTCAGCAGCGTGTCTACGTGGTATCGAAATCGAAGCTGACGTAGTTCTAAAAGCGACAAAAGTTGATGGTGTATTTACTGCTGACCCAGTAGCAAACCCAGACGCAGAGCTGTATGATAAGCTATCTTACACTGAAATTCTGGATAAAGAATTGAAAGTAATGGACTTG | MN253410 |
| JK26 | *Vibrio rotiferianus* | GGCGGCGGTAACCTATTCCGTGGTGCTGGTCTAGCTGAAGCGGGCATGAACCGTGTTGTGGGTGACCACATGGGTATGCTAGCAACAGTAATGAACGGTCTAGCAATGCGTGATGCACTTCATCGCGCATACGTAAACGCTCGTGTAATGTCTGCAATTCCGCTAAAAGGTGTGTGTGACGATTACAATTGGGCAGATGCTATTCGTGAACTTCGTCAAGGCCGTGTAGTGATCTTCTCTGCAGGTACAGGTAACCCATTCTTTACGACGGATTCAGCAGCGTGTCTACGTGGTATCGAAATCGAAGCTGACGTAGTTCTAAAAGCGACAAAAGTTGATGGTGTATTTACTGCTGACCCAGTAGCAAACCCAGACGCAGAGCTGTATGATAAGCTATCTTACACTGAAATTCTGGATAAAGAATTGAAAGTAATGGACTTG | MN253411 |
| JS27 | *Vibrio rotiferianus* | GGCGGCGGTAACCTATTCCGTGGTGCTGGTCTAGCTGAAGCGGGCATGAACCGTGTTGTGGGTGACCACATGGGTATGCTAGCAACAGTAATGAACGGTCTAGCAATGCGTGATGCACTTCATCGCGCATACGTAAACGCTCGTGTAATGTCTGCAATTCCGCTAAAAGGTGTGTGTGACGATTACAATTGGGCAGATGCTATTCGTGAACTTCGTCAAGGCCGTGTAGTGATCTTCTCTGCAGGTACAGGTAACCCATTCTTTACGACGGATTCAGCAGCGTGTCTACGTGGTATCGAAATCGAAGCTGACGTAGTTCTAAAAGCGACAAAAGTTGATGGTGTATTTACTGCTGACCCAGTAGCAAACCCAGACGCAGAGCTGTATGATAAGCTATCTTACACTGAAATTCTGGATAAAGAATTGAAAGTAATGGACTTG | MN253412 |
| JK27 | *Vibrio rotiferianus* | GGCGGCGGTAACCTGTTCCGTGGTGCTGGTCTAGCTGAAGCTGGTATGAACCGTGTTGTGGGTGACCACATGGGTATGCTAGCAACAGTAATGAACGGTCTAGCAATGCGTGATGCACTTCATCGCGCATACGTAAACGCTCGTGTAATGTCTGCAATTCCGCTAAAAGGTGTGTGTGACGATTACAATTGGGCTGATGCTATTCGTGAACTTCGTCAAGGCCGTGTAGTGATCTTCTCTGCAGGTACAGGTAACCCATTCTTTACGACGGATTCAGCAGCGTGTCTACGTGGTATCGAAATCGAAGCTGACGTAGTTCTAAAAGCGACAAAAGTTGATGGTGTATTTACTGCTGACCCAGTAGCAAACCCAGACGCAGAGCTGTATGATAAGCTATCTTACACTGAAATTCTGGATAAAGAATTGAAAGTAATGGACTTG | MN253413 |
| JL28 | *Vibrio rotiferianus* | GGCGGCGGTAACCTGTTCCGTGGTGCTGGTCTAGCTGAAGCTGGCATGAACCGTGTTGTGGGTGACCACATGGGTATGCTAGCAACAGTAATGAACGGTCTAGCAATGCGTGATGCACTTCATCGCGCATACGTAAATGCTCGTGTAATGTCTGCAATTCCTCTAAAAGGTGTGTGTGACGATTACAATTGGGCAGATGCTATTCGTGAACTTCGTCAAGGCCGTGTAGTGATCTTCTCTGCAGGTACTGGTAACCCATTCTTTACGACGGATTCAGCAGCGTGTCTACGTGGTATCGAAATCGAAGCTGACGTAGTTCTAAAAGCGACAAAAGTTGATGGTGTATTTACTGCTGACCCAGTAGCAAACCCAGACGCAGAGCTGTATGATAAGCTATCTTACACTGAAATTCTGGATAAAGAATTGAAAGTAATGGACTTG | MN253414 |
| JS28 | *Vibrio rotiferianus* | GGCGGCGGTAACCTGTTCCGTGGTGCTGGTCTAGCTGAAGCGGGCATGAACCGTGTTGTGGGTGACCACATGGGTATGCTAGCAACAGTAATGAACGGTCTAGCAATGCGTGATGCACTTCATCGCGCATACGTAAACGCTCGTGTAATGTCTGCAATTCCGCTAAAAGGTGTGTGTGACGATTACAATTGGGCAGATGCTATTCGTGAACTTCGTCAAGGCCGTGTAGTGATCTTCTCTGCAGGTACAGGTAACCCATTCTTTACGACGGATTCAGCAGCGTGTCTACGTGGTATCGAAATCGAAGCTGACGTAGTTCTAAAAGCGACAAAAGTTGATGGTGTATTTACTGCTGACCCAGTAGCAAACCCAGACGCAGAGCTGTATGATAAGCTATCTTACACTGAAATTCTGGATAAAGAATTGAAAGTAATGGACTTG | MN253415 |
| PJS02 | *Vibrio tubiashii* | GGTGGTGGTAACTTGTTCCGTGGTGCAGGCCTAGCTGAAGCGGGCATGAACCGTGTAGTAGGTGACCACATGGGTATGCTAGCGACGGTAATGAACGGCCTTGCGATGCGTGATGCACTGCACCGTGCATATGTTAATGCTCGCGTTATGTCAGCTATTCCACTAAAAGGTGTGTGTGACGACTACAATTGGGCAGACGCTATTCGTGAACTACGCCAAGGTCGCGTTGTTATCTTCTCTGCTGGTACAGGTAATCCATTCTTTACGACGGATTCTGCTGCTTGTCTACGCGGTATTGAGATTGAAGCTGACGTAGTTCTAAAAGCGACAAAAGTAGATGGTGTATTTACGGCTGACCCAGTAGCAAACCCAGACGCAGAGCTGTATGATAAGCTTTCATTCAACGCGGTTCTTGAAAAAGAGCTGAAAGTGATGGATTTG | MN253416 |
| PJS12 | *Vibrio tubiashii* | GGTGGTGGTAACTTGTTCCGTGGTGCAGGCCTAGCTGAAGCGGGCATGAACCGTGTAGTAGGTGACCACATGGGTATGCTAGCGACGGTAATGAACGGCCTTGCGATGCGTGATGCACTGCACCGTGCATATGTTAATGCTCGCGTTATGTCAGCTATTCCACTAAAAGGTGTGTGTGACGACTACAATTGGGCAGACGCTATTCGTGAACTACGCCAAGGTCGCGTTGTTATCTTCTCTGCTGGTACAGGTAATCCATTCTTTACGACGGATTCTGCTGCTTGTCTACGCGGTATTGAGATTGAAGCTGACGTAGTTCTAAAAGCGACAAAAGTAGATGGTGTATTTACGGCTGACCCAGTAGCAAACCCAGACGCAGAGCTGTATGATAAGCTTTCATTCAACGCGGTTCTTGAAAAAGAGCTGAAAGTGATGGATTTG | MN253417 |
| PJK19 | *Vibrio tubiashii* | GGTGGTGGTAACCTTTTCCGTGGTGCAGGTCTTGCTGAAGCGGGCATGAACCGAGTTGTCGGTGACCACATGGGCATGCTGGCAACAGTAATGAATGGCCTTGCAATGCGTGATGCGTTGCACCGTGCGTACGTAAATGCACGAGTAATGTCAGCGATTCCTCTAAAAGGCGTGTGTGATGATTACAATTGGGCAGACGCGATTCGTGAACTTCGTCAAGGCCGTGTAGTTATCTTCTCAGCAGGTACAGGTAACCCATTCTTTACTACGGATTCTGCTGCTTGTTTACGCGGTATTGAAATTGAAGCGGACGTAGTTCTAAAAGCAACAAAAGTGGATGGCGTATTTACGGCTGACCCAGTAGCAAACCCTGACGCAGAGCTGTATGATAAGCTTTCATTTAACGCAGTTCTTGAAAAAGAACTGAAAGTGATGGATTTG | MN253418 |
| PJS21 | *Vibrio tubiashii* | GGTGGCGGTAACTTGTTCCGTGGCGCTGGCCTAGCTGAAGCAGGAATGAACCGCGTAGTGGGTGACCACATGGGTATGTTAGCAACCGTTATGAACGGTCTTGCTATGCGTGATGCACTGCACCGTGCATACGTTAATGCTCGCGTTATGTCAGCTATCCCACTAAAAGGTGTGTGTGACGACTACAACTGGGCTGACGCTATTCGAGAACTACGCCAAGGTCGCGTAGTTATTTTCTCGGCAGGTACAGGTAACCCATTCTTTACGACGGATTCTGCTGCATGTTTGCGCGGTATTGAGATTGAAGCTGACGTAGTTCTAAAAGCGACAAAAGTAGATGGAGTATTTACGGCTGACCCAGTAGCAAACCCAGACGCAGAGCTGTATGATAAGCTTTCATTCAACGCGGTTCTTGAAAAAGAGCTGAAAGTGATGGATTTG | MN253419 |
| LK05 | *Vibrio vulnificus* | GGTGGCGGTAACTTGTTCCGTGGTGCTGGTCTTGCGCAAGCTGGTATGAACCGTGTTGTGGGTGACCACATGGGTATGCTTGCAACAGTAATGAATGGCCTAGCGATGCGCGATGCGCTACACCGTGCTTACGTAAATGCTCGCGTGATGTCTGCAATTCCACTTAACGGTGTTTGTGATGACTACAACTGGGCAGACGCTATCCGCGAACTTCGCCAAGGTCGCGTGGTTATTTTTGCCGCAGGTACTGGTAACCCATTCTTCACTACTGATTCTGCGGCTTGCCTACGTGGTATTGAGATTGAAGCTGATGTCGTTCTAAAAGCAACTAAAGTGGATGGCGTATTTACTGCTGACCCGGTAGCCAACCCAGACGCAGAGCTGTATGATAAGCTTTCTTACACCGATGTTCTTGAAAAAGAACTGAAAGTGATGGATTTG | MN253420 |
| LK18 | *Vibrio vulnificus* | GGTGGCGGTAACTTGTTCCGTGGTGCTGGTCTTGCGCAAGCTGGTATGAACCGTGTTGTGGGTGACCACATGGGTATGCTTGCAACAGTAATGAATGGCCTAGCGATGCGCGATGCGCTACACCGTGCTTACGTAAATGCTCGCGTGATGTCTGCAATTCCACTTAACGGTGTTTGTGATGACTACAACTGGGCAGACGCTATCCGCGAACTTCGCCAAGGTCGCGTGGTTATTTTTGCCGCAGGTACTGGTAACCCATTCTTCACTACTGATTCTGCGGCTTGCCTACGTGGTATTGAGATTGAAGCTGATGTCGTTCTAAAAGCAACTAAAGTGGATGGCGTATTTACTGCTGACCCGGTAGCCAACCCAGACGCAGAGCTGTATGATAAGCTTTCTTACACCGATGTTCTTGAAAAAGAACTGAAAGTGATGGATTTG | MN253421 |
| LL21 | *Vibrio vulnificus* | GGTGGCGGTAACTTGTTCCGTGGTGCTGGTCTTGCGCAAGCTGGTATGAACCGTGTTGTGGGTGACCACATGGGTATGCTTGCAACAGTAATGAATGGCCTAGCGATGCGCGATGCGCTACACCGTGCTTACGTAAATGCTCGCGTGATGTCTGCAATTCCACTTAACGGTGTTTGTGATGACTACAACTGGGCAGACGCTATCCGCGAACTTCGCCAAGGTCGCGTGGTTATTTTTGCCGCAGGTACTGGTAACCCATTCTTCACTACTGATTCTGCGGCTTGCCTACGTGGTATTGAGATTGAAGCTGATGTCGTTCTAAAAGCAACTAAAGTGGATGGCGTATTTACTGCTGACCCGGTAGCCAACCCAGACGCAGAGCTGTATGATAAGCTTTCTTACACCGATGTTCTTGAAAAAGAACTGAAAGTGATGGATTTG | MN253422 |
| LL22 | *Vibrio vulnificus* | GGTGGCGGTAACTTGTTCCGTGGTGCTGGTCTTGCGCAAGCTGGTATGAACCGTGTTGTGGGTGACCACATGGGTATGCTTGCAACAGTAATGAATGGCCTAGCGATGCGCGATGCGCTACACCGTGCTTACGTAAATGCTCGCGTGATGTCTGCAATTCCACTTAACGGTGTTTGTGATGACTACAACTGGGCAGACGCTATCCGCGAACTTCGCCAAGGTCGCGTGGTTATTTTTGCCGCAGGTACTGGTAACCCATTCTTCACTACTGATTCTGCGGCTTGCCTACGTGGTATTGAGATTGAAGCTGATGTCGTTCTAAAAGCAACTAAAGTGGATGGCGTATTTACTGCTGACCCGGTAGCCAACCCAGACGCAGAGCTGTATGATAAGCTTTCTTACACCGATGTTCTTGAAAAAGAACTGAAAGTGATGGATTTG | MN253423 |
| LS28 | *Vibrio vulnificus* | GGTGGCGGTAACTTGTTCCGTGGTGCTGGTCTTGCGCAAGCTGGTATGAACCGTGTTGTGGGTGACCACATGGGTATGCTTGCAACAGTAATGAATGGCCTAGCGATGCGCGATGCGCTACACCGTGCTTACGTAAATGCTCGCGTGATGTCTGCAATTCCACTTAACGGTGTTTGTGATGACTACAACTGGGCAGACGCTATCCGCGAACTTCGCCAAGGTCGCGTGGTTATTTTTGCCGCAGGTACTGGTAACCCATTCTTCACTACTGATTCTGCGGCTTGCCTACGTGGTATTGAGATTGAGGCAGATGTCGTTCTAAAAGCAACTAAAGTGGATGGCGTATTTACTGCTGACCCGGTAGCCAACCCAGACGCAGAGCTGTATGATAAGCTTTCTTACACCGATGTTCTTGAAAAAGAACTGAAAGTGATGGATTTG | MN253424 |
| PJK08 | *Vibrio vulnificus* | GGTGGCGGTAACTTGTTCCGTGGTGCTGGTCTAGCGCAAGCTGGTATGAACCGTGTTGTGGGTGACCACATGGGTATGCTTGCAACGGTAATGAATGGCCTAGCGATGCGCGATGCACTACACCGTGCTTACGTAAATGCTCGCGTGATGTCTGCAATTCCACTTAACGGTGTTTGTGATGACTACAACTGGGCAGACGCTATCCGCGAACTTCGCCAAGGTCGCGTGGTTATTTTTGCCGCAGGTACTGGTAACCCATTCTTCACTACTGATTCTGCGGCTTGTCTACGTGGTATTGAAATTGAAGCGGATGTCGTTCTAAAAGCGACTAAGGTGGATGGCGTATTTACTGCTGACCCGGTAGCCAACCCAGACGCAGAGCTGTATGATAAGCTTTCTTACACCGATGTTCTTGAAAAAGAACTGAAAGTGATGGATTTG | MN253425 |
| PJK12 | *Vibrio vulnificus* | GGTGGCGGTAACTTGTTCCGTGGTGCTGGTCTAGCGCAAGCTGGTATGAACCGTGTTGTGGGTGACCACATGGGTATGCTTGCAACGGTAATGAATGGCCTAGCGATGCGCGATGCGCTACACCGTGCTTACGTAAATGCTCGCGTGATGTCTGCAATTCCACTTAACGGTGTTTGTGATGACTACAACTGGGCAGACGCTATCCGCGAACTTCGCCAAGGTCGCGTGGTTATTTTTGCCGCAGGTACTGGTAACCCATTCTTCACTACTGATTCTGCGGCTTGCCTACGTGGTATTGAGATTGAAGCGGATGTCGTTCTAAAAGCGACTAAGGTGGATGGCGTATTTACTGCTGACCCGGTAGCCAACCCAGACGCAGAGCTGTATGATAAGCTTTCTTACACCGATGTTCTTGAAAAAGAACTGAAAGTGATGGATTTG | MN253426 |
| PJS16 | *Vibrio vulnificus* | GGTGGCGGTAACTTGTTCCGTGGTGCTGGTCTAGCGCAAGCTGGTATGAACCGTGTTGTGGGTGACCACATGGGTATGCTAGCAACGGTAATGAATGGCCTAGCGATGCGCGATGCGCTACACCGTGCTTACGTAAATGCACGCGTGATGTCTGCAATTCCACTTAACGGTGTTTGTGATGACTACAACTGGGCAGACGCTATCCGCGAACTTCGCCAAGGTCGCGTGGTTATTTTTGCCGCAGGTACTGGCAACCCATTCTTCACTACTGATTCTGCGGCTTGCCTACGTGGTATTGAGATTGAAGCGGATGTCGTTCTAAAAGCGACTAAAGTGGATGGCGTATTTACTGCTGACCCGGTAGCCAACCCAGACGCAGAGCTGTATGATAAGCTTTCTTACACCGATGTTCTTGAAAAAGAACTGAAAGTGATGGATTTG | MN253427 |
| PJS19 | *Vibrio vulnificus* | GGTGGCGGTAACTTGTTCCGTGGTGCTGGTCTAGCGCAAGCTGGTATGAACCGTGTTGTGGGTGACCACATGGGTATGCTTGCAACGGTAATGAATGGCCTAGCGATGCGCGATGCTCTACACCGTGCTTACGTAAATGCACGCGTGATGTCTGCAATTCCTCTTAACGGTGTTTGTGATGACTACAACTGGGCTGATGCTATCCGCGAACTTCGCCAAGGTCGCGTGGTTATCTTTGCCGCAGGTACTGGCAACCCATTCTTCACTACTGATTCTGCTGCTTGCCTACGTGGTATCGAAATCGAAGCTGATGTCGTTCTAAAAGCAACTAAAGTGGATGGCGTATTTACTGCTGACCCGGTAGCCAACCCAGACGCAGAGCTGTATGATAAGCTTTCTTACACCGATGTTCTGGAGAAAGAACTGAAAGTGATGGATTTG | MN253428 |
| PJS29 | *Vibrio vulnificus* | GGTGGCGGTAACTTGTTCCGTGGTGCTGGTCTAGCGCAAGCTGGTATGAACCGTGTTGTGGGTGACCACATGGGTATGCTTGCAACGGTAATGAATGGCCTAGCGATGCGCGATGCGCTACACCGTGCTTACGTAAATGCTCGCGTGATGTCTGCAATTCCACTTAACGGTGTTTGTGATGACTACAACTGGGCAGACGCTATCCGCGAACTTCGCCAAGGTCGCGTGGTTATTTTTGCCGCAGGTACTGGTAACCCATTCTTCACTACTGATTCTGCGGCTTGCCTACGTGGTATTGAAATTGAAGCTGATGTCGTTCTAAAAGCGACTAAAGTGGATGGCGTATTTACTGCTGACCCGGTAGCCAACCCAGACGCAGAGCTGTATGATAAGCTTTCTTACACCGATGTTCTTGAAAAAGAACTGAAAGTGATGGATTTG | MN253429 |
| PKGS02 | *Vibrio vulnificus* | GGTGGCGGTAACTTGTTCCGTGGTGCTGGTCTAGCGCAAGCTGGTATGAACCGTGTTGTGGGTGACCACATGGGTATGCTAGCAACGGTAATGAATGGCCTAGCGATGCGCGATGCGCTACACCGTGCTTACGTAAATGCACGCGTGATGTCTGCAATTCCACTTAACGGTGTTTGTGATGACTACAACTGGGCAGACGCTATCCGCGAACTTCGCCAAGGTCGCGTGGTTATTTTTGCCGCAGGTACTGGCAACCCATTCTTCACTACTGATTCTGCGGCTTGCCTACGTGGTATTGAGATTGAAGCGGATGTCGTTCTAAAAGCGACTAAAGTGGATGGCGTATTTACTGCTGACCCGGTAGCCAACCCAGACGCAGAGCTGTATGATAAGCTTTCTTACACCGATGTTCTTGAAAAAGAACTGAAAGTGATGGATTTG | MN253430 |
| PKGK03 | *Vibrio vulnificus* | GGTGGCGGTAACTTGTTCCGTGGTGCTGGTCTAGCGCAAGCTGGTATGAACCGTGTTGTGGGTGACCACATGGGTATGCTAGCAACGGTAATGAATGGCCTAGCGATGCGCGATGCGCTACACCGTGCTTACGTAAATGCACGCGTGATGTCTGCAATTCCACTTAACGGTGTTTGTGATGACTACAACTGGGCAGACGCCATCCGCGAACTTCGCCAAGGTCGCGTGGTTATTTTTGCCGCAGGTACTGGCAACCCATTCTTCACTACGGATTCTGCTGCTTGCCTACGTGGTATTGAGATTGAAGCGGATGTAGTTCTAAAAGCGACTAAAGTGGATGGCGTATTTACTGCTGACCCGGTAGCCAACCCAGACGCAGAGCTGTATGATAAGCTTTCTTACACCGATGTTCTTGAAAAAGAACTGAAAGTGATGGATTTG | MN253431 |
| PKGK04 | *Vibrio vulnificus* | GGTGGCGGTAACTTGTTCCGTGGTGCTGGTCTAGCGCAAGCTGGTATGAACCGTGTTGTGGGTGACCACATGGGTATGCTAGCAACGGTAATGAATGGCCTAGCGATGCGCGATGCGCTACACCGTGCTTACGTAAATGCACGCGTGATGTCTGCAATTCCACTTAACGGTGTTTGTGATGACTACAACTGGGCAGACGCCATCCGCGAACTTCGCCAAGGTCGCGTGGTTATTTTTGCCGCAGGTACTGGCAACCCATTCTTCACTACGGATTCTGCTGCTTGCCTACGTGGTATTGAGATTGAAGCGGATGTAGTTCTAAAAGCGACTAAAGTGGATGGCGTATTTACTGCTGACCCGGTAGCCAACCCAGACGCAGAGCTGTATGATAAGCTTTCTTACACCGATGTTCTTGAAAAAGAACTGAAAGTGATGGATTTG | MN253432 |
| PKGS22 | *Vibrio vulnificus* | GGTGGCGGTAACTTGTTCCGTGGTGCTGGTCTAGCGCAAGCTGGTATGAACCGTGTTGTGGGTGACCACATGGGTATGCTAGCAACGGTAATGAATGGCCTAGCGATGCGCGATGCGCTACACCGTGCTTACGTAAATGCACGCGTGATGTCTGCAATTCCACTTAACGGTGTTTGTGATGACTACAACTGGGCAGACGCCATCCGCGAACTTCGCCAAGGTCGCGTGGTTATTTTTGCCGCAGGTACTGGCAACCCATTCTTCACTACGGATTCTGCGGCTTGCCTACGTGGTATTGAGATTGAAGCGGATGTCGTTCTAAAAGCGACTAAAGTGGATGGCGTATTTACTGCTGACCCGGTAGCCAACCCAGACGCAGAGCTGTATGATAAGCTTTCTTACACCGATGTTCTTGAAAAAGAACTGAAAGTGATGGATTTG | MN253433 |
| PKGK22 | *Vibrio vulnificus* | GGTGGCGGTAACTTGTTCCGTGGTGCTGGTCTAGCGCAAGCTGGTATGAACCGTGTTGTGGGTGACCACATGGGTATGCTAGCAACGGTAATGAATGGCCTAGCGATGCGCGATGCGCTACACCGTGCTTACGTAAATGCACGCGTGATGTCTGCAATTCCACTTAACGGTGTTTGTGATGACTACAACTGGGCAGACGCCATCCGCGAACTTCGCCAAGGTCGCGTGGTTATTTTTGCCGCAGGTACTGGCAACCCATTCTTCACTACGGATTCTGCGGCTTGCCTACGTGGTATTGAGATTGAAGCGGATGTCGTTCTAAAAGCGACTAAAGTGGATGGCGTATTTACTGCTGACCCGGTAGCCAACCCAGACGCAGAGCTGTATGATAAGCTTTCTTACACCGATGTTCTTGAAAAAGAACTGAAAGTGATGGATTTG | MN253434 |
| TK02 | *Vibrio vulnificus* | GGTGGCGGCAACTTGTTCCGTGGTGCTGGTCTTGCGCAAGCTGGTATGAACCGTGTTGTGGGTGACCACATGGGTATGCTTGCAACAGTAATGAATGGCCTAGCGATGCGCGATGCACTTCACCGTGCTTACGTAAATGCTCGCGTGATGTCTGCAATTCCACTTAACGGTGTTTGTGATGACTACAACTGGGCAGACGCTATCCGCGAACTTCGCCAAGGTCGCGTGGTTATTTTTGCCGCAGGTACTGGTAACCCATTCTTCACTACTGATTCTGCGGCTTGCCTACGTGGTATTGAGATTGAAGCGGATGTCGTTCTAAAGGCGACTAAAGTGGATGGCGTATTTACTGCTGACCCGGTAGCCAACCCAGACGCAGAGCTGTATGATAAGCTTTCTTACACCGATGTTCTTGAAAAAGAACTGAAAGTGATGGATTTG | MN253435 |
| TL05 | *Vibrio vulnificus* | GGTGGCGGTAACTTGTTCCGTGGTGCTGGTCTAGCGCAAGCTGGTATGAACCGTGTTGTGGGTGACCACATGGGTATGCTAGCAACGGTAATGAATGGCCTAGCGATGCGCGATGCGCTACACCGTGCTTACGTAAATGCACGCGTGATGTCTGCAATTCCACTTAACGGTGTTTGTGATGACTACAACTGGGCAGACGCTATCCGCGAACTTCGCCAAGGTCGCGTGGTTATTTTTGCCGCAGGTACTGGCAACCCATTCTTCACTACTGATTCTGCGGCTTGCCTACGTGGTATTGAGATTGAAGCGGATGTCGTTCTAAAAGCGACTAAAGTGGATGGCGTATTTACTGCTGACCCGGTAGCCAACCCAGACGCAGAGCTGTATGATAAGCTTTCTTACACCGATGTTCTTGAAAAAGAACTGAAAGTGATGGATTTG | MN253436 |
| TS06 | *Vibrio vulnificus* | GGTGGCGGTAACTTGTTCCGTGGTGCTGGTCTTGCGCAAGCTGGTATGAACCGTGTTGTGGGTGACCACATGGGTATGCTTGCAACAGTAATGAATGGCCTAGCGATGCGCGATGCGCTACACCGTGCTTACGTAAATGCTCGCGTGATGTCTGCAATTCCACTTAACGGTGTTTGTGATGACTACAACTGGGCAGACGCTATCCGCGAACTTCGCCAAGGTCGCGTGGTTATTTTTGCCGCAGGTACTGGTAACCCATTCTTCACTACTGATTCTGCGGCTTGCCTACGTGGTATTGAGATTGAAGCGGATGTCGTTCTAAAAGCGACTAAAGTGGATGGCGTATTTACTGCTGACCCGGTAGCCAACCCAGACGCAGAGCTGTATGATAAGCTTTCTTACACCGATGTTCTTGAAAAAGAACTGAAAGTGATGGATTTG | MN253437 |
| TK06 | *Vibrio vulnificus* | GGTGGCGGTAACTTGTTCCGTGGTGCTGGTCTTGCGCAAGCTGGTATGAACCGTGTTGTGGGTGACCACATGGGTATGCTAGCAACGGTAATGAATGGCCTAGCGATGCGCGATGCGCTACACCGTGCTTACGTAAATGCACGCGTGATGTCTGCAATTCCACTTAACGGTGTTTGTGATGACTACAACTGGGCAGACGCTATCCGCGAACTTCGCCAAGGTCGCGTGGTTATTTTTGCCGCAGGTACTGGTAACCCATTCTTCACTACTGATTCTGCGGCTTGCCTACGTGGTATTGAGATTGAAGCGGATGTCGTTCTAAAAGCGACTAAAGTGGATGGCGTATTTACTGCTGACCCGGTAGCCAACCCAGACGCAGAACTGTATGATAAGCTTTCTTACACCGATGTTCTTGAAAAAGAACTGAAAGTGATGGATTTG | MN253438 |
| TS09 | *Vibrio vulnificus* | GGTGGCGGTAACTTGTTCCGTGGTGCTGGTCTTGCGCAAGCTGGTATGAACCGTGTTGTGGGTGACCACATGGGTATGCTAGCAACGGTAATGAATGGCCTAGCGATGCGCGATGCGCTTCACCGTGCTTACGTAAATGCTCGCGTGATGTCTGCAATTCCACTTAACGGTGTTTGTGATGACTACAACTGGGCAGACGCTATCCGCGAACTTCGCCAAGGTCGCGTGGTTATTTTTGCCGCAGGTACTGGTAACCCATTCTTCACTACTGATTCTGCGGCTTGCCTACGTGGTATTGAAATTGAAGCTGATGTCGTTCTAAAAGCGACTAAAGTGGATGGCGTATTTACTGCTGACCCGGTAGCCAACCCAGACGCAGAACTGTATGATAAGCTTTCTTACACCGATGTTCTTGAAAAAGAACTGAAAGTGATGGATTTG | MN253439 |
| TS10 | *Vibrio vulnificus* | GGTGGCGGTAATCTGTTCCGTGGTGCTGGTCTAGCGCAAGCTGGTATGAACCGTGTTGTGGGTGACCACATGGGTATGCTAGCAACGGTAATGAATGGCCTAGCGATGCGCGATGCGCTACACCGTGCTTACGTAAATGCACGCGTGATGTCTGCAATTCCACTTAACGGTGTTTGTGATGACTACAACTGGGCAGACGCTATCCGCGAACTTCGCCAAGGTCGCGTGGTTATTTTTGCCGCAGGTACTGGTAACCCATTCTTCACTACTGATTCTGCGGCTTGCCTACGTGGTATTGAGATTGAAGCTGATGTAGTTCTAAAAGCGACTAAAGTGGATGGCGTATTTACTGCTGACCCGGTAGCCAACCCAGACGCAGAGCTGTATGATAAGCTTTCTTACACCGATGTTCTTGAAAAAGAACTGAAAGTGATGGATTTG | MN253440 |
| TK10 | *Vibrio vulnificus* | GGTGGTGGTAATTTATTCCGTGGTGCTGGTCTTGCGCAAGCTGGTATGAACCGTGTTGTGGGTGACCACATGGGTATGCTTGCAACAGTAATGAATGGCCTAGCGATGCGCGATGCGCTACACCGTGCTTACGTAAATGCTCGCGTGATGTCTGCAATTCCACTTAACGGTGTTTGTGATGACTACAACTGGGCAGACGCTATCCGCGAACTTCGCCAAGGTCGCGTGGTTATCTTCGCTGCAGGTACTGGTAACCCATTCTTCACTACTGATTCTGCGGCTTGCCTACGTGGTATTGAGATTGAAGCTGATGTCGTTCTAAAAGCGACTAAAGTGGATGGCGTATTTACTGCTGACCCGGTAGCCAACCCAGACGCAGAGCTGTATGATAAGCTTTCTTACACCGATGTTCTTGAAAAAGAACTGAAAGTGATGGATTTG | MN253441 |
| TS12 | *Vibrio vulnificus* | GGTGGCGGTAACTTGTTCCGTGGTGCTGGTCTAGCGCAAGCTGGTATGAACCGTGTTGTGGGTGACCACATGGGTATGCTAGCAACGGTAATGAATGGCCTAGCGATGCGCGATGCGCTACACCGTGCTTACGTAAATGCACGCGTGATGTCTGCAATTCCACTTAACGGTGTTTGTGATGACTACAACTGGGCAGACGCTATCCGCGAACTTCGCCAAGGTCGCGTGGTTATTTTTGCCGCAGGTACTGGCAACCCATTCTTCACTACTGATTCTGCTGCTTGCCTACGTGGTATTGAGATTGAAGCGGATGTAGTTCTAAAAGCGACTAAAGTGGATGGCGTATTTACTGCTGACCCGGTAGCCAACCCAGACGCAGAGCTGTATGATAAGCTTTCTTACACCGATGTTCTTGAAAAAGAACTGAAAGTGATGGATTTG | MN253442 |
| TS19 | *Vibrio vulnificus* | GGTGGCGGTAACTTGTTCCGTGGTGCTGGTCTAGCGCAAGCTGGTATGAACCGTGTTGTGGGTGACCACATGGGTATGCTAGCAACGGTAATGAATGGCCTAGCGATGCGCGATGCGCTACACCGTGCTTACGTAAATGCACGCGTGATGTCTGCAATTCCACTTAACGGTGTTTGTGATGACTACAACTGGGCAGACGCCATCCGCGAACTTCGCCAAGGTCGCGTGGTTATTTTTGCCGCAGGTACTGGCAACCCATTCTTCACTACGGATTCTGCTGCTTGCCTACGTGGTATTGAGATTGAAGCGGATGTAGTTCTAAAAGCGACTAAAGTGGATGGCGTATTTACTGCTGACCCGGTAGCCAACCCAGACGCAGAACTGTATGATAAGCTTTCTTACACCGATGTTCTTGAAAAAGAACTGAAAGTGATGGATTTG | MN253443 |
| TS22 | *Vibrio vulnificus* | GGTGGCGGTAACTTGTTCCGTGGTGCTGGTCTAGCGCAAGCTGGTATGAACCGTGTTGTGGGTGACCACATGGGTATGCTAGCAACGGTAATGAATGGCCTAGCGATGCGCGATGCGCTACACCGTGCTTACGTAAATGCACGCGTGATGTCTGCAATTCCACTTAACGGTGTTTGTGATGACTACAACTGGGCAGACGCCATCCGCGAACTTCGCCAAGGTCGCGTGGTTATTTTTGCCGCAGGTACTGGCAACCCATTCTTCACTACGGATTCTGCTGCTTGCCTACGTGGTATTGAGATTGAAGCGGATGTAGTTCTAAAAGCGACTAAAGTGGATGGCGTATTTACTGCTGACCCGGTAGCCAACCCAGACGCAGAGCTGTATGATAAGCTTTCTTACACCGATGTTCTTGAAAAAGAACTGAAAGTGATGGATTTG | MN253444 |
| TL28 | *Vibrio vulnificus* | GGTGGCGGTAACTTGTTCCGTGGTGCTGGTCTAGCGCAAGCTGGTATGAACCGTGTTGTGGGTGACCACATGGGTATGCTAGCAACGGTAATGAATGGCCTAGCGATGCGCGATGCGCTACACCGTGCTTACGTAAATGCACGCGTGATGTCTGCAATTCCACTTAACGGTGTTTGTGATGACTACAACTGGGCAGACGCTATCCGCGAACTTCGCCAAGGTCGCGTGGTTATTTTTGCCGCAGGTACTGGCAACCCATTCTTCACTACTGATTCTGCGGCTTGCCTACGTGGTATTGAGATTGAAGCGGATGTCGTTCTAAAAGCGACTAAAGTGGATGGCGTATTTACTGCTGACCCGGTAGCCAACCCAGACGCAGAGCTGTATGATAAGCTTTCTTACACCGATGTTCTTGAAAAAGAACTGAAAGTGATGGATTTG | MN253445 |
| BK03 | *Vibrio vulnificus* | GGTGGCGGTAACTTGTTCCGTGGTGCTGGTCTAGCGCAAGCTGGTATGAACCGTGTTGTGGGTGACCACATGGGTATGCTAGCAACGGTAATGAATGGCCTAGCGATGCGCGATGCGCTACACCGTGCTTACGTAAATGCACGCGTGATGTCTGCAATTCCACTTAACGGTGTTTGTGATGACTACAACTGGGCAGACGCTATCCGCGAACTTCGCCAAGGTCGCGTGGTTATTTTTGCCGCAGGTACTGGTAACCCATTCTTCACTACTGATTCTGCGGCTTGCCTACGTGGTATTGAGATTGAAGCGGATGTCGTTCTAAAAGCGACTAAGGTGGATGGCGTATTTACTGCTGACCCGGTAGCCAACCCAGACGCAGAACTGTATGATAAGCTTTCTTACACCGATGTTCTTGAAAAAGAACTGAAAGTGATGGATTTG | MN253446 |
| NL06 | *Vibrio vulnificus* | GGTGGCGGTAACTTGTTCCGTGGTGCTGGTCTAGCGCAAGCTGGTATGAACCGTGTTGTGGGTGACCACATGGGTATGCTAGCAACGGTAATGAATGGCCTAGCGATGCGCGATGCGCTACACCGTGCTTACGTAAATGCACGCGTGATGTCTGCAATTCCACTTAACGGTGTTTGTGATGACTACAACTGGGCAGACGCTATCCGCGAACTTCGCCAAGGTCGCGTGGTTATTTTTGCCGCAGGTACTGGCAACCCATTCTTCACTACTGATTCTGCGGCTTGCCTACGTGGTATTGAGATTGAAGCGGATGTCGTTCTAAAAGCGACTAAAGTGGATGGCGTATTTACTGCTGACCCGGTAGCCAACCCAGACGCAGAGCTGTATGATAAGCTTTCTTACACCGATGTTCTTGAAAAAGAACTGAAAGTGATGGATTTG | MN253447 |
| NL11 | *Vibrio vulnificus* | GGTGGCGGTAACTTGTTCCGTGGTGCTGGTCTAGCGCAAGCTGGTATGAACCGTGTTGTGGGTGACCACATGGGTATGCTAGCAACGGTAATGAATGGCCTAGCGATGCGCGATGCGCTACACCGTGCTTACGTAAATGCACGCGTGATGTCTGCAATTCCACTTAACGGTGTTTGTGATGACTACAACTGGGCAGACGCTATCCGCGAACTTCGCCAAGGTCGCGTGGTTATTTTTGCCGCAGGTACTGGCAACCCATTCTTCACTACTGATTCTGCGGCTTGCCTACGTGGTATTGAGATTGAAGCGGATGTCGTTCTAAAAGCGACTAAAGTGGATGGCGTATTTACTGCTGACCCGGTAGCCAACCCAGACGCAGAGCTGTATGATAAGCTTTCTTACACCGATGTTCTTGAAAAAGAACTGAAAGTGATGGATTTG | MN253448 |
| NS11 | *Vibrio vulnificus* | GGTGGCGGTAACTTGTTCCGTGGTGCTGGTCTAGCGCAAGCTGGTATGAACCGTGTTGTGGGTGACCACATGGGTATGCTAGCAACGGTAATGAATGGCCTAGCGATGCGCGATGCGCTACACCGTGCTTACGTAAATGCACGCGTGATGTCTGCAATTCCACTTAACGGTGTTTGTGATGACTACAACTGGGCAGACGCTATCCGCGAACTTCGCCAAGGTCGCGTGGTTATTTTTGCCGCAGGTACTGGCAACCCATTCTTCACTACTGATTCTGCGGCTTGCCTACGTGGTATTGAGATTGAAGCGGATGTCGTTCTAAAAGCGACTAAAGTGGATGGCGTATTTACTGCTGACCCGGTAGCCAACCCAGACGCAGAGCTGTATGATAAGCTTTCTTACACCGATGTTCTTGAAAAAGAACTGAAAGTGATGGATTTG | MN253449 |
| NK11 | *Vibrio vulnificus* | GGTGGCGGTAACTTGTTCCGTGGTGCTGGTCTAGCGCAAGCTGGTATGAACCGTGTTGTGGGTGACCACATGGGTATGCTAGCAACGGTAATGAATGGCCTAGCGATGCGCGATGCGCTACACCGTGCTTACGTAAATGCACGCGTGATGTCTGCAATTCCACTTAACGGTGTTTGTGATGACTACAACTGGGCAGACGCTATCCGCGAACTTCGCCAAGGTCGCGTGGTTATTTTTGCCGCAGGTACTGGCAACCCATTCTTCACTACTGATTCTGCGGCTTGCCTACGTGGTATTGAGATTGAAGCGGATGTCGTTCTAAAAGCGACTAAAGTGGATGGCGTATTTACTGCTGACCCGGTAGCCAACCCAGACGCAGAGCTGTATGATAAGCTTTCTTACACCGATGTTCTTGAAAAAGAACTGAAAGTGATGGATTTG | MN253450 |
| NS13 | *Vibrio vulnificus* | GGTGGCGGTAACTTGTTCCGTGGCGCTGGTCTAGCGCAAGCTGGTATGAACCGTGTTGTGGGTGACCACATGGGTATGCTAGCAACGGTAATGAATGGCCTAGCGATGCGCGATGCGCTACACCGTGCTTACGTAAATGCACGCGTGATGTCTGCAATTCCACTTAACGGTGTTTGTGATGACTACAACTGGGCAGACGCTATCCGCGAACTTCGCCAAGGTCGCGTGGTTATTTTTGCCGCAGGTACTGGCAACCCATTCTTCACTACTGATTCTGCGGCTTGCCTACGTGGTATTGAGATTGAAGCGGATGTCGTTCTAAAAGCGACTAAAGTGGATGGCGTATTTACTGCTGACCCGGTAGCCAACCCAGACGCAGAGCTGTATGATAAGCTTTCTTACACCGATGTTCTTGAAAAAGAACTGAAAGTGATGGATTTG | MN253451 |
| NL14 | *Vibrio vulnificus* | GGTGGCGGTAACTTGTTCCGTGGTGCTGGTCTAGCGCAAGCTGGTATGAACCGTGTTGTGGGTGACCACATGGGTATGCTAGCAACGGTAATGAATGGCCTAGCGATGCGCGATGCGCTACACCGTGCTTACGTAAATGCACGCGTGATGTCTGCAATTCCACTTAACGGTGTTTGTGATGACTACAACTGGGCAGACGCTATCCGCGAACTTCGCCAAGGTCGCGTGGTTATTTTTGCCGCAGGTACTGGCAACCCATTCTTCACTACTGATTCTGCGGCTTGCCTACGTGGTATTGAGATTGAAGCGGATGTCGTTCTAAAAGCGACTAAAGTGGATGGCGTATTTACTGCTGACCCGGTAGCCAACCCAGACGCAGAGCTGTATGATAAGCTTTCTTACACCGATGTTCTTGAAAAAGAACTGAAAGTGATGGATTTG | MN253452 |
| NL15 | *Vibrio vulnificus* | GGTGGCGGTAACTTGTTCCGTGGCGCAGGCCTAGCGCAAGCTGGTATGAACCGTGTTGTGGGTGACCACATGGGTATGCTAGCAACGGTAATGAATGGCCTAGCGATGCGCGATGCGCTACACCGTGCTTACGTAAATGCACGCGTGATGTCTGCAATTCCACTTAACGGTGTTTGTGATGACTACAACTGGGCAGACGCTATCCGCGAACTTCGCCAAGGTCGCGTGGTTATTTTTGCCGCAGGTACTGGCAACCCATTCTTCACTACTGATTCTGCGGCTTGCCTACGTGGTATTGAGATTGAAGCGGATGTCGTTCTAAAAGCGACTAAAGTGGATGGCGTATTTACTGCTGACCCGGTAGCCAACCCAGACGCAGAGCTGTATGATAAGCTTTCTTACACCGATGTTCTTGAAAAAGAACTGAAAGTGATGGATTTG | MN253453 |
| NL18 | *Vibrio vulnificus* | GGTGGCGGTAACTTGTTCCGTGGCGCTGGTCTAGCGCAAGCAGGCATGAACCGTGTTGTGGGTGACCACATGGGGATGCTAGCAACGGTGATGAACGGCCTAGCGATGCGCGATGCGCTACACCGTGCTTACGTAAATGCACGCGTGATGTCAGCTATCCCACTTAACGGAGTGTGCGACGACTACAATTGGGCAGACGCTATCCGCGAACTTCGCCAAGGTCGCGTGGTTATCTTTGCTGCAGGTACTGGCAACCCATTCTTCACTACGGATTCTGCTGCTTGCCTACGTGGTATTGAGATTGAAGCGGATGTCGTTTTAAAAGCGACTAAAGTGGATGGCGTATTTACTGCTGACCCGGTAGCCAACCCAGACGCAGAGCTGTATGATAAGCTTTCTTATACCGATGTTCTTGAAAAAGAACTGAAAGTGATGGATTTG | MN253454 |
| NL21 | *Vibrio vulnificus* | GGTGGCGGTAACTTGTTCCGTGGTGCTGGTCTAGCGCAAGCTGGTATGAACCGTGTTGTGGGTGACCACATGGGTATGCTAGCAACGGTAATGAATGGCCTAGCGATGCGCGATGCGCTACACCGTGCTTACGTAAATGCACGCGTGATGTCTGCAATTCCACTTAACGGTGTTTGTGATGACTACAACTGGGCAGACGCTATCCGCGAACTTCGCCAAGGTCGCGTGGTTATTTTTGCCGCAGGTACTGGCAACCCATTCTTCACTACTGATTCTGCGGCTTGCCTACGTGGTATTGAGATTGAAGCGGATGTCGTTCTAAAAGCGACTAAAGTGGATGGCGTATTTACTGCTGACCCGGTAGCCAACCCAGACGCAGAGCTGTATGATAAGCTTTCTTACACCGATGTTCTTGAAAAAGAACTGAAAGTGATGGATTTG | MN253455 |
| NL26 | *Vibrio vulnificus* | GGTGGCGGTAACCTGTTCCGTGGTGCTGGTCTAGCGCAAGCTGGTATGAACCGTGTTGTGGGTGACCACATGGGTATGCTAGCAACGGTAATGAATGGCCTAGCGATGCGCGATGCGCTACACCGTGCTTACGTAAATGCACGCGTGATGTCTGCAATTCCACTTAACGGTGTTTGTGATGACTACAACTGGGCAGACGCTATCCGCGAACTTCGCCAAGGTCGCGTGGTTATTTTTGCCGCAGGTACTGGCAACCCATTCTTCACTACTGATTCTGCGGCTTGCCTACGTGGTATTGAGATTGAAGCGGATGTCGTTCTAAAAGCGACTAAAGTGGATGGCGTATTTACTGCTGACCCGGTAGCCAACCCAGACGCAGAGCTGTATGATAAGCTTTCTTACACCGATGTTCTTGAAAAAGAACTGAAAGTGATGGATTTG | MN253456 |
| JK05 | *Vibrio vulnificus* | GGTGGCGGTAACTTGTTCCGTGGTGCTGGTCTAGCGCAAGCTGGTATGAACCGTGTTGTGGGTGACCACATGGGTATGCTAGCAACGGTAATGAATGGCCTAGCGATGCGCGATGCGCTACACCGTGCTTACGTAAATGCACGCGTGATGTCTGCAATTCCACTTAACGGTGTTTGTGATGACTACAACTGGGCAGACGCTATCCGCGAACTTCGCCAAGGTCGCGTGGTTATTTTTGCCGCAGGTACTGGCAACCCATTCTTCACTACTGATTCTGCGGCTTGCCTACGTGGTATTGAGATTGAAGCGGATGTCGTTCTAAAAGCGACTAAAGTGGATGGCGTATTTACTGCTGACCCGGTAGCCAACCCAGACGCAGAGCTGTATGATAAGCTTTCTTACACCGATGTTCTTGAAAAAGAACTGAAAGTGATGGATTTG | MN253457 |
| JS07 | *Vibrio vulnificus* | GGTGGCGGTAACTTGTTCCGTGGTGCTGGTCTAGCGCAAGCTGGTATGAACCGTGTTGTGGGTGACCACATGGGTATGCTAGCAACGGTAATGAATGGCCTAGCGATGCGCGATGCGCTACACCGTGCTTACGTAAATGCACGCGTGATGTCTGCAATTCCACTTAACGGTGTTTGTGATGACTACAACTGGGCAGACGCTATCCGCGAACTTCGCCAAGGTCGCGTGGTTATTTTTGCCGCAGGTACTGGCAACCCATTCTTCACTACTGATTCTGCGGCTTGCCTACGTGGTATTGAGATTGAAGCGGATGTCGTTCTAAAAGCGACTAAAGTGGATGGCGTATTTACTGCTGACCCGGTAGCCAACCCAGACGCAGAGCTGTATGATAAGCTTTCTTACACCGATGTTCTTGAAAAAGAACTGAAAGTGATGGATTTG | MN253458 |
| JS08 | *Vibrio vulnificus* | GGTGGCGGTAACTTGTTCCGTGGTGCTGGTCTAGCGCAAGCTGGTATGAACCGTGTTGTGGGTGACCACATGGGTATGCTAGCAACGGTAATGAATGGCCTAGCGATGCGCGATGCGCTACACCGTGCTTACGTAAATGCACGCGTGATGTCTGCAATTCCACTTAACGGTGTTTGTGATGACTACAACTGGGCAGACGCTATCCGCGAACTTCGCCAAGGTCGCGTGGTTATTTTTGCCGCAGGTACTGGCAACCCATTCTTCACTACTGATTCTGCGGCTTGCCTACGTGGTATTGAGATTGAAGCGGATGTCGTTCTAAAAGCGACTAAAGTGGATGGCGTATTTACTGCTGACCCGGTAGCCAACCCAGACGCAGAGCTGTATGATAAGCTTTCTTACACCGATGTTCTTGAAAAAGAACTGAAAGTGATGGATTTG | MN253459 |
| JK08 | *Vibrio vulnificus* | GGTGGCGGTAACTTGTTCCGTGGTGCTGGTCTAGCGCAAGCTGGTATGAACCGTGTTGTGGGTGACCACATGGGTATGCTAGCAACGGTAATGAATGGCCTAGCGATGCGCGATGCGCTACACCGTGCTTACGTAAATGCACGCGTGATGTCTGCAATTCCACTTAACGGTGTTTGTGATGACTACAACTGGGCAGACGCTATCCGCGAACTTCGCCAAGGTCGCGTGGTTATTTTTGCCGCAGGTACTGGCAACCCATTCTTCACTACTGATTCTGCGGCTTGCCTACGTGGTATTGAGATTGAAGCGGATGTCGTTCTAAAAGCGACTAAAGTGGATGGCGTATTTACTGCTGACCCGGTAGCCAACCCAGACGCAGAGCTGTATGATAAGCTTTCTTACACCGATGTTCTTGAAAAAGAACTGAAAGTGATGGATTTG | MN253460 |
| JS09 | *Vibrio vulnificus* | GGTGGCGGTAACTTGTTCCGTGGTGCTGGTCTAGCGCAAGCTGGTATGAACCGTGTTGTGGGTGACCACATGGGTATGCTAGCAACGGTAATGAATGGCCTAGCGATGCGCGATGCGCTACACCGTGCTTACGTAAATGCACGCGTGATGTCTGCAATTCCACTTAACGGTGTTTGTGATGACTACAACTGGGCAGACGCTATCCGCGAACTTCGCCAAGGTCGCGTGGTTATTTTTGCCGCAGGTACTGGCAACCCATTCTTCACTACTGATTCTGCGGCTTGCCTACGTGGTATTGAGATTGAAGCGGATGTCGTTCTAAAAGCGACTAAAGTGGATGGCGTATTTACTGCTGACCCGGTAGCCAACCCAGACGCAGAGCTGTATGATAAGCTTTCTTACACCGATGTTCTTGAAAAAGAACTGAAAGTGATGGATTTG | MN253461 |
| JS12 | *Vibrio vulnificus* | GGTGGCGGTAACTTGTTCCGTGGTGCTGGTCTAGCGCAAGCTGGTATGAACCGTGTTGTGGGTGACCACATGGGTATGCTAGCAACGGTAATGAATGGCCTAGCGATGCGCGATGCGCTACACCGTGCTTACGTAAATGCACGCGTGATGTCTGCAATTCCACTTAACGGTGTTTGTGATGACTACAACTGGGCAGACGCTATCCGCGAACTTCGCCAAGGTCGCGTGGTTATTTTTGCCGCAGGTACTGGCAACCCATTCTTCACTACTGATTCTGCGGCTTGCCTACGTGGTATTGAGATTGAAGCGGATGTCGTTCTAAAAGCGACTAAAGTGGATGGCGTATTTACTGCTGACCCGGTAGCCAACCCAGACGCAGAGCTGTATGATAAGCTTTCTTACACCGATGTTCTTGAAAAAGAACTGAAAGTGATGGATTTG | MN253462 |
| JK12 | *Vibrio vulnificus* | GGTGGCGGTAACTTGTTCCGTGGTGCTGGTCTAGCGCAAGCTGGTATGAACCGTGTTGTGGGTGACCACATGGGTATGCTAGCAACGGTAATGAATGGCCTAGCGATGCGCGATGCGCTACACCGTGCTTACGTAAATGCACGCGTGATGTCTGCAATTCCACTTAACGGTGTTTGTGATGACTACAACTGGGCAGACGCTATCCGCGAACTTCGCCAAGGTCGCGTGGTTATTTTTGCCGCAGGTACTGGCAACCCATTCTTCACTACTGATTCTGCGGCTTGCCTACGTGGTATTGAGATTGAAGCGGATGTCGTTCTAAAAGCGACTAAAGTGGATGGCGTATTTACTGCTGACCCGGTAGCCAACCCAGACGCAGAGCTGTATGATAAGCTTTCTTACACCGATGTTCTTGAAAAAGAACTGAAAGTGATGGATTTG | MN253463 |
| JS19 | *Vibrio vulnificus* | GGTGGCGGTAACTTGTTCCGTGGTGCTGGTCTAGCGCAAGCTGGTATGAACCGTGTTGTGGGTGACCACATGGGTATGCTAGCAACGGTAATGAATGGCCTAGCGATGCGCGATGCGCTACACCGTGCTTACGTAAATGCACGCGTGATGTCTGCAATTCCACTTAACGGTGTTTGTGATGACTACAACTGGGCAGACGCTATCCGCGAACTTCGCCAAGGTCGCGTGGTTATTTTTGCCGCAGGTACTGGCAACCCATTCTTCACTACTGATTCTGCGGCTTGCCTACGTGGTATTGAGATTGAAGCGGATGTCGTTCTAAAAGCGACTAAAGTGGATGGCGTATTTACTGCTGACCCGGTAGCCAACCCAGACGCAGAGCTGTATGATAAGCTTTCTTACACCGATGTTCTTGAAAAAGAACTGAAAGTGATGGATTTG | MN253464 |
| JS20 | *Vibrio vulnificus* | GGTGGCGGTAACCTGTTCCGTGGTGCTGGTCTAGCGCAAGCTGGTATGAACCGTGTTGTGGGTGACCACATGGGTATGCTAGCAACGGTAATGAATGGCCTAGCGATGCGCGATGCGCTACACCGTGCTTACGTAAATGCACGCGTGATGTCTGCAATTCCACTTAACGGTGTTTGTGATGACTACAACTGGGCAGACGCTATCCGCGAACTTCGCCAAGGTCGCGTGGTTATTTTTGCCGCAGGTACTGGCAACCCATTCTTCACTACTGATTCTGCGGCTTGCCTACGTGGTATTGAGATTGAAGCGGATGTCGTTCTAAAAGCGACTAAAGTGGATGGCGTATTTACTGCTGACCCGGTAGCCAACCCAGACGCAGAGCTGTATGATAAGCTTTCTTACACCGATGTTCTTGAAAAAGAACTGAAAGTGATGGATTTG | MN253465 |
| JS21 | *Vibrio vulnificus* | GGTGGCGGTAACTTGTTCCGTGGTGCTGGTCTAGCGCAAGCTGGTATGAACCGTGTTGTGGGTGACCACATGGGTATGCTAGCAACGGTAATGAATGGCCTAGCGATGCGCGATGCGCTACACCGTGCTTACGTAAATGCACGCGTGATGTCTGCAATTCCACTTAACGGTGTTTGTGATGACTACAACTGGGCAGACGCTATCCGCGAACTTCGCCAAGGTCGCGTGGTTATTTTTGCCGCAGGTACTGGCAACCCATTCTTCACTACTGATTCTGCGGCTTGCCTACGTGGTATTGAGATTGAAGCGGATGTCGTTCTAAAAGCGACTAAAGTGGATGGCGTATTTACTGCTGACCCGGTAGCCAACCCAGACGCAGAGCTGTATGATAAGCTTTCTTACACCGATGTTCTTGAAAAAGAACTGAAAGTGATGGATTTG | MN253466 |
| JS22 | *Vibrio vulnificus* | GGTGGCGGTAACTTGTTCCGTGGTGCTGGTCTAGCGCAAGCTGGTATGAACCGTGTTGTGGGTGACCACATGGGTATGCTAGCAACGGTAATGAATGGCCTAGCGATGCGCGATGCGCTACACCGTGCTTACGTAAATGCACGCGTGATGTCTGCAATTCCACTTAACGGTGTTTGTGATGACTACAACTGGGCAGACGCTATCCGCGAACTTCGCCAAGGTCGCGTGGTTATTTTTGCCGCAGGTACTGGCAACCCATTCTTCACTACTGATTCTGCGGCTTGCCTACGTGGTATTGAGATTGAAGCGGATGTCGTTCTAAAAGCGACTAAAGTGGATGGCGTATTTACTGCTGACCCGGTAGCCAACCCAGACGCAGAGCTGTATGATAAGCTTTCTTACACCGATGTTCTTGAAAAAGAACTGAAAGTGATGGATTTG | MN253467 |
| LL18 | *Vibrio* sp. | GGTGGTGGTAACCTTTTCCGTGGTGCAGGTCTTGCGGAAGCGGGCATGAACCGCGTAGTCGGCGACCACATGGGTATGCTAGCAACAGTAATGAACGGCCTAGCGATGCGTGATGCTCTACACCGTGCTTACGTGAATGCTCGCGTAATGTCTGCTATTCCTCTAAAAGGCGTATGTGACGATTACAACTGGGCAGATGCGATTCGCGAACTGCGCCAAGGTCGTGTTGTTATCTTCTCTGCAGGTACAGGAAACCCATTCTTTACAACCGATTCTGCGGCATGTTTACGCGGTATTGAAATCGAAGCTGACGTAGTTCTAAAAGCAACAAAAGTAGATGGCGTATTTACCGCAGACCCAGTAGCAAACCCAGACGCAGAGCTGTATGATAAGCTTTCATATGCTGAGGTTCTTGACAAAGAATTGAAAGTAATGGATTTG | MN253468 |
| PJK23 | *Vibrio* sp. | GGTGGTGGTAACCTTTTCCGTGGCGCAGGTCTAGCTGAAGCGGGTATGAACCGAGTTGTGGGCGACCACATGGGTATGCTAGCTACAGTAATGAATGGTCTAGCAATGCGCGATGCACTACACCGTGCTTACGTAAATGCTCGTGTAATGTCAGCAATTCCTTTGAAAGGTGTGTGTGACGACTACAACTGGGCTGATGCTATCCGCGAACTTCGCCAAGGCCGCGTGGTTATCTTCTCAGCAGGTACTGGTAACCCATTCTTCACGACAGATTCTGCTGCTTGTTTACGCGGTATTGAAATTGAAGCGGACGTAGTTCTAAAAGCGACAAAAGTGGATGGCGTATTTACTGCTGACCCTGTAGCAAACCCAGACGCAGAACTGTATGATAAGCTTTCATATGCTGAAGTTCTTGATAAAGAACTGAAAGTGATGGATTTG | MN253469 |
| PJS27 | *Vibrio* sp. | GGTGGTGGTAACCTTTTCCGTGGTGCAGGTCTTGCGGAAGCGGGCATGAACCGCGTAGTCGGCGACCACATGGGTATGCTAGCAACAGTAATGAACGGCCTAGCGATGCGTGATGCTCTGCATCGTGCTTACGTGAATGCTCGCGTGATGTCTGCTATTCCTCTGAAAGGTGTGTGTGACGATTACAACTGGGCAGATGCTATTCGCGAACTGCGTCAAGGCCGCGTTGTTATCTTCTCTGCTGGTACAGGTAACCCATTCTTTACAACCGATTCTGCGGCATGTTTACGCGGTATCGAAATCGAAGCTGACGTAGTTCTAAAAGCAACAAAAGTAGATGGCGTATTTACCGCAGACCCAGTAGCAAACCCAGACGCAGAGCTGTATGATAAGCTTTCTTACGCTGAGGTTCTTGACAAAGAATTGAAAGTAATGGATTTG | MN253470 |
| KL20 | *Vibrio* sp. | GGTGGTGGTAACCTTTTCCGTGGCGCAGGTCTAGCTGAAGCGGGTATGAACCGAGTTGTGGGCGACCACATGGGTATGCTAGCTACAGTAATGAATGGTCTGGCAATGCGCGATGCACTACACCGTGCTTACGTAAATGCTCGTGTAATGTCAGCAATTCCTTTGAAAGGTGTGTGTGACGACTACAACTGGGCTGATGCTATCCGCGAACTTCGCCAAGGCCGCGTGGTTATCTTCTCAGCAGGTACTGGTAACCCATTCTTCACGACAGATTCTGCTGCTTGTTTACGCGGTATTGAAATTGAAGCGGACGTAGTTCTAAAAGCGACAAAAGTGGATGGCGTATTTACTGCTGACCCTGTAGCAAACCCAGACGCAGAACTGTATGATAAGCTTTCATATGCTGAAGTTCTTGATAAAGAACTGAAAGTGATGGATTTG | MN253471 |
| KK20 | *Vibrio* sp. | GGTGGTGGTAACCTTTTCCGTGGCGCAGGTCTAGCTGAAGCGGGTATGAACCGAGTTGTGGGTGACCACATGGGTATGCTAGCTACAGTAATGAATGGTCTAGCAATGCGCGATGCACTACACCGTGCTTACGTAAATGCTCGTGTAATGTCAGCAATTCCTTTGAAAGGTGTGTGTGACGACTACAACTGGGCTGATGCTATTCGCGAACTTCGCCAAGGCCGCGTGGTTATCTTCTCAGCAGGTACTGGTAACCCATTCTTCACGACAGATTCTGCTGCTTGTTTGCGCGGTATTGAAATTGAAGCGGACGTAGTTCTAAAAGCGACAAAAGTGGATGGCGTATTTACTGCTGACCCTGTAGCAAACCCAGACGCAGAACTGTATGATAAGCTTTCATATGCTGAAGTTCTTGATAAAGAACTGAAAGTGATGGATTTG | MN253472 |
| KL22 | *Vibrio* sp. | GGTGGTGGTAACCTTTTCCGTGGCGCAGGTCTAGCTGAAGCGGGTATGAACCGAGTTGTGGGTGACCACATGGGTATGCTAGCTACAGTAATGAATGGTCTAGCAATGCGCGATGCACTACACCGTGCTTACGTAAATGCTCGTGTAATGTCAGCAATTCCTTTGAAAGGTGTGTGTGACGACTACAACTGGGCTGATGCTATTCGCGAACTTCGCCAAGGCCGCGTGGTTATCTTCTCAGCAGGTACTGGTAACCCATTCTTCACGACAGATTCTGCTGCTTGTTTGCGCGGTATTGAAATTGAAGCGGACGTAGTTCTAAAAGCGACAAAAGTGGATGGCGTATTTACTGCTGACCCTGTAGCAAACCCAGACGCAGAACTGTATGATAAGCTTTCATATGCTGAAGTTCTTGATAAAGAACTGAAAGTGATGGATTTG | MN253473 |
| KS23 | *Vibrio* sp. | GGTGGTGGTAACCTTTTCCGTGGCGCAGGTCTAGCTGAAGCGGGTATGAACCGAGTTGTGGGCGACCACATGGGTATGCTAGCTACAGTAATGAATGGTCTGGCAATGCGCGATGCACTACACCGTGCTTACGTAAATGCTCGTGTAATGTCAGCAATTCCTTTGAAAGGTGTGTGTGACGACTACAACTGGGCTGATGCTATCCGCGAACTTCGCCAAGGCCGCGTGGTTATCTTCTCAGCAGGTACTGGTAACCCATTCTTCACGACAGATTCTGCTGCTTGTTTACGCGGTATTGAAATTGAAGCGGACGTAGTTCTAAAAGCGACAAAAGTGGATGGCGTATTTACTGCTGACCCTGTAGCAAACCCAGACGCAGAACTGTATGATAAGCTTTCATATGCTGAAGTTCTTGATAAAGAACTGAAAGTGATGGATTTG | MN253474 |
| KL24 | *Vibrio* sp. | GGTGGTGGTAACCTTTTCCGTGGCGCAGGTCTAGCTGAAGCGGGTATGAACCGAGTTGTGGGTGACCACATGGGTATGCTAGCTACAGTAATGAATGGTCTAGCAATGCGCGATGCACTACACCGTGCTTACGTAAATGCTCGTGTAATGTCAGCAATTCCTTTGAAAGGTGTGTGTGACGACTACAACTGGGCTGATGCTATTCGCGAACTTCGCCAAGGCCGCGTGGTTATCTTCTCAGCAGGTACTGGTAACCCATTCTTCACGACAGATTCTGCTGCTTGTTTGCGCGGTATTGAAATTGAAGCGGACGTAGTTCTAAAAGCGACAAAAGTGGATGGCGTATTTACTGCTGACCCTGTAGCAAACCCAGACGCAGAACTGTATGATAAGCTTTCATATGCTGAAGTTCTTGATAAAGAACTGAAAGTGATGGATTTG | MN253475 |
| KL30 | *Vibrio* sp. | GGTGGTGGTAACCTTTTCCGTGGCGCAGGTCTAGCTGAAGCGGGTATGAACCGAGTTGTGGGCGACCACATGGGTATGCTAGCTACAGTAATGAATGGTCTGGCAATGCGCGATGCACTACACCGTGCTTACGTAAATGCTCGTGTAATGTCAGCAATTCCTTTGAAAGGTGTGTGTGACGACTACAACTGGGCTGATGCTATCCGCGAACTTCGCCAAGGCCGCGTGGTTATCTTCTCAGCAGGTACTGGTAACCCATTCTTCACGACAGATTCTGCTGCTTGTTTACGCGGTATTGAAATTGAAGCGGACGTAGTTCTAAAAGCGACAAAAGTGGATGGCGTATTTACTGCTGACCCTGTAGCAAACCCAGACGCAGAACTGTATGATAAGCTTTCATATGCTGAAGTTCTTGATAAAGAACTGAAAGTGATGGATTTG | MN253476 |
| TL29 | *Vibrio* sp. | GGTGGTGGTAACCTTTTCCGTGGCGCAGGTCTAGCTGAAGCGGGTATGAACCGAGTTGTGGGTGACCACATGGGTATGCTAGCTACAGTAATGAATGGTCTAGCAATGCGCGATGCACTACACCGTGCTTACGTAAATGCTCGTGTAATGTCAGCAATTCCTTTGAAAGGTGTGTGTGACGACTACAACTGGGCTGATGCTATCCGCGAACTTCGCCAAGGCCGCGTGGTTATCTTCTCAGCAGGTACTGGTAACCCATTCTTCACGACAGATTCTGCTGCTTGTTTGCGCGGTATTGAAATTGAAGCGGACGTAGTTCTAAAAGCGACAAAAGTGGATGGCGTATTTACTGCTGACCCTGTAGCAAACCCAGACGCAGAACTGTATGATAAGCTTTCATATGCTGAAGTTCTTGATAAAGAACTGAAAGTGATGGATTTG | MN253477 |
| PKL21 | *Vibrio* sp. | GGTGGTGGTAACCTGTTCCGTGGTGCAGGTCTTGCTGAAGCGGGTATGAACCGCGTAGTGGGTGACCACATGGGTATGCTTGCTACAGTAATGAATGGTCTAGCAATGCGCGATGCACTACACCGTGCTTACGTAAATGCTCGCGTAATGTCTGCAATTCCTCTGAAAGGTGTGTGTGACGACTACAACTGGGCTGATGCTATCCGCGAACTTCGCCAAGGCCGCGTGGTTATTTTCTCTGCAGGTACTGGTAACCCATTCTTCACTACTGATTCCGCTGCTTGTTTGCGCGGTATTGAAATTGAAGCGGACGTAGTTCTAAAAGCGACAAAAGTGGATGGCGTATTTACTGCTGACCCTGTAGCAAACCCAGACGCAGAACTGTATGATAAGCTTTCATATGCTGAAGTTCTTGATAAAGAACTGAAAGTGATGGATTTG | MN253478 |
